# Supplementary material for: Integrated analysis of oral tongue squamous cell carcinoma identifies key variants and pathways linked to risk habits, HPV, clinical parameters and tumor recurrence
Source: F1000Res. 2015 Nov 5;4:1215. [Version 1] doi: 10.12688/f1000research.7302.1 (PMC4706066; doi:10.12688/f1000research.7302.1)
Supplement: Supplementary file 1 [file f1000research-4-7870-s0000.tgz › 4007fd05-e466-4e59-a4ef-512f9d41f650.zip!/Additional files/Additional file 14.doc]

**Supplementary Methods**

*1. Exome sequencing library preparation*

*a. Agilent*

The libraries for the samples were prepared as per “SureSelectXT Target Enrichment System for Illumina Paired-and Sequencing Library” protocol. 3 µg of high quality genomic DNA extracted from the samples were sheared to a peak size of 150 to 200 bp using the Covaris S2. Fragmented DNA was purified using Agencourt AMPure XP beads, end repaired, A-Tailed and adaptor ligated with SureSelect Adaptor Oligos. Post adaptor ligation the libraries were amplified for 6 cycles (with Sureselect ILM Indexing Pre Capture PCR Reverse Primer). The libraries post purification with Ampure XP beads were quantified using Qubit and its quality was assessed on a Agilent 2100 Bioanalyzer using DNA 1000 chip. 500 ng of the purified library was then hybridized to biotinylated RNA library baits for 24 hrs and 65 ºC. These were then captured using streptavidin magnetic beads and further purified using AMPure XP beads. Unique index sequences for multiplex sequencing were added to captured DNA libraries and amplified for 10 cycles. Post PCR amplification, the libraries were purified by AMPure XP beads, quantified by Qubit and the quality was assessed on an Agilent 2100 Bioanalyzer using DNA high sensitivity chip. A final concentration of 8 pM was used for cluster generation and sequenced using 75 bp paired end recipe on GAIIx and 100 bp paired end recipe on HiSeq 2000.

*b. TruSeq*

The libraries for the samples were prepared as per “TruSeq DNA sample preparation guide & TruSeq Enrichment Guide”. 1 µg of high quality genomic DNA extracted from the samples was sheared to a peak size of 200 to 300 bp using the Covaris S2. The samples were end repaired, A-Tailed and adaptor ligated with unique adaptors for multiplex sequencing. Libraries between 300-400bp were selected, purified and PCR amplified. Libraries incubated with Ampure XP beads was quantified using Qubit and its quality was assessed on Agilent 2100 Bioanalyzer using DNA 1000 chip. For exome enrichment, multiple libraries (upto 6 samples) with different indices were combined to a single pool. 500 ng of each library was used for enrichment and the final volume was adjusted to 40 µl. In the hybridization phase, the DNA library was mixed with capture probes to target regions of interest. Capture target oligos and buffer were added as recommended and incubated at 58ºC for 18 hours. Streptavidin beads were used to capture probes hybridized to the targeted regions of interest. A series of wash procedures were performed as recommended to remove non-specific binding from the beads. The enriched library was then eluted from the beads and prepared for a second round of hybridization. Here, the eluted DNA library from the first enrichment round was combined with additional capture probes to targeted regions of interest and incubated at 58ºC for 18 hours. This was to ensure high specificity of the captured regions. Streptavidin beads were used to capture probes hybridized to the targeted regions of interest. A series of wash procedures were performed later to remove non-specific binding from the beads followed by a cleanup using sample purification beads. The samples were then PCR amplified again for 10 cycles as recommended. The libraries were purified by sample purification beads, quantified by Qubit and the quality was assessed on Agilent 2100 Bioanalyzer using DNA high sensitivity chip. A final concentration of 8 - 10 pM was used for cluster generation and sequenced using 75 bp paired end recipe on GAIIx and 100 bp paired end recipe on HiSeq 2000.

*c. Nextera*

The libraries for the samples were prepared as per “Nextera Rapid Capture Guide”. 50 ng of high quality genomic DNA extracted from the samples were simultaneously fragmented and adapter sequences added to their ends (tagmentation) by Nextera transposome. A cleanup was performed using sample purification beads provided to remove all Nextera transposome which can bind tightly to DNA ends and interfere with downstream processes if not removed . Unique index 1 (i7) and index 2 (i5) was added to each samples and PCR amplified for 10 cycles as recommended. The libraries were purified by sample purification beads, quantified by Qubit and the quality was assessed on a Agilent 2100 Bioanalyzer using DNA 1000 chip. For exome enrichment multiple libraries (upto 12 samples) with different indices were combined to a single pool. 500 ng of each library was used for enrichment and the final volume was adjusted to 40 µL. First hybridization was set up during which the the DNA library is mixed with capture probes to targeted regions of interest. Expanded exome oligos and buffer were added as recommended and incubated at 58C for 3 hours. Streptavidin beads were used to capture probes hybridized to the targeted regions of interest. Later two heated wash procedures were performed to remove non-specific binding from the beads. The enriched library was then eluted from the beads and prepared for a second round of hybridization. In the second hybridization, the eluted DNA library from the first enrichment round is combined with additional capture probes to targeted regions of interest and incubated at 58C for 18 hours. This is to ensure high specificity of the captured regions. Streptavidin beads were used to capture probes hybridized to the targeted regions of interest. Later two heated wash procedures were performed to remove non-specific binding from the beads followed by a cleanup using sample purification beads. The samples were then PCR amplified again for 10 cycles as recommended. The libraries were purified by sample purification beads, quantified by Qubit and the quality was assessed on a Agilent 2100 Bioanalyzer using DNA high sensitivity chip. A final concentration of 8 - 10 pM was used for cluster generation and sequenced using 100 bp paired end recipe on HiSeq 2000.

2. **Capillary sequencing**

*a. Primer designing*

Primers were designed using NCBI primer designing tool. Specificity of the designed primers were ensured by UCSC(University of California Santa Cruz) In Silico PCR. Primers were 18 to 20 mer in size and were get it synthesized from IDT(Integrated DNA Technologies).

*b. PCR amplification*

Region of interest having the SNP were amplified by using specific primers. 25ul of total reaction containing 5ng of template, 200uM dNTPs, 200nM forward and reverse primers, 10x Platinum High fidelity buffer, Platinum taq DNA polymerase High fidelity enzyme and the reaction volume was made up to 25ul with nuclease free water.

The amplifications were carried out in Gene Amp PCR System 9700 thermal cycler. Initial denaturation was done at 95^0^c for 3min followed by 10 cycles of touchdown PCR cycles with 1^0^c reduction in annealing temperature. The 10^th^ cycle is the the expected annealing temperature and with this temperature 25 more cycles of amplifications were performed. Extension temperature was 68^0^C for 3 min. After PCR, a portion of amplicons say 3 to 4ul were loaded in agarose gel to check the specificity of amplification.

*c. PCR purification*

Specific amplicons were purified by using Minelute PCR purification kit from Qiagen and the concentration was checked by Qubit (with double stranded HS reagent).

*d. Cycle sequencing PCR*

A range of 1-3ngs of DNA were taken for the cycle sequencing PCR for the reaction volume of 10ul. Cycle sequencing PCR was done by using Big Dye Terminator cycle sequencing (v1.1) kit (Applied biosystems) with specific primers. Reaction volume of 10ul contains 0.25ul of BDT reagent(1/16^th^ dilution), 3.2pmol of either forward or reverse primer and 1-3ng of DNA (template quantity depends on the size of the PCR fragment), 5x sequencing buffer and rest added water to make up the volume to 10ul. The amplifications were carried out using the Gene Amp PCR System 9700 thermal cycler.

*e. Ethanol precipitation*

PCR product should be cleaned before sequencing and which was done by EDTA/Sodium

acetate/ethanol precipitation. 12ul of mix I (milli Q + 125mM EDTA) and 52ul of mix II (3M Sodium acetate + Merck ethanol) added to each of the reaction well, mixed and incubated for 15 minutes at room temperature. After incubation plate was centrifuged at 3000rcf for 20 min at room temperature and the supernatant was discarded.

The pellet was washed with 70% ethanol, centrifuged (3000rcf for 10 min at room temperature) and the supernatant was discarded. The pellet was resuspended in HiDi formamide (12-14ul) and after denaturation (95^o^C for 3min), the plate was snap chilled, gently spun and loaded on to the 3500DX Genetic Analyzer (Applied Biosystems) for sequencing.
