# Supplementary material for: Integrated analysis of oral tongue squamous cell carcinoma identifies key variants and pathways linked to risk habits, HPV, clinical parameters and tumor recurrence
Source: F1000Res. 2015 Nov 5;4:1215. [Version 1] doi: 10.12688/f1000research.7302.1 (PMC4706066; doi:10.12688/f1000research.7302.1)
Supplement: Supplementary file 1 [file f1000research-4-7870-s0000.tgz › 4007fd05-e466-4e59-a4ef-512f9d41f650.zip!/Additional files/Additional file 16.doc]

**Scripts used in the study**

*1. Read QC*

qc_reads_extractor_22082012 -1 R1.fastq -2 R2.fastq

*2. Alignment and post-processing*

/Apps/mvapich2/bin/mpiexec.hydra -np $NSLOTS -hostfile $TMPDIR/machines /Apps/NOVO/novo_3.00.05/novoalignMPI/novoalignMPI -d hg19_GATK_Ref.nidx -f R1_QC_passed.fastq R2_QC_passed.fastq -a -r All -o SAM '@RG\tID:default\tPU:illumina\tSM:samplename' > samplename_NovoV3.00.05.sam

if [ $# -lt 1 ] || [ ! -e "$1.sam" ]

then

echo -e "Usage:\n\tbash $0 <sam file prefix>"

exit 1

fi

mkdir flagstats

samtools view -bS $1.sam > $1.bam

samtools sort $1.bam $1.coordSorted

samtools index $1.coordSorted.bam

samtools flagstat $1.coordSorted.bam > ./flagstats/$1.flagstat.txt

java -jar /Apps/picard-tools-1.39/AddOrReplaceReadGroups.jar INPUT=$1.coordSorted.bam OUTPUT=$1.coordSorted.RG.bam RGID=default RGLB=PE RGPL=illumina RGPU=illumina RGSM=`echo $1 | cut -b 1-5`

samtools index $1.coordSorted.RG.bam

samtools view -h samplename.coordSorted.RG.bam | grep -v NH | samtools view -bS - > samplename.coordSorted.RG.uniqMapped.bam

#!/bin/bash

#$ -N gatk_CL

#$ -cwd

#$ -V

#$ -o $JOB_ID.$JOB_NAME.OUT

#$ -e $JOB_ID.$JOB_NAME.ERR

#$ -pe mvapich2 4

bam=$v1

outfile=$v2

java -Xmx10g -jar /Apps/GenomeAnalysisTK-2.5-2-gf57256b/GenomeAnalysisTK.jar -T CallableLoci -I $bam -R /common/Data/Internal/Others/Reference_genome/hg_19/hg19_GATK_Ref.fa -log $outfile.log -summary $outfile.summary -o $outfile.callable.bed -format BED

*3. Variant discovery and post-processing*

*a. SNPs & indels from GATK*

#!/bin/bash

echo -n "$1::" >> gatk_log

date >> gatk_log

################################################

#Required Files :

#1. hg19_GATK_Ref.fa

#2. hg19_GATK_Ref.dict

#3. hg19_GATK_Ref.fa.fai

#4. dbsnp_137.hg19.vcf

#5. 1000G_phase1.indels.hg19.vcf

if [ $# -lt 4 ]

then

echo -e "Usage:\n\tbash $0 <input bam> <prefix> <RG_tag> <output_dir>"

exit 1

fi

###################################################

input=$1

prefix=$2

RG=$3

out_dir=$4

mkdir $out_dir

echo "#!/bin/bash" > gatk.$prefix.sh

echo "#$ -N gatk" >> gatk.$prefix.sh

echo "#$ -cwd" >> gatk.$prefix.sh

echo "#$ -V" >> gatk.$prefix.sh

echo "#$ -o $out_dir/\$JOB_ID.\$JOB_NAME.OUT" >> gatk.$prefix.sh

echo "#$ -e $out_dir/\$JOB_ID.\$JOB_NAME.ERR" >> gatk.$prefix.sh

echo "#$ -pe smp 6" >> gatk.$prefix.sh

echo "#$ -j y" >> gatk.$prefix.sh

echo "#$ -m aes" >> gatk.$prefix.sh

echo "echo \"$1\" >> $out_dir/gatk_prefix_log; date >> $out_dir/gatk_prefix_log" >> gatk.$prefix.sh

echo >> gatk.$prefix.sh

echo "java -Xmx1g -jar /Apps/GATK/GenomeAnalysisTK-1.2-62/GenomeAnalysisTK.jar -I $input -T RealignerTargetCreator -R hg19_GATK_Ref.fa -o $out_dir/$prefix.IndelRealigner.intervals --known 1000G_phase1.indels.hg19.vcf" >> gatk.$prefix.sh

echo >> gatk.$prefix.sh

echo "java -Xmx4g -jar /Apps/GATK/GenomeAnalysisTK-1.2-62/GenomeAnalysisTK.jar -I $input -R hg19_GATK_Ref.fa -T IndelRealigner --targetIntervals $out_dir/$prefix.IndelRealigner.intervals -o $out_dir/$prefix.realigned.bam" >> gatk.$prefix.sh

echo >> gatk.$prefix.sh

echo "java -jar /Apps/picard-tools-1.39/MarkDuplicates.jar INPUT=$out_dir/$prefix.realigned.bam OUTPUT=$out_dir/$prefix.realigned.DupRem.bam REMOVE_DUPLICATES=true ASSUME_SORTED=true CREATE_INDEX=true READ_NAME_REGEX='[-a-zA-Z0-9]+:[0-9]:([0-9]+):([0-9]+):([0-9]+).*' METRICS_FILE=$out_dir/$prefix.picardMetrics.txt" >> gatk.$prefix.sh

echo >> gatk.$prefix.sh

echo "samtools index $out_dir/$prefix.realigned.DupRem.bam" >> gatk.$prefix.sh

echo >> gatk.$prefix.sh

echo "java -jar /Apps/GATK/GenomeAnalysisTK-1.2-62/GenomeAnalysisTK.jar -R hg19_GATK_Ref.fa -knownSites:VCF dbsnp_137.hg19.vcf -I $out_dir/$prefix.realigned.DupRem.bam -T CountCovariates -nt 6 -cov QualityScoreCovariate -cov CycleCovariate -recalFile $out_dir/$prefix.realigned.DupRem_firstRecal.csv --default_read_group $RG --default_platform illumina" >> gatk.$prefix.sh

echo >> gatk.$prefix.sh

echo "java -jar /Apps/GATK/GenomeAnalysisTK-1.2-62/GenomeAnalysisTK.jar -R hg19_GATK_Ref.fa -I $out_dir/$prefix.realigned.DupRem.bam -T TableRecalibration -o $out_dir/$prefix.realigned.DupRem.firstRecal.bam -recalFile $out_dir/$prefix.realigned.DupRem_firstRecal.csv --default_read_group $RG --default_platform illumina" >> gatk.$prefix.sh

echo >> gatk.$prefix.sh

echo "java -jar /Apps/GATK/GenomeAnalysisTK-1.2-62/GenomeAnalysisTK.jar -R hg19_GATK_Ref.fa -knownSites:VCF dbsnp_137.hg19.vcf -I $out_dir/$prefix.realigned.DupRem.firstRecal.bam -T CountCovariates -nt 6 -cov QualityScoreCovariate -cov CycleCovariate -recalFile $out_dir/$prefix.realigned.DupRem_secondRecal.csv --default_read_group $RG --default_platform illumina" >> gatk.$prefix.sh

echo >> gatk.$prefix.sh

echo "java -Xmx4g -jar /Apps/GATK/GenomeAnalysisTK-1.2-62/AnalyzeCovariates.jar -recalFile $out_dir/$prefix.realigned.DupRem_secondRecal.csv -outputDir $out_dir/$prefix.AnalysisCovariateOutput -ignoreQ 5 -resources /Apps/GATK/GenomeAnalysisTK-1.2-62/resources/" >> gatk.$prefix.sh

echo >> gatk.$prefix.sh

echo "samtools index $out_dir/$prefix.realigned.DupRem.firstRecal.bam" >> gatk.$prefix.sh

echo >> gatk.$prefix.sh

echo "java -Xmx10g -jar ~/apps/GenomeAnalysisTK-2.5-2-gf57256b/GenomeAnalysisTK.jar -R hg19_GATK_Ref.fa -T UnifiedGenotyper -I $out_dir/$prefix.realigned.DupRem.firstRecal.bam --dbsnp /common/Data/Internal/Others/GATK_References_Package/dbsnp_138.hg19.11012014.withChr.vcf -o $out_dir/$prefix.raw.vcf -stand_call_conf 50.0 -stand_emit_conf 10.0 -glm BOTH" >> gatk.$prefix.sh

echo "echo \"$1\" >> $out_dir/gatk_prefix_log; date >> $out_dir/gatk_prefix_log" >> gatk.$prefix.sh

echo -n "$1::" >> gatk_log

date >> gatk_log

echo -e "Run: qsub gatk.$prefix.sh"

*b. Post-processing SNPs from GATK*

#Exomefilter PASS SNPs

##############################################

intersectBed -a sample_UGv2.5.2.raw.vcf -b /common/Data/projects/internal/OSCC/33MB_hg19.bed -wa | sort -u | grep -v LowQual | grep " [AGTC] [AGTC] \|

[AGTC] [AGTC],[AGTC] " > exomeFilteredSNPs/33MB/sample_Ugv2.5.2.raw.onlySNPs.PASS.33MB.vcf

intersectBed -a sample_UGv2.5.2.raw.vcf -b /common/Data/projects/internal/OSCC/SS50MBunique_hg19_woRandom.bed -wa | sort -u | grep -v LowQual | grep " [AGTC] [AGTC] \|

[AGTC] [AGTC],[AGTC] " > exomeFilteredSNPs/33MB/sample_Ugv2.5.2.raw.onlySNPs.PASS.50MB.vcf

intersectBed -a sample_UGv2.5.2.raw.vcf -b /common/Data/projects/internal/OSCC/TS62MBunique_hg19.bed -wa | sort -u | grep -v LowQual | grep "	[AGTC] [AGTC] \|	[AGTC] [AGTC],[AGTC]	" > exomeFilteredSNPs/TS62MBunique/sample_Ugv2.5.2.raw.onlySNPs.PASS.62MB.vcf

#Merge across exome baits

##############################################

cat exomeFilteredSNPs/*/sample_Ugv2.5.2.raw.onlySNPs.PASS.*.vcf > exomeFilteredSNPs/mergedVCFs/sample_Ugv2.5.2.raw.onlySNPs.PASS.33MB_50MB_62MB.vcf

##Annotate dbSNP and COSMIC:

##############################################

#!/bin/bash

if [ $# -lt 1 ]

then

echo -en "Usage:\n\tbash $0 <input.vcf>\n";

exit 0;

fi

vcf=$1

id=`echo $vcf | sed 's/.vcf//'`

awk -v OFS="\t" 'FNR==NR{a[$1"#"$2"#"$4"#"$5]=$3; next} {if(a[$1"#"$2"#"$4"#"$5]) {$3=a[$1"#"$2"#"$4"#"$5]; print} else {$3="."; print} }' /common/Data/projects/internal/OSCC/dbSNP138_hg19_onlySNPs.vcf $vcf | grep -v "^#"| awk -v OFS="\t" 'FNR==NR{a[$1"#"$2"#"$4"#"$5]=$3; next} {if(a[$1"#"$2"#"$4"#"$5]) {$8=$8";COSMIC="a[$1"#"$2"#"$4"#"$5]} print}' /common/Data/projects/internal/OSCC/CosmicCodingMuts_v67_20131024.onlySNPs.vcf - > $id.withDBSNP.COSMIC.vcf

#Callability

##############################################

intersectBed -a exomeFilteredSNPs/mergedVCFs/sample_Ugv2.5.2.raw.onlySNPs.PASS.33MB_50MB_62MB.vcf -b OSCC/GATK/CallableLoci/realignedDupRem/SAMPLE_T.callable.formatted.bed -loj | grep CALLABLE | cut -f1-10 | /Apps/BEDTools/bin/intersectBed -a - -b OSCC/GATK/CallableLoci/realignedDupRem/SAMPLE_N.callable.formatted.bed -loj | grep CALLABLE | cut -f1-10 > exomeFilteredSNPs/mergedVCFs/callable/SAMPLE.vcf

#Depth of coverage

##############################################

#!/bin/bash

#$ -cwd

#$ -N DepthOfCov

#$ -V

#$ -o $JOB_ID.$JOB_NAME.out

#$ -e $JOB_ID.$JOB_NAME.err

#$ -pe mvapich2 7

#$ -j y

#$ -m aes

echo -en "Usage:\n\tqsub -v bam=<bam>,target=<target_bed>,prefix=<out prefix> $0\n";

outID=$prefix

echo $PWD

echo $bam

echo $target

echo $prefix

echo $outID

java -Xmx10g -jar /Apps/GenomeAnalysisTK-2.2-15-g9214b2f/GenomeAnalysisTK.jar -T DepthOfCoverage -I $bam -L $target -R hg19_GATK_Ref.fa --printBaseCounts -o $outID

##############################################

#Matched Normal Variant Depth filter:

nohup gawk 'ARGIND==1 {cpos[$1] = $5 $6 $7 $8; next} $1 in cpos {printf("%s\t%s\n", $0, cpos[$1])}' <(gunzip -c OSCC/bams/uniqMapped/allBamsForAnalysis/pileup/SAMPLE_N_depth.gz) <(cut -f1,2 --output-delimiter=":" SAMPLE.vcf) | sed 's/[ACTGN]:/ /g' | paste -d"\t" - <(cut -f4-5 SAMPLE.vcf) > SAMPLE.depth

sed 's/t\([ACTG]\)/ \1/' SAMPLE.depth | sed 's/\t/ /g' | awk '{if (($7 == "A" && $2 == 0) || ($7 == "C" && $3 == 0) || ($7 == "G" && $4 == 0) || ($7 == "T" && $5 == 0)) print $1}' | grep "$(sed 's/:/\t/g')" SAMPLE.vcf > SAMPLE.callable.altFiltered.SNPs

*c. Indels using DINDEL*

#The $prefix.realigned.DupRem.firstRecal.bam from GATK was used to call indels using DINDEL.

##############################################

/Apps/Dindel/binaries/dindel-1.01-linux-64bit --ref hg19_GATK_Ref.fa --outputFile sample_recal --bamFile sample.realigned.DupRem.firstRecal.bam --analysis getCIGARindels

/Apps/Python2.6/bin/python /Apps/Dindel/dindel-1.01-python/makeWindows.py --inputVarFile sample_recal.variants.txt --windowFilePrefix sample_recal.realign_windows --numWindowsPerFile 1000

ls -1 sample_recal.realign_windows.* | parallel "/Apps/Dindel/binaries/dindel-1.01-linux-64bit --analysis indels --doDiploid --bamFile sample.realigned.DupRem.firstRecal.bam --ref hg19_GATK_Ref.fa --varFile {} --libFile sample_recal.libraries.txt --outputFile {}.out"

ls -1 *.glf.txt > sample_recal_stage3_outputfiles.txt

/Apps/Python2.6/bin/python ~/Oral_cancer/mergeOutputDiploid.py --inputFiles sample_recal_stage3_outputfiles.txt --outputFile sample_recal.variantCalls.VCF --ref hg19_GATK_Ref.fa

*d. Post-processing and merging Indels from GATK and DINDEL*

##############################################

#Files:

#/common/Data/projects/internal/OSCC/GATK/calls_UGv2.5.2/sample_UGv2.5.2.raw.vcf

#/common/Data/projects/internal/OSCC/TS62MBunique_hg19.bed

#/common/Data/projects/internal/OSCC/SS50MBunique_hg19_woRandom.bed

#/common/Data/projects/internal/OSCC/33MB_hg19.bed

#/common/Data/projects/internal/OSCC/postToolAnalysis/INDELs_GATK_dindel/hg19_Microsat.SimpRep.UCSC.26032014_TRFdb.28032014_withFlanks_woUnplaced.bed

#/common/Data/Internal/Others/GATK_References_Package/dbsnp_138.hg19.11012014.withChr.vcf

#/common/Data/Internal/Others/GATK_References_Package/CosmicCodingMuts_v67_20131024.sorted.vcf

#/common/Data/projects/internal/OSCC/merged_SS38MB_SS50MB_TS62MB_hg19.bed

#/common/Data/Internal/Others/Reference_genome/hg_19/hg19_GATK_Ref.fa

#/common/Data/projects/internal/OSCC/GATK/CallableLoci/realignedDupRem/Callable.Loci.only.1.april.2014/*[BN].callable.formatted.bed.callable.loci.only

#/common/Data/Internal/Others/GATK_References_Package/CosmicCodingMuts_v67_20131024.sorted.vcf.gz

#Utils:

#/Apps/vcftools_0.1.11/bin/vcf-sort

#/Apps/GenomeAnalysisTK-2.5-2-gf57256b/GenomeAnalysisTK.jar

#/Apps/variant_effect_predictor/variant_effect_predictor.pl

###########################################

#GATK vcfs:

##############################################

#get only Indels, ExomeFilter, PASS filter:

##############################################

#grep to be checked here - LowQual for GATK 2.5

intersectBed -a sample_UGv2.5.2.raw.vcf -b TS62MBunique_hg19.bed -wa | sort -u | grep -v LowQual | grep -v "	[AGTC]	[AGTC]	\|	[AGTC]	[AGTC],[AGTC]	" > sample_UGv2.5.2.raw.onlyINDELs.PASS.62MBu.vcf

intersectBed -a sample_UGv2.5.2.raw.vcf -b SS50MBunique_hg19_woRandom.bed -wa | sort -u | grep -v LowQual | grep -v "	[AGTC]	[AGTC]	\|	[AGTC]	[AGTC],[AGTC]	" > sample_UGv2.5.2.raw.onlyINDELs.PASS.50MBu.vcf

intersectBed -a sample_UGv2.5.2.raw.vcf -b 33MB_hg19.bed -wa | sort -u | grep -v LowQual | grep -v "	[AGTC]	[AGTC]	\|	[AGTC]	[AGTC],[AGTC]	" > sample_UGv2.5.2.raw.onlyINDELs.PASS.33MB.vcf

##############################################

#Merge 3 partitions sample-wise vcfs:

cat sample_UGv2.5.2.raw.onlyINDELs.PASS.33MB.vcf sample_UGv2.5.2.raw.onlyINDELs.PASS.50MBu.vcf sample_UGv2.5.2.raw.onlyINDELs.PASS.62MBu.vcf | sort -u > sample_UGv2.5.2.PASS.onlyINDELs.33MB.SS50MBu.TS62MBu.vcf

##############################################

#Annotate dbSNP and COSMIC:

perl5.10.1 variant_effect_predictor.pl -i sample_UGv2.5.2.PASS.onlyINDELs.33MB.SS50MBu.TS62MBu.vcf -o sample_UGv2.5.2.PASS.onlyINDELs.33MB.SS50MBu.TS62MBu.withCOSMIC.vcf --force_overwrite --custom CosmicCodingMuts_v67_20131024.sorted.vcf.gz,COSMIC,vcf,exact,0 --vcf --offline --no_stats --cache

sed -i 's/;CSQ[^;	][^;	]*\([	;]\)/\1/' sample_UGv2.5.2.PASS.onlyINDELs.33MB.SS50MBu.TS62MBu.withCOSMIC.vcf

##############################################

#Repeat filter:

grep -v ^# sample_UGv2.5.2.PASS.onlyINDELs.33MB.SS50MBu.TS62MBu.withCOSMIC.vcf | awk 'OFS="\t" {$8=$8";GATK"; print $0}' | grep -v STR | intersectBed -v -f 0.85 -a - -b hg19_Microsat.SimpRep.UCSC.26032014_TRFdb.28032014_withFlanks_woUnplaced.bed | vcf-sort -c > sample_UGv2.5.2.PASS.onlyINDELs.33MB.SS50MBu.TS62MBu.withCOSMIC.RepFilt.vcf

##############################################

#Format header:

(grep "^#" sample_UGv2.5.2.raw.vcf; grep -v "^#" sample_UGv2.5.2.PASS.onlyINDELs.33MB.SS50MBu.TS62MBu.withCOSMIC.RepFilt.vcf) > sample_UGv2.5.2.PASS.onlyINDELs.33MB.SS50MBu.TS62MBu.withCOSMIC.RepFilt.withHeader.vcf

sed -i '10i##INFO=<ID=COSMIC,Number=.,Type=String,Description="/common/Data/Internal/Others/GATK_References_Package/CosmicCodingMuts_v67_20131024.sorted.vcf.gz (exact)">' sample_UGv2.5.2.PASS.onlyINDELs.33MB.SS50MBu.TS62MBu.withCOSMIC.RepFilt.withHeader.vcf

sed -i '11i##INFO=<ID=GATK,Number=.,Type=Flag,Description="Source: GATK">' sample_UGv2.5.2.PASS.onlyINDELs.33MB.SS50MBu.TS62MBu.withCOSMIC.RepFilt.withHeader.vcf

#############################################

#Dindel vcfs:

##############################################

#Annotate dbSNP and COSMIC:

awk -v OFS="\t" 'FNR==NR {$4=toupper($4); $5=toupper($5); if($1~/^#/) {next}; split($5,dbalts,","); for (j in dbalts) {if(length($4)==length(dbalts[j]) && length($4)==2) {split($4,ref1,""); split(dbalts[j],alt1,""); for (k in ref1) {pos=$2+k-1; a[$1"#"pos"#"ref1[k]"#"alt1[k]]=$3}} else {a[$1"#"$2"#"$4"#"dbalts[j]]=$3;}} next} {$4=toupper($4); $5=toupper($5); split($5, alts, ","); for(i in alts) {if(a[$1"#"$2"#"$4"#"alts[i]]) {$3=a[$1"#"$2"#"$4"#"alts[i]] }} print}' dbsnp_138.hg19.11012014.withChr.vcf sample_dindel.vcf | awk -v OFS="\t" 'FNR==NR {a[$1"#"$2"#"$4"#"$5]=$3; next} {split($5,alts,","); for(i in alts) {if(a[$1"#"$2"#"$4"#"alts[i]]) $8=$8";COSMIC="a[$1"#"$2"#"$4"#"alts[i]] } if(cosmic) $8=$8";COSMIC="cosmic; print}' CosmicCodingMuts_v67_20131024.sorted.vcf - > sample_dindel.withDBSNP.COSMIC.vcf

##############################################

#ExomeFilter, PASS filter, Repeat filter:

(grep ^# sample_dindel.withDBSNP.COSMIC.vcf; grep -v ^# sample_dindel.withDBSNP.COSMIC.vcf | awk 'OFS="\t" {$8=$8";Dindel"; print $0}' | intersectBed -a - -b merged_SS38MB_SS50MB_TS62MB_hg19.bed | grep PASS | intersectBed -v -f 0.85 -a - -b hg19_Microsat.SimpRep.UCSC.26032014_TRFdb.28032014_withFlanks_woUnplaced.bed | vcf-sort -c) > sample_dindel.withDBSNP.COSMIC.38_50_62.PASS.RepFilt.vcf

##############################################

#Add header:

sed -i '5i##INFO=<ID=Dindel,Number=0,Type=Flag,Description="Source: Dindel">' sample_dindel.withDBSNP.COSMIC.38_50_62.PASS.RepFilt.vcf

##############################################

#Format VCF:

sed -i 's/;COSMIC=ID//' sample_dindel.withDBSNP.COSMIC.38_50_62.PASS.RepFilt.vcf

##############################################

#Delete ambiguous variants:

awk '{if (($5!~/,/) && ($10~/1\/2/)) next; else print}' sample_dindel.withDBSNP.COSMIC.38_50_62.PASS.RepFilt.vcf > t; mv t sample_dindel.withDBSNP.COSMIC.38_50_62.PASS.RepFilt.vcf

##############################################

#CombineVariants:

/Apps/smrtanalysis/redist/jdk1.7.0_02/bin/java -jar GenomeAnalysisTK.jar -T CombineVariants -V sample_dindel.withDBSNP.COSMIC.38_50_62.PASS.RepFilt.vcf -V sample_UGv2.5.2.PASS.onlyINDELs.33MB.SS50MBu.TS62MBu.withCOSMIC.RepFilt.withHeader.vcf -R hg19_GATK_Ref.fa -o sample.dindel_GATK.33_50_62.PASS.dbSNP_COSMIC.repFilt.vcf -log logs/sample_combine.log

##############################################

#T-specific indels:

intersectBed -v -a *T.dindel_GATK.33_50_62.PASS.dbSNP_COSMIC.repFilt.vcf -b *[BN].dindel_GATK.33_50_62.PASS.dbSNP_COSMIC.repFilt.vcf > *.T-specific.vcf

##############################################

#Callable T-specific indels:

intersectBed -a *.T-specific.vcf -b *[BN].callable.formatted.bed.callable.loci.only -wo | awk -v OFS="\t" '{sum[$1 OFS $2 OFS $3 OFS $4 OFS $5 OFS $6 OFS $7 OFS $8 OFS $9 OFS $10 OFS $11]+=$16} END {for (a in sum) print a,sum[a]}' | awk 'OFS="\t" {if ($12/length($4) >= 0.6) print}' | cut -f 1-11 > *.T-specific.Callable.vcf

##############################################

#Matched Normal Variant Depth filter:

grep "$(cut -f1,2 --output-delimiter=" " SAMPLE.T-specific.Callable.vcf)" OSCC/dindel/PostPicardBAM/SAMPLE_N/SAMPLE_N_recal.variants.txt > SAMPLE.1;grep "$(cut -d" " -f1,2 --output-delimiter=" " SAMPLE.1)" SAMPLE.T-specific.Callable.vcf > SAMPLE.2;sort -k1,1V -k2,2g -o SAMPLE.1 SAMPLE.1;sort -k1,1V -k2,2g -o SAMPLE.2 SAMPLE.2;sort -k1,1V -k2,2g -o SAMPLE.T-specific.Callable.vcf SAMPLE.T-specific.Callable.vcf;/Apps/BEDTools/bin/subtractBed -a SAMPLE.T-specific.Callable.vcf -b SAMPLE.2 > SAMPLE.T-specific.Callable.altFiltered.vcf

##############################################

*4. Estimating cross-sample contamination using ContEst*

a. CONTEST.sh:

if [ $# -lt 3 ]

then

echo -e "Usage:\n\tbash $0 <bam_file> <snp_vcf_file> <target_bed_file>"

exit 0

fi

bam=$1

vcf=$2

target_bed=$3

if [ ! -e $bam -o ! -e $vcf -o ! -e $target_bed ]

then

echo "Error: One of the input files is incorrect, please check the input files!!!"

exit 0

fi

echo -e "Bam:\t$bam"

echo -e "VCF:\t$vcf"

echo -e "Target:\t$target_bed"

java -Xmx2400m -jar /Apps/contest/contest-1.0.24530-bin/ContEst.jar \

-T Contamination \

-I $bam \

-R /common/Data/Internal/Others/Reference_genome/hg_19/hg19_GATK_Ref.fa \

-L $target_bed_file \

-B:pop,vcf /home/venkatesh/contest/pooledNormal450.vcf \

-B:genotypes,vcf $vcf \

-BTI genotypes \

-o $vcf.contest.out

b.

#NAME = step1

#DESCRIPTION = This file creates the pooled normal file of all the gatk PASS exome-filtered(cat of 33,50,62MB)

#INPUT = path to the file containing normal samples names and path to the vcf files

#OUTPUT = output.step1

$inL=$ARGV[0];

if($#ARGV==1){

$string="";

$loc=$ARGV[1];

@list=`cat $inL`;

for ($i=0; $i<=$#list; ++$i){

chomp ($list[$i]);

$string=$string." ".$loc.$list[$i];

}

print "$string\n";

`cat $string > output.step1`;

}else{

print "\n\n\tUSAGE # perl step1.pl <path-2-file> <path-2-vcf>\n\n\tNAME = step1\n\tDESCRIPTION = This file creates the pooled normal file of all the raw gatks\n\tINPUT = path to the file containing normal samples names and path to the vcf files\n\tOUTPUT = output.step1\n\n\n";

}

c.

#NAME = step2.run

#DESCRIPTION = This file sorts the output.step1 file using /Apps/coreutils-8.13/src/sort

#INPUT = nothing it takes the output of step1.pl script

#OUTPUT = output.step2

#$inL=$ARGV[0];

if ($#ARGV<0){

`/Apps/coreutils-8.13/src/sort -k1,1V -k2n /home/venkatesh/contest/development/output.step1 -o /home/venkatesh/contest/development/output.step2.tmp`;

`grep PASS output.step2.tmp > output.step2`;

\#	`awk \'{if(length($4)==1 && length($5)==1) print $0}\' output.step2.pass> output.step2`;

`rm output.step2.tmp`;

}else{

print "\n\n\tUSAGE # perl step2.pl\n\n\tNAME = step2.run\n\tDESCRIPTION = This file sorts the output.step1 file using /Apps/coreutils-8.13/src/sort\n\tINPUT = nothing it takes the output of step1.pl script\n\tOUTPUT = output.step2\n\n\n";

}

d.

=head

NAME = step4.pl

DESCRIPTION = This script calculates the AC,AF and allele frequency

INPUT = output.step3

INPUT FORMAT

chr1	14464	.	A	T	1434.92	PASS	AC=1;AF=0.50;AN=2;BaseQRankSum=-0.273;DP=200;Dels=0.00;FS=38.566;HRun=0;HaplotypeScore=3.4411;MQ=45.62;MQ0=0;MQRankSum=3.451;QD=7.17;ReadPosRankSum=1.737	GT:AD:DP:GQ:PL	0/1:143,57:200:99:1465,0,4396

chr1	14464	.	A	T	438.28	PASS	AC=1;AF=0.50;AN=2;BaseQRankSum=-1.441;DP=112;Dels=0.00;FS=0.869;HRun=0;HaplotypeScore=0.0000;MQ=37.74;MQ0=0;MQRankSum=0.490;QD=3.91;ReadPosRankSum=1.736	GT:AD:DP:GQ:PL	0/1:89,23:112:99:468,0,2537

OUTPUT = output.step4

OUTPUT FORMAT

chr1	14464	.	A	T	1783.79	PASS	5	0.50000	0.13158

chr1	14522	.	G	A	197.64	PASS	13	0.50000	0.34211

=cut

#!/usr/bin/perl -w

open (INFILE, "output.step2");

open (OUTFILE, ">output.step3");

$line=0;$sum=1;$AC=0;$AF=0;$QUAL=0;

do{

$line++;

my $linedata = <INFILE>;

my @data=split '\t', $linedata;

my @sub=split ';', $data[7];

my @ac=split '=', $sub[0];

my @af=split '=', $sub[1];

$AC=$ac[1];

$AF=$af[1];

$QUAL=$data[5];

if($line>1){

if(($data[0] eq $temp[0] && $data[1] == $temp[1]) && ($data[3] eq $temp[3] && $data[4] eq $temp[4]) && $data[2] eq $temp[2]){

$sum=$temp[5]+1;

$AC=$temp[6] + $AC;

$AF=$temp[7] + $AF;

$QUAL=$temp[8] + $QUAL;

}else{

$sum=1;

}

if($sum<=$temp[5]){

$freqP=sprintf("%0.5f",$temp[5]/38);

$afP=sprintf("%0.5f",$temp[7]/$temp[5]);

$qualP=sprintf("%0.2f",$temp[8]/$temp[5]);

print OUTFILE $temp[0],"\t",$temp[1],"\t",$temp[2],"\t",$temp[3],"\t",$temp[4],"\t",$qualP,"\tPASS\t",$temp[6],"\t",$afP,"\t",$freqP,"\n";

}

}

@temp=($data[0],$data[1],$data[2],$data[3],$data[4],$sum,$AC,$AF,$QUAL);

}until eof;

$freqP=sprintf("%0.5f",$temp[5]/38);

$afP=sprintf("%0.5f",$temp[7]/$temp[5]);

$qualP=sprintf("%0.2f",$temp[8]/$temp[5]);

print OUTFILE $temp[0],"\t",$temp[1],"\t",$temp[2],"\t",$temp[3],"\t",$temp[4],"\t",$qualP,"\tPASS\t",$temp[6],"\t",$afP,"\t",$freqP,"\n";

close(OUTFILE);

close(INFILE);

e.

=head

NAME = step5.pl

DESCRIPTION = This script removes those lines, where there are more than one instance of CHR and POS field

INPUT output.step4

INPUT FORMAT

chr1	14464	.	A	T	1783.79	PASS	5	0.50000	0.13158

chr1	14522	.	G	A	197.64	PASS	13	0.50000	0.34211

OUTPUT output.step5

OUTPUT FORMAT

chr1	14464	.	A	T	xxx	1783.79	PASS	5	xxx	0.50000	xxx	0.13158	xxx

chr1	14522	.	G	A	xxx	197.64	PASS	13	xxx	0.50000	xxx	0.34211	xxx

=cut

#!/usr/bin/perl -w

open(INFILE, "output.step3");

open(OUT, ">output.step4.tmp");

$line=0;

do{

$line++;

my $lineData = <INFILE>;

@split = split '\t', $lineData;

$skip=0;

if ($line>1){

if(($split[1] eq $temp[1]) && ($split[0] eq $temp[0])){

print OUT $split[0],"\t",$split[1],"\t",$split[2],"\t",$split[3],"\t",$temp[4],"\t",$split[4],"\t",($split[5]+$temp[5]/2),"\t",$split[6],"\t",$temp[7],"\t",$split[7],"\t",$temp[8],"\t",$split[8],"\t",substr($temp[9],0,-1),"\t",substr($split[9],0,-1),"\n";

$skip=1;

}

if(($split[1] ne $temp[1]) && $mem == 0 ){

print OUT $temp[0],"\t",$temp[1],"\t",$temp[2],"\t",$temp[3],"\t",$temp[4],"\txxx\t",$temp[5],"\t",$temp[6],"\t",$temp[7],"\txxx\t",$temp[8],"\txxx\t",substr($temp[9],0,-1),"\txxx\n";

$skip=0;

}

}

@temp = @split;	$mem = $skip;

}until eof;

close (OUT);

close(INFILE);

@p=`cut -f2 output.step4.tmp|uniq -d`;

$s="";

for ($x=0;$x<=$#p;++$x){

$s=$s.substr($p[$x],0,-1);

if($x!=$#p){

$s=$s."\\|";

}

}

print "$s\n";

`grep -vw \'$s\' output.step4.tmp > output.step4`;

`rm output.step4.tmp`;

f.

=head

NAME = step5.pl

DESCRIPTION = This perl script creates a custom population file for the pooled normals

by substituting the calculated values from the previous step

INPUT output.step4

OUTPUT output.step5

=cut

#!/usr/bin/perl -w

$file=$ARGV[0];

$lines=`wc -l output.step4\|cut -d " " -f1`;

chomp $lines;

`tail -$lines $file > population.tmp`;

open (OUTFILE, ">output.step5");

open (INFILE1, "output.step4");

open (INFILE2, "population.tmp");

$line = 0;

do{

$line++;

$linedata1 = <INFILE1>;

@data=split '\t', $linedata1;

$chr=$data[0];

$pos=$data[1];

$id=$data[2];

$ref=$data[3];

$alt1=$data[4];

$alt2=$data[5];

$qual=$data[6];

$pass=$data[7];

$ac1=$data[8];

$ac2=$data[9];

$af1=$data[10];

$af2=$data[11];

$freq1=$data[12];

$freq2=$data[13];

$linedata2 = <INFILE2>;

@popu=split '\t', $linedata2;

@pop=split ';', $popu[7];

for($i=0;$i<=$#pop;++$i){

@sub=split '=', $pop[$i];

push (@full, @sub);

}

if($alt2 eq "xxx"){

print OUTFILE $chr,"\t",$pos,"\t",$id,"\t",$ref,"\t",$alt1,"\t",$qual,"\t",$pass,"\tAC=",$ac1,";AF=",$af1,";ALL={",$ref,"*=",substr($full[6],0,-1),$alt1,"=",$full[7],";AN=",$full[9],";ASW={",$ref,"*=",substr($full[12],0,-1),$alt1,"=",$full[13],";CEU={",$ref,"*=",(1-$freq1),", ",$alt1,"=",$freq1,"};CHB={",$ref,"*=",substr($full[20],0,-1),$alt1,"=",$full[21],";CHD={",$ref,"*=",substr($full[24],0,-1),$alt1,"=",$full[25],";CHS={",$ref,"*=",substr($full[28],0,-1),$alt1,"=",$full[29],";CLM={",$ref,"*=",substr($full[32],0,-1),$alt1,"=",$full[33],";FIN={",$ref,"*=",substr($full[36],0,-1),$alt1,"=",$full[37],";GBR={",$ref,"*=",substr($full[40],0,-1),$alt1,"=",$full[41],";GIH={",$ref,"*=",substr($full[44],0,-1),$alt1,"=",$full[45],";IBS={",$ref,"*=",substr($full[48],0,-1),$alt1,"=",$full[49],";JPT={",$ref,"*=",substr($full[52],0,-1),$alt1,"=",$full[53],";LWK={",$ref,"*=",substr($full[56],0,-1),$alt1,"=",$full[57],";MKK={",$ref,"*=",substr($full[60],0,-1),$alt1,"=",$full[61],";MXL={",$ref,"*=",substr($full[64],0,-1),$alt1,"=",$full[65],";PUR={",$ref,"*=",substr($full[68],0,-1),$alt1,"=",$full[69],";TSI={",$ref,"*=",substr($full[72],0,-1),$alt1,"=",$full[73],";YRI={",$ref,"*=",substr($full[76],0,-1),$alt1,"=",$full[77],";set=",$full[79],"\tGT\n";

}else{

print OUTFILE $chr,"\t",$pos,"\t",$id,"\t",$ref,"\t",$alt1,",",$alt2,"\t",$qual,"\t",$pass,"\tAC=",$ac1,",",$ac2,";AF=",$af1,",",$af2,";ALL={",$ref,"*=",substr($full[6],0,-1),$alt1,"=",substr($full[7],0,-1),", ",$alt2,"=",$full[7],";AN=",$full[9],";ASW={",$ref,"*=",substr($full[12],0,-1),$alt1,"=",substr($full[13],0,-1),", ",$alt2,"=",$full[13],";CEU={",$ref,"*=",(1-($freq1+$freq2)),", ",$alt1,"=",$freq1,", ",$alt2,"=",substr($freq2,0,-1),"};CHB={",$ref,"*=",substr($full[20],0,-1),$alt1,"=",substr($full[21],0,-1),", ",$alt2,"=",$full[21],";CHD={",$ref,"*=",substr($full[24],0,-1),$alt1,"=",substr($full[25],0,-1),", ",$alt2,"=",$full[25],";CHS={",$ref,"*=",substr($full[28],0,-1),$alt1,"=",substr($full[29],0,-1),", ",$alt2,"=",$full[29],";CLM={",$ref,"*=",substr($full[32],0,-1),$alt1,"=",substr($full[33],0,-1),", ",$alt2,"=",$full[33],";FIN={",$ref,"*=",substr($full[36],0,-1),$alt1,"=",substr($full[37],0,-1),", ",$alt2,"=",$full[37],";GBR={",$ref,"*=",substr($full[40],0,-1),$alt1,"=",substr($full[41],0,-1),", ",$alt2,"=",$full[41],";GIH={",$ref,"*=",substr($full[44],0,-1),$alt1,"=",substr($full[45],0,-1),", ",$alt2,"=",$full[45],";IBS={",$ref,"*=",substr($full[48],0,-1),$alt1,"=",substr($full[49],0,-1),", ",$alt2,"=",$full[49],";JPT={",$ref,"*=",substr($full[52],0,-1),$alt1,"=",substr($full[53],0,-1),", ",$alt2,"=",$full[53],";LWK={",$ref,"*=",substr($full[56],0,-1),$alt1,"=",substr($full[57],0,-1),", ",$alt2,"=",$full[57],";MKK={",$ref,"*=",substr($full[60],0,-1),$alt1,"=",substr($full[61],0,-1),", ",$alt2,"=",$full[61],";MXL={",$ref,"*=",substr($full[64],0,-1),$alt1,"=",substr($full[65],0,-1),", ",$alt2,"=",$full[65],";PUR={",$ref,"*=",substr($full[68],0,-1),$alt1,"=",substr($full[69],0,-1),", ",$alt2,"=",$full[69],";TSI={",$ref,"*=",substr($full[72],0,-1),$alt1,"=",substr($full[73],0,-1),", ",$alt2,"=",$full[73],";YRI={",$ref,"*=",substr($full[76],0,-1),$alt1,"=",substr($full[77],0,-1),", ",$alt2,"=",$full[77],";set=",$full[79],"\tGT\n";

}

@full=();

}until eof;

close(INFILE2);

close(INFILE1);

close(OUTFILE);

`cat ~/samples/header.population.vcf output.step5 > pooledNormal450.vcf`;

`rm population.tmp`;

g.

#!/usr/bin/perl -w

if ($#ARGV!=3){

print "\n\n\tUSAGE:\n\t\t#perl runContest.pl <script> <bam-list> <vcf-list> <bed-file>\n\n\n";

}elsif ($#ARGV==3){

$script=$ARGV[0];

$locBam=$ARGV[1];

$locVcf=$ARGV[2];

$bed=$ARGV[3];

@bam=`cat $locBam`;

@vcf=`cat $locVcf`;

chomp (@bam); chomp (@vcf);

open (CONTEST, ">RUN");

for ($i=0; $i<=$#vcf; ++$i){

print CONTEST "bash $script $bam[$i] $vcf[$i] $bed\n";

}

close (CONTEST);

}

h.

bash /home/venkatesh/contest/CONTEST.sh /common/Data/projects/internal/OSCC/bams/uniqMapped/allBamsForAnalysis/Sample.T.NovoV3.00.05.coordSorted.RG.uniqMapped.bam /common/Data/projects/internal/OSCC/exomeFilteredAllTargets/Sample.T.hd.vcf /common/Data/projects/internal/OSCC/allTarget.bed

i.

paste tumorList OUT tumorLines |awk '{ print $1"\t"$5"\t"$9"\t"$10"\t"(100*$9/$10) }'> contest.out

*5. Post-CRAVAT filter to short-list potentially driver genes*

=head

NAME: cravatLocusAnalysis.pl

INPUT: <path-to-zip-files> <sample-list> <callable-bed-file>

OUTPUT: sampleFreq.OUT

INPUT FORMAT: zip-files of the CRAVAT output, sample-list file containing the list of samples in lexicographical manner, callable-bed-file from costerwell

OUTPUT FORMAT: a file containing list of t-specific genes and its corresponding sample frequency

=cut

#!/usr/bin/perl -w

use List::MoreUtils 'true';

use List::MoreUtils qw(uniq);

use IPC::System::Simple qw(capture);

if ($#ARGV!=2){

print "\n\tUSAGE:\n\t\t# perl cravatLocusAnalysis.pl <path-to-zip-files> <sample-list> <callable-bed-file> \n\n\n";

}elsif ($#ARGV==2){

$home="/home/ganit";

$loc=$ARGV[0];

$list=$ARGV[1];

$bed=$ARGV[2];

chdir "$loc";

@listSample=`cat $list`;

chomp (@listSample);

@zip=`ls -1 $loc\/**\/*zip`;

chomp (@zip);

@samples=`ls -1 $loc\/**\/*zip\| cut -d\"\/\" -f6`;

chomp (@samples);

for ($i=0; $i<=$#zip; ++$i){

$pwd=substr($zip[$i],0,-4);

$samLoc=substr($zip[$i],0,-30);

chdir "$samLoc";

`unzip $zip[$i]`;

chdir "$pwd";

$tempo=capture(q@awk '{ if ((1*$12)>0 && (1*$12)<=0.35) print $2"\t"$3-1"\t"$3"\t"$1 }' Var*|sed 's/_.*//g'|sed 's/CHR/chr/g'@);

push (@t, $tempo);

chdir "$home";

}

chdir "$loc";

open (O, ">$loc/OUT");

print O @t;

close (O);

`sort -k1,1V -k2g $loc\/OUT>$loc\/OUT.sorted`;

capture(q@cut -f1-3 OUT.sorted |uniq -c|awk '{ print $2"\t"$3"\t"$4"\t"$1 }' > OUT.sorted.cnt@);

@cnt=`cut -f4 $loc\/OUT.sorted.cnt`;

chomp (@cnt);

$cntSum=0;

open (BED, ">$loc/OUT.bed");

for ($c=0; $c<=$#cnt; ++$c){

$cc=$c+1;

$cntSum=$cntSum+(1*$cnt[$c]);

@numSam=`head -$cntSum $loc\/OUT.sorted\|tail -$cnt[$c]\|cut -f4`;

$str=&makeString(@numSam, ",");

$numLine=`head -$cc $loc\/OUT.sorted.cnt\|tail -1`;

chomp ($numLine);

print BED "$numLine\t$str\n";

@numSam=();

}

close (BED);

capture(q@awk '{ print $4"\t"$5"\t"$7"\t"$9"\t"$14 }' OUT.ann|sort -k1 >OUT.ann.upd8.sorted@);

capture(q@cut -f1 OUT.ann.upd8.sorted|uniq>OUT.gene@);

@genes=capture(q@cat OUT.gene@);

chomp (@genes);

open (GFREQ, ">$loc/geneFreq.out");

print GFREQ "GENE\tPERCENTAGE\tSAMPLES\n";

open (LOCUS, ">$loc/locus.out");

print LOCUS "GENE\tCHR\tPOS\tNUM\tDEN\tRATIO\tSAMPLES\n";

for ($g=0; $g<=$#genes; ++$g){

$sum=0;@sink=();

@locus=`grep -i $genes[$g] OUT.ann.upd8.sorted`;

chomp (@locus);

for ($l=0; $l<=$#locus; ++$l){

$num=$den=$cal=0;

@splits=split '\t', $locus[$l];

@n=split ',', $splits[3];

@d=split ',', $splits[4];

for ($s1=0; $s1<=$#n; ++$s1){

if ($n[$s1] =~ m/...T/){

$set1=substr ($n[$s1],0,-1);

$nt1=true { /$set1/ } @d;

if ($nt1%2==0){

++$num;

}

}

}

for ($s2=0; $s2<=$#listSample; ++$s2){

if (($s2+1)%2==0){

$set2=substr ($listSample[$s2],0,-1);

$nt2=true { /$set2/ } @d;

if ($nt2%2==0){

++$den;

}

}

}

if ($den>0){

$cal=((1*$num)/(1*$den));

$sum=$sum+$cal;

}else{

$sum=$sum;

}

print "\t$genes[$g]\t$splits[1]\t$splits[2]\t$num\t$den\t$cal\n";

print LOCUS "$genes[$g]\t$splits[1]\t$splits[2]\t$num\t$den\t$cal\t$splits[3]\n";

push (@sink,@n);

}

@filterT=grep (/...T/, @sink);

@unique=uniq @filterT;

$uniqueSamples=&makeString(@unique, ",");

$avg=100*($sum/($#locus+1));

$sig=sprintf("%0.3f",$avg);

print "$genes[$g]\t$sig\n";

print GFREQ "$genes[$g]\t$sig\t$uniqueSamples\n";

}

close(LOCUS);

close(GFREQ);

}

sub makeString{

my $string="";

my @list=@_;

my $gap=pop (@_);

my $y;

chomp (@list);

for ($y=0; $y<$#list; ++$y){

$string=$string.$list[$y];

if ($y!=($#list-1)){

$string=$string.$gap;

}

}

return $string;

}

*6. Assessing disease significance of the variant-associated genes using MutSigCV*

*a. create maf file from vcf files*

\# Required files

\# 1. ensembl73_ENST_ENSG_strand_27112013_sorted.tsv

\# 2. mutationDict.txt

\# Extract chr, position, sample, ref, alt1, alt2, coverage

grep . /common/Data/projects/internal/OSCC/GATK/calls_UGv2.5.2/exomeFilteredSNPs/merged_VCFs/*[BN]_UGv2.5.2*vcf| sed 's/^.*merged_VCFs\///'|sed 's/_UGv2.5.2.PASS.onlySNPs.33MB.SS50MBu.TS62MBu.withCOSMIC.vcf:/\t/' |sed 's/\([AGTC]\)\t[0-9][0-9]*.*;DP=\([0-9][0-9]*\);.*PL\t\([0-9]\/[0-9]\):.*/\1\t\3\t\2/' | awk -v OFS="\t" '{if($7~/^0/) print $0"\t"$5; else if($7~/^1\/1/) print $0"\t"$6; else {split($6,alts,",");$6=alts[1];print $0"\t"alts[2]}}' | cut -f 1,2,3,5,6,8,9 > allBN_exomeFiltered_SNPs_intermediate1.tsv

grep . /common/Data/projects/internal/OSCC/GATK/calls_UGv2.5.2/exomeFilteredSNPs/merged_VCFs/*T_UGv2.5.2*vcf| sed 's/^.*merged_VCFs\///'|sed 's/_UGv2.5.2.PASS.onlySNPs.33MB.SS50MBu.TS62MBu.withCOSMIC.vcf:/\t/' |sed 's/\([AGTC]\)\t[0-9][0-9]*.*;DP=\([0-9][0-9]*\);.*PL\t\([0-9]\/[0-9]\):.*/\1\t\3\t\2/' | awk -v OFS="\t" '{if($7~/^0/) print $0"\t"$5; else if($7~/^1\/1/) print $0"\t"$6; else {split($6,alts,",");$6=alts[1];print $0"\t"alts[2]}}' | cut -f 1,2,3,5,6,8,9 > allT_exomeFiltered_SNPs_intermediate1.tsv

\# format files for VEP

sed 's/^.*chr//' allBN_exomeFiltered_SNPs_intermediate1.tsv | awk '{print $1"\t"$2"\t.\t"$3"\t"$4"\t.\tPASS\t."}' | sort -u > allBN_exomeFiltered_SNPs_intermediate1_vepInput.vcf

sed 's/^.*chr//' allT_exomeFiltered_SNPs_intermediate1.tsv | awk '{print $1"\t"$2"\t.\t"$3"\t"$4"\t.\tPASS\t."}' | sort -u > allT_exomeFiltered_SNPs_intermediate1_vepInput.vcf

\# Run VEP

perl5.10.1 /Apps/variant_effect_predictor/variant_effect_predictor.pl --cache -i allBN_exomeFiltered_SNPs_intermediate1_vepInput.vcf -o allBN_exomeFiltered_SNPs_intermediate1_vepOutput.txt --offline --force_overwrite --symbol --fork 6 --protein --plugin Condel,/home/saurabh/.vep/Plugins/config/Condel/config,b -sift b -polyphen b 1> vepLogs/allBN_veplog_$$.out 2> vepLogs/allBN_veplog_$$.err

perl5.10.1 /Apps/variant_effect_predictor/variant_effect_predictor.pl --cache -i allT_exomeFiltered_SNPs_intermediate1_vepInput.vcf -o allT_exomeFiltered_SNPs_intermediate1_vepOutput.txt --offline --force_overwrite --symbol --fork 6 --protein --plugin Condel,/home/saurabh/.vep/Plugins/config/Condel/config,b -sift b -polyphen b 1> vepLogs/allT_veplog_$$.out 2> vepLogs/allT_veplog_$$.err

\# format VEP output to delete intergenic variants, and extract GENE name, ENST id, ENSG id and vep annotation

grep -v "^#" allBN_exomeFiltered_SNPs_intermediate1_vepOutput.txt | cut -f 2,3,4,5,7,14 | sed 's/\t[^\t]*SYMBOL=\([^;][^;]*\)\([;$]*\)/\t\1\2/' | sed 's/;.*//' | sed 's/:/\t/' | awk '{print $1"\t"$2"\t"$3"\t"$4"\t"$5"\t"$7"\t"$6}' | sed 's/,.*//' | grep -v intergenic_variant > allBN_exomeFiltered_SNPs_intermediate1_vepOutput_formatted.txt

grep -v "^#" allT_exomeFiltered_SNPs_intermediate1_vepOutput.txt | cut -f 2,3,4,5,7,14 | sed 's/\t[^\t]*SYMBOL=\([^;][^;]*\)\([;$]*\)/\t\1\2/' | sed 's/;.*//' | sed 's/:/\t/' | awk '{print $1"\t"$2"\t"$3"\t"$4"\t"$5"\t"$7"\t"$6}' | sed 's/,.*//' | grep -v intergenic_variant > allT_exomeFiltered_SNPs_intermediate1_vepOutput_formatted.txt

\# add strand information

sort -k 5 allT_exomeFiltered_SNPs_intermediate1_vepOutput_formatted.txt |join -1 5 -2 1 - ensembl73_ENST_ENSG_strand_27112013_sorted.tsv |cut -f 2,3,4,6,7,8 |sort -u | sed 's/^/chr/' > allT_exomeFiltered_SNPs_intermediate1_vepOutput_formatted_withStrand.txt

sort -k 5 allBN_exomeFiltered_SNPs_intermediate1_vepOutput_formatted.txt |join -1 5 -2 1 - ensembl73_ENST_ENSG_strand_27112013_sorted.tsv |cut -f 2,3,4,6,7,8 |sort -u | sed 's/^/chr/' > allBN_exomeFiltered_SNPs_intermediate1_vepOutput_formatted_withStrand.txt

\# remove duplicate annotations for same locus, giving preference to nonsilent > silent > noncoding > null

join -1 1 -2 5 mutationDict.txt <(sort -k 5,5 allT_exomeFiltered_SNPs_intermediate1_vepOutput_formatted_withStrand.txt)|cut -f 2-| sort -u | awk 'NR>1{a[$2"#"$3"#"$4"#"$5"#"$6]=a[$2"#"$3"#"$4"#"$5"#"$6]?a[$2"#"$3"#"$4"#"$5"#"$6]";"$1:$1}END{for (i in a) print i"\t"a[i]}'|sed 's/#/\t/g' | sed 's/;null$//;s/null;//;s/noncoding;//;s/;silent//' > allT_exomeFiltered_SNPs_intermediate1_vepOutput_formatted_withStrand_formatted.txt

join -1 1 -2 5 mutationDict.txt <(sort -k 5,5 allBN_exomeFiltered_SNPs_intermediate1_vepOutput_formatted_withStrand.txt)|cut -f 2-| sort -u | awk 'NR>1{a[$2"#"$3"#"$4"#"$5"#"$6]=a[$2"#"$3"#"$4"#"$5"#"$6]?a[$2"#"$3"#"$4"#"$5"#"$6]";"$1:$1}END{for (i in a) print i"\t"a[i]}'|sed 's/#/\t/g' | sed 's/;null$//;s/null;//;s/noncoding;//;s/;silent//' > allBN_exomeFiltered_SNPs_intermediate1_vepOutput_formatted_withStrand_formatted.txt

\# merge VEP annotations with the intermediate files and add header, hence creating maf file for input to MutSig

#(echo -en "Tumor_Sample_Barcode\tChromosome\tStart_position\tEnd_position\tReference_Allele\tTumor_Seq_Allele1\tCoverage\tTumor_Seq_Allele2\tHugo_Symbol\tVariant_Classification\tStrand\tVariant_Type\n";awk 'FNR==NR{a[$1"\t"$2]=$3"\t"$4"\t"$5;next}{ if(a[$2"\t"$3]) print $0"\t"a[$2"\t"$3]"\tSNP"}' allT_exomeFiltered_SNPs_intermediate1_vepOutput_formatted_withStrand.txt allT_exomeFiltered_SNPs_intermediate1.tsv | awk '{print $1"\t"$2"\t"$3"\t"$3"\t"$4"\t"$5"\t"$6"\t"$7"\t"$8"\t"$9"\t"$10"\t"$11}') > allT_exomeFiltered_SNPs_intermediate1_vepOutput_formatted_withStrand_mutsigInput.maf

#(echo -en "Tumor_Sample_Barcode\tChromosome\tStart_position\tEnd_position\tReference_Allele\tTumor_Seq_Allele1\tCoverage\tTumor_Seq_Allele2\tHugo_Symbol\tVariant_Classification\tStrand\tVariant_Type\n";awk 'FNR==NR{a[$1"\t"$2]=$3"\t"$4"\t"$5;next}{ if(a[$2"\t"$3]) print $0"\t"a[$2"\t"$3]"\tSNP"}' allBN_exomeFiltered_SNPs_intermediate1_vepOutput_formatted_withStrand.txt allBN_exomeFiltered_SNPs_intermediate1.tsv | awk '{print $1"\t"$2"\t"$3"\t"$3"\t"$4"\t"$5"\t"$6"\t"$7"\t"$8"\t"$9"\t"$10"\t"$11}') > allBN_exomeFiltered_SNPs_intermediate1_vepOutput_formatted_withStrand_mutsigInput.maf

(echo -en "Tumor_Sample_Barcode\tChromosome\tStart_position\tEnd_position\tReference_Allele\tTumor_Seq_Allele1\tCoverage\tTumor_Seq_Allele2\tHugo_Symbol\tVariant_Classification\tStrand\tVariant_Type\n"; join -1 1 -2 1 <(awk '{print $2"#"$3"#"$5"\t"$1"\t"$4"\t"$6"\t"$7}' allT_exomeFiltered_SNPs_intermediate1.tsv|sort -k1,1) <(awk '{print $1"#"$2"#"$3"\t"$4"\t"$5"\t"$6}' allT_exomeFiltered_SNPs_intermediate1_vepOutput_formatted_withStrand_formatted.txt | sort -k 1,1)| sed 's/#/\t/g'|awk '{print $4"\t"$1"\t"$2"\t"$2"\t"$5"\t"$3"\t"$6"\t"$7"\t"$8"\t"$10"\t"$9"\tSNP"}') > allT_exomeFiltered_SNPs_intermediate1_vepOutput_formatted_withStrand_mutsigInput.maf

(echo -en "Tumor_Sample_Barcode\tChromosome\tStart_position\tEnd_position\tReference_Allele\tTumor_Seq_Allele1\tCoverage\tTumor_Seq_Allele2\tHugo_Symbol\tVariant_Classification\tStrand\tVariant_Type\n"; join -1 1 -2 1 <(awk '{print $2"#"$3"#"$5"\t"$1"\t"$4"\t"$6"\t"$7}' allBN_exomeFiltered_SNPs_intermediate1.tsv|sort -k1,1) <(awk '{print $1"#"$2"#"$3"\t"$4"\t"$5"\t"$6}' allBN_exomeFiltered_SNPs_intermediate1_vepOutput_formatted_withStrand_formatted.txt | sort -k 1,1)| sed 's/#/\t/g'|awk '{print $4"\t"$1"\t"$2"\t"$2"\t"$5"\t"$3"\t"$6"\t"$7"\t"$8"\t"$10"\t"$9"\tSNP"}') > allBN_exomeFiltered_SNPs_intermediate1_vepOutput_formatted_withStrand_mutsigInput.maf

#(echo -en "Tumor_Sample_Barcode\tChromosome\tStart_position\tEnd_position\tReference_Allele\tTumor_Seq_Allele1\tCoverage\tTumor_Seq_Allele2\tHugo_Symbol\tVariant_Classification\tStrand\tVariant_Type\n"; join -1 1 -2 2 <(sed 's/\t/#/' allT_exomeFiltered_SNPs_intermediate1_vepOutput_formatted_withStrand.txt | sort -k1,1) <(awk '{print $1"\t"$2"#"$3"\t"$4"\t"$5"\t"$6"\t"$7}' allT_exomeFiltered_SNPs_intermediate1.tsv | sort -k2,2) | sed 's/#/\t/' | awk '{print $6"\t"$1"\t"$2"\t"$2"\t"$7"\t"$8"\t"$9"\t"$10"\t"$3"\t"$4"\t"$5"\tSNP"}') > allT_exomeFiltered_SNPs_intermediate1_vepOutput_formatted_withStrand_mutsigInput.maf1

#(echo -en "Tumor_Sample_Barcode\tChromosome\tStart_position\tEnd_position\tReference_Allele\tTumor_Seq_Allele1\tCoverage\tTumor_Seq_Allele2\tHugo_Symbol\tVariant_Classification\tStrand\tVariant_Type\n"; join -1 1 -2 2 <(sed 's/\t/#/' allBN_exomeFiltered_SNPs_intermediate1_vepOutput_formatted_withStrand.txt | sort -k1,1) <(awk '{print $1"\t"$2"#"$3"\t"$4"\t"$5"\t"$6"\t"$7}' allBN_exomeFiltered_SNPs_intermediate1.tsv | sort -k2,2) | sed 's/#/\t/' | awk '{print $6"\t"$1"\t"$2"\t"$2"\t"$7"\t"$8"\t"$9"\t"$10"\t"$3"\t"$4"\t"$5"\tSNP"}') > allBN_exomeFiltered_SNPs_intermediate1_vepOutput_formatted_withStrand_mutsigInput.maf1

#(echo -en "Tumor_Sample_Barcode\tChromosome\tStart_position\tEnd_position\tReference_Allele\tTumor_Seq_Allele1\tCoverage\tTumor_Seq_Allele2\tHugo_Symbol\tVariant_Classification\tStrand\tVariant_Type\n";awk 'FNR==NR{a[$1"\t"$2]=$3"\t"$4"\t"$5;next}{ if(a[$2"\t"$3]) print $0"\t"a[$2"\t"$3]"\tSNP"}' allT_exomeFiltered_SNPs_intermediate1_vepOutput_formatted_withStrand.txt allT_exomeFiltered_SNPs_intermediate1.tsv | awk '{print $1"\t"$2"\t"$3"\t"$3"\t"$4"\t"$5"\t"$6"\t"$7"\t"$8"\t"$9"\t"$10"\t"$11}') > allT_exomeFiltered_SNPs_intermediate1_vepOutput_formatted_withStrand_mutsigInput.maf

#(echo -en "Tumor_Sample_Barcode\tChromosome\tStart_position\tEnd_position\tReference_Allele\tTumor_Seq_Allele1\tCoverage\tTumor_Seq_Allele2\tHugo_Symbol\tVariant_Classification\tStrand\tVariant_Type\n";awk 'FNR==NR{a[$1"\t"$2]=$3"\t"$4"\t"$5;next}{ if(a[$2"\t"$3]) print $0"\t"a[$2"\t"$3]"\tSNP"}' allBN_exomeFiltered_SNPs_intermediate1_vepOutput_formatted_withStrand.txt allBN_exomeFiltered_SNPs_intermediate1.tsv | awk '{print $1"\t"$2"\t"$3"\t"$3"\t"$4"\t"$5"\t"$6"\t"$7"\t"$8"\t"$9"\t"$10"\t"$11}') > allBN_exomeFiltered_SNPs_intermediate1_vepOutput_formatted_withStrand_mutsigInput.maf

\# remove ambiguous(multiple annotations for same gene i.e. diff ENSG id and strand) genes from maf file

grep -v "DHRS4-AS1\|KBTBD4\|MIR3615\|RBL1\|snoU13\|Y_RNA\|ZNF668" allBN_exomeFiltered_SNPs_intermediate1_vepOutput_formatted_withStrand_mutsigInput.maf > allBN_exomeFiltered_SNPs_intermediate1_vepOutput_formatted_withStrand_mutsigInput_woAmbGenes.maf

grep -v "DHRS4-AS1\|KBTBD4\|MIR3615\|RBL1\|snoU13\|Y_RNA\|ZNF668" allT_exomeFiltered_SNPs_intermediate1_vepOutput_formatted_withStrand_mutsigInput.maf > allT_exomeFiltered_SNPs_intermediate1_vepOutput_formatted_withStrand_mutsigInput_woAmbGenes.maf

\# softlinking the maf files for short name

ln -s $PWD/allBN_exomeFiltered_SNPs_intermediate1_vepOutput_formatted_withStrand_mutsigInput_woAmbGenes.maf allBN_exomeFiltered_SNPs.maf

ln -s $PWD/allT_exomeFiltered_SNPs_intermediate1_vepOutput_formatted_withStrand_mutsigInput_woAmbGenes.maf allT_exomeFiltered_SNPs.maf

\# create germline and somatic mafs

awk -F"\t" 'FNR==NR{sam=substr($1,1,3); a[sam"#"$2"#"$3"#"$5"#"$6"#"$8"#"$9]=1;next}{sam=substr($1,1,3);if(a[sam"#"$2"#"$3"#"$5"#"$6"#"$8"#"$9]==1) print }' allBN_exomeFiltered_SNPs.maf allT_exomeFiltered_SNPs.maf > allT_germline_exomeFiltered_SNPs.maf

(head -1 allT_exomeFiltered_SNPs.maf; awk -F"\t" 'FNR==NR{sam=substr($1,1,3); a[sam"#"$2"#"$3"#"$5"#"$6"#"$8"#"$9]=1;next}{sam=substr($1,1,3);if(a[sam"#"$2"#"$3"#"$5"#"$6"#"$8"#"$9]!=1) print }' allBN_exomeFiltered_SNPs.maf allT_exomeFiltered_SNPs.maf) > allT_somatic_exomeFiltered_SNPs.maf

\# cut -f 1,2,3,5,6,8,9

#Tumor_Sample_Barcode	Chromosome	Start_position	End_position	Reference_Allele	Tumor_Seq_Allele1	Coverage	Tumor_Seq_Allele2	Hugo_Symbol	Variant_Classification	Strand	Variant_Type

#OT3_T	chr10	100004360	100004360	G	A	48	G	R3HCC1L	noncoding	+	SNP

#OT7_T	chr10	100004360	100004360	G	A	38	G	R3HCC1L	noncoding	+	SNP

#OT9_T	chr10	100004360	100004360	G	A	48	G	R3HCC1L	noncoding	+	SNP

#OT21_T	chr10	100004360	100004360	G	A	29	G	R3HCC1L	noncoding	+	SNP

*b. depth filter the maf with respect to matched normal*

#repeat the following for all 48 samples

grep "$(cut -f1,2 /common/Data/projects/internal/OSCC/GATK/calls_UGv2.5.2/exomeFilteredSNPs/somatic/new/callableSNPsIndels/sample.depth.altFiltered.SNPs)" allT_somatic_exomeFiltered_SNPs.maf | grep -w sample >> somatic.callable.altFiltered.exomeFiltered.SNPs.maf

*c. comp.sh:*

#!/bin/sh

if [ $# -lt 1 ]

then

echo -en "Usage:\n\tbash $0 <mutations.tsv> > <outfile>\n";

exit 0;

fi

compA='T'

compT='A'

compC='G'

compG='C'

#Tumor_Sample_Barcode	Chromosome	Start_position	End_position	Reference_Allele	Tumor_Seq_Allele1	Coverage	Tumor_Seq_Allele2	Hugo_Symbol	Variant_Classification	Strand	Variant_Type

#OT1_T	chr1	15038	15038	A	C	25	A	WASH7P	splice_region_variant	-	SNP

#OT1_T	chr1	16968	16968	C	T	39	C	WASH7P	non_coding_exon_variant	-	SNP

echo -en "Hugo_Symbol\tChromosome\tStart_position\tEnd_position\tStrand\tVariant_Classification\tVariant_Type\tReference_Allele\tTumor_Seq_Allele1\tTumor_Seq_Allele2\tTumor_Sample_Barcode\tCoverage\n"

#head -1 $1

sed '1d' $1 | while read sample chr start end ref alt1 cov alt2 gene class strand type

#sed '1d' $1 | while read gene chr start end strand class type ref alt1 alt2 sample cov

do

if [ "$strand" = "+" ]

then

echo -en "$gene\t$chr\t$start\t$end\t$strand\t$class\t$type\t$ref\t$alt1\t$alt2\t$sample\t$cov\n"

else

REF="comp$ref"

ALT1="comp$alt1"

ALT2="comp$alt2"

echo -en "$gene\t$chr\t$start\t$end\t$strand\t$class\t$type\t"${!REF}"\t"${!ALT1}"\t"${!ALT2}"\t$sample\t$cov\n"

fi

done

*d. create coverage intermediate for mutsig input*

if [ $# -lt 1 ]

then

echo -en "Usage:\n\tbash $0 <maf file>\n"

exit 1

fi

maf=$1

id=`echo $maf | sed 's/.maf$//'`

\# Required files:

\# 1. hg19.genome

\# 2. hg19_GATK_Ref.fa

\# 3. comp.sh

(head -1 $maf; bash comp.sh $maf | sed '1d' | awk '{print $2"\t"$3-1"\t"$4"\t"$1"#("$8"->"$9")#"$6"#"$7"#"$11"\t"$5"\t"$12}'| slopBed -b 1 -g /common/Data/Internal/Others/Reference_genome/hg_19/hg19.genome |fastaFromBed -s -fi /common/Data/Internal/Others/Reference_genome/hg_19/hg19_GATK_Ref.fa -bed stdin -tab -fo stdout |sed 's/\t[AGTCacgt][acgtAGTC]*\t/\U\0/' | sed 's/^\(.*\):\([0-9][0-9]*\)-\([0-9][0-9]*\)(\([0-9][0-9]*\))\t\(.\)\(.\)\(.\)/\1\t\2\t\3\t\U\5\t\U\6\t\U\7\t\4/') | paste $maf - | sed '1d' | awk '{print $9"\t"$10"\t"$16"\t"$17"\t"$6"\t"$18"\t"$1"\t"$7"\t"$11}' > $id\_covIntermediate.tsv

*e. format the coverage file to meet mutsig's requirements.*

#!/usr/bin/perl

if(@ARGV < 2){

die "Usage:\n\tperl $0 <maf with coverage file> <outfile>\n";

}

($input, $outfile) = (@ARGV);

%comp = ('A' => 'T', 'T' => 'A', 'G' => 'C', 'C' => 'G');

@bases=('A','T','C','G');

foreach $b1 (@bases){

foreach $b2 (@bases){

foreach $b3 (@bases){

foreach $b4 (@bases){

push @categ, "$b1($b2->$b3)$b4"	if($b2 ne $b3);

}

}

}

}

#map {print $_,"\n"}	(@categ);

@samples=("OT1_T","OT2_T","OT3_T","OT4_T","OT6_T","OT7_T","OT8_T","OT9_T","OT10_T","OT11_T","OT12_T","OT14_T","OT17_T","OT18_T","OT19_T","OT20_T","OT21_T","OT22_T","OT23_T","OT24_T","OT25_T","OT26_T","OT27_T","OT28_T","OT29_T","OT30_T","OT31_T","OT32_T","OT33_T","OT34_T","OT37_T","OT38_T","OT40_T","OT41_T","OT42_T","OT43_T","OT44_T","OT45_T","OT46_T","OT47_T","OT48_T","OT49_T","OT50_T","OT51_T","OT52_T","OT53_T","OT54_T","OT55_T");

#open(IN, "allT_SamplesProveanGATKout.withNoncoding.withComp.coverage.txt") or die "$!\n";

open(IN, "$input") or die "$!\n";

print scalar(@categ),"\n";

while($line=<IN>){

chomp $line;

($gene, $classification, $left, $ref, $alt, $right, $sampleID, $cov, $strand) = split(/\t/, $line);

if($strand eq '-'){

$left=	$comp{$left};

$ref=	$comp{$ref};

$alt=	$comp{$alt};

$right=	$comp{$right};

}

$categ = "$left($ref->$alt)$right";

#WASH7P	splice_region_variant	C	A	C	C	OT1_T	5	-

#WASH7P	non_coding_exon_variant	G	C	T	A	OT1_T	7	-

#WASH7P	non_coding_exon_variant	G	G	A	C	OT1_T	14	-

if($classification eq "missense_variant" || $classification eq "nonsilent"){

$classification="nonsilent";

}elsif($classification eq "synonymous_variant" || $classification eq "silent"){

$classification="silent";

}elsif($classification eq "noncoding" || $classification eq "3_prime_UTR_variant" || $classification eq "5_prime_UTR_variant" || $classification eq "downstream_gene_variant" || $classification eq "intergenic_variant" || $classification eq "intron_variant" || $classification eq "mature_miRNA_variant" || $classification eq "nc_transcript_variant" || $classification eq "non_coding_exon_variant" || $classification eq "upstream_gene_variant"){

$classification="noncoding";

}else{

print "$line\tnull\n";

next;

}

$genes{"$gene"}=1;

$hash{"$gene\t$classification\t$categ\t$sampleID"} += $cov;

}

close IN;

open(OUT,">$outfile") or die "$!\n";

print OUT "gene\teffect\tcateg";

map {print OUT "\t$_" } (@samples);

print OUT "\n";

foreach $gene (sort keys %genes){

\#	print OUT "$key\t";

foreach $type ("noncoding","silent","nonsilent"){ #,"null"){

foreach $categ (@categ){

print OUT "$gene\t$type\t$categ";

foreach $sample (@samples){

if(!exists $hash{"$gene\t$type\t$categ\t$sample"}){

print OUT "\t0";

next

}

print OUT "\t",$hash{"$gene\t$type\t$categ\t$sample"}

}

print OUT "\n";

}

}

}

close OUT;

*7. Using MUSIC2 to calculate the background mutation rate (bmr) of genes*

*a. Prepare the roi.tsv file from merged exome coverage bed file (38MB, 50MB, 62MB)* using the

#prepareROI.pl:

#!/usr/bin/perl -w

if ($#ARGV!=1){

print "\n\n\tUSAGE:\n\t\t# perl prepareMusic2.pl <path-to-bed-file> <path-to-output>\n\n\n";

}elsif ($#ARGV==1){

$inL=$ARGV[0];

$outL=$ARGV[1];

open (OUT, ">$outL");

open (IN, $inL);

print OUT "chr\tstart\tstop\tgene_name\n";

do{

$tmp=<IN>;

chomp ($tmp);

@data=split '\t', $tmp;

\#	@gene=split '#', $data[-1];

$chr=substr ($data[0],3);

print OUT $chr,"\t",$data[1]+1,"\t",$data[2],"\t",$data[-1],"\n";

}until eof;

close (IN);

close (OUT);

}

#prepareROI.pl outputs:

#CHR START(1 based) STOP ENSG#GENE#STRAND without header

#Example:

#chr1	14362	14883	ENSG00000223972#DDX11L1#-1

#chr1	14362	14883	ENSG00000227232#WASH7P#+1

#chr1	14943	15064	ENSG00000227232#WASH7P#+1

#chr1	15671	15990	ENSG00000227232#WASH7P#+1

*b. Sort the ROI file based on chromosome positions.*

sort -k1,1V -k2,2g -k3,3g roi.tsv > roi.sorted.tsv

*c.Prepare the normal (either N, B or N+B) and tumor bam.*

#If more than one sample is involved, prepare a combined bam for both normal and tumor samples. For example MUSIC needs to be run with 10 samples, prepare 10 samples combined bam for normal (B+N) and 10 tumor (all tumor samples. if one sample has to be run, prepare bam from normal for the same sample and also its corresponding bam for its tumor. When preparing the bam file (either single bam or combined bam), use their corresponding index files using the script mergedBamNormal.sh and mergeBamTumor.sh

\# mergedBamNormal.sh:

samtools merge /common/Data/projects/internal/OSCC/bams/uniqMapped/allBamsForAnalysis/allNormalBam_dec14.bam /common/Data/projects/internal/OSCC/bams/uniqMapped/allBamsForAnalysis/OT1_N_runH1_NovoV3.00.05.coordSorted.RG.uniqMapped.bam /common/Data/projects/internal/OSCC/bams/uniqMapped/allBamsForAnalysis/OT2_N_runH1_NovoV3.00.05.coordSorted.RG.uniqMapped.bam /common/Data/projects/internal/OSCC/bams/uniqMapped/allBamsForAnalysis/OT3_B_merged_run6_9_H1_NovoV3.00.05.coordSorted.RG.uniqMapped.bam /common/Data/projects/internal/OSCC/bams/uniqMapped/allBamsForAnalysis/OT4_B_NE62MB_runGHHT5_NovoV3.00.05.coordSorted.RG.uniqMapped.bam /common/Data/projects/internal/OSCC/bams/uniqMapped/allBamsForAnalysis/OT6_N_runH1_NovoV3.00.05.coordSorted.RG.uniqMapped.bam /common/Data/projects/internal/OSCC/bams/uniqMapped/allBamsForAnalysis/OT7_B_NE62MB_runGHHT5_NovoV3.00.05.coordSorted.RG.uniqMapped.bam /common/Data/projects/internal/OSCC/bams/uniqMapped/allBamsForAnalysis/OT8_B_runGH1_NovoV3.00.05.coordSorted.RG.uniqMapped.bam /common/Data/projects/internal/OSCC/bams/uniqMapped/allBamsForAnalysis/OT9_B_NE62MB_runGHHT5_NovoV3.00.05.coordSorted.RG.uniqMapped.bam /common/Data/projects/internal/OSCC/bams/uniqMapped/allBamsForAnalysis/OT10_B_runGH1_NovoV3.00.05.coordSorted.RG.uniqMapped.bam /common/Data/projects/internal/OSCC/bams/uniqMapped/allBamsForAnalysis/OT11_B_runGH1_NovoV3.00.05.coordSorted.RG.uniqMapped.bam /common/Data/projects/internal/OSCC/bams/uniqMapped/allBamsForAnalysis/OT12_B_runGH1_NovoV3.00.05.coordSorted.RG.uniqMapped.bam /common/Data/projects/internal/OSCC/bams/uniqMapped/allBamsForAnalysis/OT14_B_merged_run14_6_GH1_NovoV3.00.05.coordSorted.RG.uniqMapped.bam /common/Data/projects/internal/OSCC/bams/uniqMapped/allBamsForAnalysis/OT17_B_runGH1_NovoV3.00.05.coordSorted.RG.uniqMapped.bam /common/Data/projects/internal/OSCC/bams/uniqMapped/allBamsForAnalysis/OT18_N_runH1_NovoV3.00.05.coordSorted.RG.uniqMapped.bam /common/Data/projects/internal/OSCC/bams/uniqMapped/allBamsForAnalysis/OT19_N_runH1_NovoV3.00.05.coordSorted.RG.uniqMapped.bam /common/Data/projects/internal/OSCC/bams/uniqMapped/allBamsForAnalysis/OT20_N__runH1_NovoV3.00.05.coordSorted.RG.uniqMapped.bam /common/Data/projects/internal/OSCC/bams/uniqMapped/allBamsForAnalysis/OT21_B__NE62MB_runGHHT5_NovoV3.00.05.coordSorted.RG.uniqMapped.bam /common/Data/projects/internal/OSCC/bams/uniqMapped/allBamsForAnalysis/OT22_N__runH1_NovoV3.00.05.coordSorted.RG.uniqMapped.bam /common/Data/projects/internal/OSCC/bams/uniqMapped/allBamsForAnalysis/OT23_B__runGH1_NovoV3.00.05.coordSorted.RG.uniqMapped.bam /common/Data/projects/internal/OSCC/bams/uniqMapped/allBamsForAnalysis/OT24_B__merged_run6_9_GH1_NovoV3.00.05.coordSorted.RG.uniqMapped.bam /common/Data/projects/internal/OSCC/bams/uniqMapped/allBamsForAnalysis/OT25_B_NE62MB_runGHHT5_NovoV3.00.05.coordSorted.RG.uniqMapped.bam /common/Data/projects/internal/OSCC/bams/uniqMapped/allBamsForAnalysis/OT26_N_runH1_NovoV3.00.05.coordSorted.RG.uniqMapped.bam /common/Data/projects/internal/OSCC/bams/uniqMapped/allBamsForAnalysis/OT27_B_runGH1_NovoV3.00.05.coordSorted.RG.uniqMapped.bam /common/Data/projects/internal/OSCC/bams/uniqMapped/allBamsForAnalysis/OT28_N_runH1_NovoV3.00.05.coordSorted.RG.uniqMapped.bam /common/Data/projects/internal/OSCC/bams/uniqMapped/allBamsForAnalysis/OT29_B_merged_run14_6_GH1_NovoV3.00.05.coordSorted.RG.uniqMapped.bam /common/Data/projects/internal/OSCC/bams/uniqMapped/allBamsForAnalysis/OT30_B_runGH1_NovoV3.00.05.coordSorted.RG.uniqMapped.bam /common/Data/projects/internal/OSCC/bams/uniqMapped/allBamsForAnalysis/OT31_B_NE62MB_runGHHT5_NovoV3.00.05.coordSorted.RG.uniqMapped.bam /common/Data/projects/internal/OSCC/bams/uniqMapped/allBamsForAnalysis/OT32_B_merged_run6_9_GH1_NovoV3.00.05.coordSorted.RG.uniqMapped.bam /common/Data/projects/internal/OSCC/bams/uniqMapped/allBamsForAnalysis/OT33_B_runGH1_NovoV3.00.05.coordSorted.RG.uniqMapped.bam /common/Data/projects/internal/OSCC/bams/uniqMapped/allBamsForAnalysis/OT34_B_runH_NovoV3.00.05.coordSorted.RG.uniqMapped.bam /common/Data/projects/internal/OSCC/bams/uniqMapped/allBamsForAnalysis/OT37_N_runH1_NovoV3.00.05.coordSorted.RG.uniqMapped.bam /common/Data/projects/internal/OSCC/bams/uniqMapped/allBamsForAnalysis/OT38_N_runH1_NovoV3.00.05.coordSorted.RG.uniqMapped.bam /common/Data/projects/internal/OSCC/bams/uniqMapped/allBamsForAnalysis/OT40_B_runGH1_NovoV3.00.05.coordSorted.RG.uniqMapped.bam /common/Data/projects/internal/OSCC/bams/uniqMapped/allBamsForAnalysis/OT41_N_runH1_NovoV3.00.05.coordSorted.RG.uniqMapped.bam /common/Data/projects/internal/OSCC/bams/uniqMapped/allBamsForAnalysis/OT42_B_NE62MB_runGHHT5_NovoV3.00.05.coordSorted.RG.uniqMapped.bam /common/Data/projects/internal/OSCC/bams/uniqMapped/allBamsForAnalysis/OT43_B_NE62MB_runGHHT5_NovoV3.00.05.coordSorted.RG.uniqMapped.bam /common/Data/projects/internal/OSCC/bams/uniqMapped/allBamsForAnalysis/OT44_B_merged_run5_GHRR1FCA_FCB_H1_NovoV3.00.05.coordSorted.RG.uniqMapped.bam /common/Data/projects/internal/OSCC/bams/uniqMapped/allBamsForAnalysis/OT45_B_runGH1_NovoV3.00.05.coordSorted.RG.uniqMapped.bam /common/Data/projects/internal/OSCC/bams/uniqMapped/allBamsForAnalysis/OT46_B_NE62MB_runGHHT5_NovoV3.00.05.coordSorted.RG.uniqMapped.bam /common/Data/projects/internal/OSCC/bams/uniqMapped/allBamsForAnalysis/OT47_B_NE62MB_runGHHT5_NovoV3.00.05.coordSorted.RG.uniqMapped.bam /common/Data/projects/internal/OSCC/bams/uniqMapped/allBamsForAnalysis/OT48_B_NE62MB_runGHHT5_NovoV3.00.05.coordSorted.RG.uniqMapped.bam /common/Data/projects/internal/OSCC/bams/uniqMapped/allBamsForAnalysis/Ot49_N_runH1_NovoV3.00.05.coordSorted.RG.uniqMapped.bam /common/Data/projects/internal/OSCC/bams/uniqMapped/allBamsForAnalysis/OT50_N_merged_run5_H4_NovoV3.00.05.coordSorted.RG.uniqMapped.bam /common/Data/projects/internal/OSCC/bams/uniqMapped/allBamsForAnalysis/OT51_N_runH1_NovoV3.00.05.coordSorted.RG.uniqMapped.bam /common/Data/projects/internal/OSCC/bams/uniqMapped/allBamsForAnalysis/OT52_N_runH1_NovoV3.00.05.coordSorted.RG.uniqMapped.bam /common/Data/projects/internal/OSCC/bams/uniqMapped/allBamsForAnalysis/OT53_N_runH1_NovoV3.00.05.coordSorted.RG.uniqMapped.bam /common/Data/projects/internal/OSCC/bams/uniqMapped/allBamsForAnalysis/OT54_N_runH1_NovoV3.00.05.coordSorted.RG.uniqMapped.bam /common/Data/projects/internal/OSCC/bams/uniqMapped/allBamsForAnalysis/OT55_B_NE62MB_runGHHT5_NovoV3.00.05.coordSorted.RG.uniqMapped.bam

samtools index /common/Data/projects/internal/OSCC/bams/uniqMapped/allBamsForAnalysis/allNormalBam_dec14.bam /common/Data/projects/internal/OSCC/bams/uniqMapped/allBamsForAnalysis/allNormalBam_dec14.bam.bai

\# mergeBamTumor.sh:

samtools merge /common/Data/projects/internal/OSCC/bams/uniqMapped/allBamsForAnalysis/allTumorBam_dec14.bam /common/Data/projects/internal/OSCC/bams/uniqMapped/allBamsForAnalysis/OT1_T_runH1_NovoV3.00.05.coordSorted.RG.uniqMapped.bam /common/Data/projects/internal/OSCC/bams/uniqMapped/allBamsForAnalysis/OT2_T_runH1_NovoV3.00.05.coordSorted.RG.uniqMapped.bam /common/Data/projects/internal/OSCC/bams/uniqMapped/allBamsForAnalysis/OT3_T_merged_run6_9_H1_NovoV3.00.05.coordSorted.RG.uniqMapped.bam /common/Data/projects/internal/OSCC/bams/uniqMapped/allBamsForAnalysis/OT4_T_NE62MB_runGHHT5_NovoV3.00.05.coordSorted.RG.uniqMapped.bam /common/Data/projects/internal/OSCC/bams/uniqMapped/allBamsForAnalysis/OT6_T_runH1_NovoV3.00.05.coordSorted.RG.uniqMapped.bam /common/Data/projects/internal/OSCC/bams/uniqMapped/allBamsForAnalysis/OT7_T_NE62MB_runGHHT5_NovoV3.00.05.coordSorted.RG.uniqMapped.bam /common/Data/projects/internal/OSCC/bams/uniqMapped/allBamsForAnalysis/OT8_T_runGH1_NovoV3.00.05.coordSorted.RG.uniqMapped.bam /common/Data/projects/internal/OSCC/bams/uniqMapped/allBamsForAnalysis/OT9_T_NE62MB_runGHHT5_NovoV3.00.05.coordSorted.RG.uniqMapped.bam /common/Data/projects/internal/OSCC/bams/uniqMapped/allBamsForAnalysis/OT10_T_runGH1_NovoV3.00.05.coordSorted.RG.uniqMapped.bam /common/Data/projects/internal/OSCC/bams/uniqMapped/allBamsForAnalysis/OT11_T_runGH1_NovoV3.00.05.coordSorted.RG.uniqMapped.bam /common/Data/projects/internal/OSCC/bams/uniqMapped/allBamsForAnalysis/OT12_T_runGH1_NovoV3.00.05.coordSorted.RG.uniqMapped.bam /common/Data/projects/internal/OSCC/bams/uniqMapped/allBamsForAnalysis/OT14_T_merged_run14_6_9_GH1_NovoV3.00.05.coordSorted.RG.uniqMapped.bam /common/Data/projects/internal/OSCC/bams/uniqMapped/allBamsForAnalysis/OT17_T_runGH1_NovoV3.00.05.coordSorted.RG.uniqMapped.bam /common/Data/projects/internal/OSCC/bams/uniqMapped/allBamsForAnalysis/OT18_T_runH1_NovoV3.00.05.coordSorted.RG.uniqMapped.bam /common/Data/projects/internal/OSCC/bams/uniqMapped/allBamsForAnalysis/OT19_T_runH1_NovoV3.00.05.coordSorted.RG.uniqMapped.bam /common/Data/projects/internal/OSCC/bams/uniqMapped/allBamsForAnalysis/OT20_T_runH1_NovoV3.00.05.coordSorted.RG.uniqMapped.bam /common/Data/projects/internal/OSCC/bams/uniqMapped/allBamsForAnalysis/OT21_T_NE62MB_runGHHT5_NovoV3.00.05.coordSorted.RG.uniqMapped.bam /common/Data/projects/internal/OSCC/bams/uniqMapped/allBamsForAnalysis/OT22_T_runH1_NovoV3.00.05.coordSorted.RG.uniqMapped.bam /common/Data/projects/internal/OSCC/bams/uniqMapped/allBamsForAnalysis/OT23_T_runGH1_NovoV3.00.05.coordSorted.RG.uniqMapped.bam /common/Data/projects/internal/OSCC/bams/uniqMapped/allBamsForAnalysis/OT24_T_merged_run6_9_GH1_NovoV3.00.05.coordSorted.RG.uniqMapped.bam /common/Data/projects/internal/OSCC/bams/uniqMapped/allBamsForAnalysis/OT25_T_NE62MB_runGHHT5_NovoV3.00.05.coordSorted.RG.uniqMapped.bam /common/Data/projects/internal/OSCC/bams/uniqMapped/allBamsForAnalysis/OT26_T_runH1_NovoV3.00.05.coordSorted.RG.uniqMapped.bam /common/Data/projects/internal/OSCC/bams/uniqMapped/allBamsForAnalysis/OT27_T_merged_runGH1_GHRR1FCA_FCB_NovoV3.00.05.coordSorted.RG.uniqMapped.bam /common/Data/projects/internal/OSCC/bams/uniqMapped/allBamsForAnalysis/OT28_T_runH1_NovoV3.00.05.coordSorted.RG.uniqMapped.bam /common/Data/projects/internal/OSCC/bams/uniqMapped/allBamsForAnalysis/OT29_T_merged_run14_6_9_GH1_NovoV3.00.05.coordSorted.RG.uniqMapped.bam /common/Data/projects/internal/OSCC/bams/uniqMapped/allBamsForAnalysis/OT30_T_runGH1_NovoV3.00.05.coordSorted.RG.uniqMapped.bam /common/Data/projects/internal/OSCC/bams/uniqMapped/allBamsForAnalysis/OT31_T_NE62MB_runGHHT5_NovoV3.00.05.coordSorted.RG.uniqMapped.bam /common/Data/projects/internal/OSCC/bams/uniqMapped/allBamsForAnalysis/OT32_T_merged_run6_9_GH1_NovoV3.00.05.coordSorted.RG.uniqMapped.bam /common/Data/projects/internal/OSCC/bams/uniqMapped/allBamsForAnalysis/OT33_T_runGH1_NovoV3.00.05.coordSorted.RG.uniqMapped.bam /common/Data/projects/internal/OSCC/bams/uniqMapped/allBamsForAnalysis/OT34_T_runH_NovoV3.00.05.coordSorted.RG.uniqMapped.bam /common/Data/projects/internal/OSCC/bams/uniqMapped/allBamsForAnalysis/OT37_T_runH1_NovoV3.00.05.coordSorted.RG.uniqMapped.bam /common/Data/projects/internal/OSCC/bams/uniqMapped/allBamsForAnalysis/OT38_T_runH1_NovoV3.00.05.coordSorted.RG.uniqMapped.bam /common/Data/projects/internal/OSCC/bams/uniqMapped/allBamsForAnalysis/OT40_T_runGH1_NovoV3.00.05.coordSorted.RG.uniqMapped.bam /common/Data/projects/internal/OSCC/bams/uniqMapped/allBamsForAnalysis/OT41_T_runH1_NovoV3.00.05.coordSorted.RG.uniqMapped.bam /common/Data/projects/internal/OSCC/bams/uniqMapped/allBamsForAnalysis/OT42_T_NE62MB_runGHHT5_NovoV3.00.05.coordSorted.RG.uniqMapped.bam /common/Data/projects/internal/OSCC/bams/uniqMapped/allBamsForAnalysis/OT43_T_NE62MB_runGHHT5_NovoV3.00.05.coordSorted.RG.uniqMapped.bam /common/Data/projects/internal/OSCC/bams/uniqMapped/allBamsForAnalysis/OT44_T_merged_run5_H1_NovoV3.00.05.coordSorted.RG.uniqMapped.bam /common/Data/projects/internal/OSCC/bams/uniqMapped/allBamsForAnalysis/OT45_T_runGH1_NovoV3.00.05.coordSorted.RG.uniqMapped.bam /common/Data/projects/internal/OSCC/bams/uniqMapped/allBamsForAnalysis/OT46_T_NE62MB_runGHHT5_NovoV3.00.05.coordSorted.RG.uniqMapped.bam /common/Data/projects/internal/OSCC/bams/uniqMapped/allBamsForAnalysis/OT47_T_NE62MB_runGHHT5_NovoV3.00.05.coordSorted.RG.uniqMapped.bam /common/Data/projects/internal/OSCC/bams/uniqMapped/allBamsForAnalysis/OT48_T_NE62MB_runGHHT5_NovoV3.00.05.coordSorted.RG.uniqMapped.bam /common/Data/projects/internal/OSCC/bams/uniqMapped/allBamsForAnalysis/OT49_T_runH1_NovoV3.00.05.coordSorted.RG.uniqMapped.bam /common/Data/projects/internal/OSCC/bams/uniqMapped/allBamsForAnalysis/OT50_T_merged_run3_GH1_NovoV3.00.05.coordSorted.RG.uniqMapped.bam /common/Data/projects/internal/OSCC/bams/uniqMapped/allBamsForAnalysis/OT51_T_runH1_NovoV3.00.05.coordSorted.RG.uniqMapped.bam /common/Data/projects/internal/OSCC/bams/uniqMapped/allBamsForAnalysis/OT52_T_runH1_NovoV3.00.05.coordSorted.RG.uniqMapped.bam /common/Data/projects/internal/OSCC/bams/uniqMapped/allBamsForAnalysis/OT53_T_runH1_NovoV3.00.05.coordSorted.RG.uniqMapped.bam /common/Data/projects/internal/OSCC/bams/uniqMapped/allBamsForAnalysis/OT54_T_runH1_NovoV3.00.05.coordSorted.RG.uniqMapped.bam /common/Data/projects/internal/OSCC/bams/uniqMapped/allBamsForAnalysis/OT55_T_NE62MB_runGHHT5_NovoV3.00.05.coordSorted.RG.uniqMapped.bam

samtools index /common/Data/projects/internal/OSCC/bams/uniqMapped/allBamsForAnalysis/allTumorBam_dec14.bam /common/Data/projects/internal/OSCC/bams/uniqMapped/allBamsForAnalysis/allTumorBam_dec14.bam.bai

The reference file to be used, for example hg19, has to take care of uppercase/lowercase issues. For example, if any base is present in lower case letter, it may spit out an error later.

music2 bmr calc-covg-helper --normal-tumor-bam-pair="NORMAL	allNormalBam.bam	allTumorBam.bam" --roi-file=roi.tsv --reference-sequence=hg19_GATK_Ref.fa --output-file=/home/ganit/MUSIC2/roi_covgs/NORMAL.covg --normal-min-depth=6 --tumor-min-depth=8 --min-mapq=20 --bp-class-types=AT,CpG,GC

*d. Run the command*

music2 bmr calc-covg --bam-list pooledBamList --output-dir ~/MUSIC2 --reference-sequence hg19_GATK_Ref.fa --roi-file roi.tsv

e. *Prepare maf file*

#maf file always has to correspond the normal (either N, B or N+B) samples. If more than one sample is involved, prepare a maf from all those normal samples. maf file can be used that is an output from any annotation tool, for example VEP, Annovar or oncotator. The mandatory fields for MUSIC in the maf file are; chr notated lines. Replace the hugo symbol with the combination of ENSG#HUGO using join command(use hugoSort and the altered maf files), replace the oncotator variant classification with the maf classification using join command(use maf and variantClassification files CMD: join -1 1 -2 1 -a 1 tmpNormal.sorted variantClassification), remove duplicates.

f. Run the command

music2 bmr calc-bmr --bam-list pooledBamList --maf-file normal.ensg.ann.variantClassificationConverted.dupRem.maf --output-dir ~/MUSIC2 --reference-sequence hg19_GATK_Ref.fa --roi-file roi.tsv

g. Run the command

R --slave --args < Smg.pm.R gene_mrs pval smg_test 1 0

#pval file has the list of genes with their p-values of different tests

*8. Validation of SNPs found using GATK by Omni SNP Genotyping array*

*a. Change to single letter code*

if [ $# -lt 1 ]

then

echo -e "Usage:\n\tbash $0 <input_bed>"

exit 0

fi

outfile=`echo $1 | sed 's/.bed$//'`

sed 's/A#A#G#.*/R/;s/A#G#A#.*/R/;s/A#G#G#.*/R/;s/G#A#G#.*/R/;s/G#G#A#.*/R/;s/G#A#A#.*/R/' $1 | sort -u > $outfile.singleLetter.bed

sed -i 's/A#A#C#.*/M/;s/A#C#A#.*/M/;s/A#C#C#.*/M/;s/C#A#C#.*/M/;s/C#C#A#.*/M/;s/C#A#A#.*/M/' $outfile.singleLetter.bed

sed -i 's/A#A#T#.*/W/;s/A#T#A#.*/W/;s/A#T#T#.*/W/;s/T#A#T#.*/W/;s/T#T#A#.*/W/;s/T#A#A#.*/W/' $outfile.singleLetter.bed

sed -i 's/T#T#G#.*/K/;s/T#G#T#.*/K/;s/T#G#G#.*/K/;s/G#T#G#.*/K/;s/G#G#T#.*/K/;s/G#T#T#.*/K/' $outfile.singleLetter.bed

sed -i 's/T#T#C#.*/Y/;s/T#C#T#.*/Y/;s/T#C#C#.*/Y/;s/C#T#C#.*/Y/;s/C#C#T#.*/Y/;s/C#T#T#.*/Y/' $outfile.singleLetter.bed

sed -i 's/C#C#G#.*/S/;s/C#G#C#.*/S/;s/C#G#G#.*/S/;s/G#C#G#.*/S/;s/G#G#C#.*/S/;s/G#C#C#.*/S/' $outfile.singleLetter.bed

sort -u $outfile.singleLetter.bed > $outfile.singleLetter.bed1 #; mv t $outfile.singleLetter.bed

*b. comp.pl:*

#chr1	82153	82154	T#C#OT1_N#+

#chr1	534246	534247	T#C#OT1_N#+

if(@ARGV < 1){

die "Usage:\n\tperl $0 <input.bed>\n";

}

open(IN, "$ARGV[0]") or die "$!\n";

open(OUT, ">$ARGV[0].comp.bed") or die "$!\n";

while($line = <IN>){

chomp($line);

($id, $a2, $sample, $strand) = split(/#/, $line);

($chr, $start, $end, $a1) = split(/\t/, $id);

if($strand eq '-'){

$a1 =~ tr/AGTC/TCAG/;

$a2 =~ tr/AGTC/TCAG/;

}

print OUT "$chr\t$start\t$end\t$a1#$a2#$sample#$strand\n"

}

close IN;

close OUT;

*c.*

\# formatting array outputs

sed '1,10d' /common/LAB/Lab_files/Data_files/Microarray/Genotyping/Projects/Final_Reports_30_04_2014/2011_02_10_GS_LCG_2-5_4_Human_Tongue_Cancer_B0_FinalReport_30_04_2014.txt | egrep -v -w "(OT34_T|OT34_B)" | awk '{print "chr"$5"\t"$6-1"\t"$6"\t"$7"#"$2"#"$8}'|sed 's/.\([AGTC]\)\/\([AGTC]\)./\1#\2/'| grep "[AGTC]#[AGTC]" |grep -v "\-#" | grep -v "\-1" | sort -u | sortBed > B0_withSNP.bed

sed '1,10d' /common/LAB/Lab_files/Data_files/Microarray/Genotyping/Projects/Final_Reports_30_04_2014/2011_10_17_GS_LCG_2-5_8_1_Human_Tongue_Cancer_B2_FinalReport_30_04_2014.txt | awk '{print "chr"$5"\t"$6-1"\t"$6"\t"$7"#"$2"#"$8}'|sed 's/.\([AGTC]\)\/\([AGTC]\)./\1#\2/'| grep "[AGTC]#[AGTC]" |grep -v "\-#" | grep -v "\-1" | sort -u | sortBed > B2_withSNP.bed

sed '1,10d' /common/LAB/Lab_files/Data_files/Microarray/Genotyping/Projects/Final_Reports_30_04_2014/2012_12_03_GS_LCG_2-5_8_1_Human_Tongue_Cancer_B3_FinalReport_30_04_2014.txt | awk '{print "chr"$5"\t"$6-1"\t"$6"\t"$7"#"$2"#"$8}'|sed 's/.\([AGTC]\)\/\([AGTC]\)./\1#\2/'| grep "[AGTC]#[AGTC]" |grep -v "\-#" | grep -v "\-1" | sort -u | sortBed > B3_withSNP.bed

sed '1,10d' /common/LAB/Lab_files/Data_files/Microarray/Genotyping/Projects/Final_Reports_30_04_2014/2013_06_14_GS_LCG_2-5_8_1-1_Human_Tongue_Cancer_B4_FinalReport_30_04_2014.txt | awk '{print "chr"$5"\t"$6-1"\t"$6"\t"$7"#"$2"#"$8}'|sed 's/.\([AGTC]\)\/\([AGTC]\)./\1#\2/'| grep "[AGTC]#[AGTC]" |grep -v "\-#" | grep -v "\-1" | sort -u | sortBed > B4_withSNP.bed

sed '1,10d' /common/LAB/Lab_files/Data_files/Microarray/Genotyping/Projects/Final_Reports_30_04_2014/2013_12_04_GS_LCG_2-5_8_1-1_Human_Tongue_Cancer_B5_FinalReport_30_04_2014.txt | awk '{print "chr"$5"\t"$6-1"\t"$6"\t"$7"#"$2"#"$8}'|sed 's/.\([AGTC]\)\/\([AGTC]\)./\1#\2/'| grep "[AGTC]#[AGTC]" |grep -v "\-#" | grep -v "\-1" | sort -u | sortBed > B5_withSNP.bed

sed '1,10d' /common/LAB/Lab_files/Data_files/Microarray/Genotyping/Projects/Final_Reports_30_04_2014/2013_12_10_GS_LCG_2-5_8_1-1_Human_Tongue_Cancer_B6_FinalReport_9_05_2014_new.txt | awk '{print "chr"$5"\t"$6-1"\t"$6"\t"$7"#"$2"#"$8}'|sed 's/.\([AGTC]\)\/\([AGTC]\)./\1#\2/'| grep "[AGTC]#[AGTC]" |grep -v "\-#" | grep -v "\-1" | sort -u | sortBed > B6_withSNP.bed

sed '1,10d' /common/LAB/Lab_files/Data_files/Microarray/Genotyping/Projects/Final_Reports_30_04_2014/2013_12_18_GS_LCG_2-5_8_1-1_Human_Tongue_Cancer_B7_FinalReport_30_04_2014.txt | awk '{print "chr"$5"\t"$6-1"\t"$6"\t"$7"#"$2"#"$8}'|sed 's/.\([AGTC]\)\/\([AGTC]\)./\1#\2/'| grep "[AGTC]#[AGTC]" |grep -v "\-#" | grep -v "\-1" | sort -u | sortBed > B7_withSNP.bed

awk -F"\t" '{print > $6"_array_withSNP.bed" }' B0_withSNP.bed

awk -F"\t" '{print > $6"_array_withSNP.bed" }' B2_withSNP.bed

awk -F"\t" '{print > $6"_array_withSNP.bed" }' B3_withSNP.bed

awk -F"\t" '{print > $6"_array_withSNP.bed" }' B4_withSNP.bed

awk -F"\t" '{print > $6"_array_withSNP.bed" }' B5_withSNP.bed

awk -F"\t" '{print > $6"_array_withSNP.bed" }' B6_withSNP.bed

awk -F"\t" '{print > $6"_array_withSNP.bed" }' B7_withSNP.bed

\# filtering mergedExome, callable, adding Ref, extracting only SNPs

perl comp.pl sample_array_withSNP.bed

intersectBed -a sample_array_withSNP.comp.bed -b mergedExome.bed -wa | sort -u > sample_array_mergedExomeFiltered.bed

intersectBed -a sample_array_mergedExomeFiltered.bed -b /common/Data/projects/internal/OSCC/GATK/CallableLoci/realignedDupRem/Callable.Loci.only.1.april.2014/sample.callable.formatted.bed.callable.loci.only -wa > sample_array_mergedExomeFiltered_callableFilter.bed

fastaFromBed -bed sample_array_mergedExomeFiltered_callableFilter.bed -fi /common/Data/Internal/Others/Reference_genome/hg_19/hg19_GATK_Ref.fa -fo stdout -tab | sed 's/[:-]/\t/g' | awk '{print $1"\t"$2"\t"$3"\t"toupper($4)}' | intersectBed -a stdin -b sample_array_mergedExomeFiltered_callableFilter.bed -wo | sort -u | awk '{print $1"\t"$2"\t"$3"\t"$4"#"$8}' > sample_array_mergedExomeFiltered_callableFilter_withRef.bed

sed 's/#/\t/g' sample_array_mergedExomeFiltered_callableFilter_withRef.bed | awk '{if($4!=$5 || $4!=$6) {print $1"\t"$2"\t"$3"\t"$4"#"$5"#"$6"#"$7"#"$8} }' > sample_array_mergedExomeFiltered_callableFilter_withRef_onlySNPs.bed

sed 's/#/\t/g' sample_array_mergedExomeFiltered_callableFilter_withRef.bed | awk '{if(!($4!=$5 || $4!=$6)) {print $1"\t"$2"\t"$3"\t"$4"#"$5"#"$6"#"$7"#"$8} }' > sample_array_mergedExomeFiltered_callableFilter_withRef_onlyNoSNPs.bed

subtractBed -a sample_array_mergedExomeFiltered_callableFilter_withRef.bed -b sample_seq_exomeFiltered.callableFilter.bed | awk '{print $1"\t"$2"\t"$3}' > sample_seq_exomeFiltered.callabledFilter.noSNPs.bed

bash changeToSingleLetterCode.sh sample_array_mergedExomeFiltered_callableFilter_withRef_onlySNPs.bed

bash changeToSingleLetterCode.sh sample_seq_exomeFiltered.callableFilter.bed

echo -en "sample\t"; cat array/sample_array_mergedExomeFiltered_callableFilter_withRef_onlySNPs.singleLetter.bed seq/callableFiltered/sample_seq_exomeFiltered.callableFilter.singleLetter.bed | sort | uniq -D | uniq | wc -l

*9. Pre-processing of expression intensities and differential expression analyses*

\# dasl & nondasl expression array analyses using R:-

\# Working directory: /Apps/expression/genewise

\# Shown below are steps to be followed for analysis of WG-DASL expression array.

\# The same steps are to be followed for analysis of WG expression array.

\# A variety of normalization and transformation methods are implemented, and with each

\# combination, the IAC (inter-array correlation coefficient) is calculated. The combination

\# yielding the highest IAC is processed for batch correction using Combat.

\# Data directory - /common/LAB/Lab_files/Data_files/Microarray/Gene_Expression/GX_Human_Tongue_Cancer_Analysis/No_Norm_data_lumi

ln -s /common/LAB/Lab_files/Data_files/Microarray/Gene_Expression/GX_Human_Tongue_Cancer_Analysis/No_Norm_data_lumi/DASL_Sample_Gene_Profile_no_norm.txt DASL.raw.expr.txt

ln -s /common/LAB/Lab_files/Data_files/Microarray/Gene_Expression/GX_Human_Tongue_Cancer_Analysis/No_Norm_data_lumi/NonDASL_Sample_Gene_Profile_no_norm.txt NonDASL.raw.expr.txt

R

library(lumi)

fileName <- 'DASL.raw.expr.txt'

dasl.lumi <- lumiR.batch(fileName)

\# VST and Quantile/SSN/RSN/RI/LOESS/VSN

dasl.lumi.VST <-lumiT(dasl.lumi)

dasl.lumi.VST.Q <- lumiN(dasl.lumi.VST,method='quantile')

dasl.lumi.VST.SSN <- lumiN(dasl.lumi.VST,method='ssn')

dasl.lumi.VST.RSN <- lumiN(dasl.lumi.VST,method='rsn')

dasl.lumi.VST.RI <- lumiN(dasl.lumi.VST,method='rankinvariant')

dasl.lumi.VST.LOESS <- lumiN(dasl.lumi.VST,method='loess')

dasl.lumi.VST.VSN <- lumiN(dasl.lumi.VST, method='vsn')

write.exprs(dasl.lumi,"dasl.raw.expr.txt")

write.exprs(dasl.lumi.VST,"dasl.VST.expr.txt")

write.exprs(dasl.lumi.VST.Q,"dasl.VST.Q.expr.txt")

write.exprs(dasl.lumi.VST.SSN,"dasl.VST.SSN.expr.txt")

write.exprs(dasl.lumi.VST.RSN,"dasl.VST.RSN.expr.txt")

write.exprs(dasl.lumi.VST.RI,"dasl.VST.RI.expr.txt")

write.exprs(dasl.lumi.VST.LOESS,"dasl.VST.LOESS.expr.txt")

write.exprs(dasl.lumi.VST.VSN,"dasl.VST.VSN.expr.txt")

x=read.table("dasl.raw.expr.txt",header=TRUE)

y=as.matrix(cor(x))

mean(y)

#0.7717528

x=read.table("dasl.VST.expr.txt",header=TRUE)

y=as.matrix(cor(x))

mean(y)

#0.7923227

x=read.table("dasl.VST.Q.expr.txt",header=TRUE)

y=as.matrix(cor(x))

mean(y)

#0.8156327

x=read.table("dasl.VST.SSN.expr.txt",header=TRUE)

y=as.matrix(cor(x))

mean(y)

#0.8101744

x=read.table("dasl.VST.RSN.expr.txt",header=TRUE)

y=as.matrix(cor(x))

mean(y)

#0.8160936

x=read.table("dasl.VST.RI.expr.txt",header=TRUE)

y=as.matrix(cor(x))

mean(y)

#0.8060439

x=read.table("dasl.VST.LOESS.expr.txt",header=TRUE)

y=as.matrix(cor(x))

mean(y)

#0.851167

x=read.table("dasl.VST.VSN.expr.txt",header=TRUE)

y=as.matrix(cor(x))

mean(y)

#0.7922153

\# LRT and Quantile/SSN/RSN/RI/LOESS/VSN

dasl.lumi.LRT <- lumiT(dasl.lumi,method='log2')

dasl.lumi.LRT.Q <- lumiN(dasl.lumi.LRT,method='quantile')

dasl.lumi.LRT.SSN <- lumiN(dasl.lumi.LRT,method='ssn')

dasl.lumi.LRT.RSN <- lumiN(dasl.lumi.LRT, method='rsn')

dasl.lumi.LRT.RI <- lumiN(dasl.lumi.LRT, method='rankinvariant')

dasl.lumi.LRT.LOESS <- lumiN(dasl.lumi.LRT, method='loess')

dasl.lumi.LRT.VSN <- lumiN(dasl.lumi.LRT, method='vsn') #Error

write.exprs(dasl.lumi.LRT,"dasl.LRT.expr.txt")

write.exprs(dasl.lumi.LRT.Q,"dasl.LRT.Q.expr.txt")

write.exprs(dasl.lumi.LRT.SSN,"dasl.LRT.SSN.expr.txt")

write.exprs(dasl.lumi.LRT.RSN,"dasl.LRT.RSN.expr.txt")

write.exprs(dasl.lumi.LRT.RI,"dasl.LRT.RI.expr.txt")

write.exprs(dasl.lumi.LRT.LOESS,"dasl.LRT.LOESS.expr.txt")

x=read.table("dasl.LRT.expr.txt",header=TRUE)

y=as.matrix(cor(x))

mean(y)

#0.813508

x=read.table("dasl.LRT.Q.expr.txt",header=TRUE)

y=as.matrix(cor(x))

mean(y)

#0.8133523

x=read.table("dasl.LRT.SSN.expr.txt",header=TRUE)

y=as.matrix(cor(x))

mean(y)

#0.813897

x=read.table("dasl.LRT.RSN.expr.txt",header=TRUE)

y=as.matrix(cor(x))

mean(y)

#0.8096424

x=read.table("dasl.LRT.RI.expr.txt",header=TRUE)

y=as.matrix(cor(x))

mean(y)

#0.8168109

x=read.table("dasl.LRT.LOESS.expr.txt",header=TRUE)

y=as.matrix(cor(x))

mean(y)

#0.8152007

\# CRT and Quantile/SSN/RSN/RI/LOESS/VSN

dasl.lumi.CRT <- lumiT(dasl.lumi,method='cubicRoot')

dasl.lumi.CRT.Q <- lumiN(dasl.lumi.CRT,method='quantile')

dasl.lumi.CRT.SSN <- lumiN(dasl.lumi.CRT,method='ssn')

dasl.lumi.CRT.RSN <- lumiN(dasl.lumi.CRT, method='rsn')

dasl.lumi.CRT.RI <- lumiN(dasl.lumi.CRT, method='rankinvariant')

dasl.lumi.CRT.LOESS <- lumiN(dasl.lumi.CRT, method='loess')

dasl.lumi.CRT.VSN <- lumiN(dasl.lumi.CRT, method='vsn')

dasl.lumi.VSN <- lumiN(dasl.lumi, method='vsn')

write.exprs(dasl.lumi.VSN,"dasl.VSN.expr.txt")

write.exprs(dasl.lumi.CRT,"dasl.CRT.expr.txt")

write.exprs(dasl.lumi.CRT.Q,"dasl.CRT.Q.expr.txt")

write.exprs(dasl.lumi.CRT.SSN,"dasl.CRT.SSN.expr.txt")

write.exprs(dasl.lumi.CRT.RSN,"dasl.CRT.RSN.expr.txt")

write.exprs(dasl.lumi.CRT.RI,"dasl.CRT.RI.expr.txt")

write.exprs(dasl.lumi.CRT.LOESS,"dasl.CRT.LOESS.expr.txt")

write.exprs(dasl.lumi.CRT.VSN,"dasl.CRT.VSN.expr.txt")

x=read.table("dasl.CRT.expr.txt",header=TRUE)

y=as.matrix(cor(x))

mean(y)

#0.7717528

x=read.table("dasl.CRT.Q.expr.txt",header=TRUE)

y=as.matrix(cor(x))

mean(y)

#0.7715799

x=read.table("dasl.CRT.SSN.expr.txt",header=TRUE)

y=as.matrix(cor(x))

mean(y)

#0.7717528

x=read.table("dasl.CRT.RSN.expr.txt",header=TRUE)

y=as.matrix(cor(x))

mean(y)

#0.7703628

x=read.table("dasl.CRT.RI.expr.txt",header=TRUE)

y=as.matrix(cor(x))

mean(y)

#0.7717528

x=read.table("dasl.CRT.LOESS.expr.txt",header=TRUE)

y=as.matrix(cor(x))

mean(y)

#NA

dasl.lumi.CRT.VSN <- lumiN(dasl.lumi.CRT, method='vsn')

write.exprs(dasl.lumi.CRT.VSN,"dasl.CRT.VSN.expr.txt")

x=read.table("dasl.CRT.VSN.expr.txt",header=TRUE)

y=as.matrix(cor(x))

mean(y)

#0.808575

x=read.table("dasl.VSN.expr.txt",header=TRUE)

y=as.matrix(cor(x))

mean(y)

#0.8085658

\# VST & LOESS combination yield the highest IAC for the DASL assay (0.851167)

q()

cp sample_information.txt genewise/dasl.sample.info.txt

cp sample_information.nondasl.txt genewise/nondasl.sample.info.txt

\# Added a fourth column called Covariate 1, describing tissue type (Normal or Tumor)

\# snippet of dasl.sample.info.txt file

Sample	Array	Batch	Covariate 1

OT52_N	8981245084	I	N

OT52_T	8981245084	I	T

OT5_N	8981245084	I	N

OT5_T	8981245084	I	T

OT19_N	8981245084	I	N

OT19_T	8981245084	I	T

OT1_N	8981245084	I	N

OT1_T	8981245084	I	T

OT6_T	8981245084	I	T

R

source('ComBat.R')

ComBat('dasl.VST.LOESS.expr.txt','dasl.sample.info.txt')

q()

cut -f2-29 Adjusted_dasl.VST.LOESS.expr.txt_.xls > T2

cut -f1 dasl.VST.LOESS.expr.txt > T1

paste -d"\t" T1 T2 > Adjusted_dasl.VST.LOESS.expr.txt

R

x=read.table("Adjusted_dasl.VST.LOESS.expr.txt ",header=TRUE)

y=as.matrix(cor(x))

mean(y)

#0.8646946

q()

awk '{print NR"\t"$0}' Adjusted_dasl.VST.LOESS.expr.txt | cut -f1,3-30 > Adjusted_dasl.VST.LOESS.expr.geneIDRem.txt

R

dataMatrix <- read.table("Adjusted_dasl.VST.LOESS.expr.geneIDRem.txt",header=TRUE)

sampleType <- c('N', 'N', 'N', 'N', 'N', 'N', 'N', 'N', 'N', 'N', 'N','N', 'N', 'N', 'T', 'T', 'T', 'T', 'T', 'T', 'T', 'T', 'T', 'T', 'T', 'T', 'T', 'T')

if(require(limma)) {

design <- model.matrix(~factor(sampleType))

fit <- lmFit(dataMatrix, design)

fit <- eBayes(fit)

diffExpDASL <- topTable(fit, coef='factor(sampleType)T',adjust='fdr', number=50000)

write.table(file="Differential.Expression.DASL.txt",diffExpDASL,sep="\t")

}

q()

sed 's/"//g' Differential.Expression.DASL.txt | tail -20818 | sort -n -k1 > Differential.Expression.DASL.woH.sorted.txt

tail -20818 Adjusted_dasl.VST.LOESS.expr.txt | awk '{print NR+1"\t"$1}' > id.gene.mapping

join -1 1 -2 1 id.gene.mapping Differential.Expression.DASL.woH.sorted.txt > Differential.Expression.DASL.woH.sorted.gene.txt

sed 's/ /\t/g' Differential.Expression.DASL.woH.sorted.gene.txt | cut -f 2-8 > T

mv T Differential.Expression.DASL.woH.sorted.gene.txt

*9. Pathway analyses*

*a. Method choice for pathway analyses*

\# For our pathway analysis, we used a topology based approach with the 32 short-listed genes. Graphite web's browser (http://graphiteweb.bio.unipd.it/browse.html) was used to annotate the affected genes per sample. Due to redundancy in the HUGO symbol of most genes, we use entrez ID for submission to graphite web. It came to our attention that genes with multiple alias like KMT2B (MLL4) was not annotated by graphite web when HUGO symbol was used. Both KEGG and Reactome databases were selected from the options provided by the tool.

*b. Processing of the results.*

The results were downloaded as zip files, one per sample.

1. For unzipping the archives:

Sample=($(cat $1)) #file list of sample name with provided command line while running the script; In our analysis each folder with the respective sample_name.graphite.web was created which in turn would have KEGG (has KEGG results) and Reactome (has Reactome results) directories in each.

for i in ${Sample[@]}

do

cd $i*/KEGG

unzip *.zip

cd ../

cd Reactome

unzip *.zip

cd ../../

echo $i

done

2.

i. Get.all.the.pathway.and.genes.for.G.W.sh

#Read the file from sumary and get the serial no. for each pathway

if [ $# -lt 3 ]

then

printf "\n\t\t\tUsage:\n\t\t\t\t\t\t\tbash Get.all.the.pathway.and.genes.for.G.W.sh <Summary file> <path to results file>\n> <Database.used>"

exit 0

fi

serial_no=($(cut -f1 $1 | sed '1d' | awk '{if(length($1)<2){print "00"$1} else if(length($1)<3){print "0"$1}else {print $1}}')) # this is done because serial number in the summary file and the individual resultant file differ when it comes to single and double digits.

IFS="|" pathway_List=($(cut -f2 $1 | sed '1d' |tr "\n" "|"))

for ((l=0; l<${#serial_no[@]}; l++))

do

printf "${pathway_List[$l]}\t$(cat $2/${serial_no[$l]}*AnalyzedGenes* | cut -f2 | sed '1d' | tr '\n' '\t')\n" >> Genes.per.pathway.$3.`date +"%m_%d_%y"`.tsv

done

ii. Convert.ID.sh

while read line ; do echo $line | sed 's/ /\t/g' > tmp.file; printf "$(cut -f1 tmp.file| grep -wf - $2 | cut -f2)\t$(cut -f2 tmp.file)\t$(cut -f3 tmp.file| grep -wf - $2 | cut -f2)\n"; done< <(awk -F"\t" '{sub(/ /,"_",$2);print}' $1)

iii. Find.Gene.Network.sh

\# $1= file containing the mutation/indel genes

\# $2= ALL_sif_Hugo file

grep -w $1 $2> tmp.file.1.$1

Check=1

i=1

j=1

echo $1 > remove

until [ $Check -lt 1 ]

do

j=` expr $j + 1 `

cut -f1,3 tmp.file.$i.$1 | tr '\t' '\n' |sort -u | awk 'FNR==NR{a[$1]=1;next}{if(a[$1] || a[$3]){print}}' - $2 |awk 'FNR==NR{a[$1]=1;next}{if(!a[$1] && !a[$3]){print}}' remove - > tmp.file.$j.$1

cat remove <(cut -f1 tmp.file.$j.$1) | sort -u > tmp; mv tmp remove

Check=$(cat tmp.file.$j.$1 | wc -l)

i=` expr $i + 1 `

done

cat tmp.file.*.$1 | sort -u> $1.sif

rm tmp.file.*.$1 remove

iv. run.sh

Sample=($(cat $1)) #file with sample list

for i in ${Sample[@]}

do

###########Process the Graphite web results to a single tsv file######################

cd $i\.*

#Get the path for KEGG and Reactome directories

KEGG_PATH=$(find . | grep 'KEGG/' | cut -d"/" -f2,3 | sort -u | grep -v zip)

Reactome_PATH=$(find . | grep 'Reactome/' | cut -d"/" -f2,3 | sort -u | grep -v zip)

#Compile the results into individual KEGG and Reactome .tsv file from Graphite web results.

bash ../Get.all.the.pathway.and.genes.for.G.W.sh $KEGG_PATH/summary.tsv $KEGG_PATH $i\.KEGG.All.Gene

bash ../Get.all.the.pathway.and.genes.for.G.W.sh $Reactome_PATH/summary.tsv $Reactome_PATH $i\.Reactome.All.Gene

#combine the individual KEGG and Reactome file into one

cat Genes.per.pathway.* > $i\.Genes.per.pathway.KEGG.Reactome.`date +"%m_%d_%y"`.tsv

#########Get all genes form the result with both hugo and entrez ID##################

cat $KEGG_PATH/*Genes.tsv $Reactome_PATH/*Genes.tsv | cut -f1,2 | grep -v entrez | sort -u > $i\.entrez_Hugo_ID_all_genes.tsv

######################Get All .sif ###################

cat $KEGG_PATH/*sif $Reactome_PATH/*sif | sort -u > $i\.All_Sif_in_entrez.txt

###############Convert the entrez ID to Hugo in the combined sif###############

bash ../Convert.ID.sh $i\.All_Sif_in_entrez.txt $i\.entrez_Hugo_ID_all_genes.tsv > $i\.All_sif_in_HUGO.txt

#################Get the connections of the Genes with Mutations/Indel#########

bash ../Find.Gene.Network.sh $i\.Mutation.genes.txt $i\.All_sif_in_HUGO.txt

cd ../

done

*c. Fetch pathways affected in majority of samples*

1. Combine all the pathway files across samples

cat *.Graphite.web/*.Genes.per.pathway.KEGG.Reactome.*| cut -f1| sort -u | sed 's/\'/\\\'/g'

> combinedPathways

2.

\# $1 combinedPathways

\# $2 Sample LIst

IFS="|" Pathway=($( cat $1 | tr '\n' '|'))

Sample=($(cat $2 | tr '\n' '|'))

printf "\t"

for s in ${Sample[@]}

do

printf "$s\t"

done

printf "100\n"

for p in ${Pathway[@]}

do

N=0

printf "$p\t"

grep -w "$p" *.Graphite.web/*.Genes.per.pathway.KEGG.Reactome.08_06_14.tsv | cut -d"." -f1| sort -u > tmp

for s in ${Sample[@]}

do

check=$(grep -cw "$s" tmp)

if [[ $check -eq 1 ]]

then

cd $s\.Graphite.web

check2=$(grep -w "$p" *.per.pathway.KEGG.Reactome.08_06_14.tsv | awk '{print substr($0,index($0,$2))}' | tr '\t' '\n' | sort -u | awk '$1!=""'| grep -wcf - $s\.Mutation.genes.txt)

if [[ $check2 -gt 0 ]]

then

printf "P\t"

N=` expr $N + $check`

else

printf "A\t"

fi

cd ../

else

printf "A\t"

fi

done

printf "$N\n"

done

Results from running the above script can be directed to a .csv/.tsv file. we can get the frequency with the highest frequency by sorting the last column which has the sample count for the affected pathway across sample.

*d. build pathways*

Cytoscape was used to build the pathway. To find connectivity of the a gene to others, the file Mutation/indel.affected.gene.sif (say TP53.sif) resulting from running run.sh was used. This file contains all the list of genes connected to the gene in question.

E.g.

If I want to know if CASP8 is connected to TP53.

I would grep -w CASP8 from the TP53.sif;

You would find CASP8 but its not connected directly to TP53 but is indirectly, via a series of other genes.

*10. Estimating frequencies of nucleotide changes and Ti/Tv ratios*

a.

Location:

/common/Data/projects/internal/OSCC/GATK/exomeFilteredSNPs/

Concat all the three bait-filtered files :

perl -e 'open(FH,"SampleDetails"); foreach $a(<FH>){chomp $a; print "cat 33MB/PASS/$a\_*.PASS.vcf TS62MB_unique/PASS/$a\_*.PASS.vcf SS50MB_unique/PASS/$a\_*.PASS.vcf > TiTV/$a.33.55.62.vcf\n"}' > ConCAt.sh

SampleDetails contains sample name with their respective types. For e.g. OT1	T

b. bash ConCAt.sh

c. Mutation.frequencies.sh:

#! /usr/bin/bash

sample=$(cat $1 | grep -w $2 | awk '{print $1$2$3$4 }' | tr "\n" " ")

echo $sample

Sample=($sample)

printf "\t"

printf "%s\t%s\t%s\t%s\t%s\t%s\t%s\t%s\t%s\t%s\t%s\t%s\t%s\t%s\t%s\t\t" G_C_transversion G_T_transversion G_A_transition A_C_transversion A_T_transversion A_G_transition T_A_transversion T_G_transversion T_C_transition C_A_transversion C_G_transversion C_T_transition transversionTotal transitionToTal Ratio

printf "%s\t%s\t%s\t%s\t%s\t%s\t%s\t%s\t%s\t%s\t%s\t%s\n" percentage_GC percentage_GT percentage_GA percentage_AC percentage_AT percentage_AG percentage_TA percentage_TG percentage_TC percentage_CA percentage_CG percentage_CT

printf "\n"

for((j=0; j <${#Sample[@]}; j++ ))

do

cat ${Sample[$j]}.33.55.62.vcf > temp

ALL=$(cat temp | wc -l)

printf "%s\t" ${Sample[$j]}

G_C_transversion=$(awk '{if($4 ~/G/ && $5 ~/C/){count++; print count}}' temp| tail -1)

G_T_transversion=$(awk '{if($4 ~/G/ && $5 ~/T/){count++; print count}}' temp| tail -1)

G_A_transition=$(awk '{if($4 ~/G/ && $5 ~/A/){count++; print count}}' temp| tail -1)

A_C_transversion=$(awk '{if($4 ~/A/ && $5 ~/C/){count++; print count}}' temp| tail -1)

A_T_transversion=$(awk '{if($4 ~/A/ && $5 ~/T/){count++; print count}}' temp| tail -1)

A_G_transition=$(awk '{if($4 ~/A/ && $5 ~/G/){count++; print count}}' temp| tail -1)

T_A_transversion=$(awk '{if($4 ~/T/ && $5 ~/A/){count++; print count}}' temp| tail -1)

T_G_transversion=$(awk '{if($4 ~/T/ && $5 ~/G/){count++; print count}}' temp| tail -1)

T_C_transition=$(awk '{if($4 ~/T/ && $5 ~/C/){count++; print count}}' temp| tail -1)

C_A_transversion=$(awk '{if($4 ~/C/ && $5 ~/A/){count++; print count}}' temp| tail -1)

C_G_transversion=$(awk '{if($4 ~/C/ && $5 ~/G/){count++; print count}}' temp| tail -1)

C_T_transition=$(awk '{if($4 ~/C/ && $5 ~/T/){count++; print count}}' temp| tail -1)

percentage_GC=$(echo "($G_C_transversion/$ALL)*100" | bc -l)

percentage_GT=$(echo "($G_T_transversion/$ALL)*100" | bc -l)

percentage_GA=$(echo "($G_A_transition/$ALL)*100" | bc -l)

percentage_AC=$(echo "($A_C_transversion/$ALL)*100" | bc -l)

percentage_AT=$(echo "($A_T_transversion/$ALL)*100" | bc -l)

percentage_AG=$(echo "($A_G_transition/$ALL)*100" | bc -l)

percentage_TA=$(echo "($T_A_transversion/$ALL)*100" | bc -l)

percentage_TG=$(echo "($T_G_transversion/$ALL)*100" | bc -l)

percentage_TC=$(echo "($T_C_transition/$ALL)*100" | bc -l)

percentage_CA=$(echo "($C_A_transversion/$ALL)*100" | bc -l)

percentage_CG=$(echo "($C_G_transversion/$ALL)*100" | bc -l)

percentage_CT=$(echo "($C_T_transition/$ALL)*100" | bc -l)

transversion_total=$( echo "($G_C_transversion + $G_T_transversion + $A_C_transversion + $A_T_transversion + $T_A_transversion + $T_G_transversion + $C_A_transversion + $C_G_transversion)" | bc)

transition_total=$(echo "($G_A_transition + $A_G_transition + $T_C_transition + $C_T_transition)" | bc)

Ratio=$(echo "$transition_total / $transversion_total" | bc -l)

printf "%i\t%i\t%i\t%i\t%i\t%i\t%i\t%i\t%i\t%i\t%i\t%i\t%i\t%i\t%f\t\t" $G_C_transversion $G_T_transversion $G_A_transition $A_C_transversion $A_T_transversion $A_G_transition $T_A_transversion $T_G_transversion $T_C_transition $C_A_transversion $C_G_transversion $C_T_transition $transversion_total $transition_total $Ratio

printf "%f\t%f\t%f\t%f\t%f\t%f\t%f\t%f\t%f\t%f\t%f\t%f\n" $percentage_GC $percentage_GT $percentage_GA $percentage_AC $percentage_AT $percentage_AG $percentage_TA $percentage_TG $percentage_TC $percentage_CA $percentage_CG $percentage_CT

done

rm temp

d. bash Mutation_frequencies.sh SampleDetails

e. The ti/tv ratios are estimated for each sample as a ratio of sum of all transition type mutations to sum of all transversion type mutations, and the frequencies of individual nucleotide change types are also reported.

*11. Effect of epidemiology on nucleotide change frequencies*

df<-read.table("changes.forboxplot",sep="\t",header=TRUE,na.strings="NA")

df<-na.omit(df)

library(plyr)

library(ggplot2)

plot_Data <-ddply(df, .(HPV, MutationType), mutate,med=median(Frequency),min=min(Frequency),max=max(Frequency),Q1=quantile(Frequency, 1/4),Q3=quantile(Frequency, 3/4), IQR=Q3-Q1, upper.limit=Q3+1.5*IQR, lower.limit=Q1-1.5*IQR)

p <-ggplot(data = plot_Data, aes(x = HPV))

p <-p + geom_boxplot(aes(lower = Q1, upper = Q3, middle = med, ymin = min, ymax = max), stat= "identity", colour="blue", outlier.colour = "black", outlier.shape = 16, outlier.size = 16)

p <-p +geom_point(data=plot_Data[plot_Data$Frequency > plot_Data$upper.limit |plot_Data$Frequency < plot_Data$lower.limit,], aes(y=Frequency))

p <-p + facet_grid(~ MutationType, scales="free", space="free")

p <-p + theme(plot.title = element_text("Frequency"),axis.text.x = element_text(angle = 90, hjust = 1, size = 8, colour = "grey50"),plot.title = element_text(face="bold", size=11),axis.title.x = element_text(face="bold", size=9),axis.title.y = element_text(face="bold", size=9, angle=90),panel.grid.major = element_blank(),panel.grid.minor = element_blank())

p <-p + scale_fill_hue(c=45, l=80)

p<- p + coord_cartesian(ylim=c(0,100))

ggsave("HPV.Frequency.png",plot=p,dpi=600)

Snippet of changes.forboxplot file:

Samples	Age	Gender	Smoking	Alcohol	Chewing	HPV	Survival	Grade	Node	Recurrence	Stage	MutationType	Frequency

OT51	A50	F	N	N	Y	Y	D	WD	N	M	T1	A_C	3.16

OT1	B50	F	N	N	N	N	A	WD	N	N	T1	A_C	3.56

OT14	B50	M	N	Y	Y	N	A	MD	N	N	T1	A_C	3.69

OT30	A50	M	Y	N	N	Y	A	MD	N	N	T2	A_C	3.46

OT54	B50	F	NA	NA	N	Y	A	MD	N	N	T2	A_C	3.57

OT20	B50	M	Y	N	N	NA	A	MD	N	Y	T2	A_C	2.74

OT2	B50	M	N	Y	Y	Y	A	MD	N	M	T3	A_C	3.27

OT18	A50	M	Y	Y	N	Y	D	MD	N	M	T3	A_C	2.92

OT3	B50	M	N	Y	N	Y	A	MD	N	N	T3	A_C	3.46

OT11	A50	M	Y	Y	N	Y	A	MD	N	N	T3	A_C	3.45

OT19	A50	M	Y	Y	Y	Y	A	WD	N	N	T3	A_C	2.93

OT10	A50	M	N	N	N	NA	A	MD	N	Y	T3	A_C	3.19

OT9	B50	M	N	Y	Y	Y	A	MD	N	N	T4	A_C	4.84

OT8	B50	M	N	N	Y	NA	D	MD	N	N	T4	A_C	3.64

OT17	B50	M	Y	Y	Y	N	D	PD	N	N	T4	A_C	3.37

OT4	B50	M	N	N	Y	N	A	MD	N	Y	T4	A_C	4.47

OT40	B50	M	Y	Y	N	Y	D	PD	N	Y	T4	A_C	3.85

OT34	B50	M	N	N	Y	Y	D	PD	N	Y	T4	A_C	3.21

OT41	B50	F	N	N	N	NA	A	WD	P	N	T1	A_C	3.05

12. Scripts for running random forest analyses to predict tumor recurrence

*1. R script (Recurrence.Genes.RF.R)*

library(varSelRF)

DS=read.table("Recurrence_genes_matrix_for_RF.tsv",header=TRUE,na.strings="NA",sep="\t")

DS<-na.omit(DS)

for (i in 1:500) {

set.seed(i)

DS.rf.vsf=varSelRF(DS[,-1],DS[,1],ntree=3000,ntreeIterat=2000,vars.drop.frac=0.2,whole.range = FALSE,keep.forest = TRUE)

print(DS.rf.vsf$selected.vars)

print(predict(DS.rf.vsf$rf.model,subset(DS[,-1],select=DS.rf.vsf$selected.vars)))

}

*2. Post-processing R output*

rm recurrence

egrep '\[[0-9]*\]|M |NR |R ' Recurrence.Genes.RF.Rout | grep -v Recurrence_genes_matrix_for_RF.tsv | sed '1,5d' |grep -v Levels | sed 's/\[[0-9]*\]/[]/g' | sed 's/ *$//g' |sed 's/^ *//g' | sed -e :a -e '/[N,M,NR]$/N; s/\([N,M,NR]\)\n\([N,M,NR]\)/\1 \2/; ta'| sed -e :a -e '/\"$/N;s/\"\n\[\] \"/\" \"/; ta' | sed 's/\"//g' | sed 's/\[\] //g'|sed 's/ / /g' > Recurrence.Genes.RF.Rout.Parsed

tail -n+2 Recurrence_genes_matrix_for_RF.tsv |grep -v 'NA' | cut -f1 | tr '\n' '\t' | sed 's/\t$//g' > recurrence

awk '{for(i=0;i<500;i++) print}' survival | tr '\t' '\n' > a.txt

grep -E $' R | M | NR ' Recurrence.Genes.RF.Rout.Parsed | grep -vE '\.' | tr ' ' '\n' | awk '$1!="" && length($1)<3'> b.txt

paste -d"\." a.txt b.txt | xargs -n$(wc -w recurrence | sed 's/ .*//') | awk '{count=0;for (i=1;i<=NF;i++) {count1[i]=0;} for (i=1;i<=NF;i++) {if ($i == "R.R" || $i == "NR.NR" || $i == "M.M") {count++;count1[i]++;}} for (i=1;i<=NF;i++) {printf "%f\t",count1[i]*100;}; print count/NF*100}' > c.txt

grep -vE $' NR | R | M ' Recurrence.Genes.RF.Rout.Parsed | awk '{print NF"\t"$0}' > d.txt

paste -d"\t" c.txt d.txt | awk -F"\t" '{print NR"\t"$1"\t"$2"\t"$3"\t"$4"\t"$5"\t"$6"\t"$7"\t"$8"\t"$9"\t"$10"\t"$11"\t"$12"\t"$13"\t"$14"\t"$15"\t"$16"\t"$17"\t"$18"\t"$19"\t"$20"\t"$21"\t"$22"\t"$23"\t"$24"\t"$25"\t"$26"\t"$27"\t"$28"\t"$29"\t"$30"\t"$31"\t"$32"\t"$33"\t"$34"\t"$35"\t"$36"\t"$37"\t"$38"\t"$39"\t"$40"\t"$41"\t"$42"\t"$43"\t"$44"\t"$45"\t"$46"\t"$47"\t"$48"\t"$49"\t"$50"\t"$51"\t"$52}' > individual.Recurrence.Genes.RF.Rout.Result

*3. Snippet of Recurrence_genes_matrix_for_RF.tsv:*

Recurrence	A2ML1	AAK1	AARSD1	AASDH	AATK	AATK-AS1

NR	0	0	0	0	0	0

M	0	0	0	0	0	0

NR	0	1	0	0	0	0

R	0	0	0	0	0	0

NR	0	0	0	0	0	0

NR	0	0	0	0	0	0

NR	0	0	0	0	0	0

NR	0	0	0	0	0	0

NR	0	0	0	0	0	0

4. *0.632+ bootstrapping*:

library(varSelRF)

DS=read.table("Recurrence_genes_matrix_for_RF.tsv",header=TRUE,na.strings="NA",sep="\t")

DS<-na.omit(DS)

for (i in 1:100) {

set.seed(i)

DS.rf.vsf=varSelRF(DS[,-1],DS[,1],ntree=3000,ntreeIterat=2000,vars.drop.frac=0.2,whole.range = FALSE,keep.forest = TRUE)

DS.bootrf.vsf=varSelRFBoot(DS[,c(DS.rf.vsf$selected.vars)],DS[,1],ntree=3000,ntreeIterat=2000,vars.drop.frac=0.2,whole.range = FALSE,keep.forest = TRUE, bootnumber=50, usingCluster = FALSE)

file2=paste("Recurrence.boot.allDataRandomForest",i,sep="")

write.table(file=file2,DS.bootrf.vsf$all.data.randomForest$confusion,sep="\t",quote=FALSE)

}

5. Re-computing variable importances after FDR correction of variable importances using Benjamin-Hochberg test

library(varSelRF)

DS=read.table("Recurrence_genes_matrix_for_RF.tsv",header=TRUE,na.strings="NA",sep="\t")

DS<-na.omit(DS)

for (i in 1:500) {

set.seed(i)

DS.rf.vsf=varSelRF(DS[,-1],DS[,1],ntree=3000,ntreeIterat=2000,vars.drop.frac=0.2,whole.range = FALSE,keep.forest = TRUE)

DS.rf=randomForest(DS[,-1],DS[,1],ntree=3000,keep.forest=FALSE,importance=TRUE)

fileb=paste("Recurrence.beforeFDR",i,sep="")

filea=paste("Recurrence.afterFDR",i,sep="")

before<-DS.rf.vsf$initialImportances

after<-p.adjust(DS.rf$importance[,3], method = "BH", n = 6)

write.table(x=before,file=fileb,sep=”\t”,quote=FALSE)

write.table(x=after,file=filea,sep=”\t”,quote=FALSE)

}
