# Supplementary material for: Integrated analysis of oral tongue squamous cell carcinoma identifies key variants and pathways linked to risk habits, HPV, clinical parameters and tumor recurrence
Source: F1000Res. 2015 Nov 5;4:1215. [Version 1] doi: 10.12688/f1000research.7302.1 (PMC4706066; doi:10.12688/f1000research.7302.1)

Event: chr17 7577120

Sample: OT1

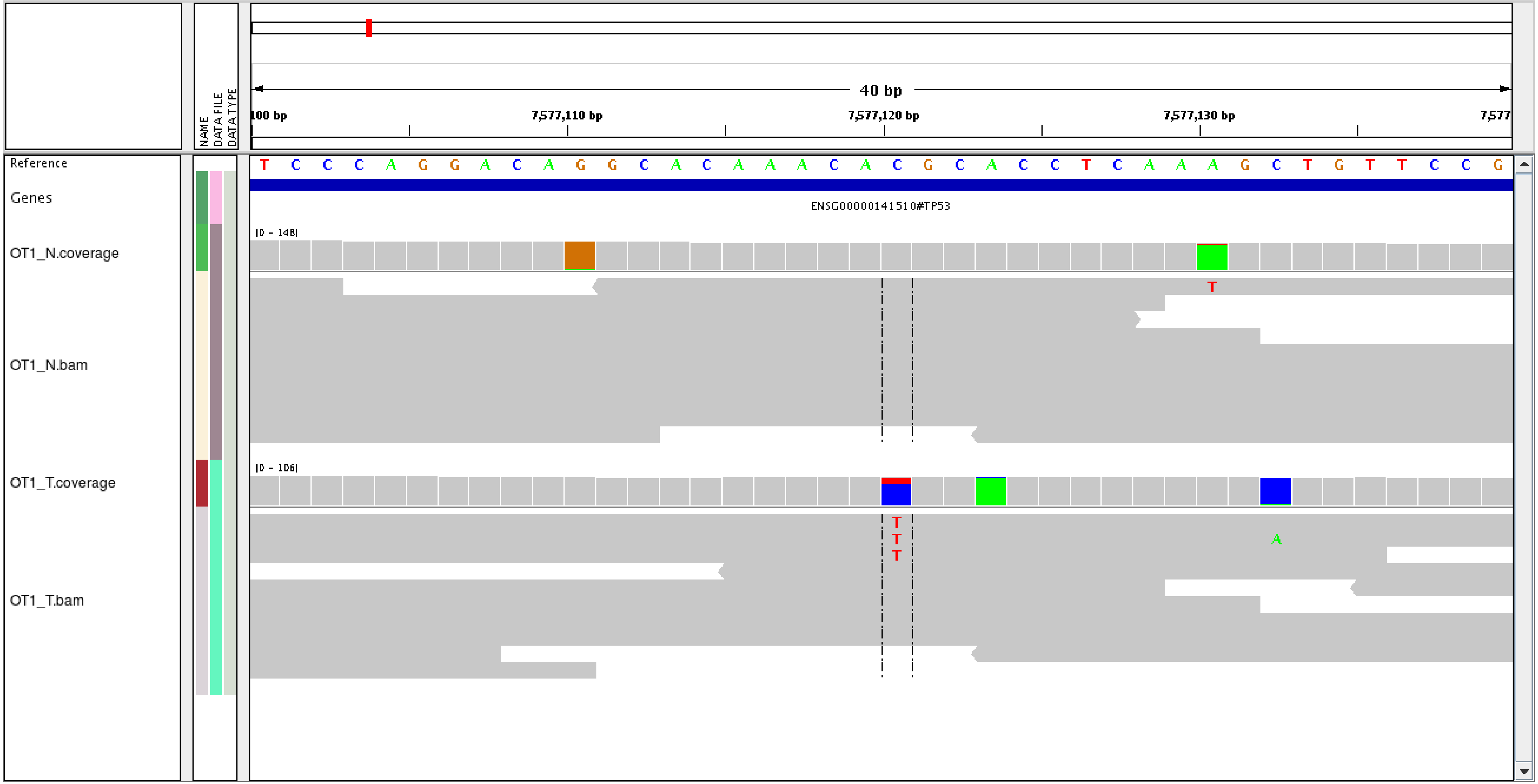

Event: chr1 228564884

Sample: OT2

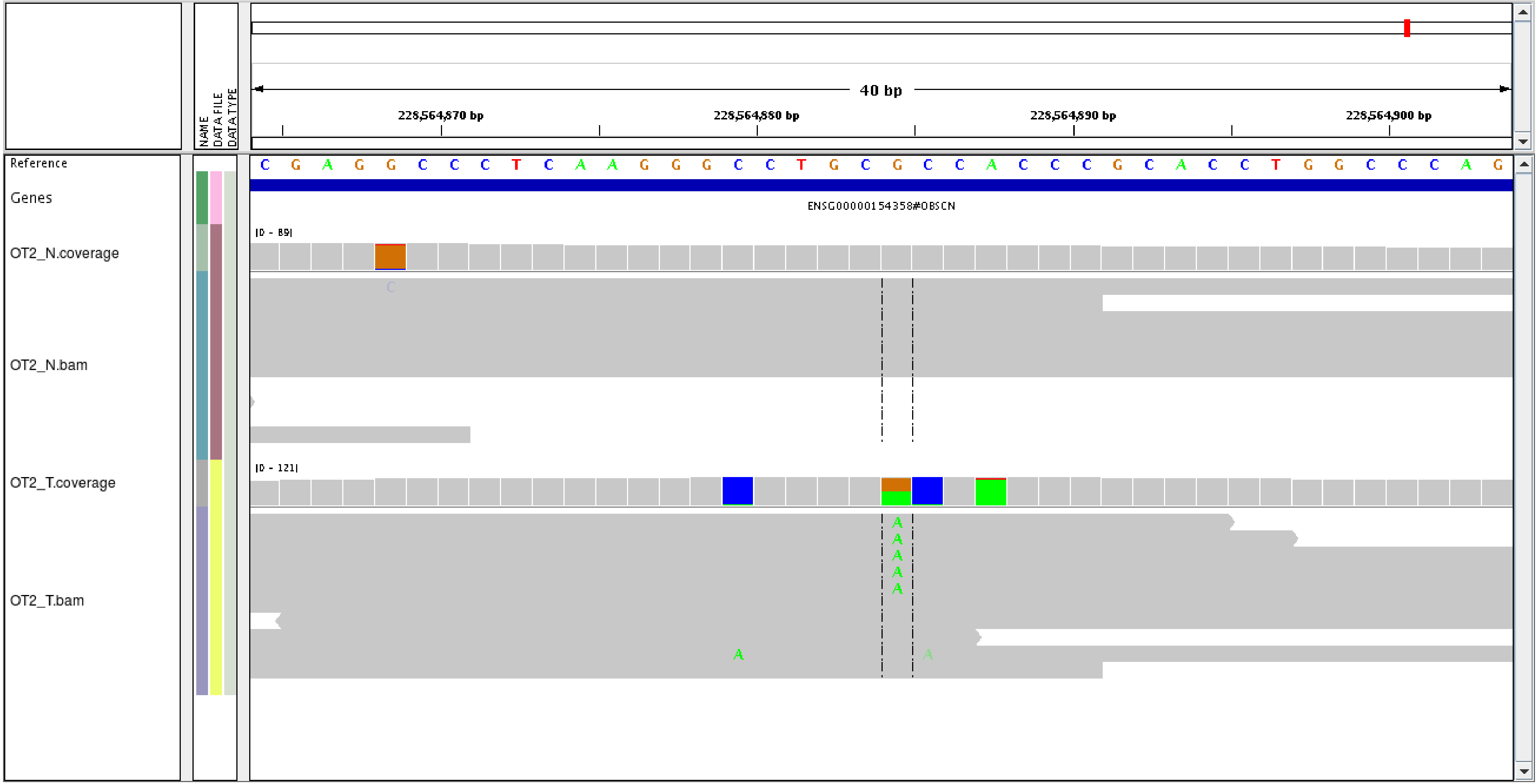

Event: chr13 25670804

Sample: OT4

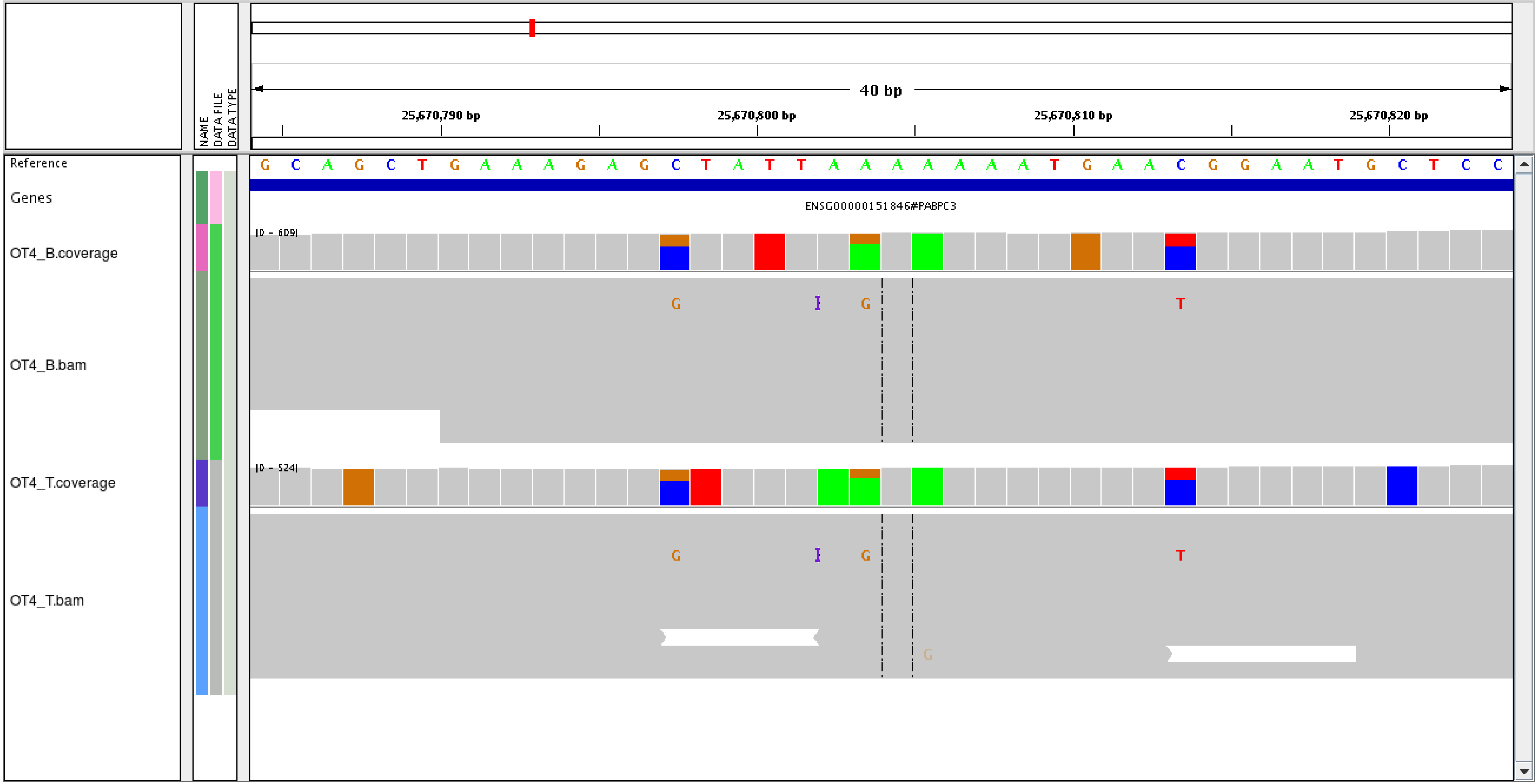

Event: chr9 139405261

Sample: OT6

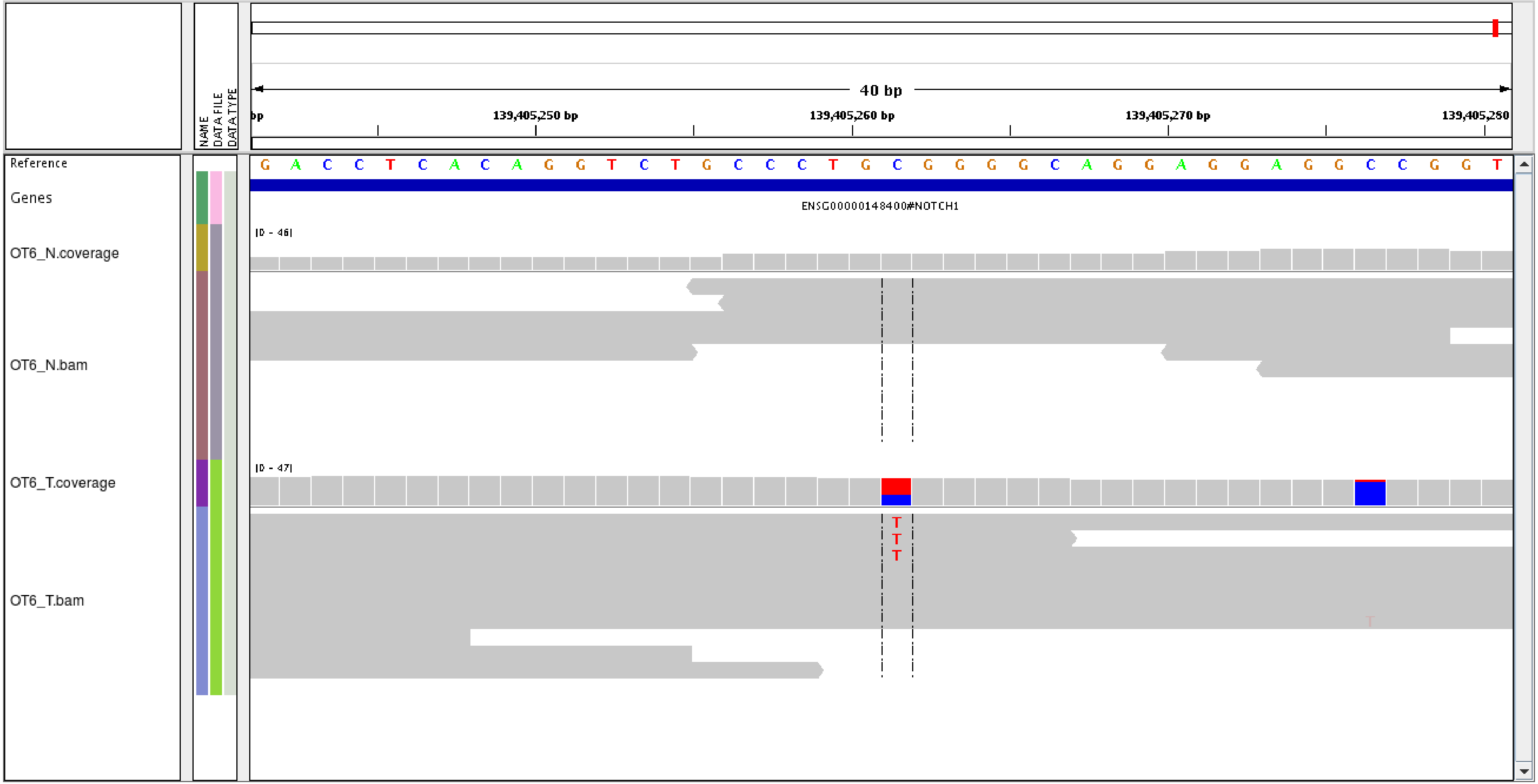

Event: chr17 7578212

Sample: OT6

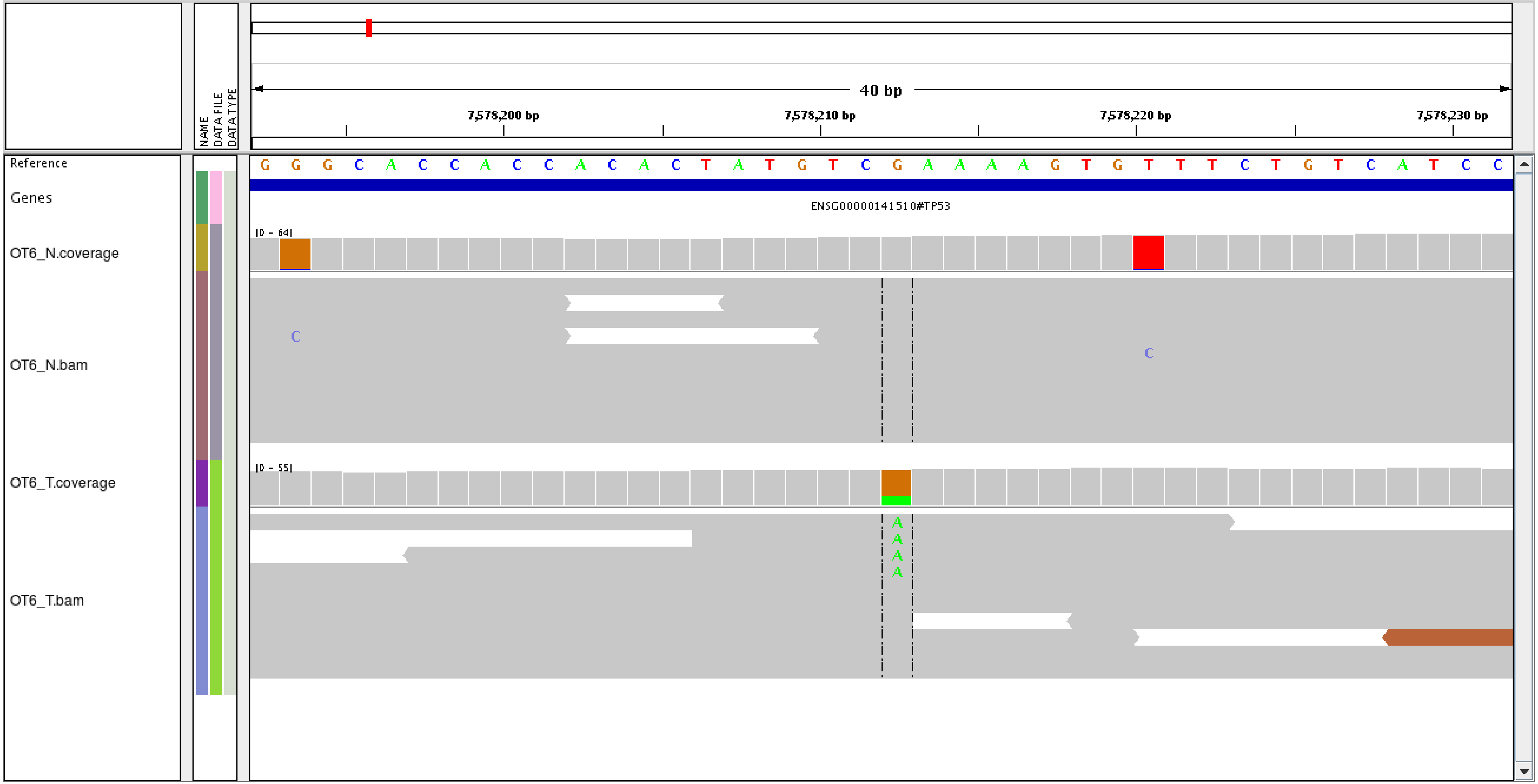

Event: chr2 202141594

Sample: OT7

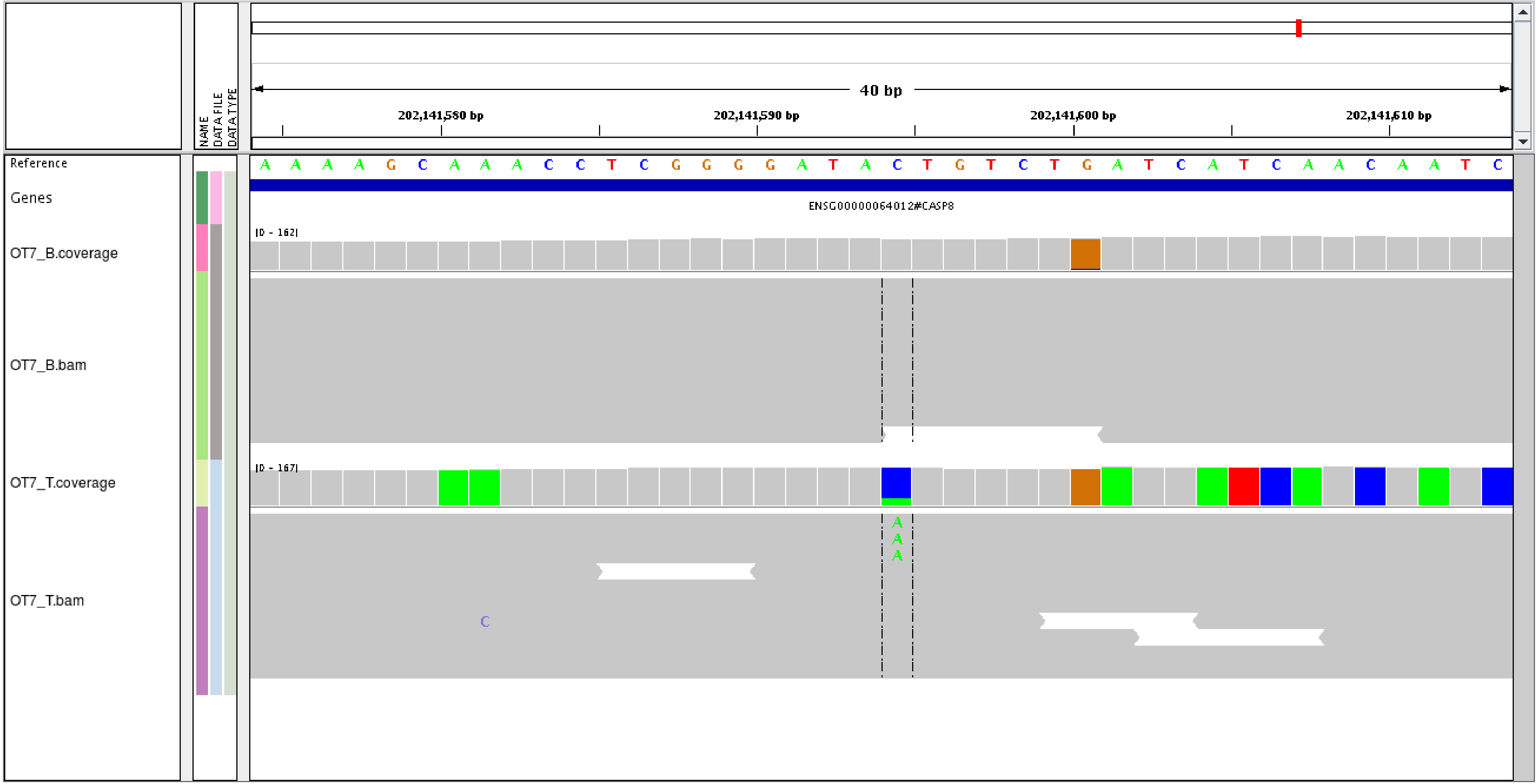

Event: chr6 29910716

Sample: OT8

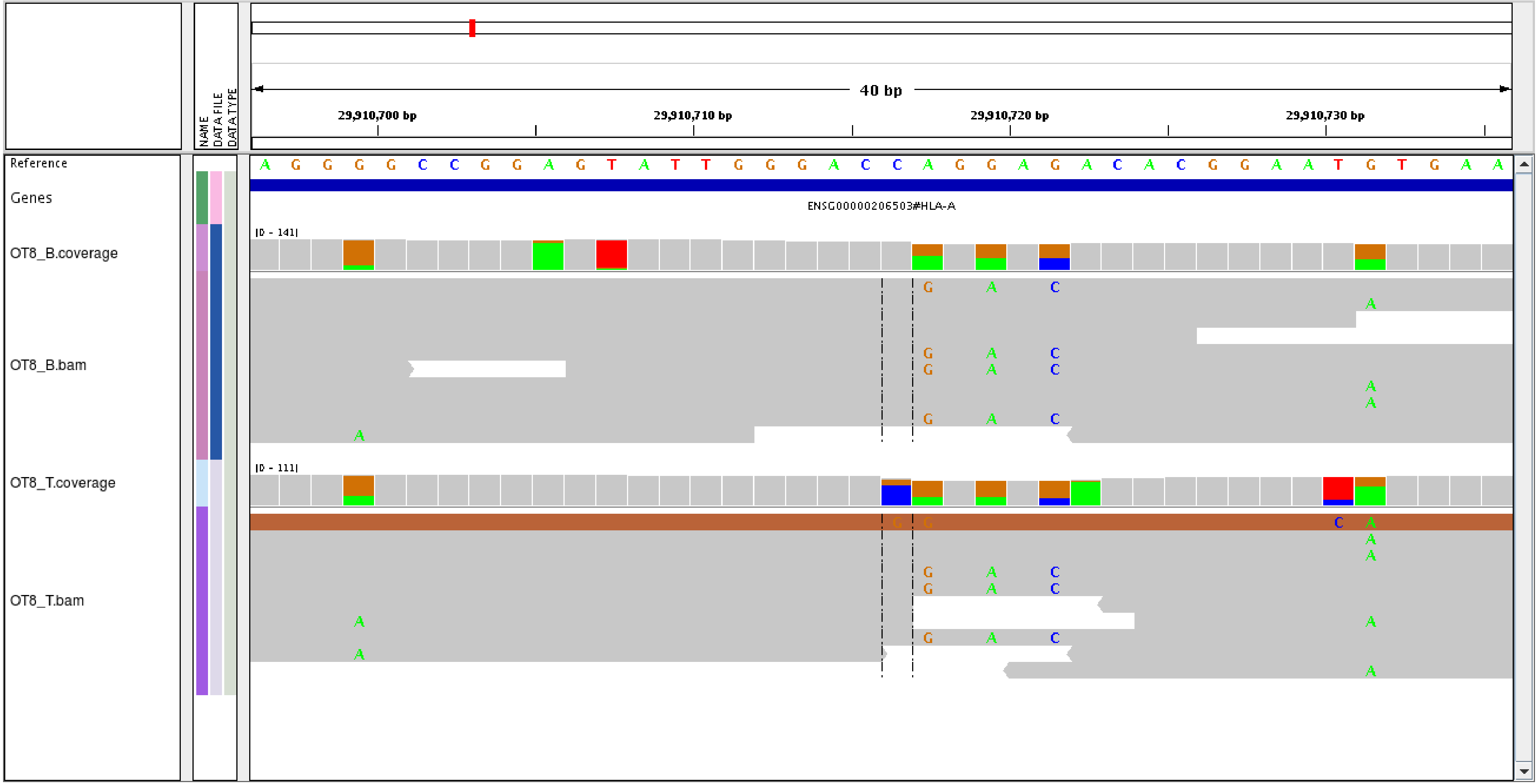

Event: chr6 29910730

Sample: OT8_B

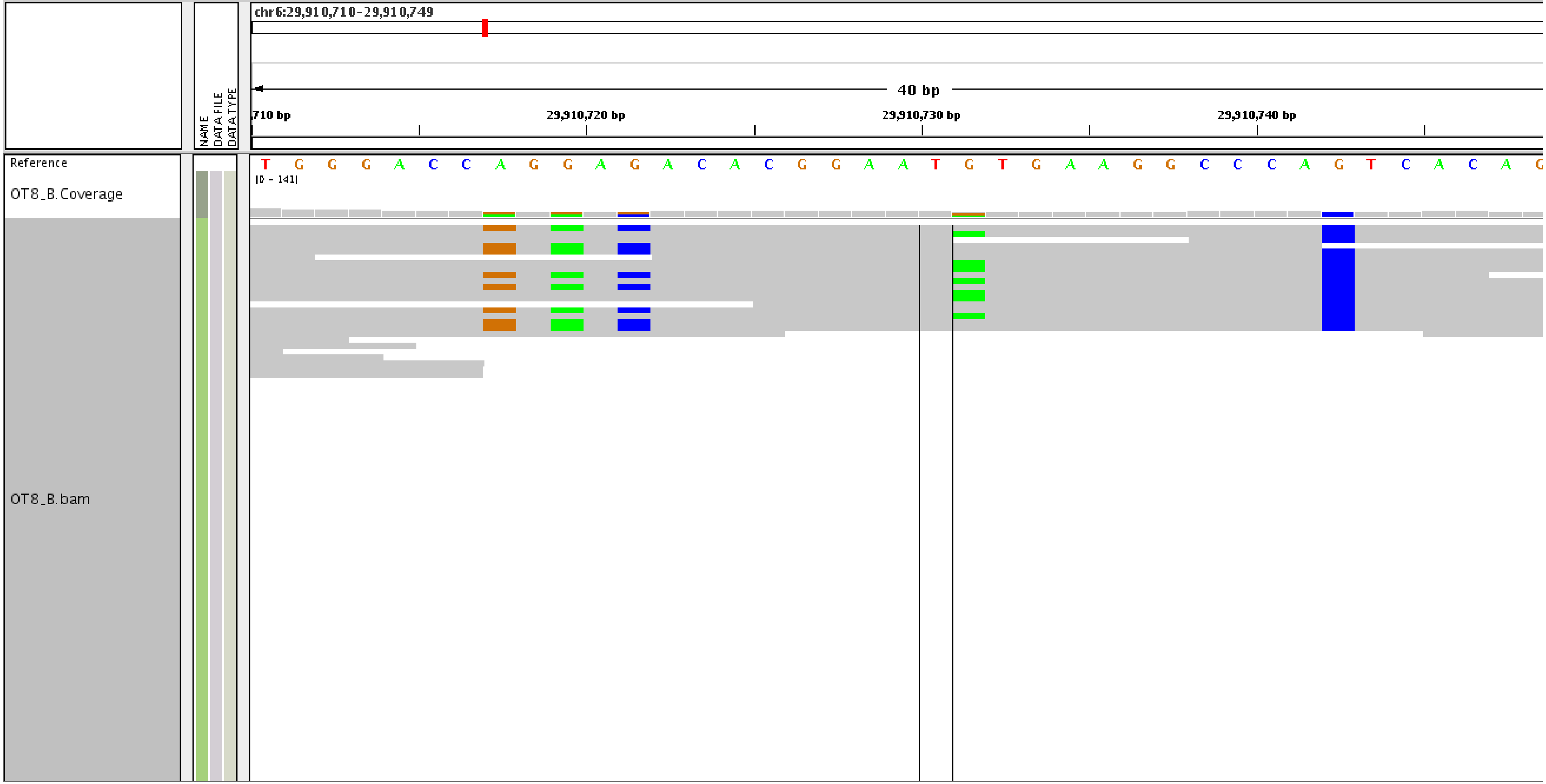

Event: chr6 29910730

Sample: OT8_T

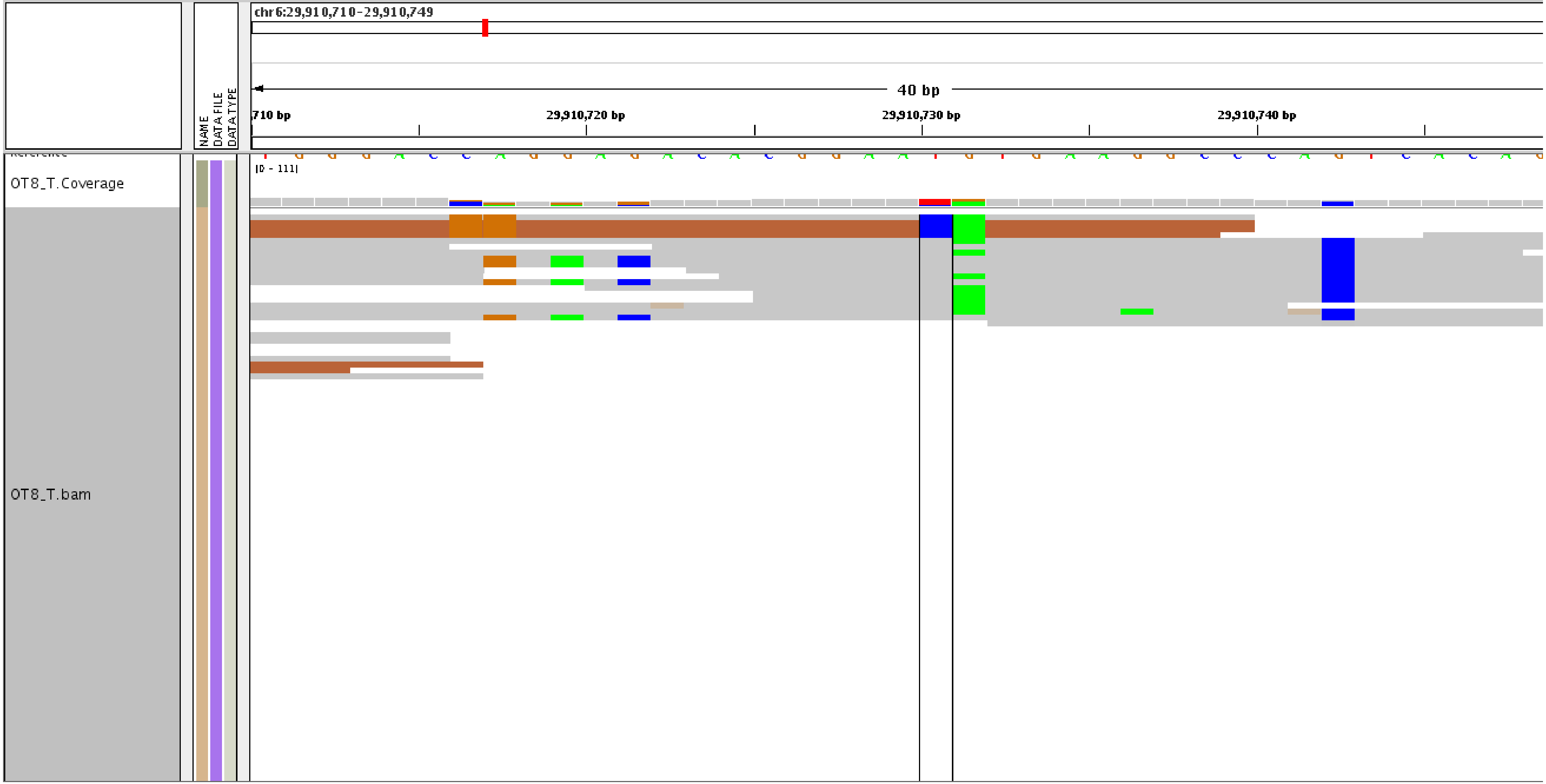

Event: chr17 7577570

Sample: OT9

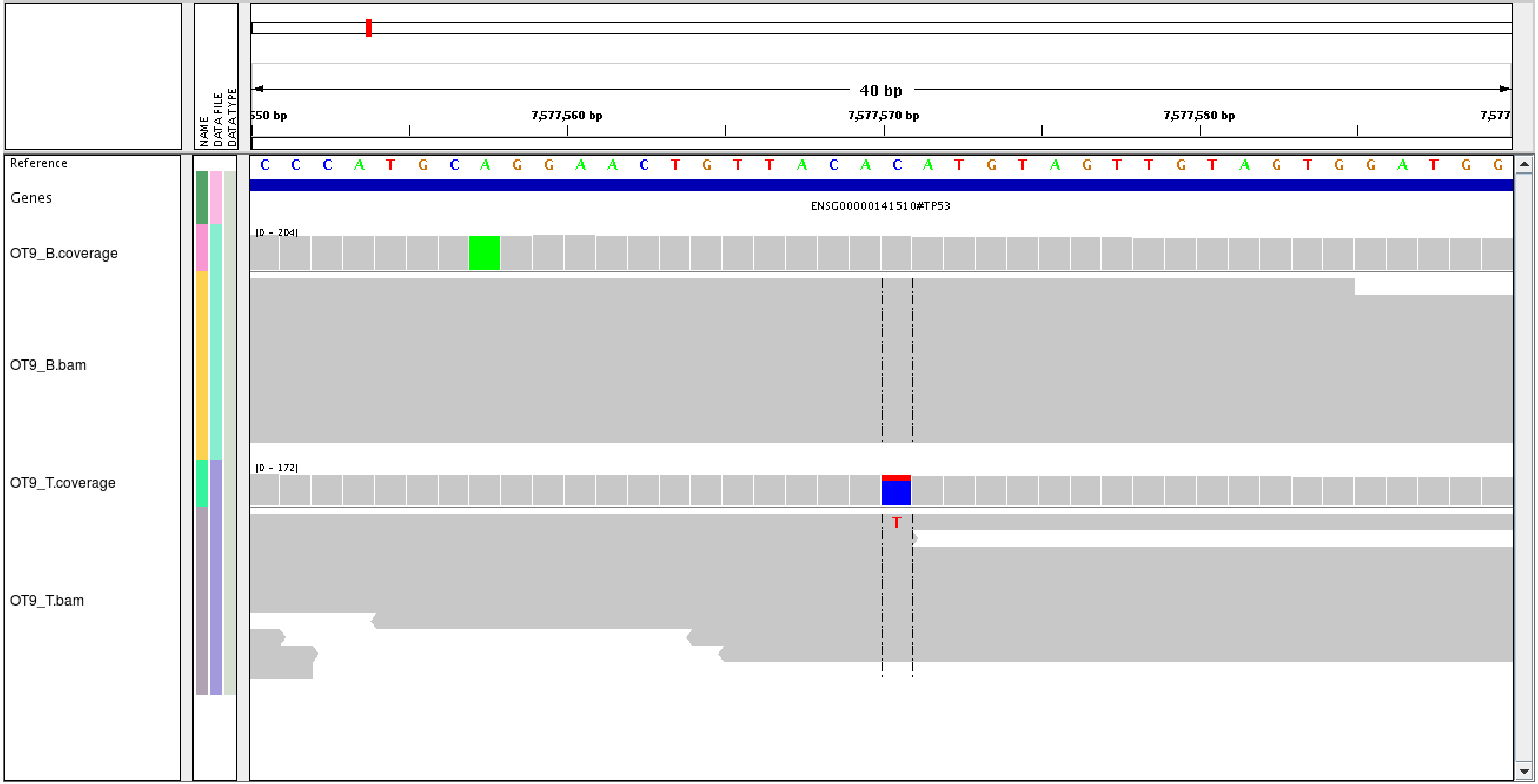

Event: chr1 120464917

Sample: OT10_B

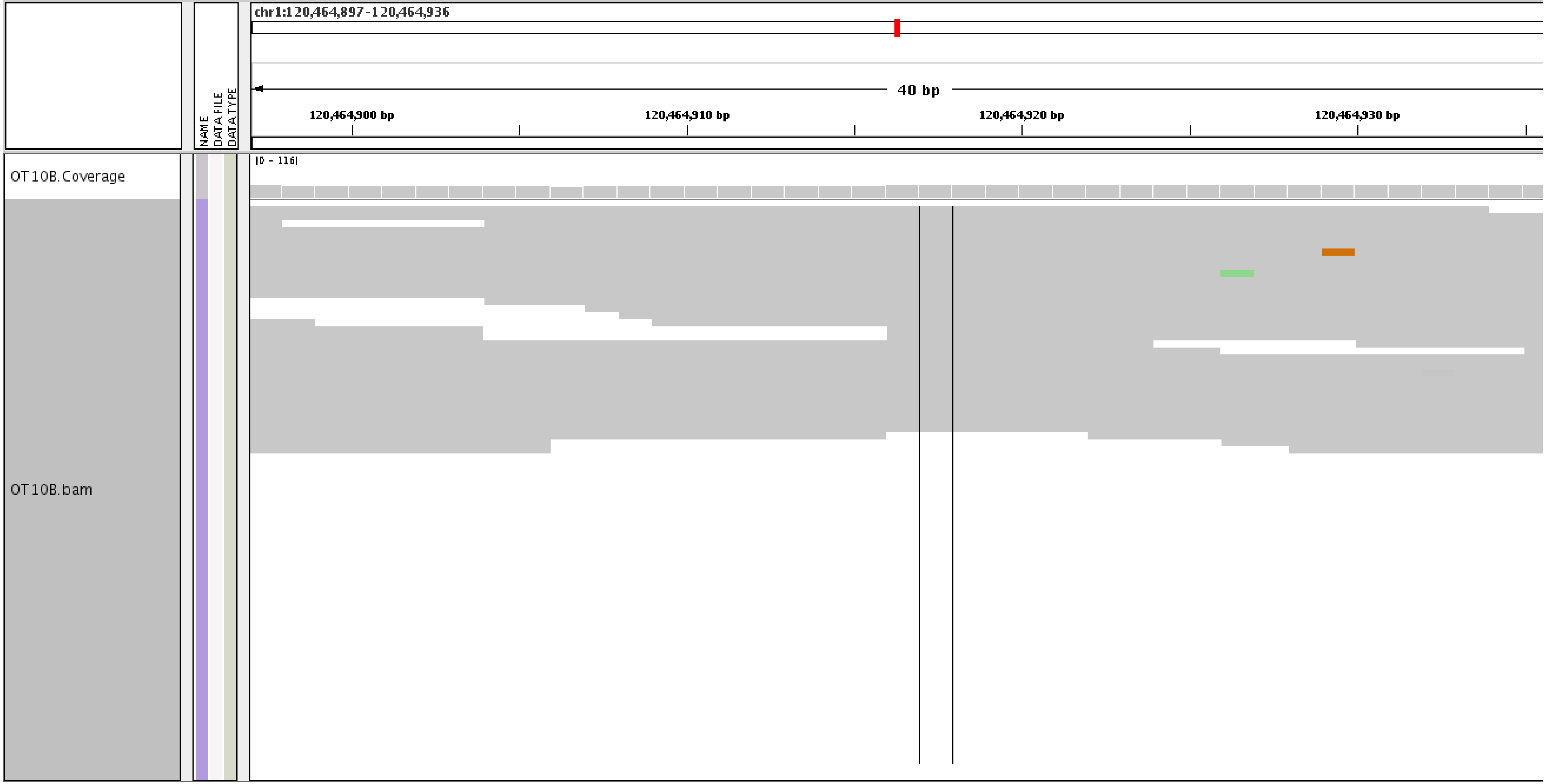

Event: chr1 120464917

Sample: OT10_T

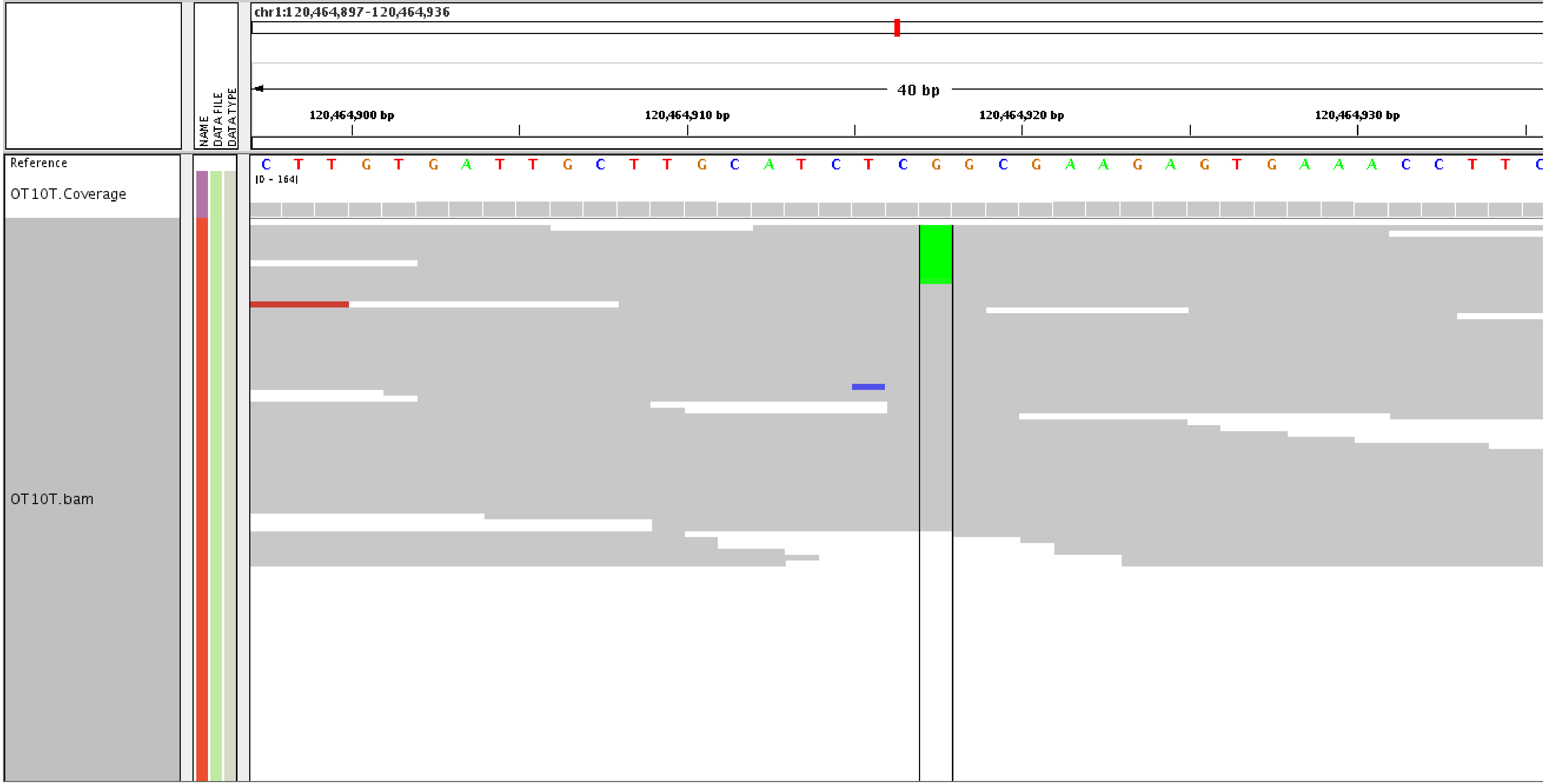

Event: chr17 7578462

Sample: OT11

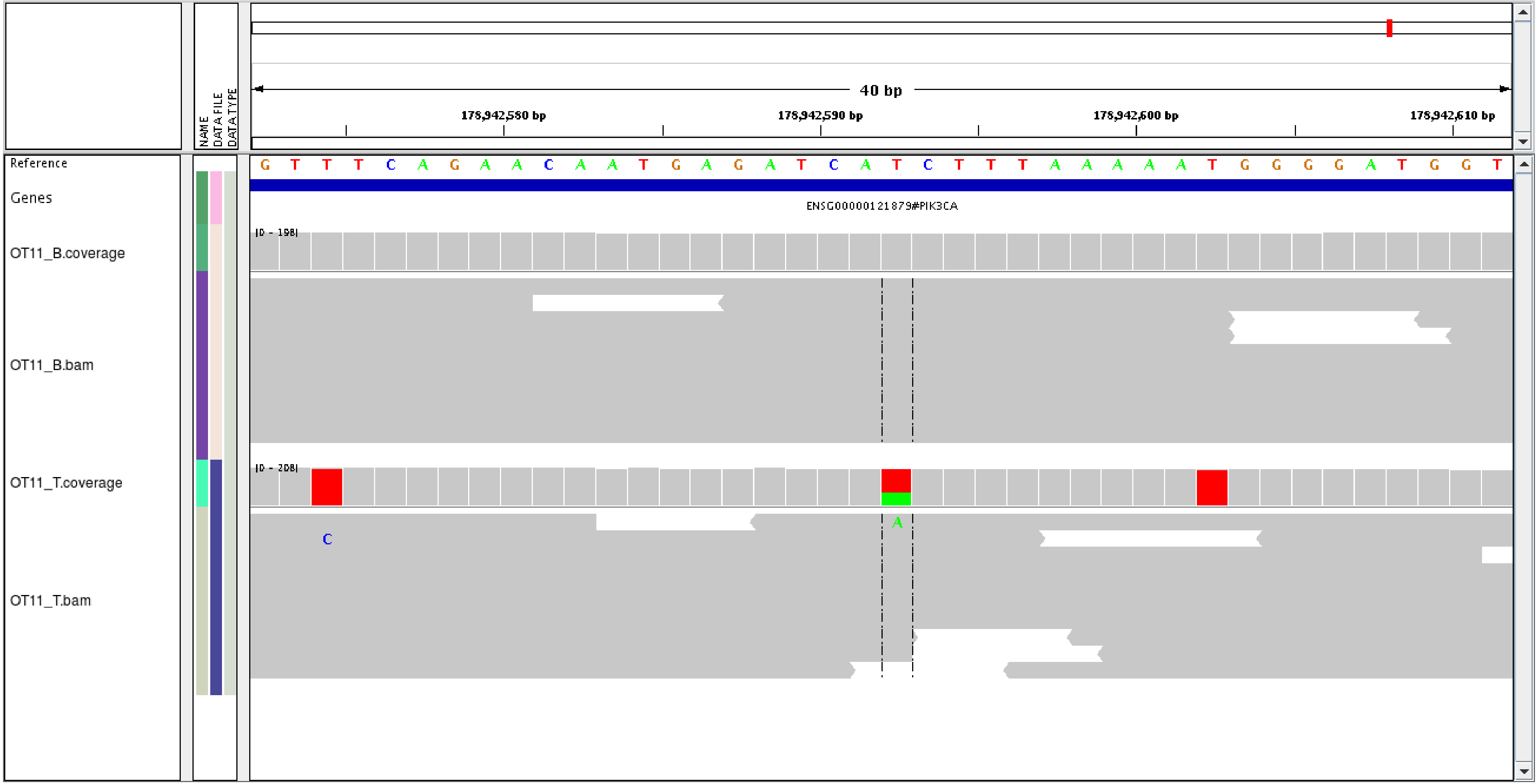

Event: chr3 178942592

Sample: OT11

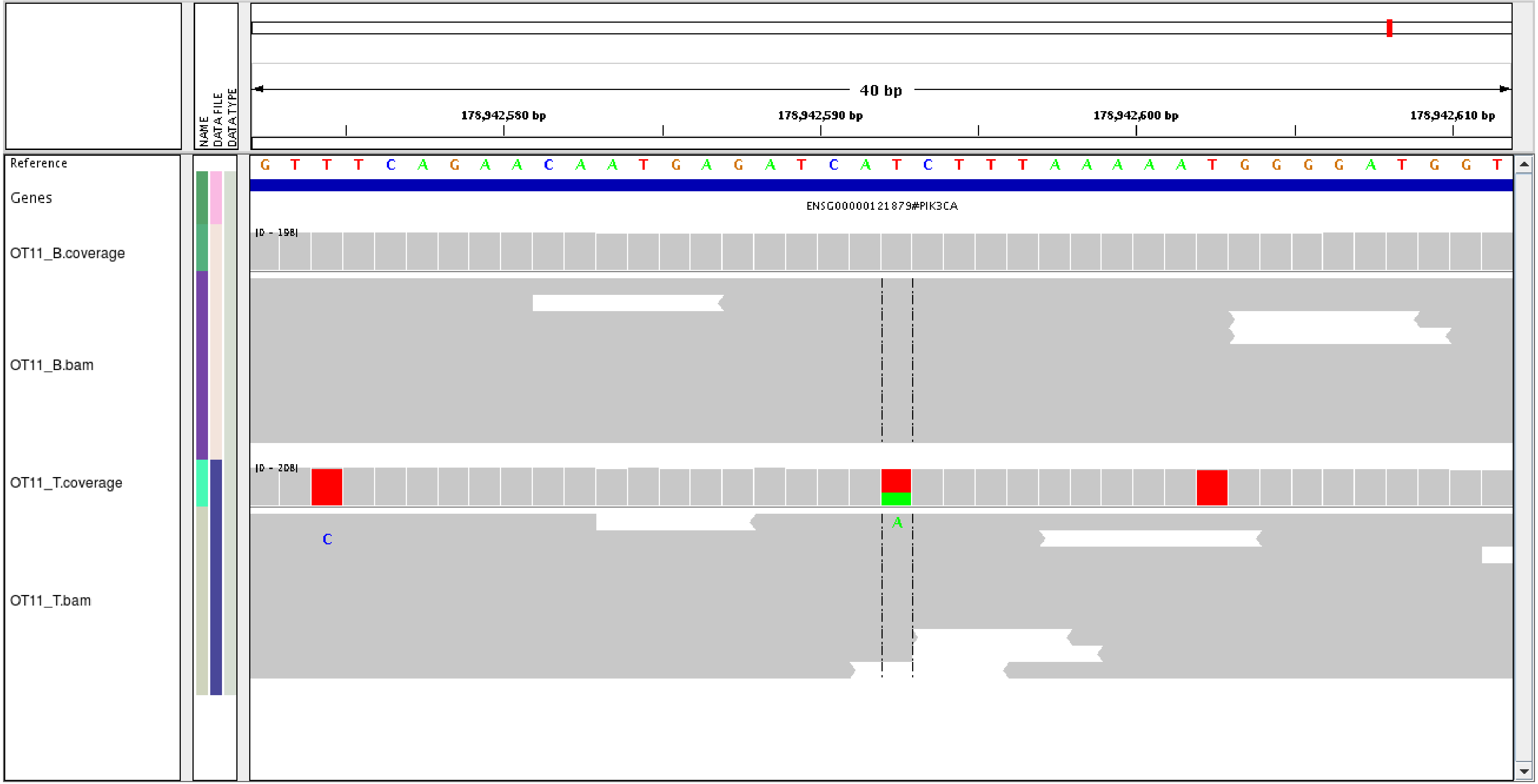

Event: chr17 7577105

Sample: OT12

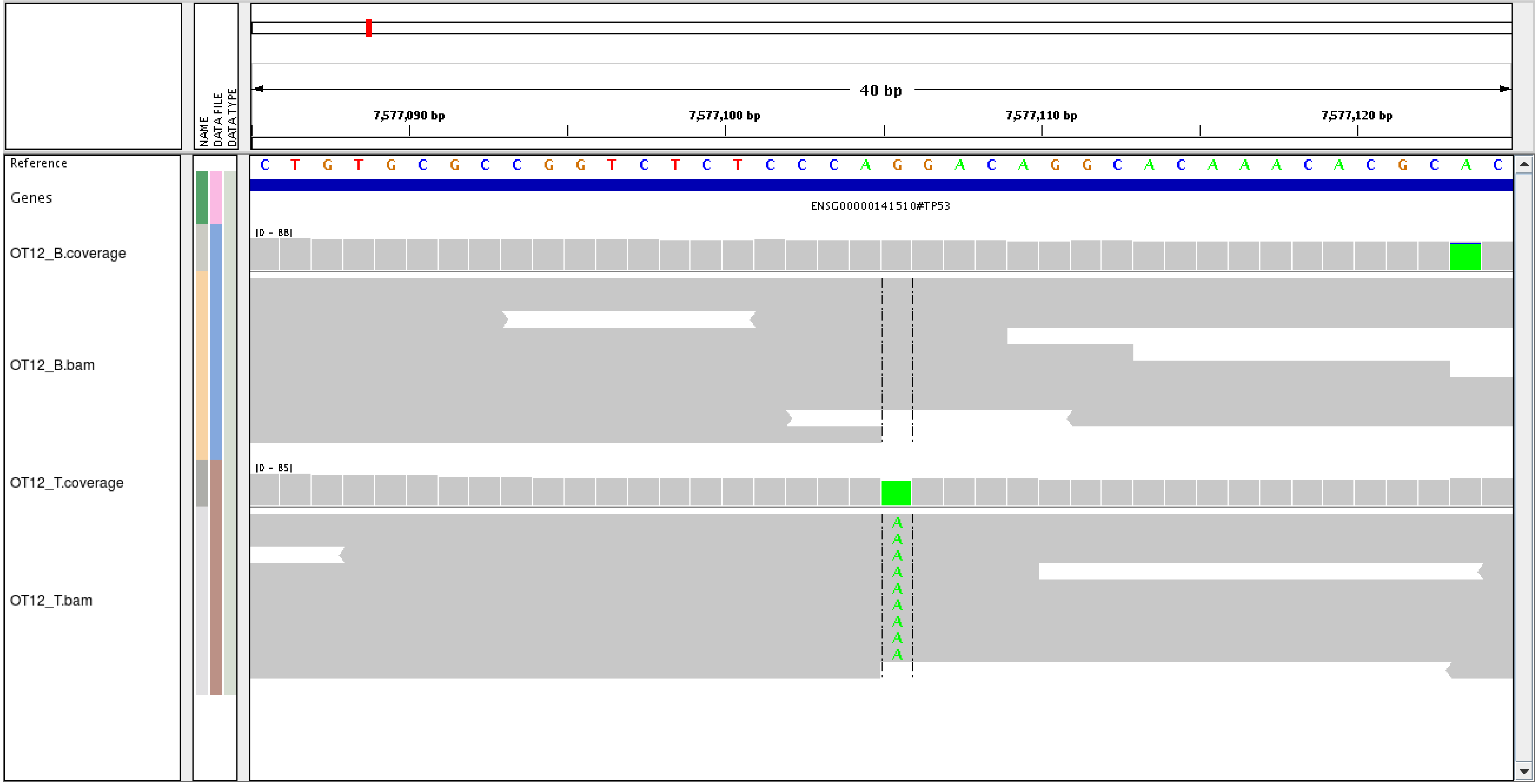

Event: chr9 139409752

Sample: OT14

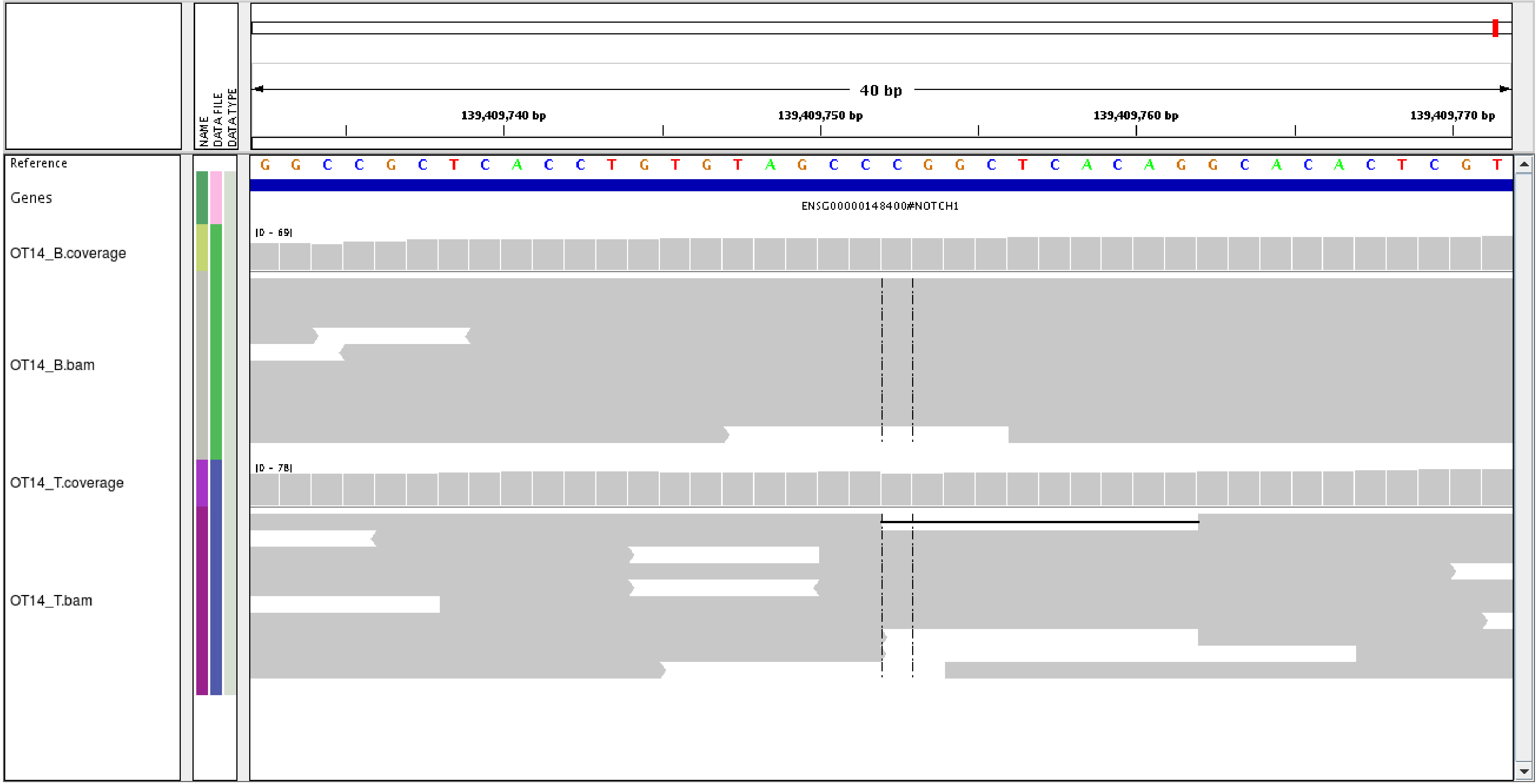

Event: chr10 61956370

Sample: OT18

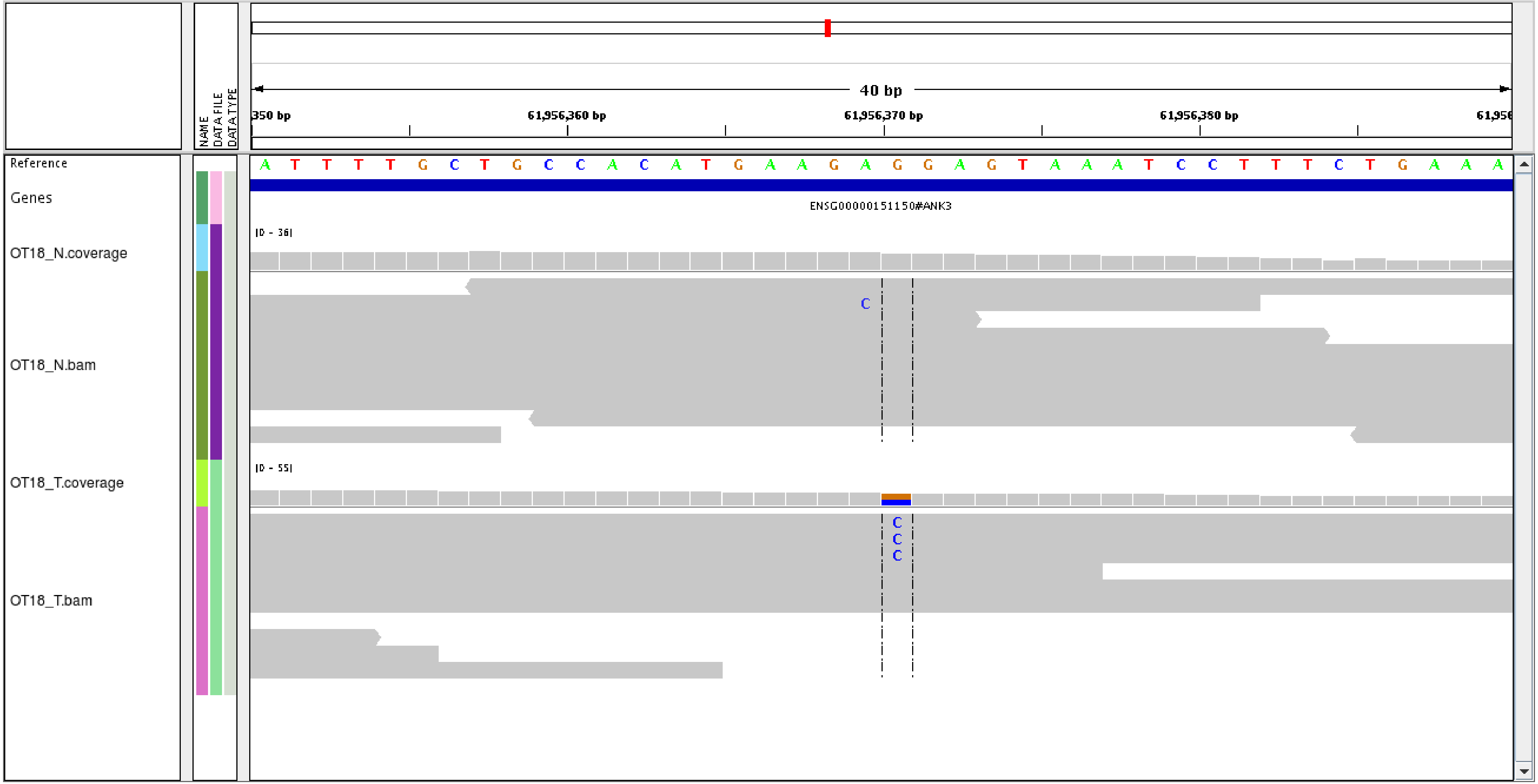

Event: chr17 7578190

Sample: OT18

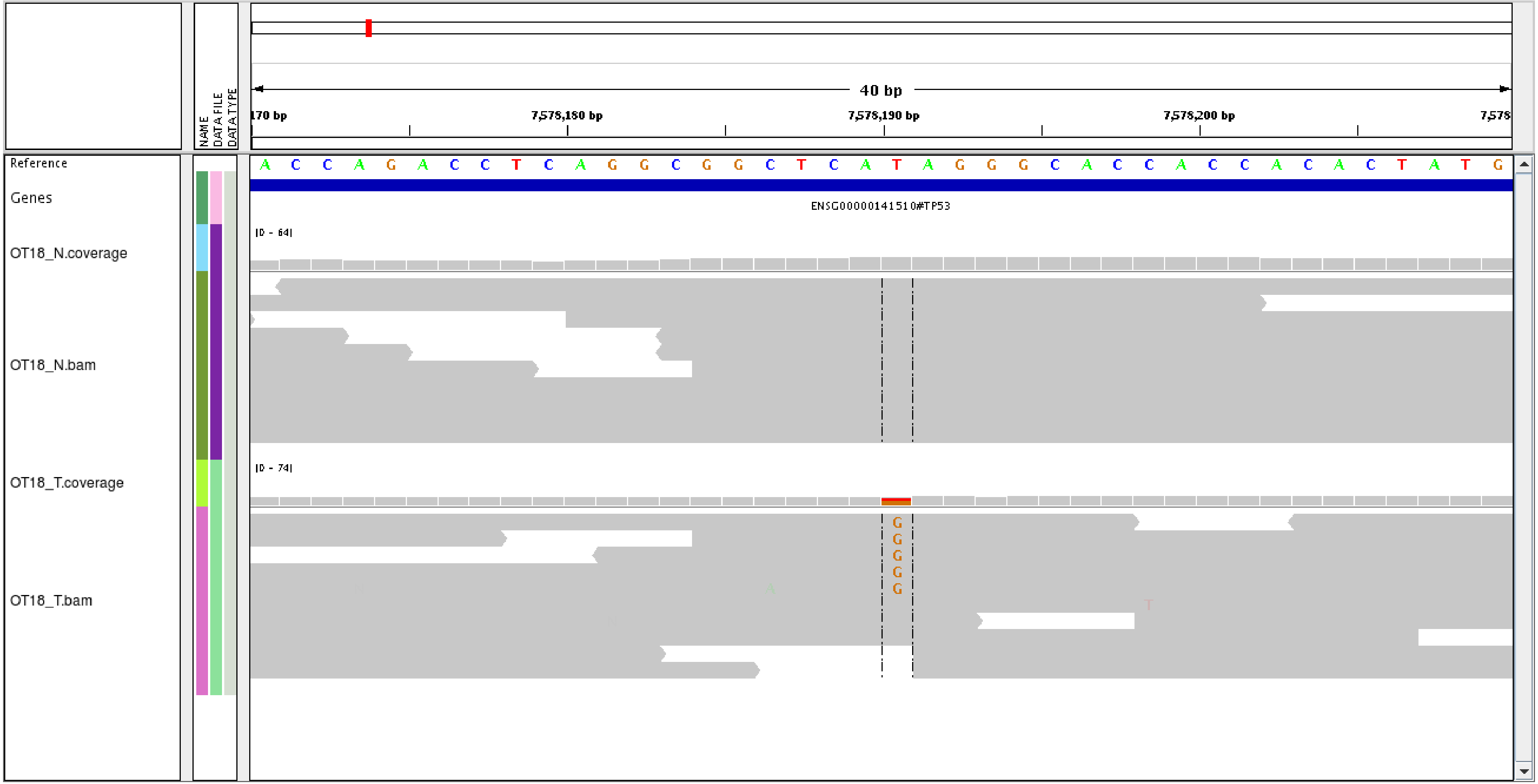

Event: chr1 120572547

Sample: OT21_B

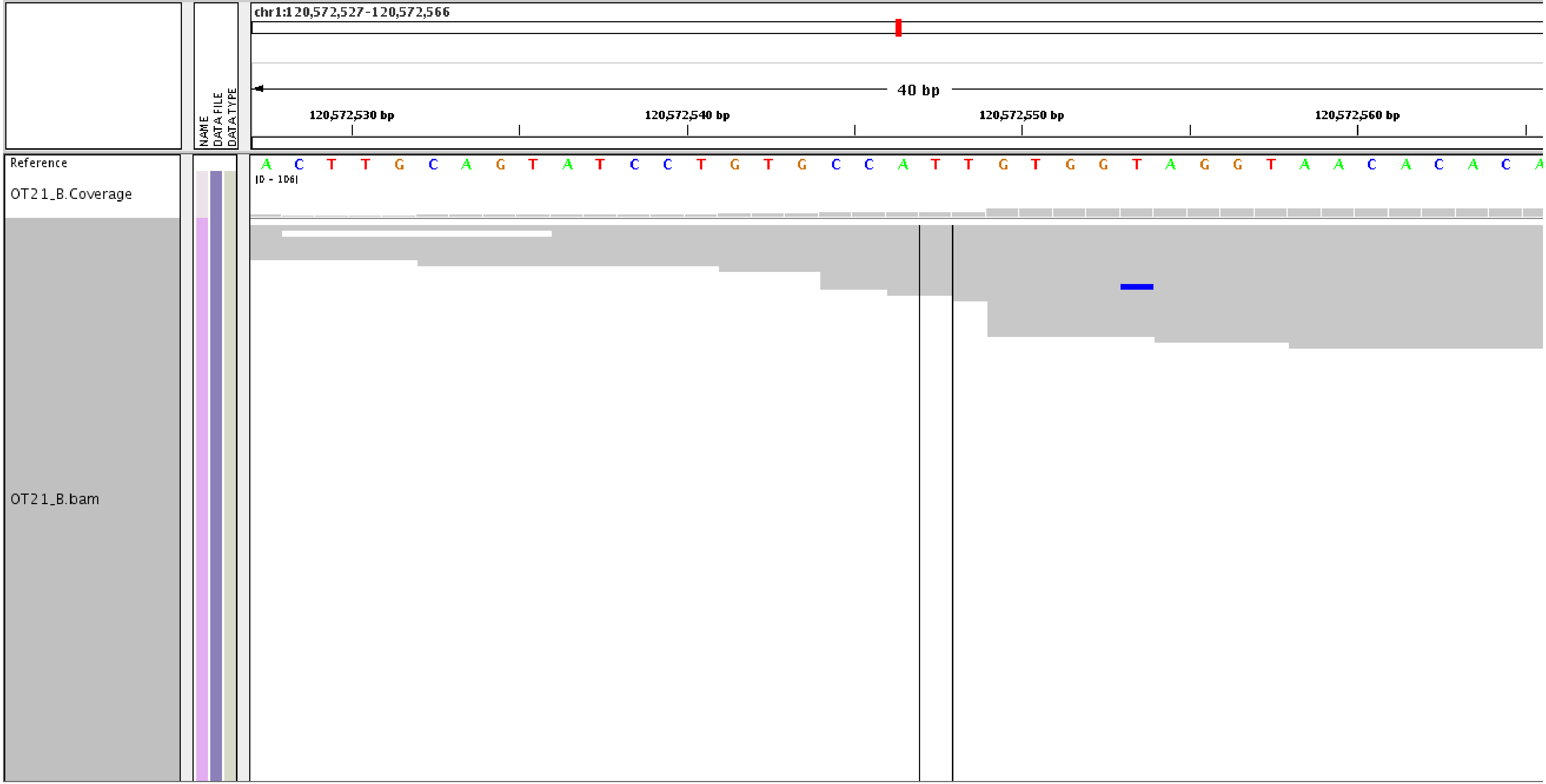

Event: chr1 120572547

Sample: OT21_T

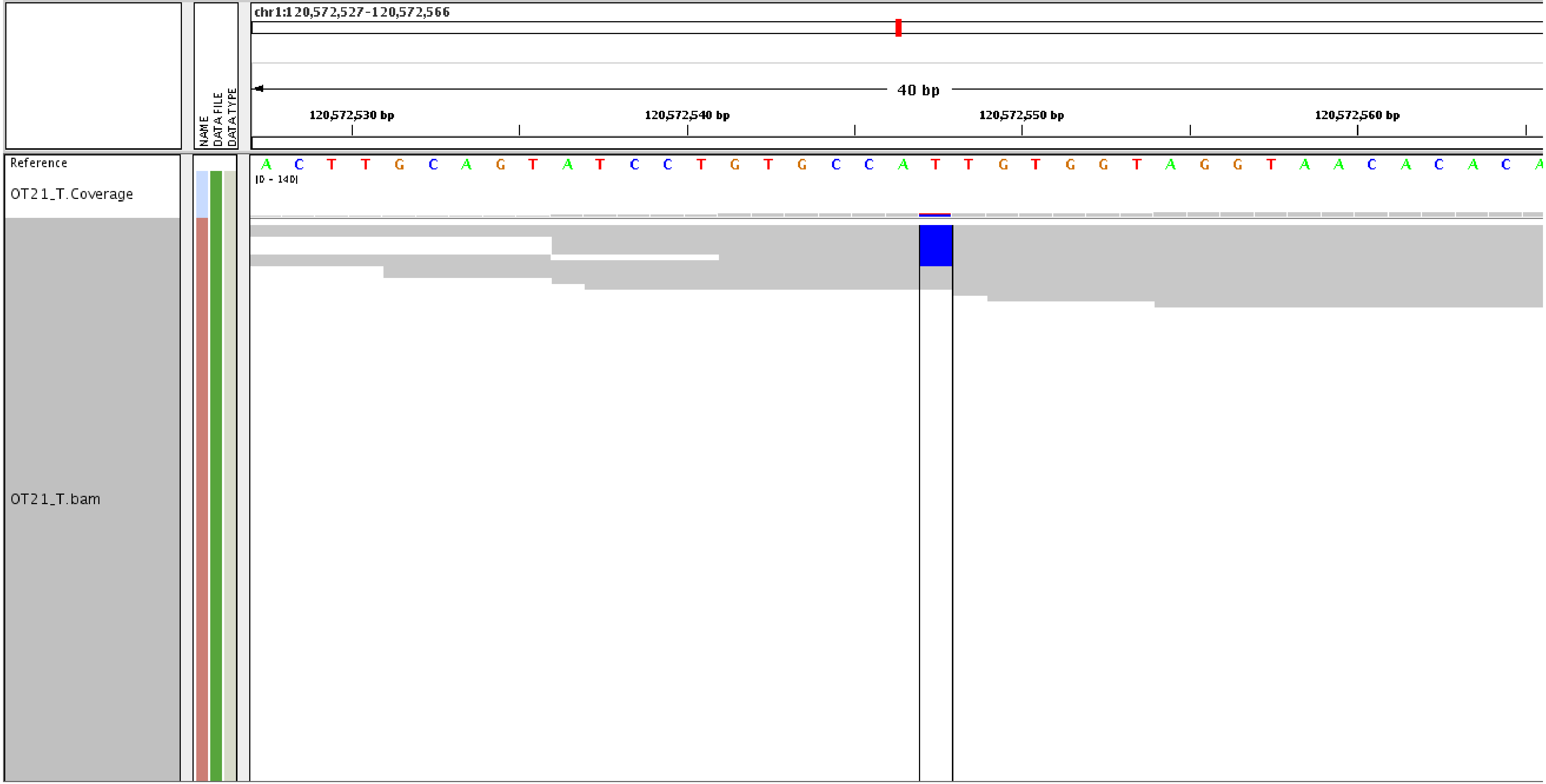

Event: chr9 21971110

Sample: OT21

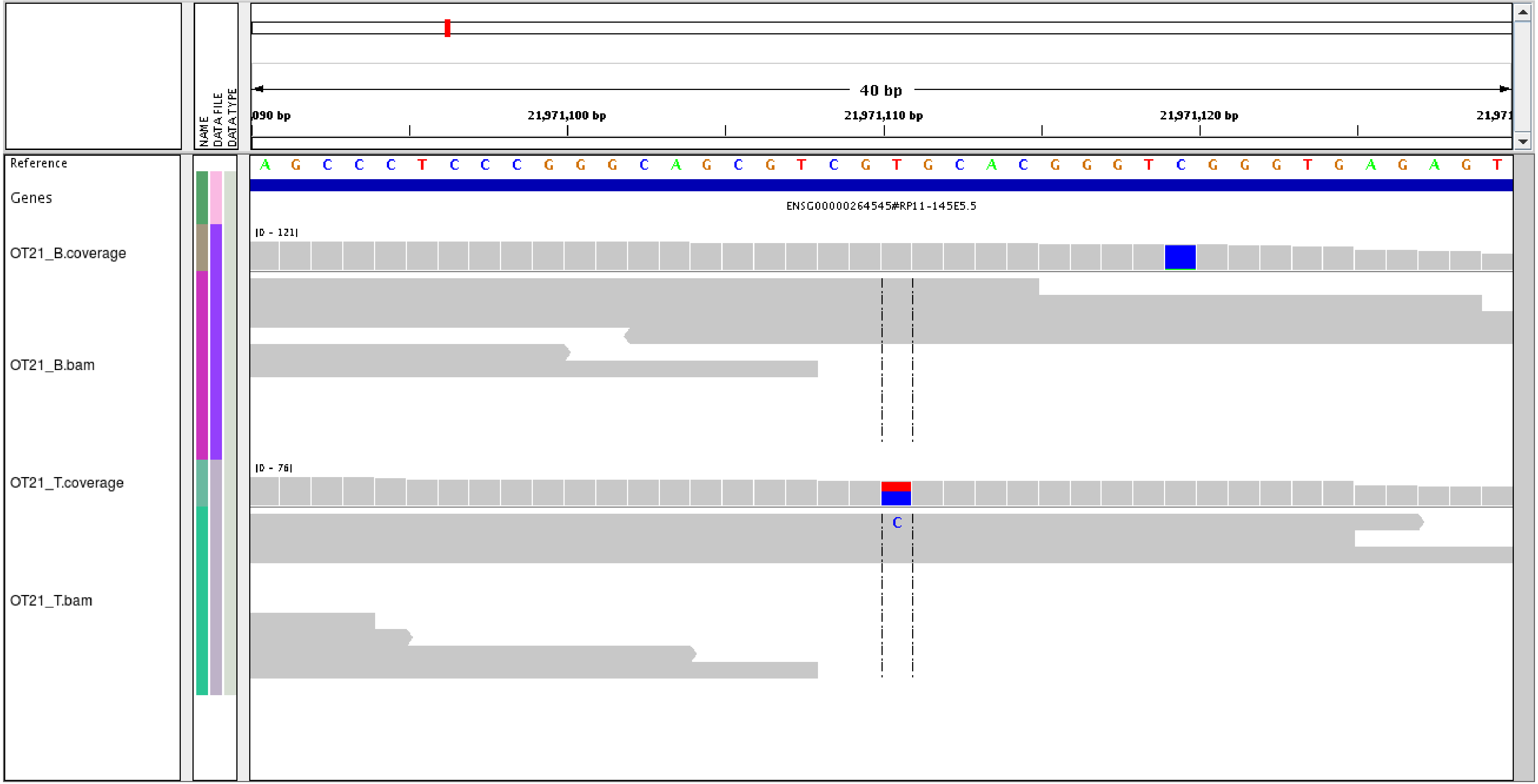

Event: chr17 7578524

Sample: OT22

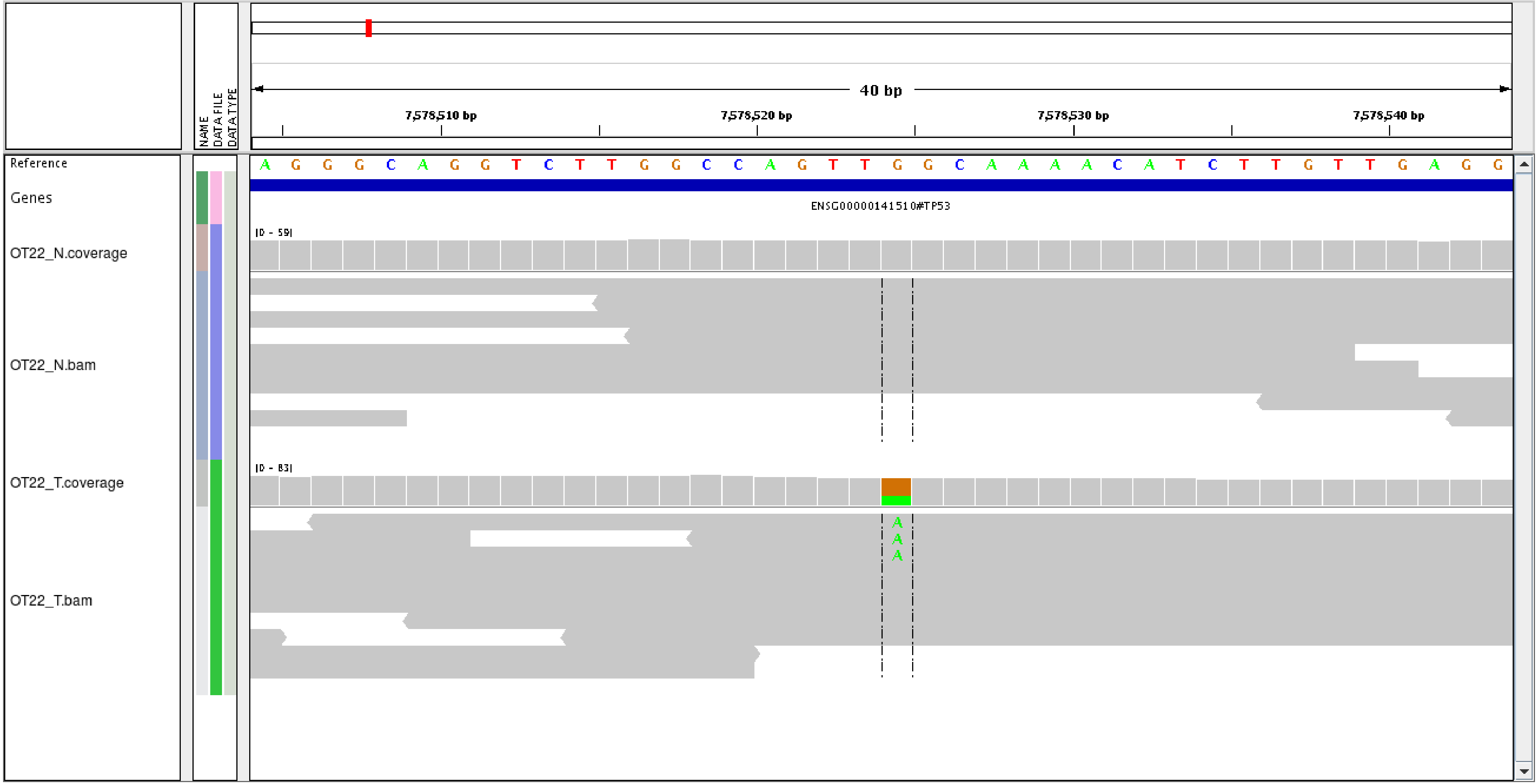

Event: chr3 142281612

Sample: OT23

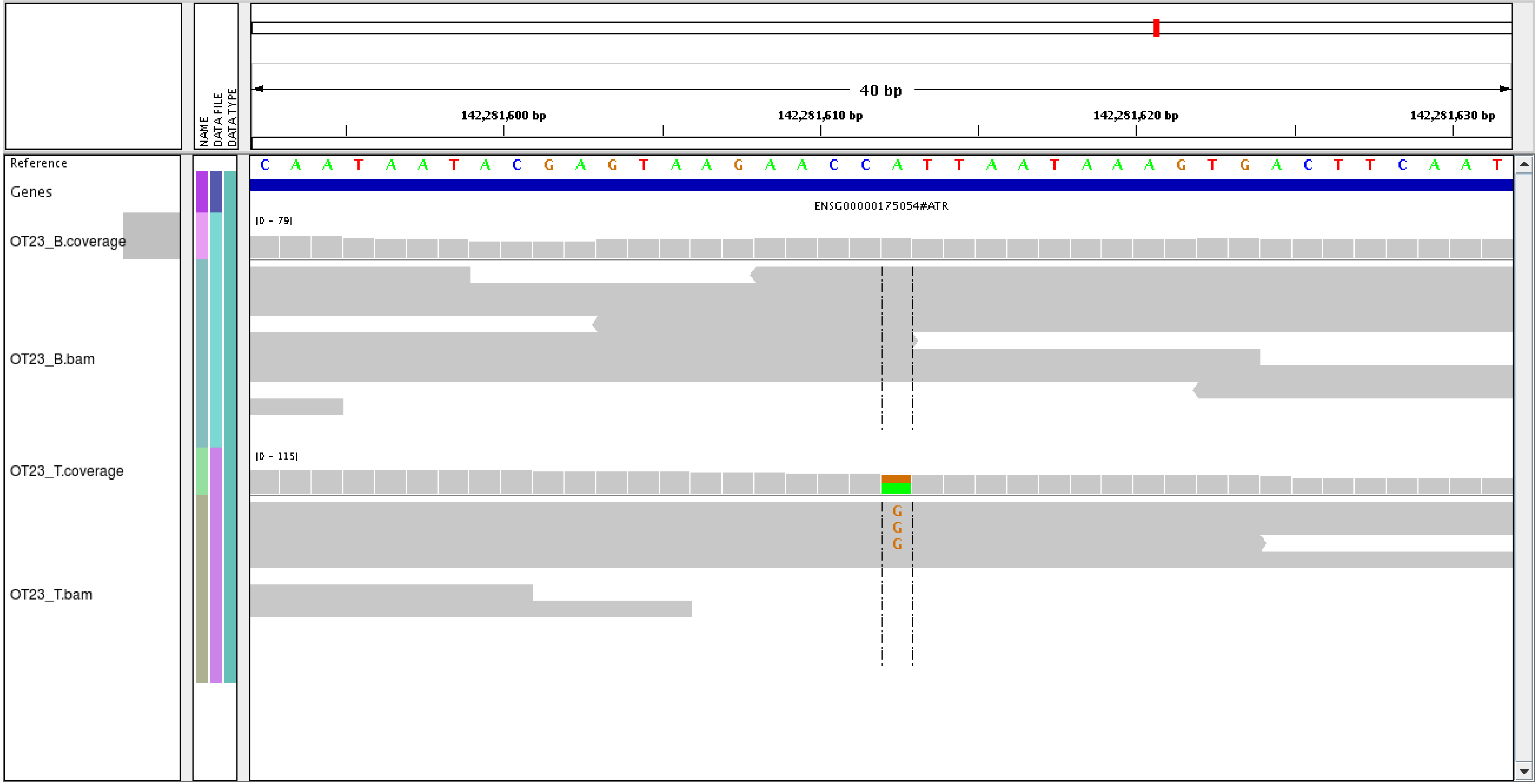

Event: chr17 7578395

Sample: OT23

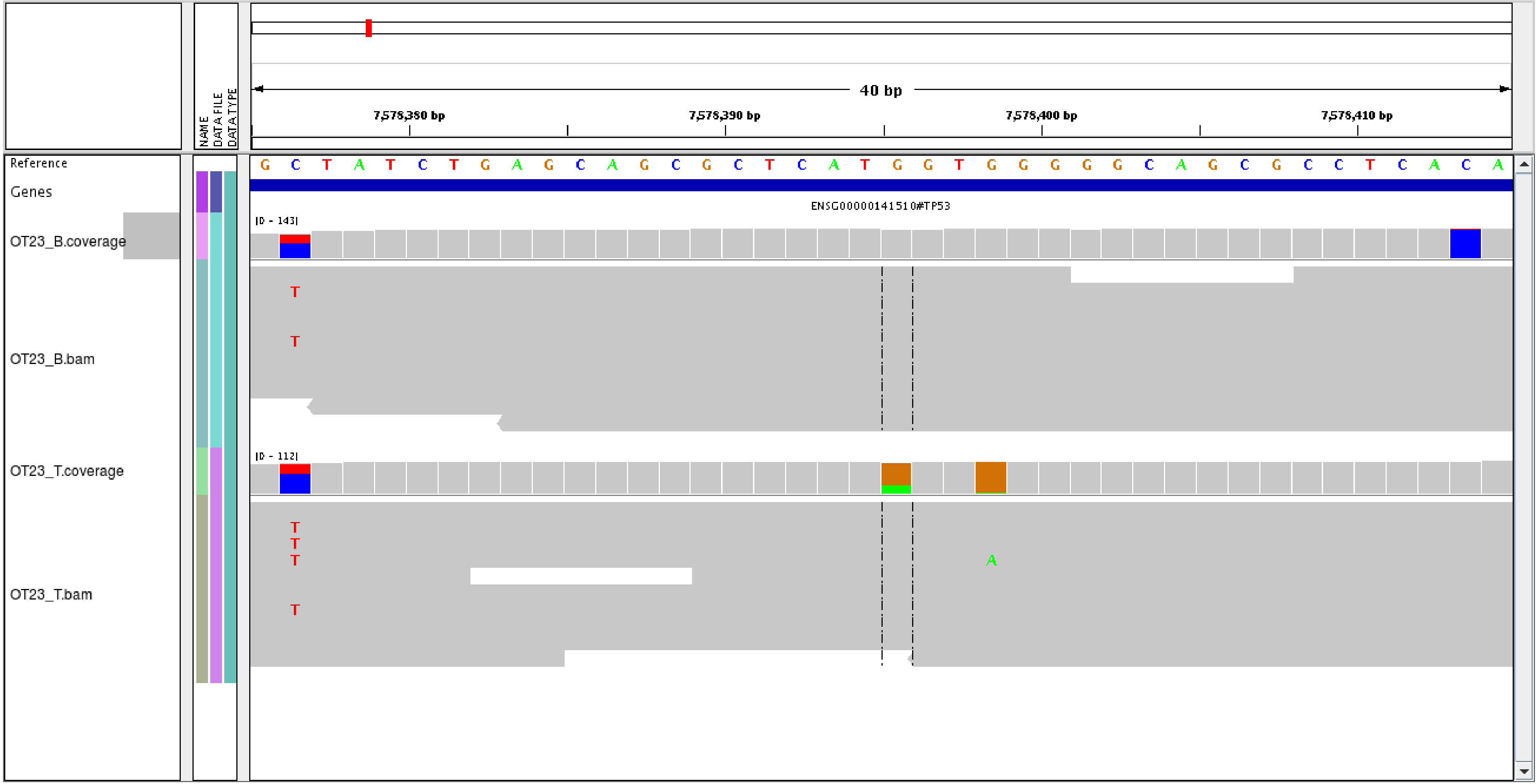

Event: chr4 187549359

Sample: OT24_B

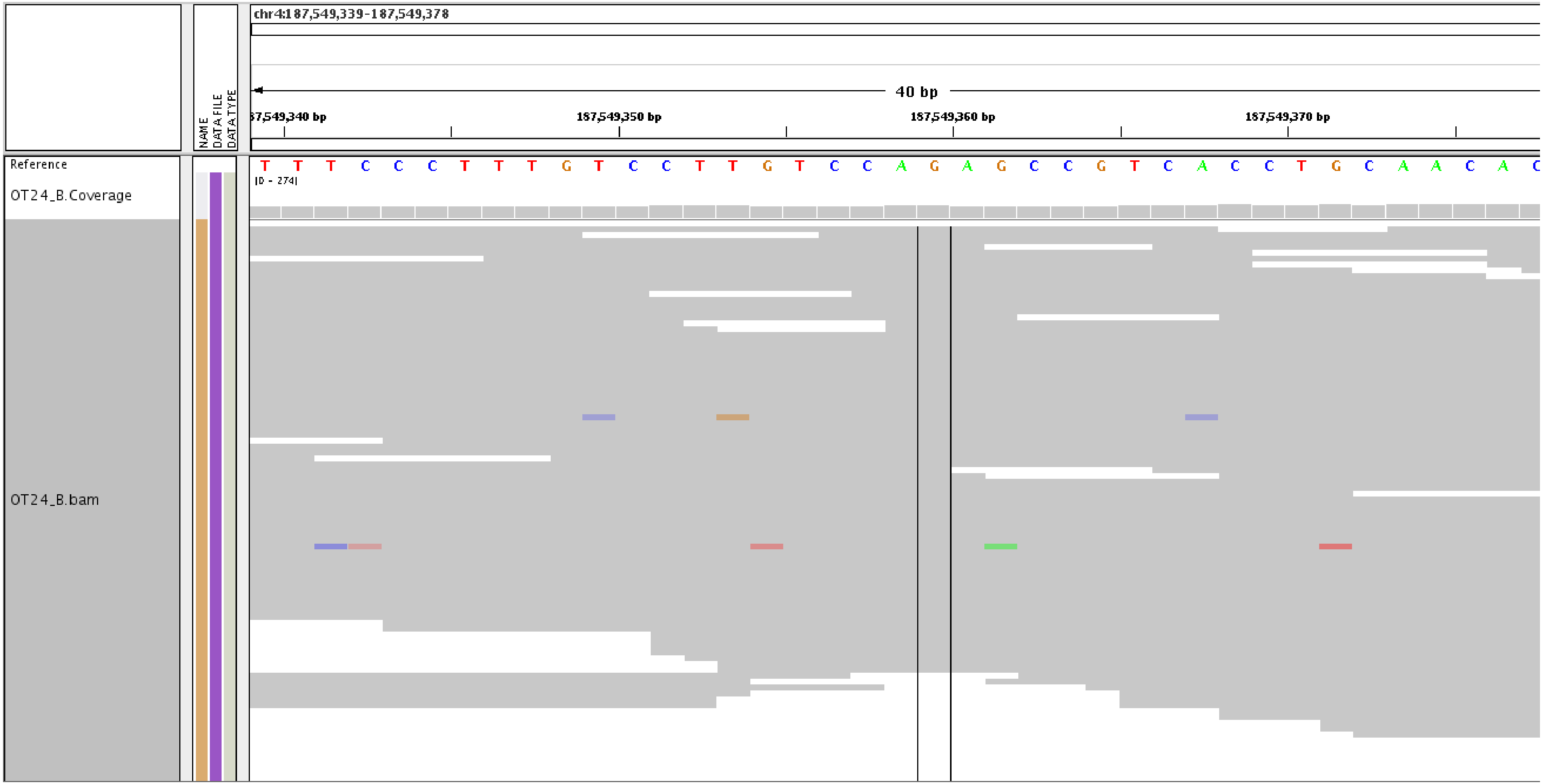

Event: chr4 187549359

Sample: OT24_T

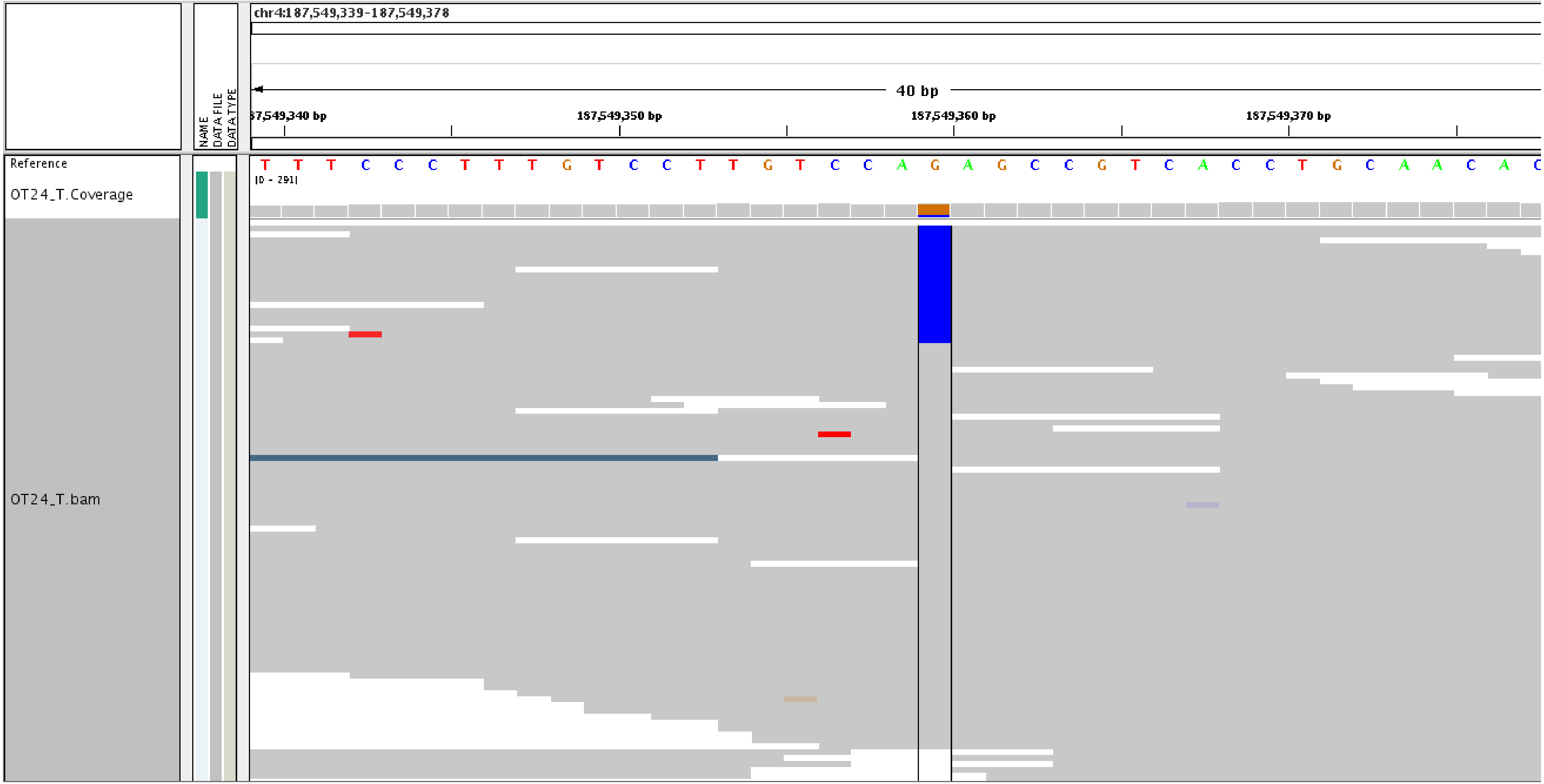

Event: chr17 7578211

Sample: OT24

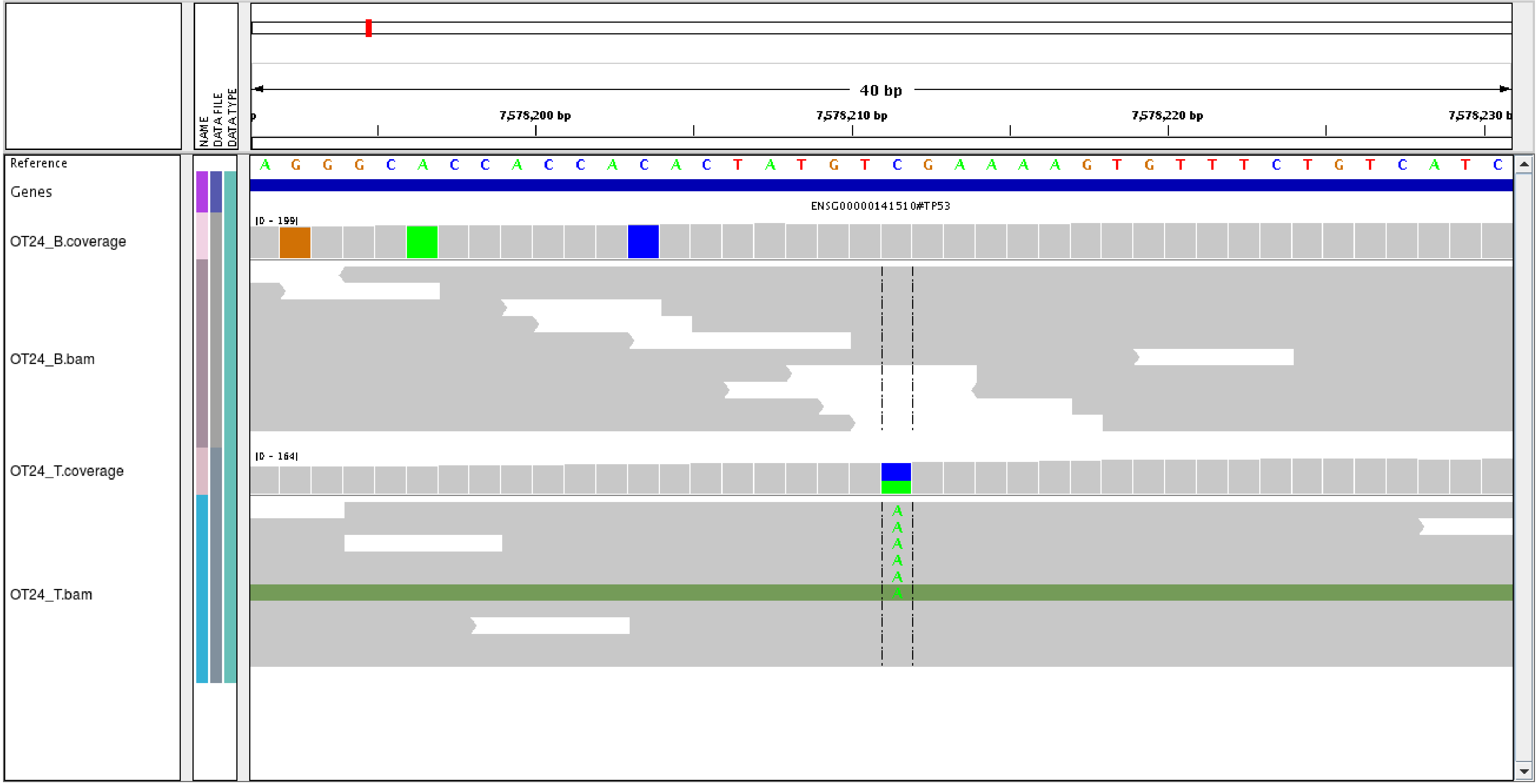

Event: chr1 27023377

Sample: OT25

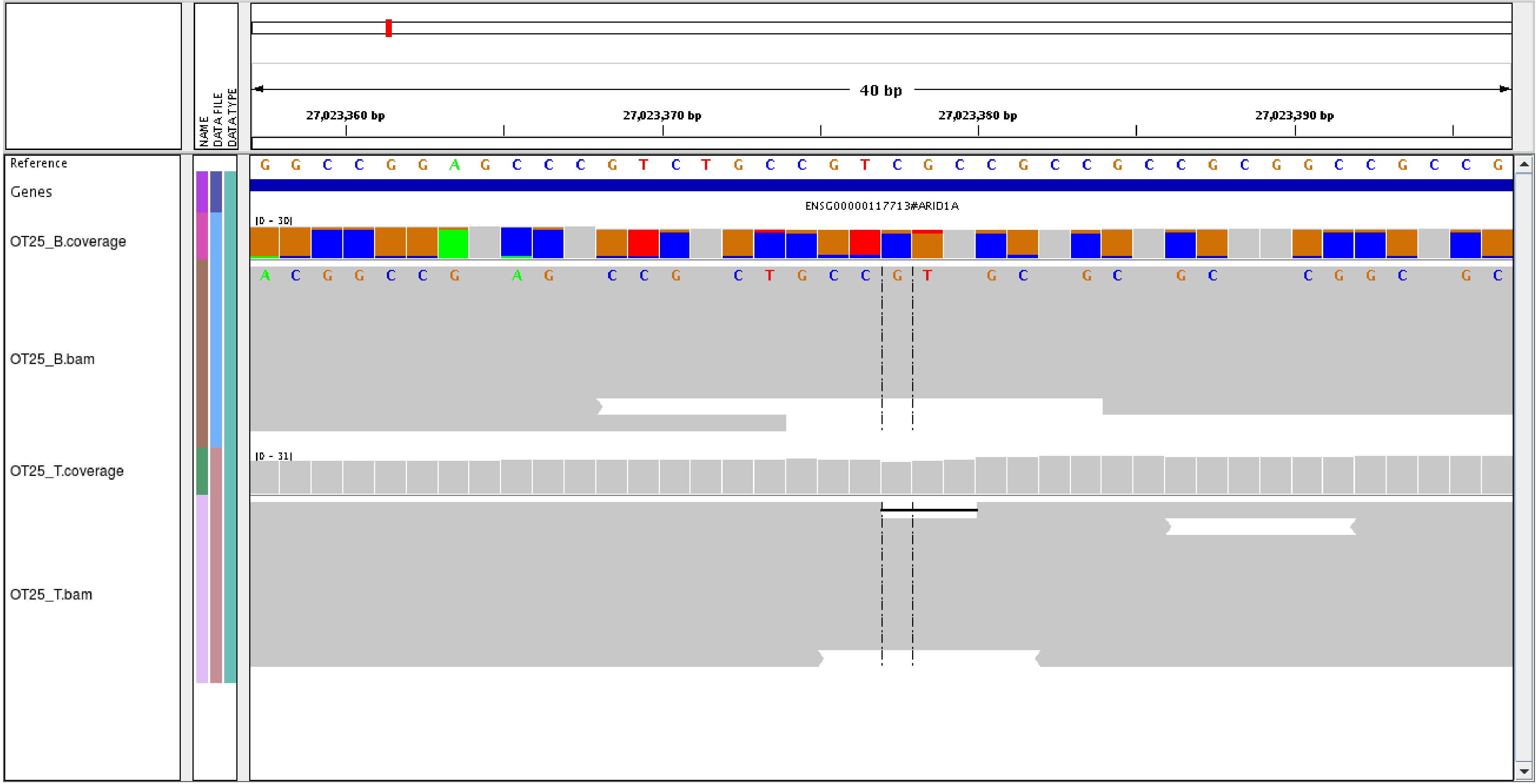

Event: chr2 202136260

Sample: OT25

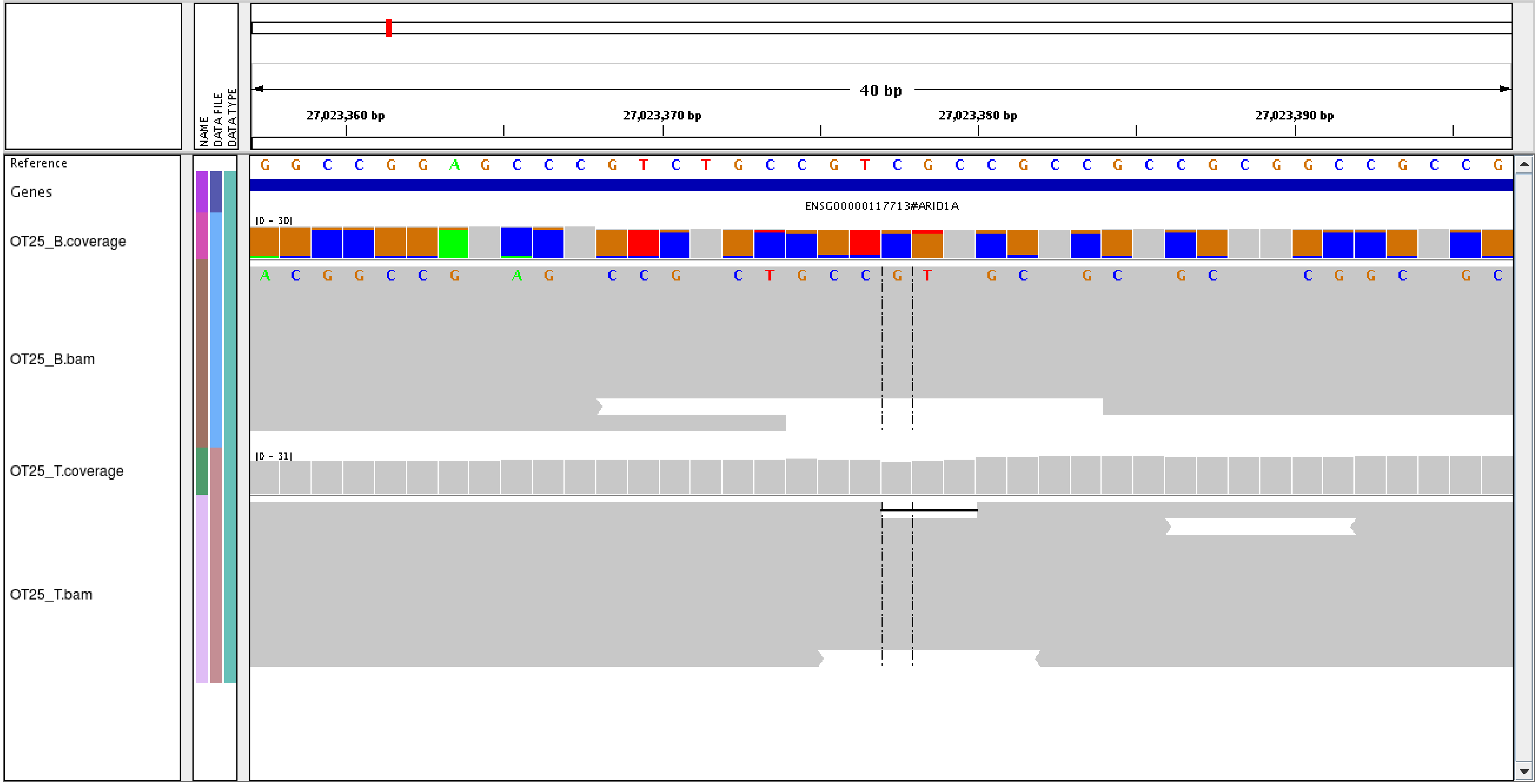

Event: chr17 7578406

Sample: OT25

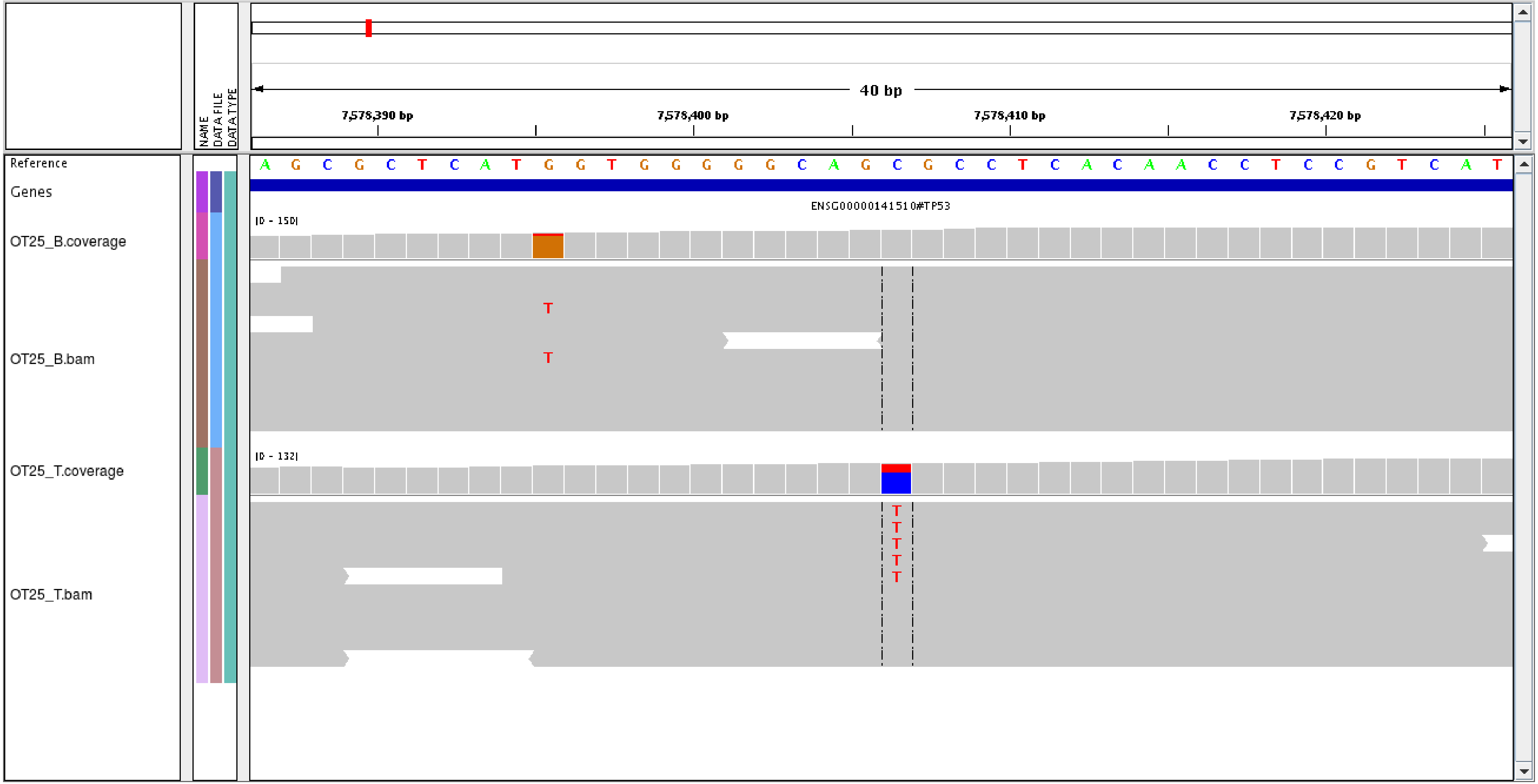

Event: chr1 228464276

Sample: OT27

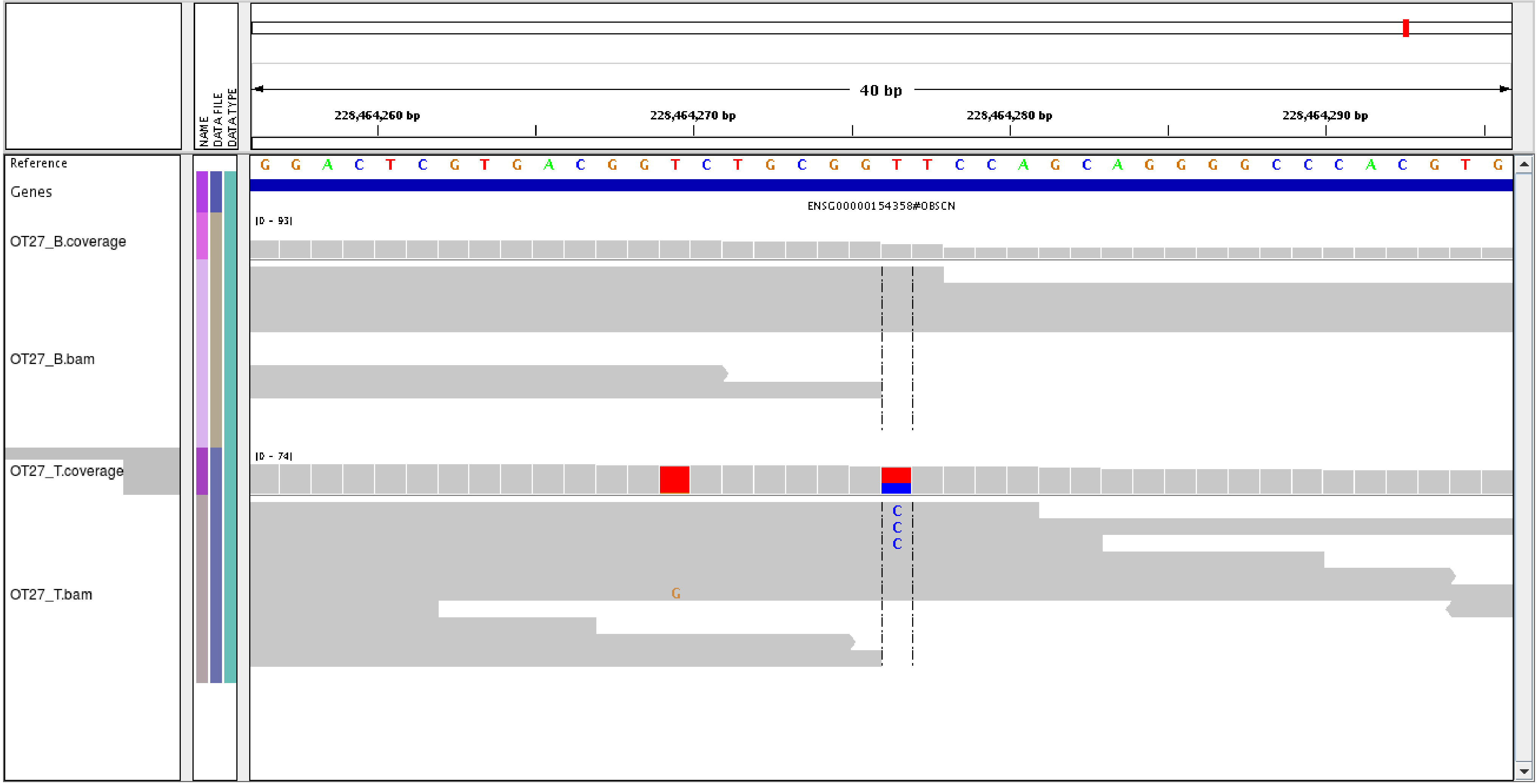

Event: chr17 7574003

Sample: OT27

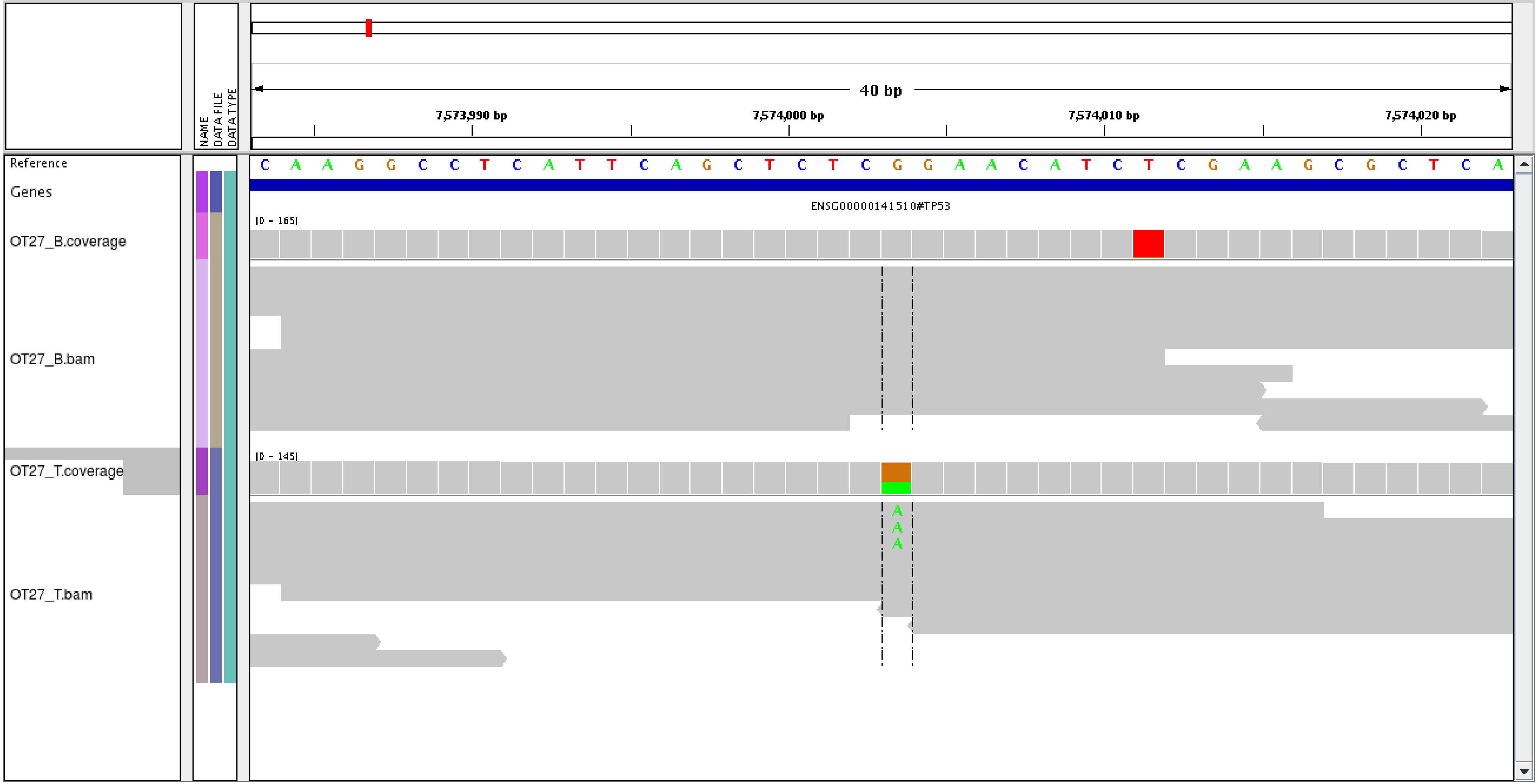

Event: chr17 7577539

Sample: OT27

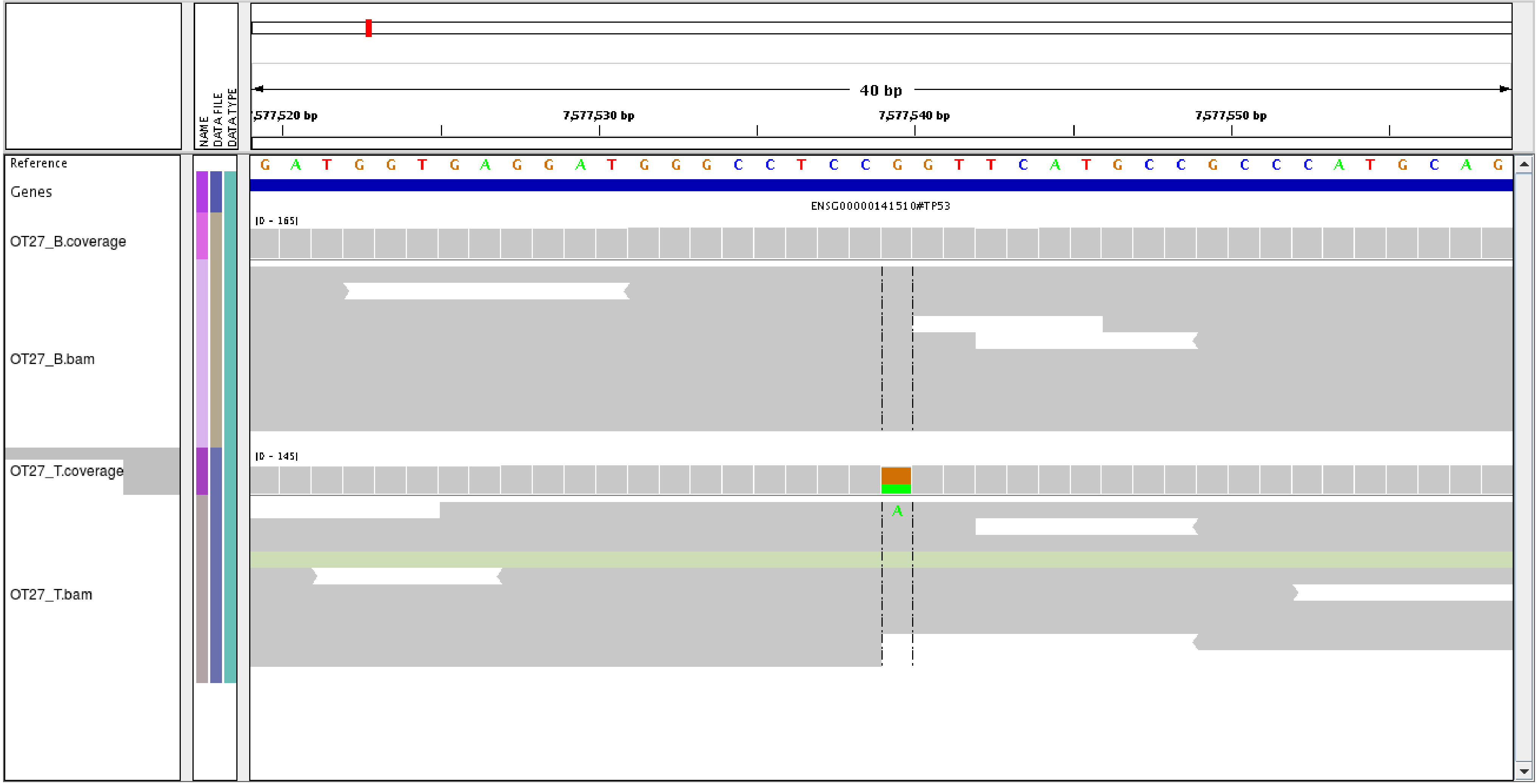

Event: chr2 202149914

Sample: OT27

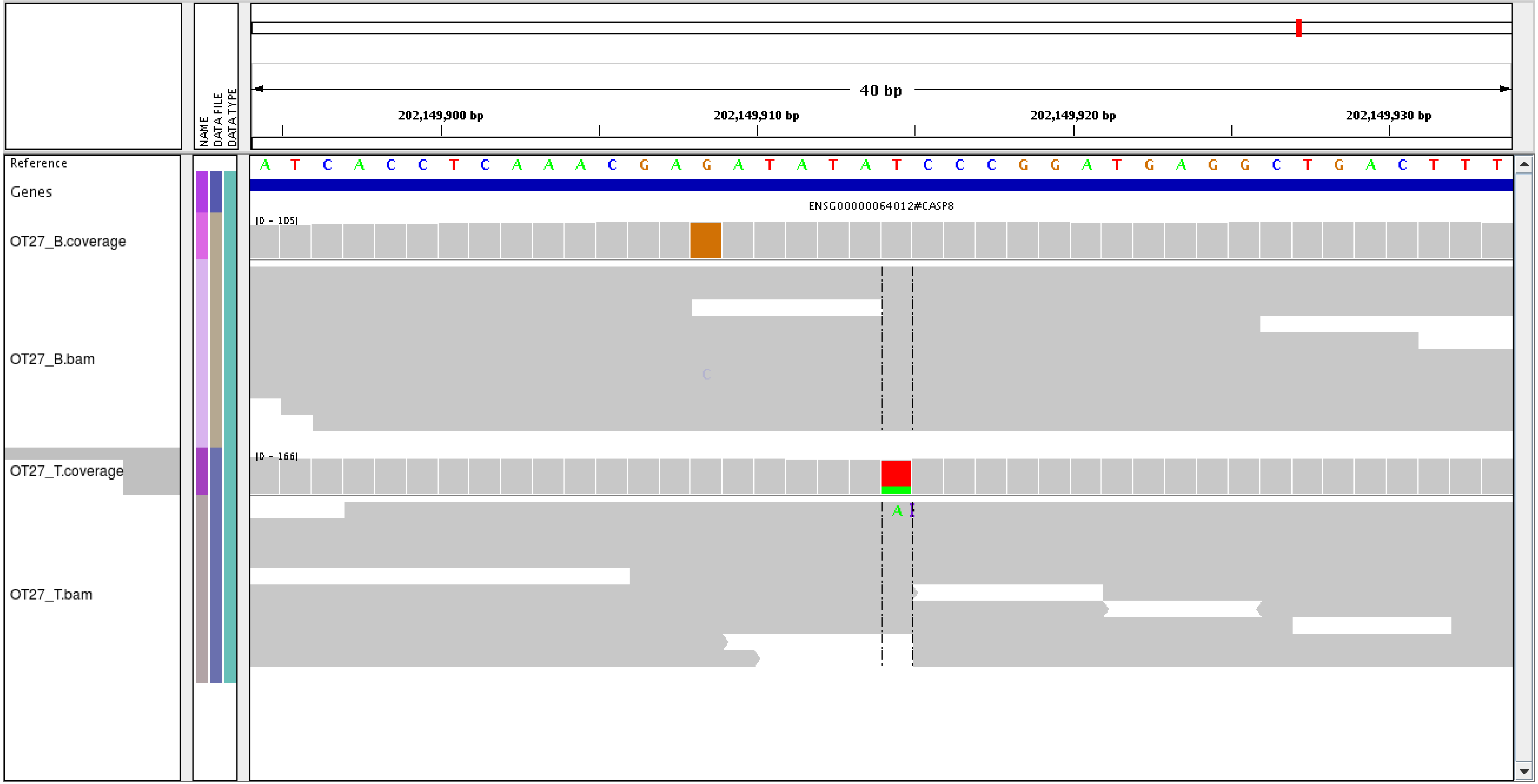

Event: chr3 178952085

Sample: OT27

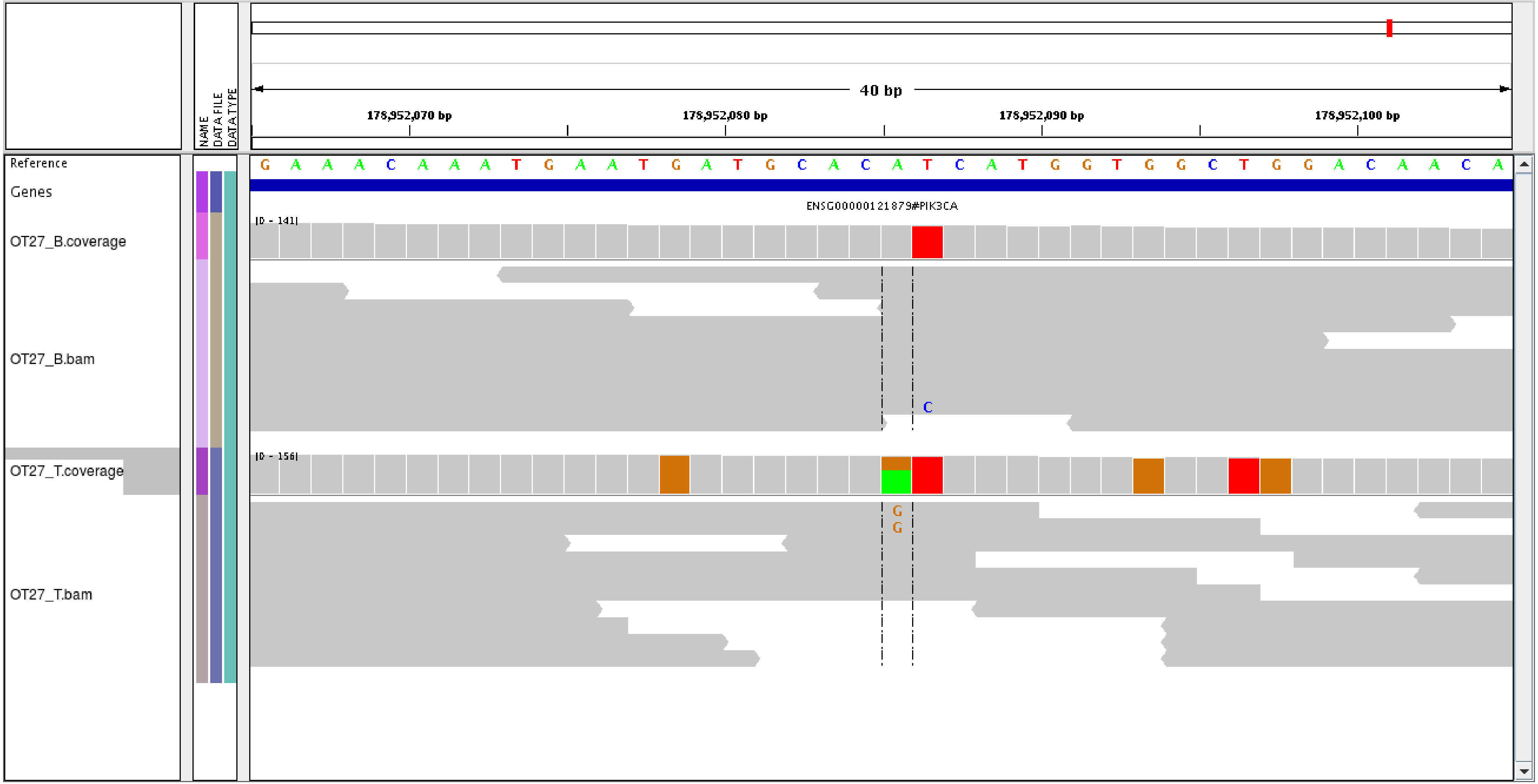

Event: chrX 32519887

Sample: OT27

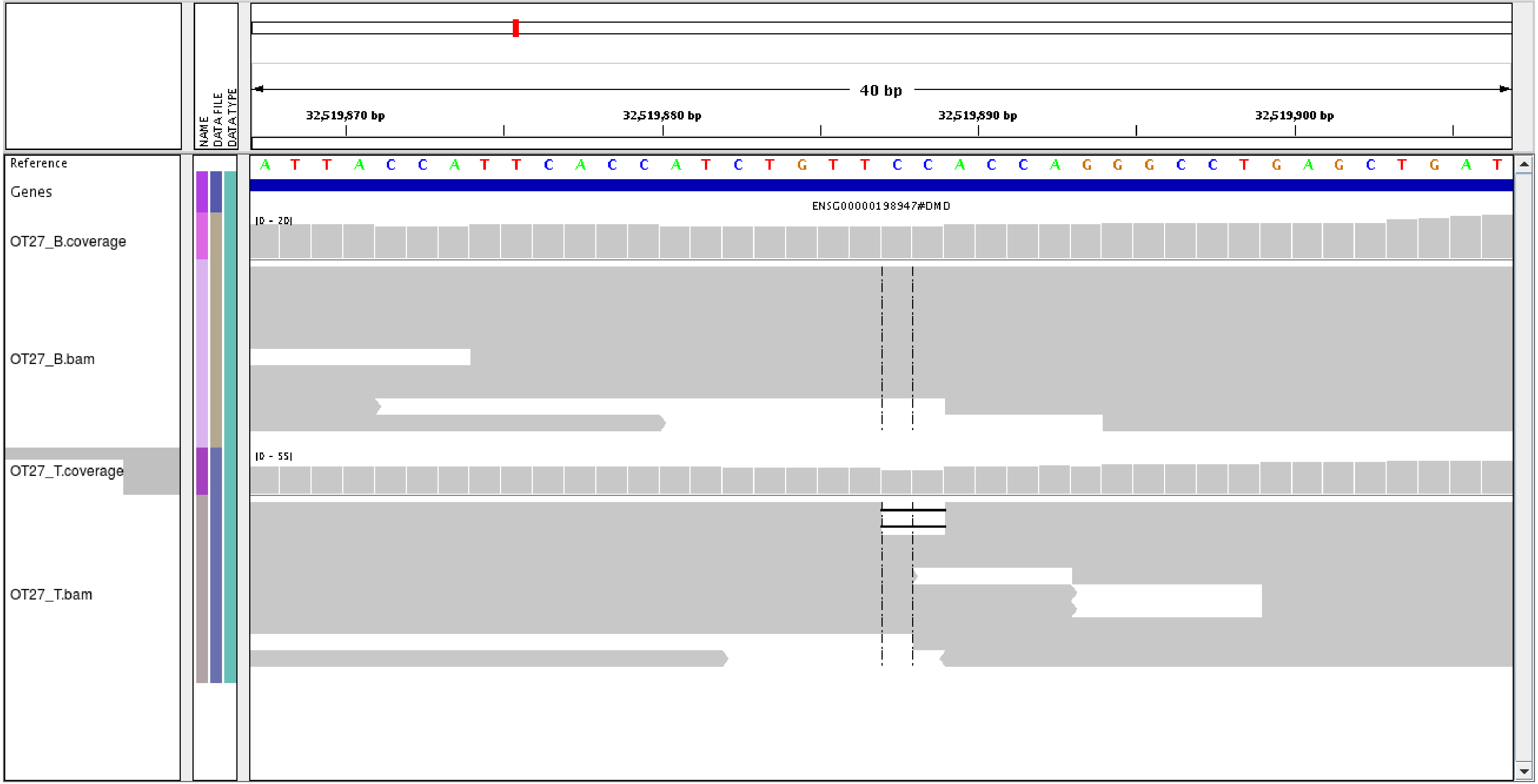

Event: chr5 86564737

Sample: OT32_B

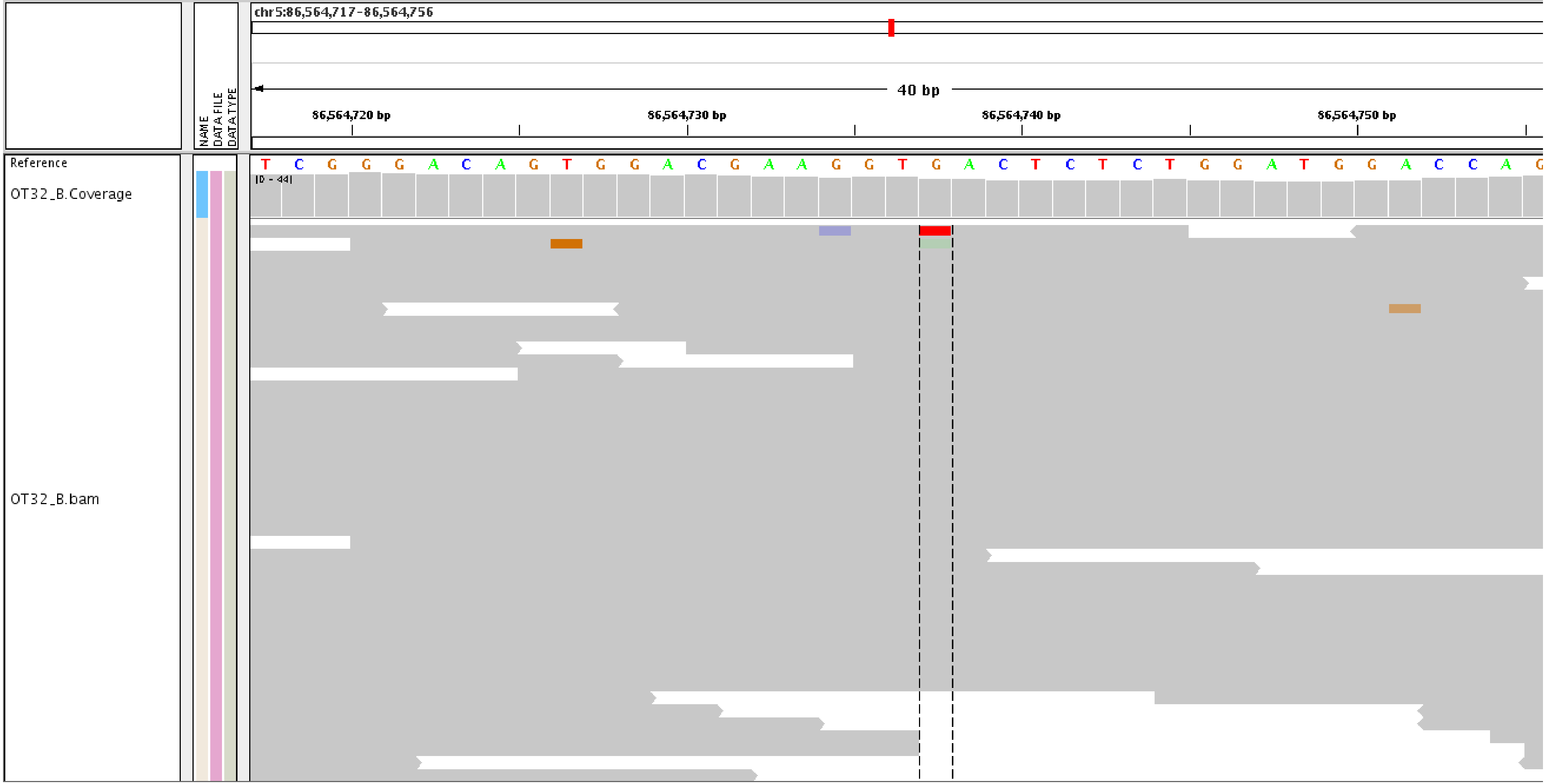

Event: chr5 86564737

Sample: OT32_T

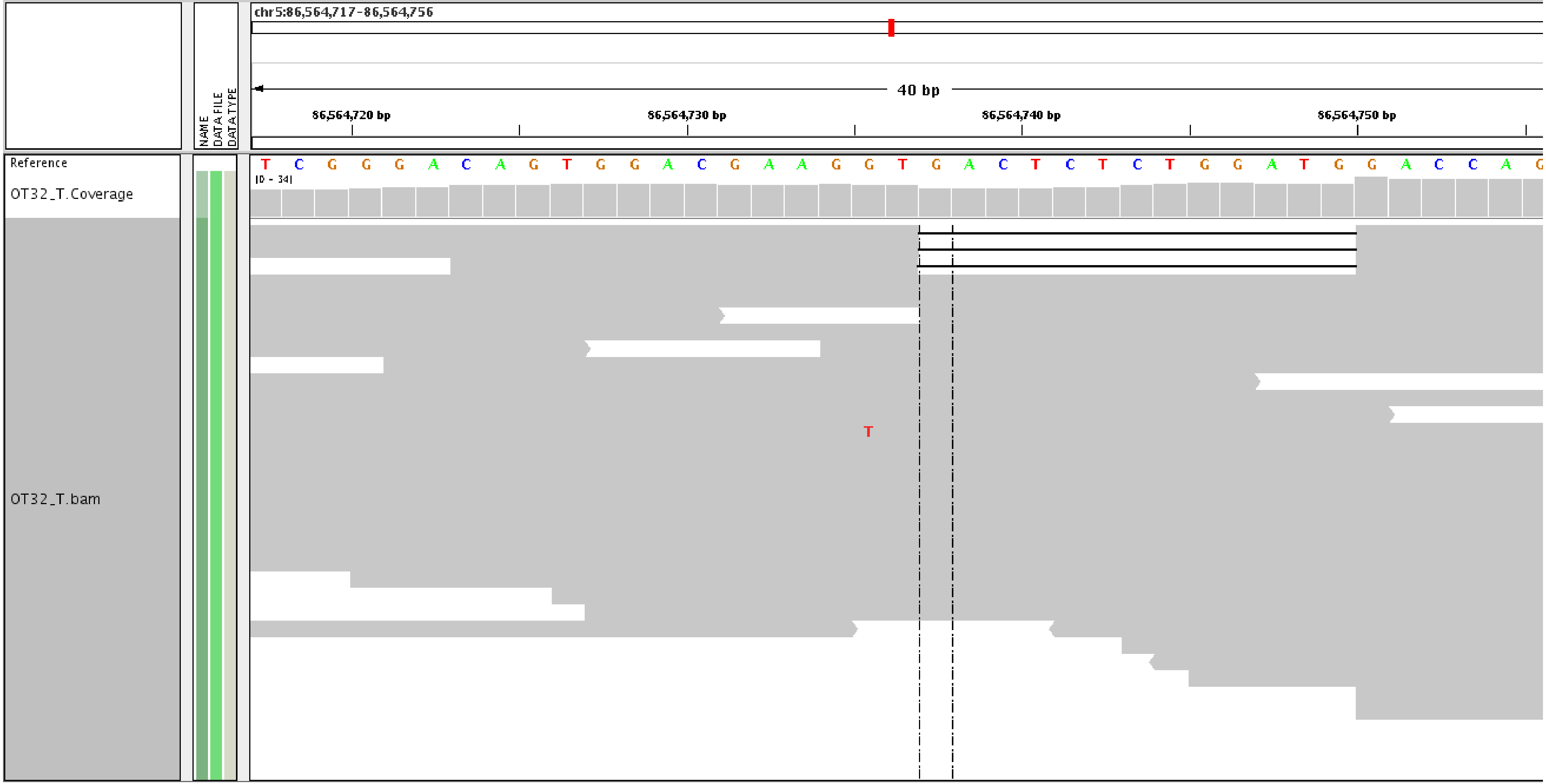

Event: chr9 21970971

Sample: OT32

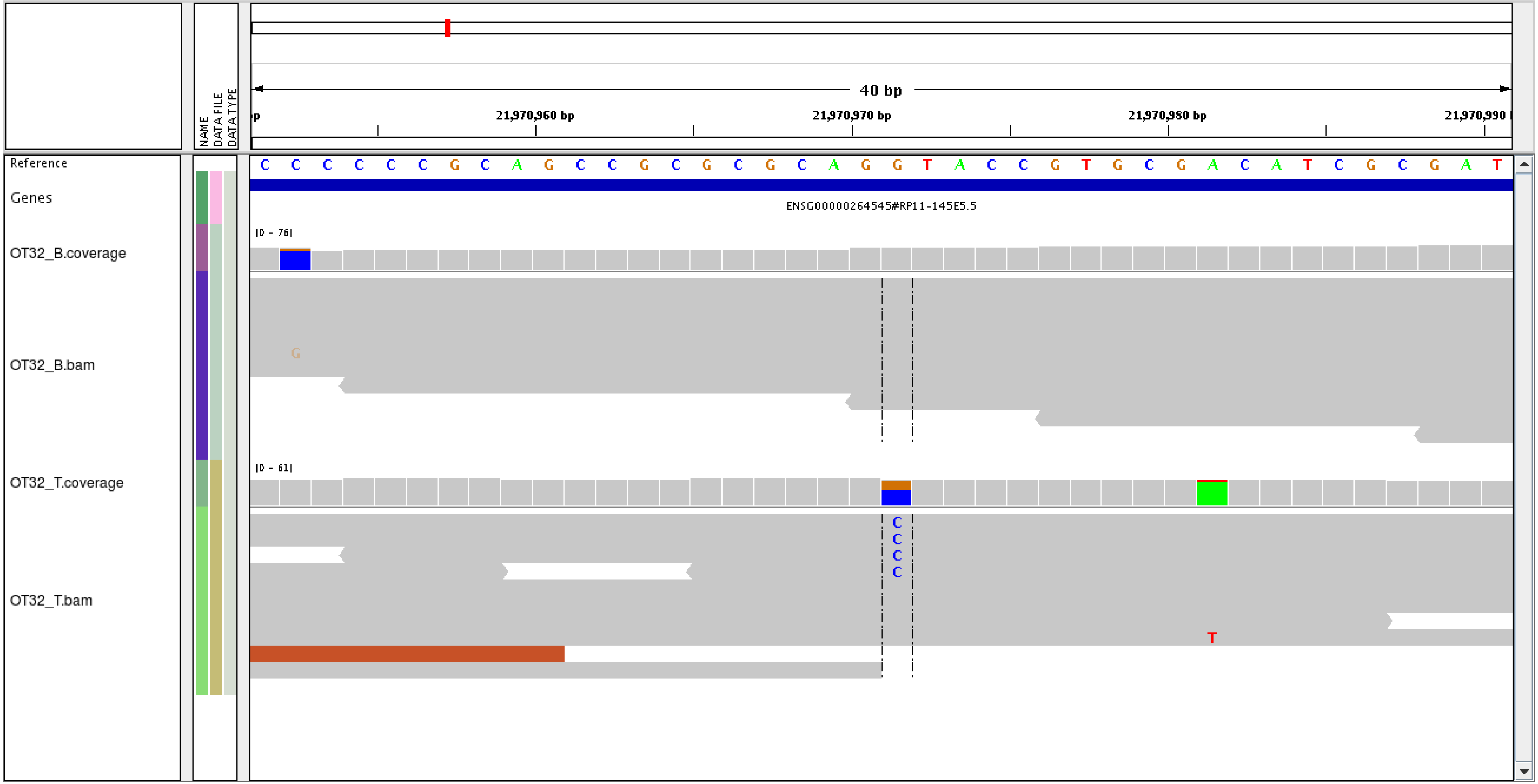

Event: chr9 139413179

Sample: OT32

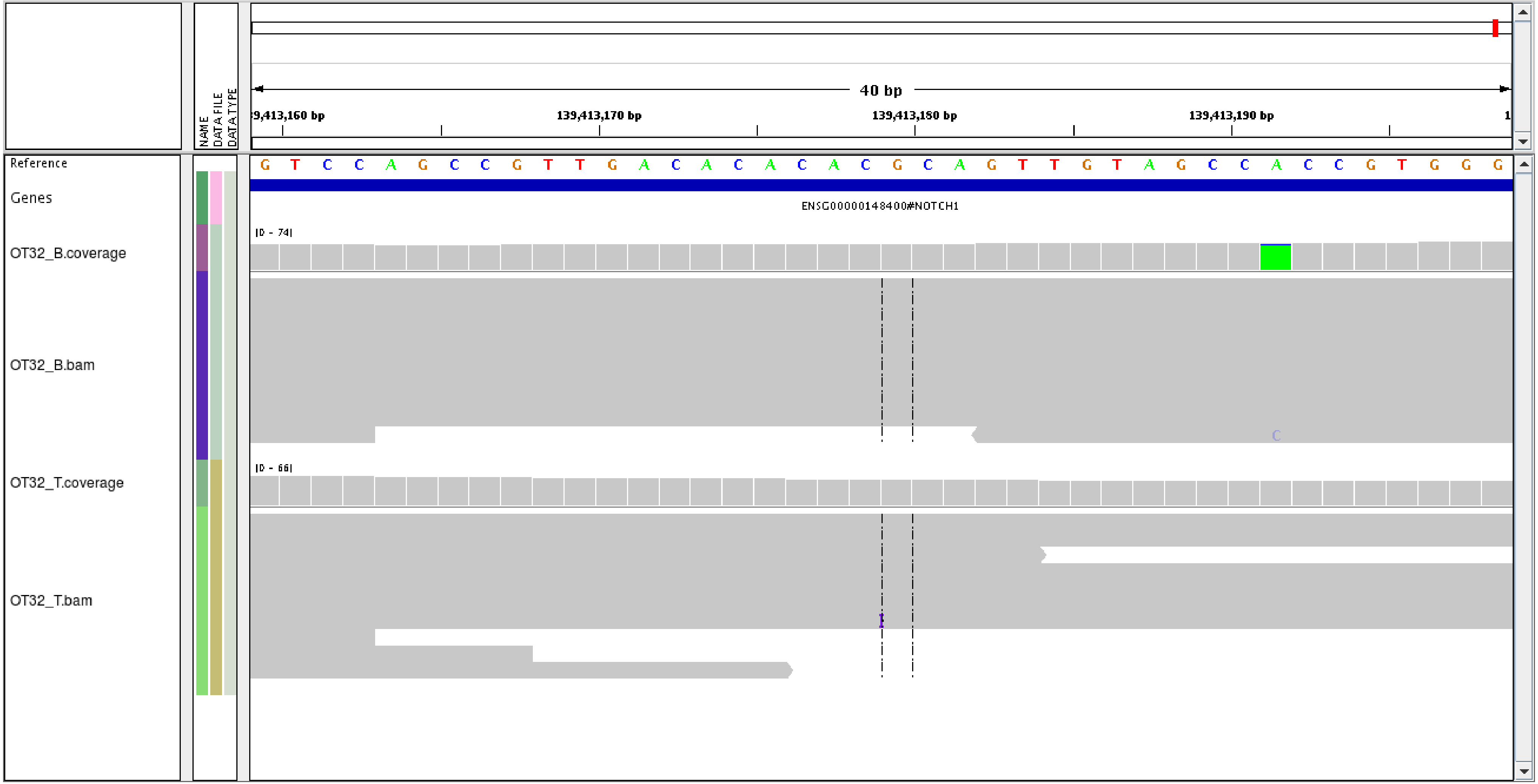

Event: chr1 120611964

Sample: OT33_B

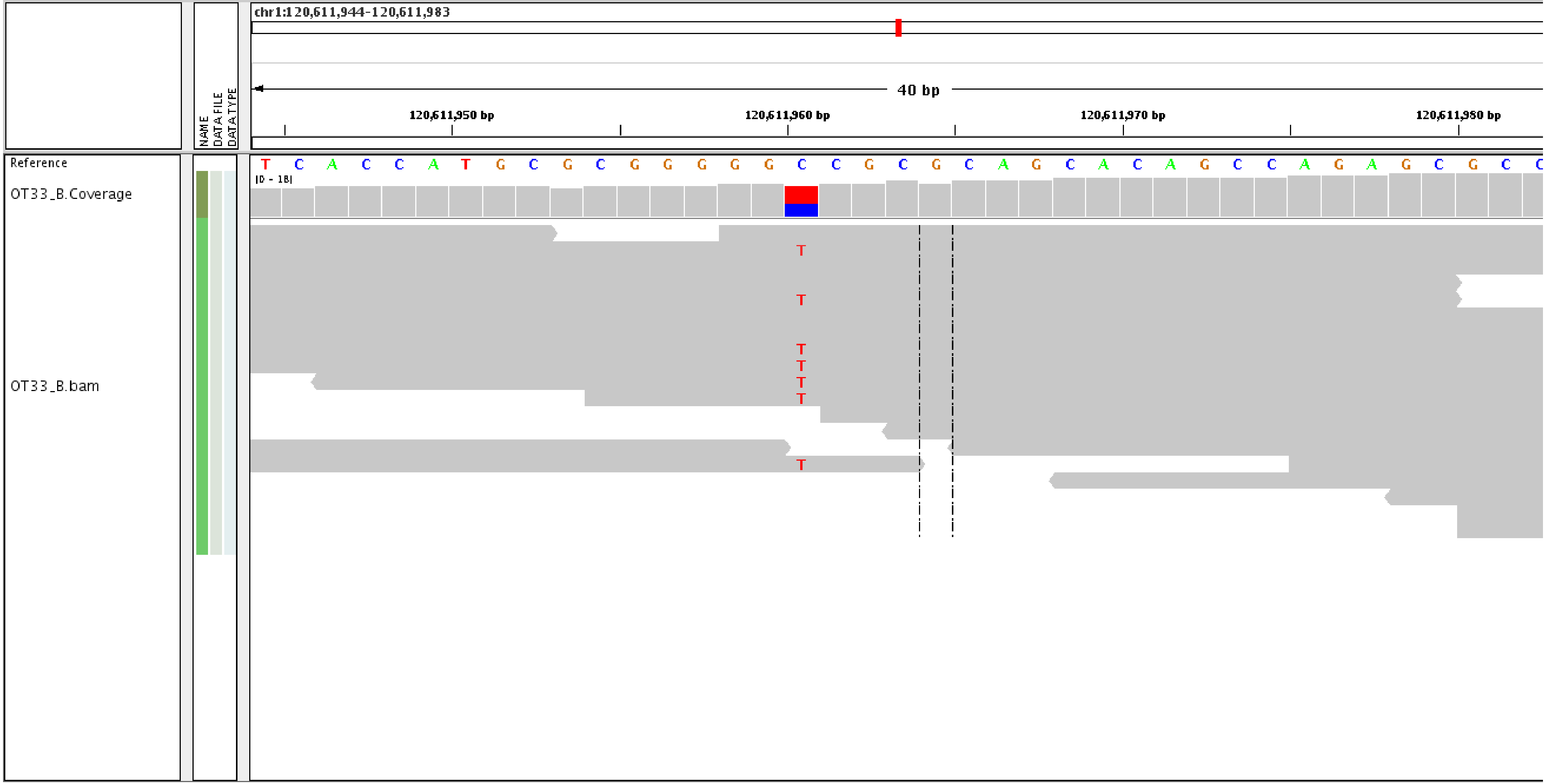

Event: chr1 120611964

Sample: OT33_T

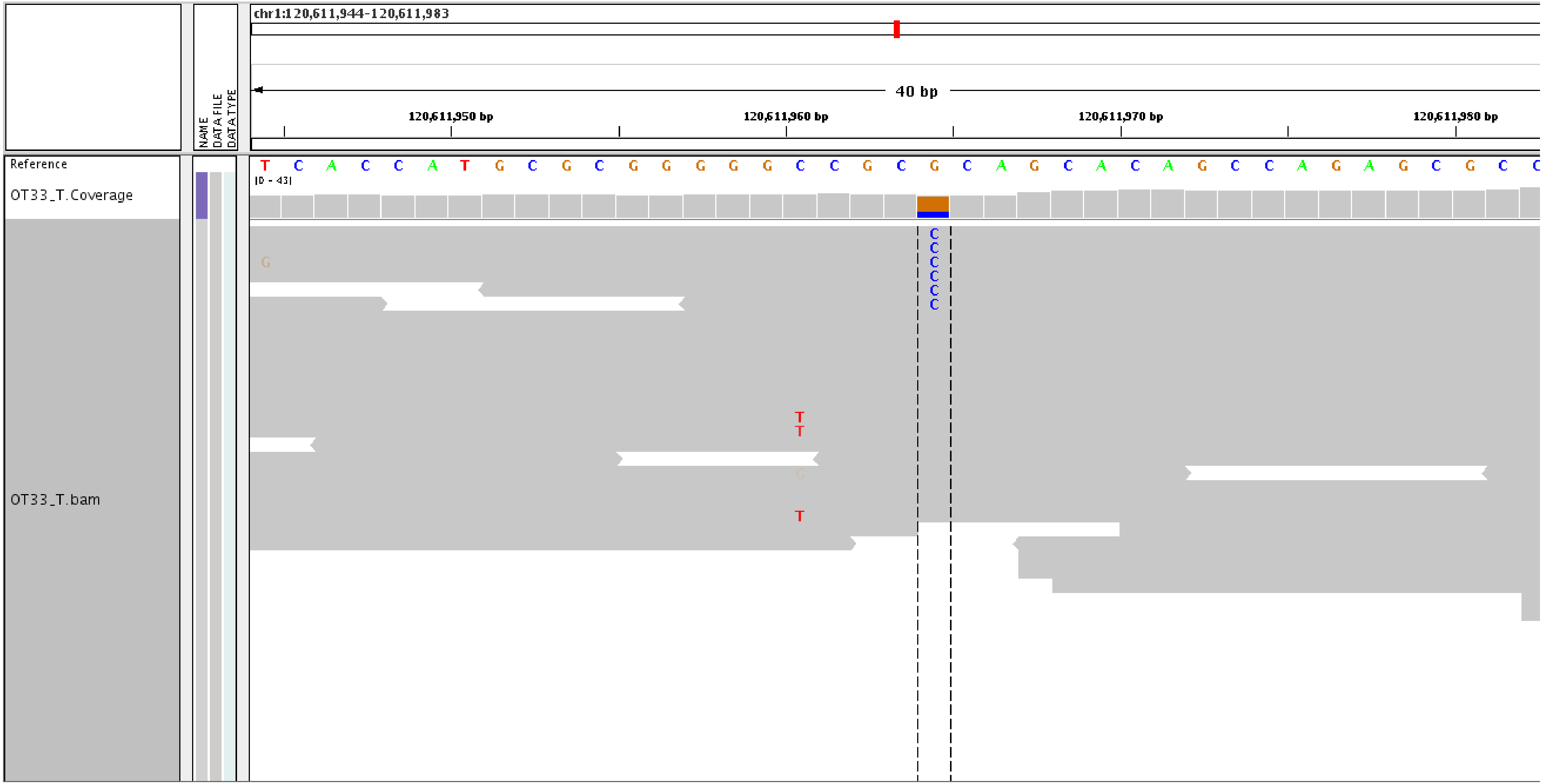

Event: chr17 7577527

Sample: OT37

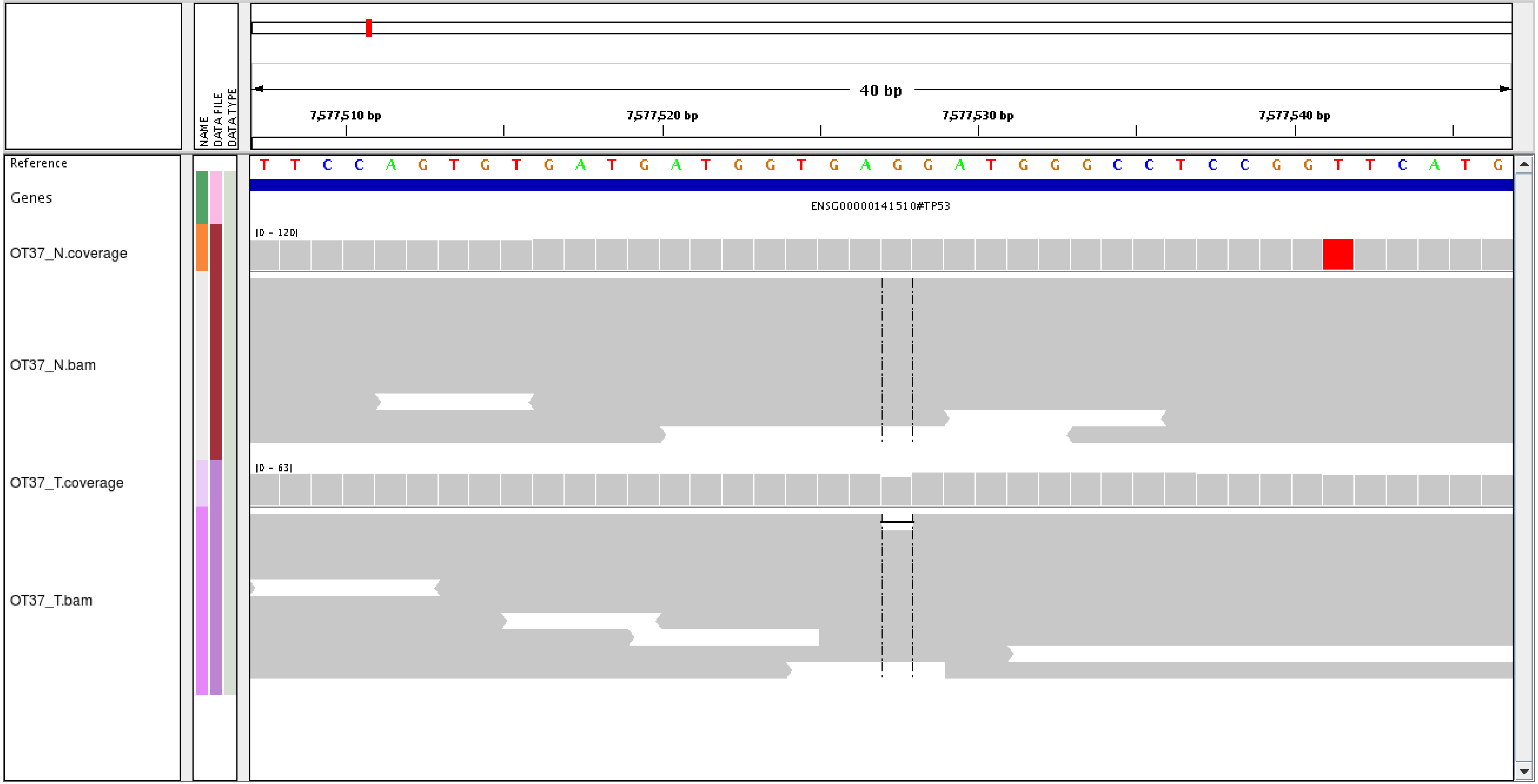

Event: chr9 21971186

Sample: OT38

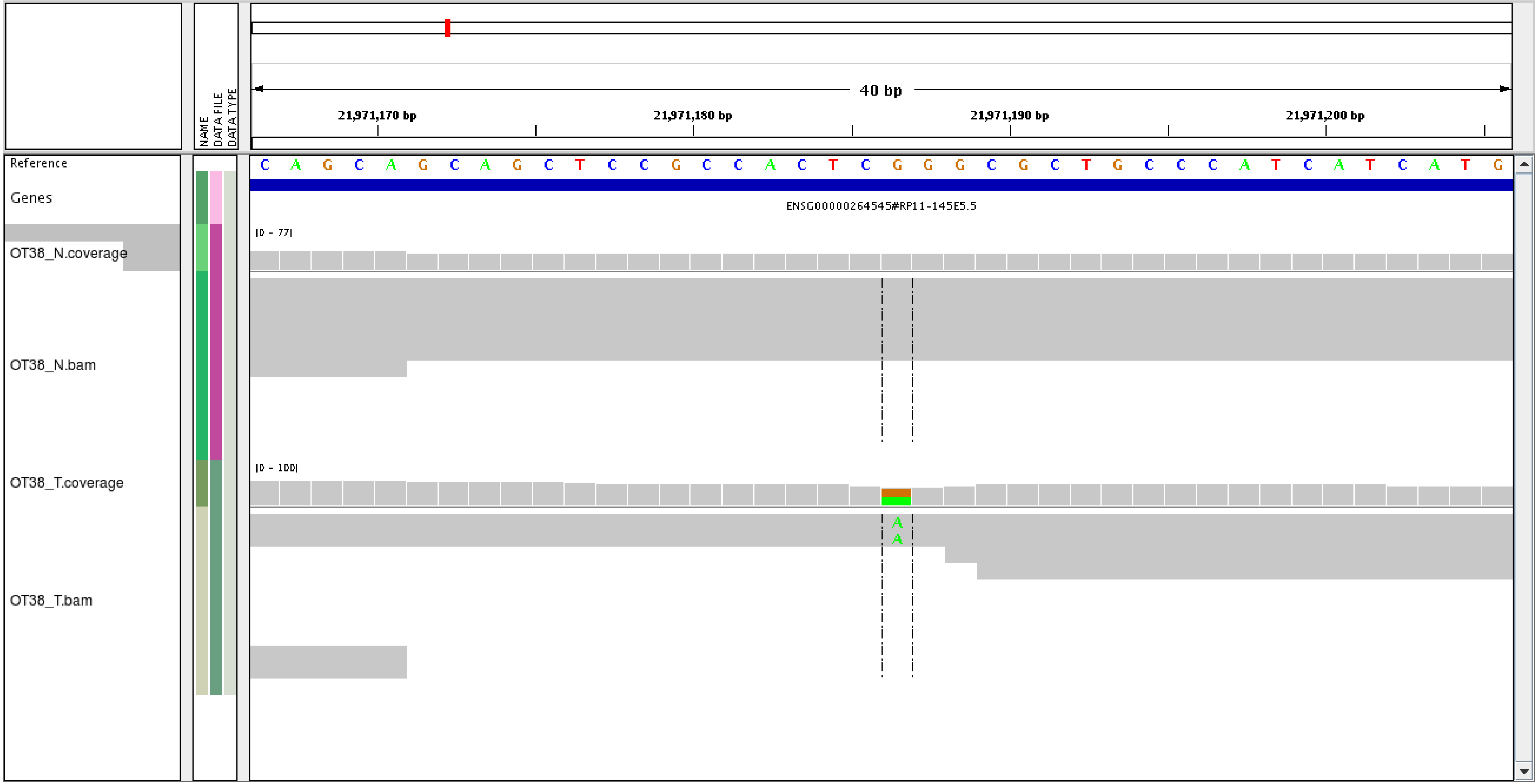

Event: chr1 228506577

Sample: OT41

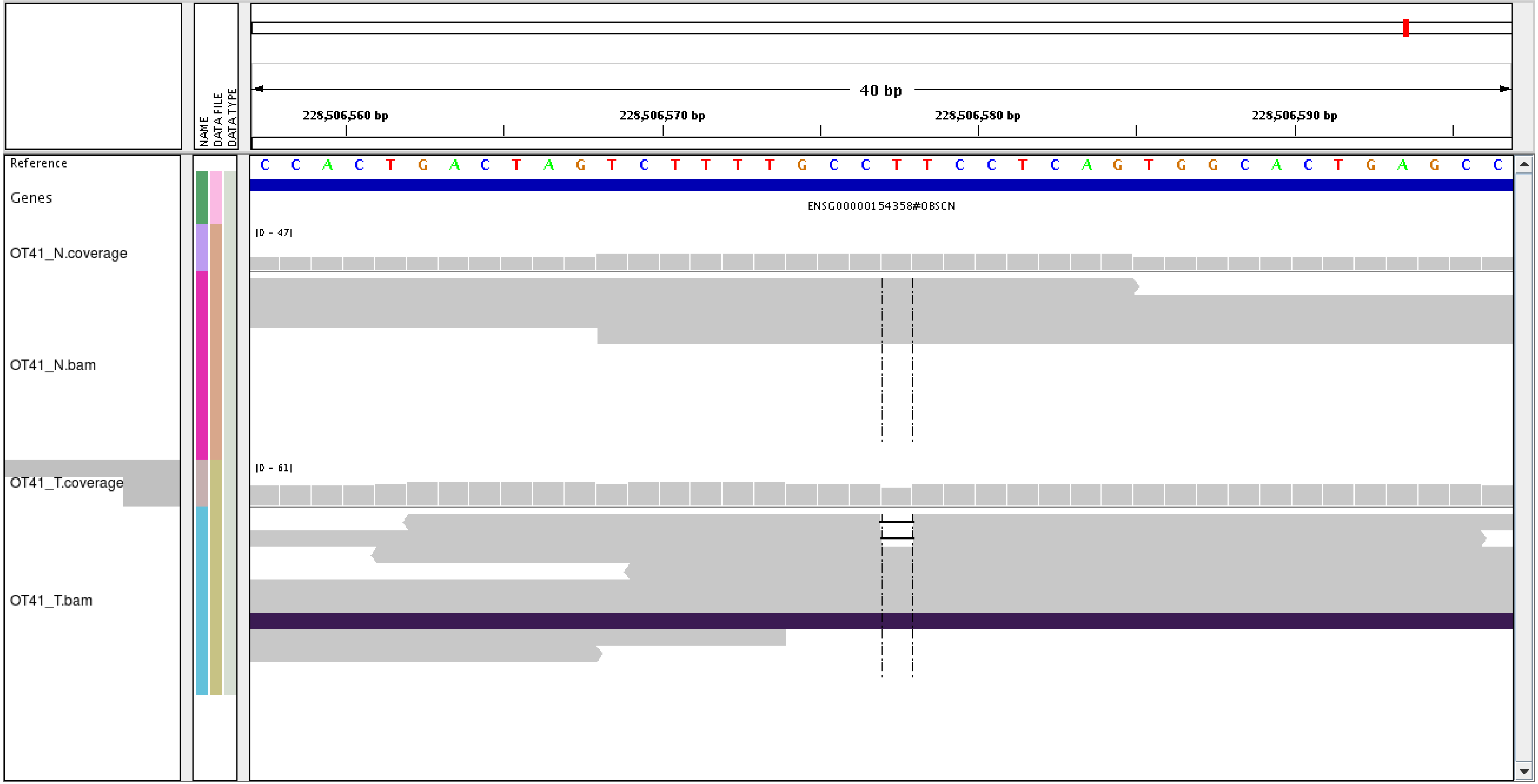

Event: chr5 86648996

Sample: OT42_B

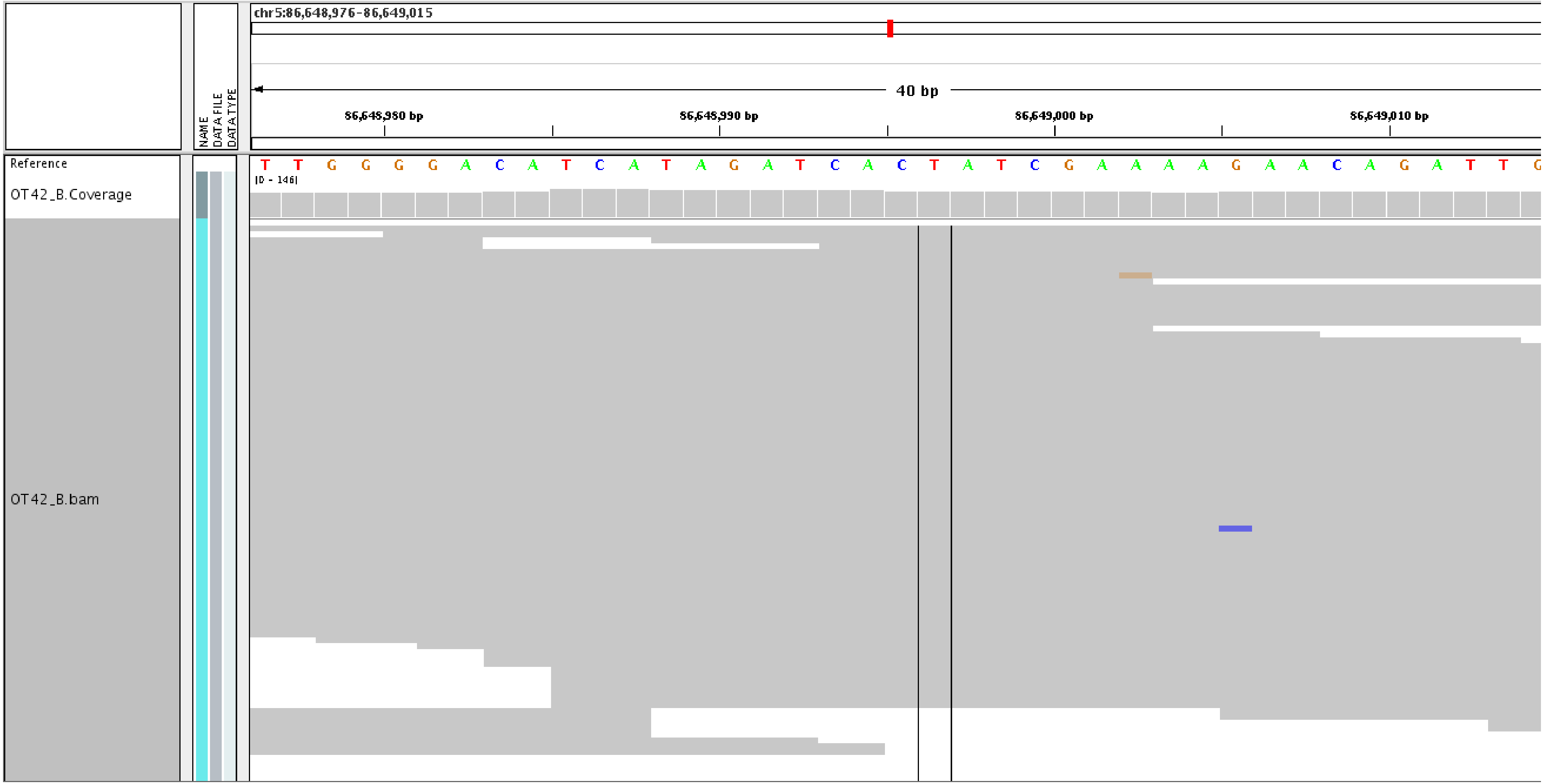

Event: chr5 86648996

Sample: OT42_T

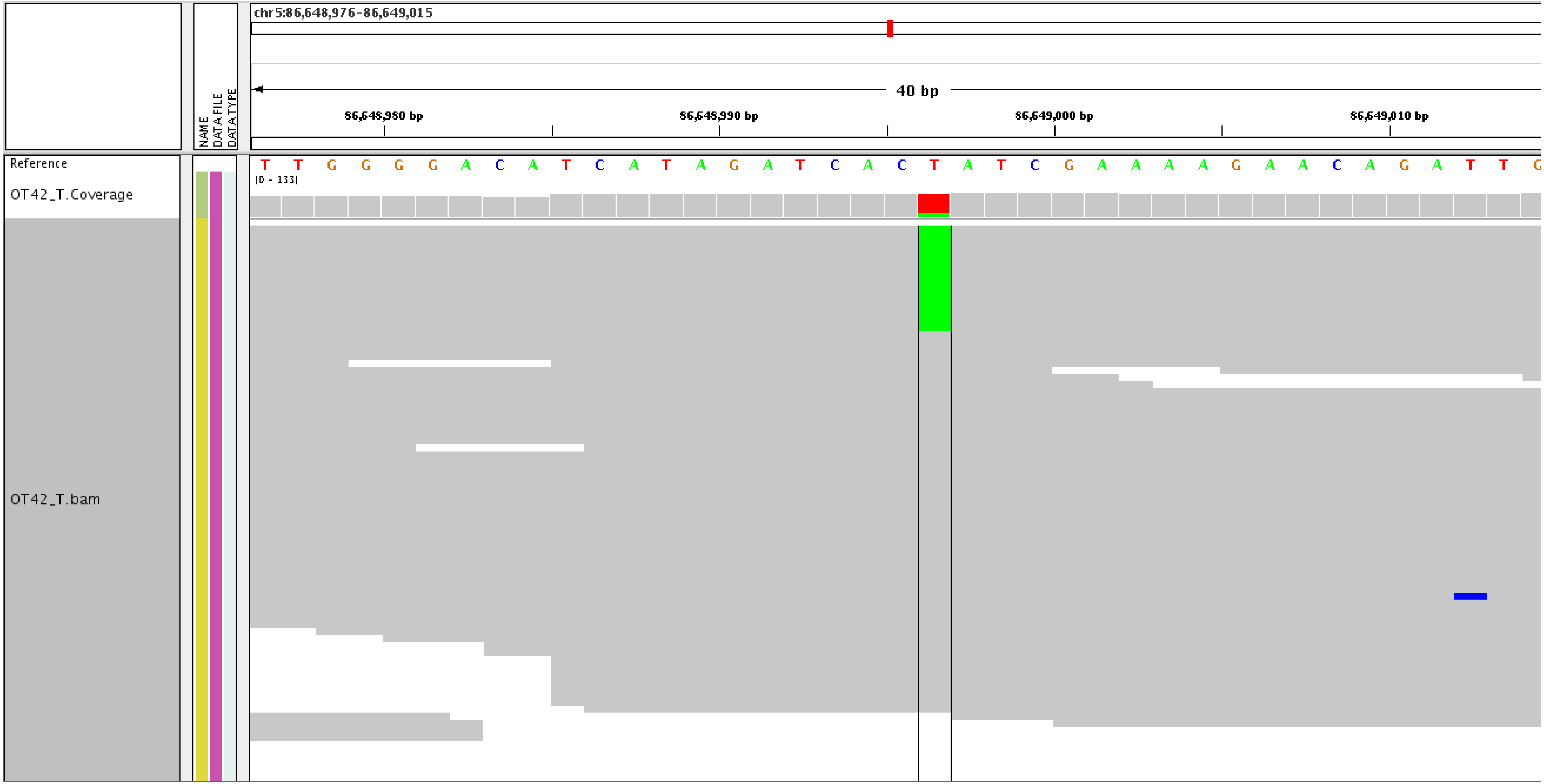

Event: chr13 111366561

Sample: OT42

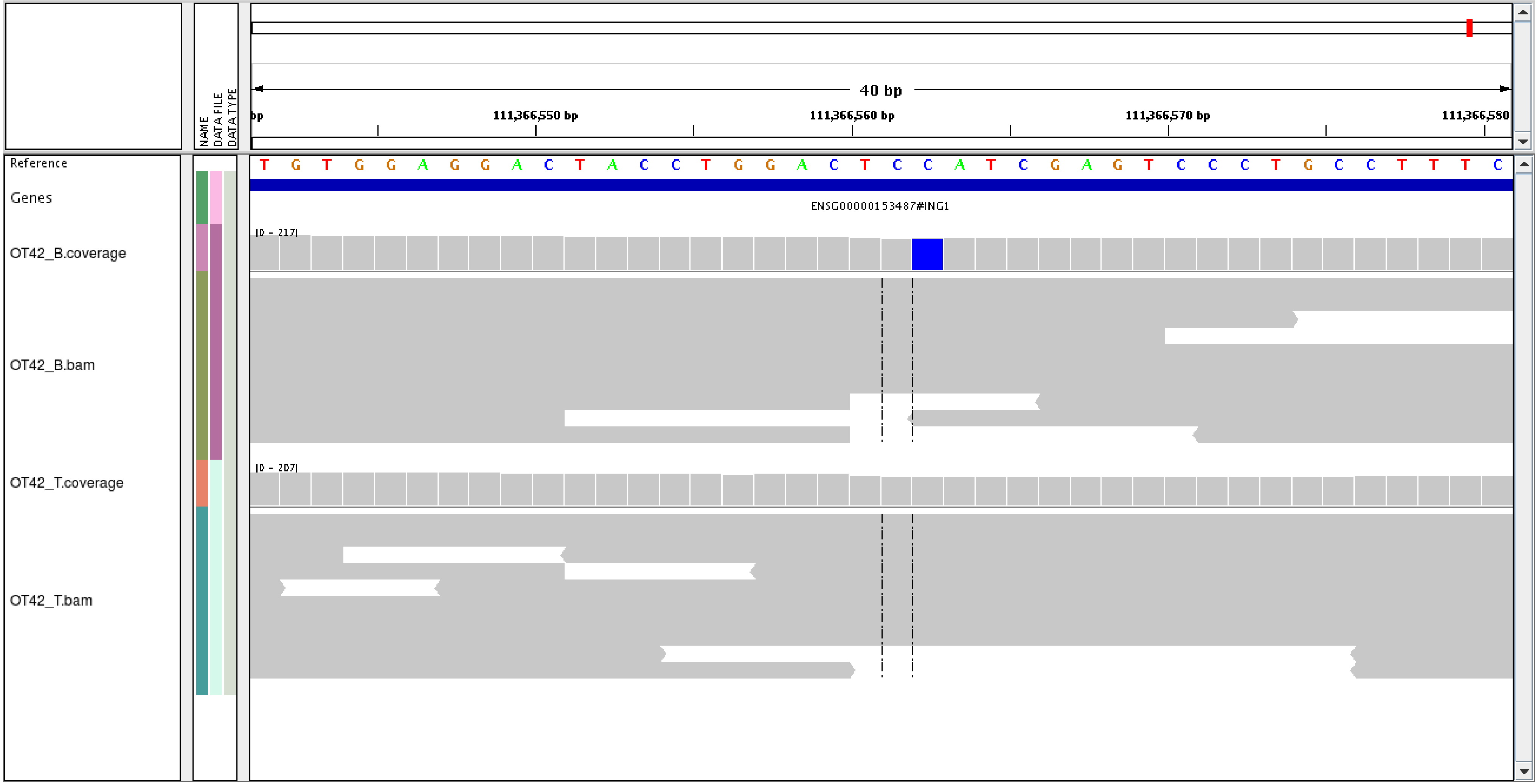

Event: chr17 7574017

Sample: OT42

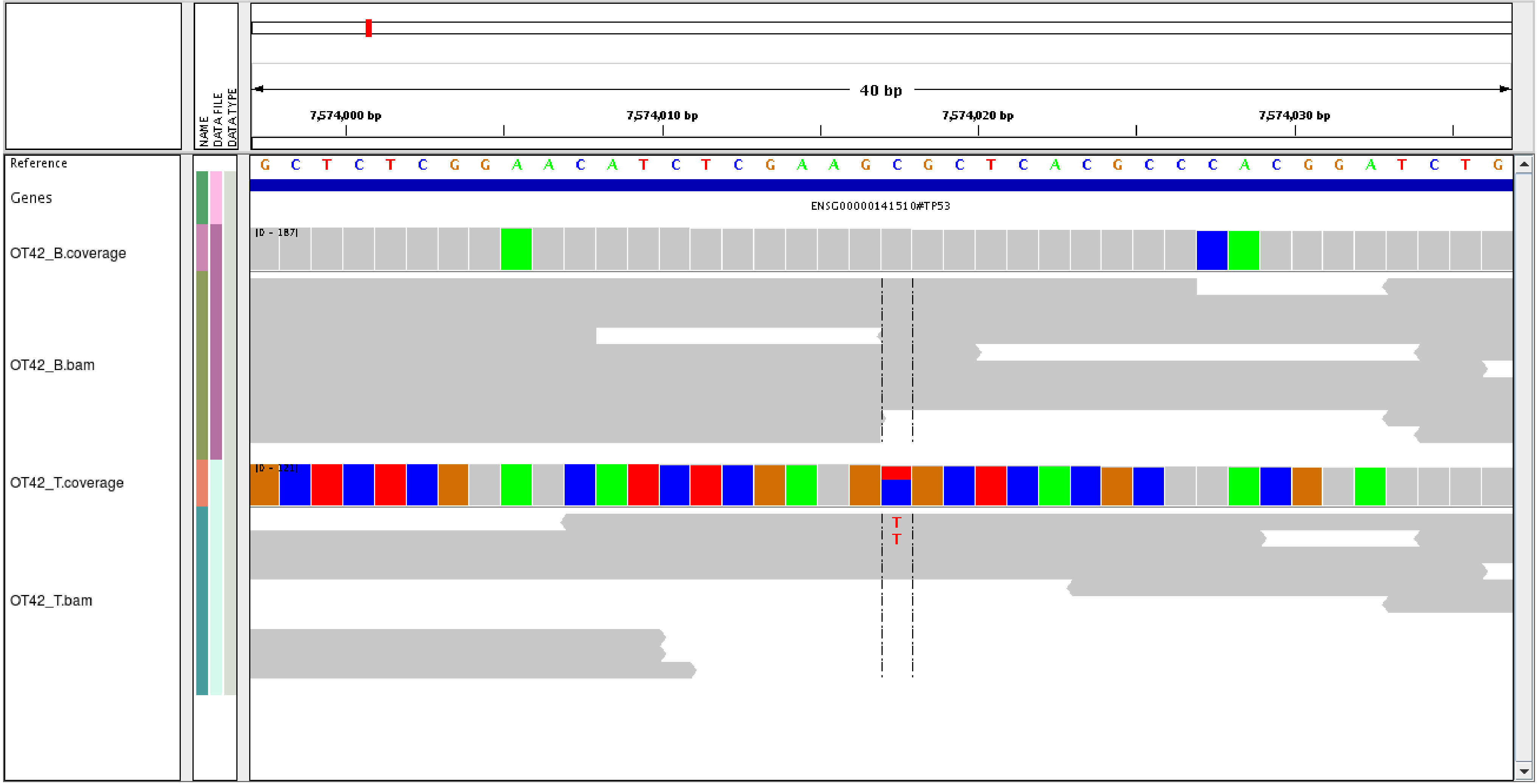

Event: chr19 10602439

Sample: OT42

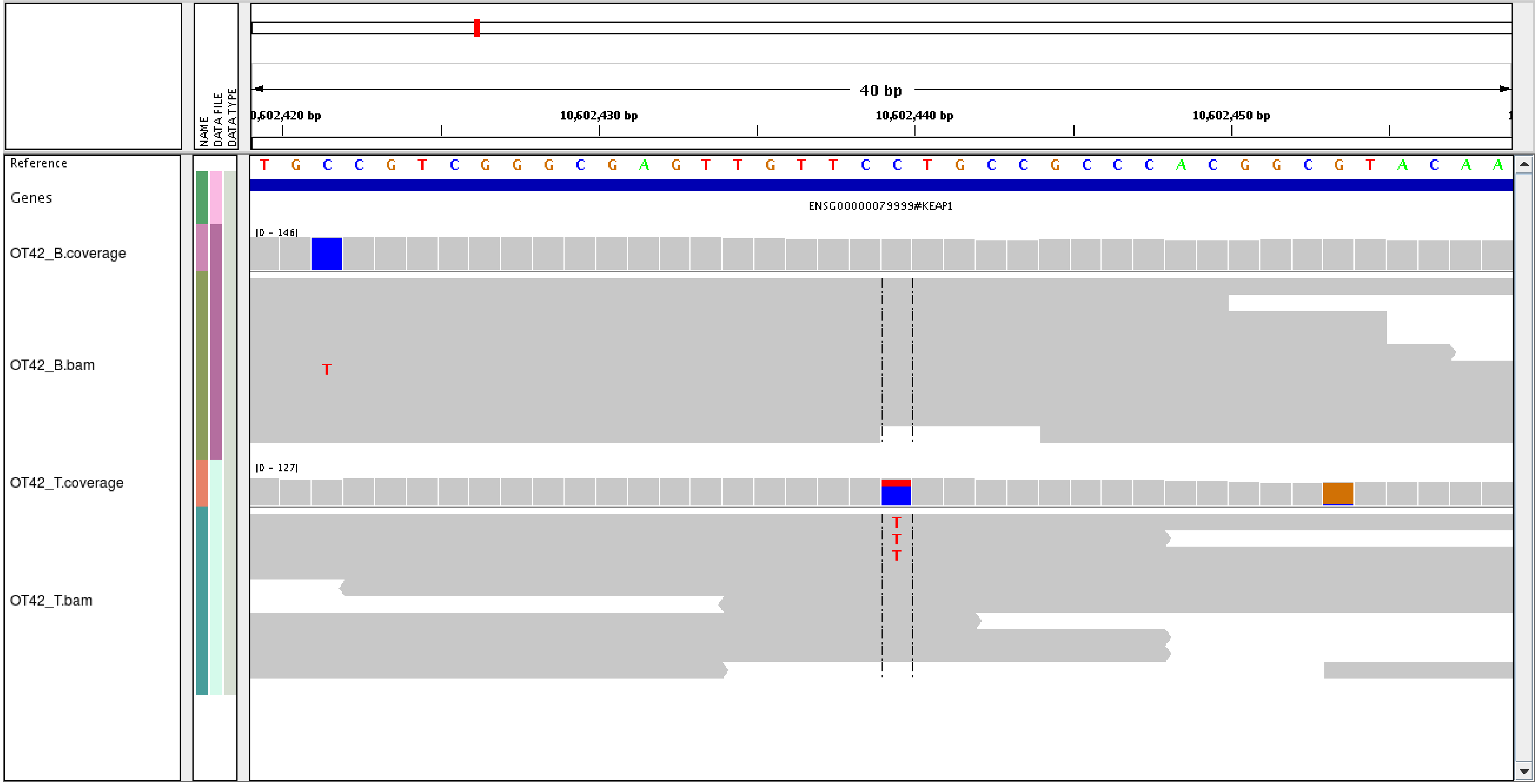

Event: chr21 44513282

Sample: OT43

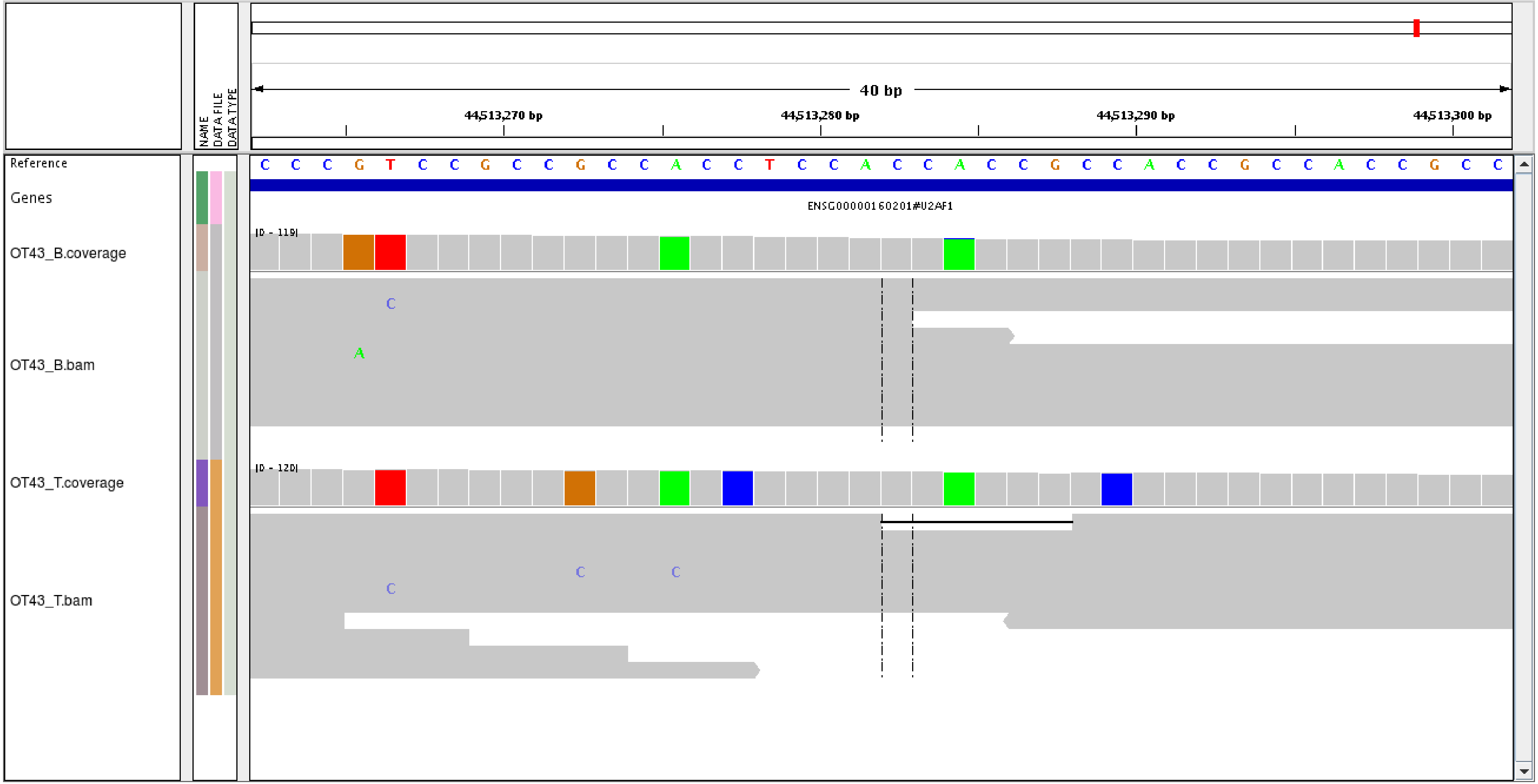

Event: chrX 31676140

Sample: OT43

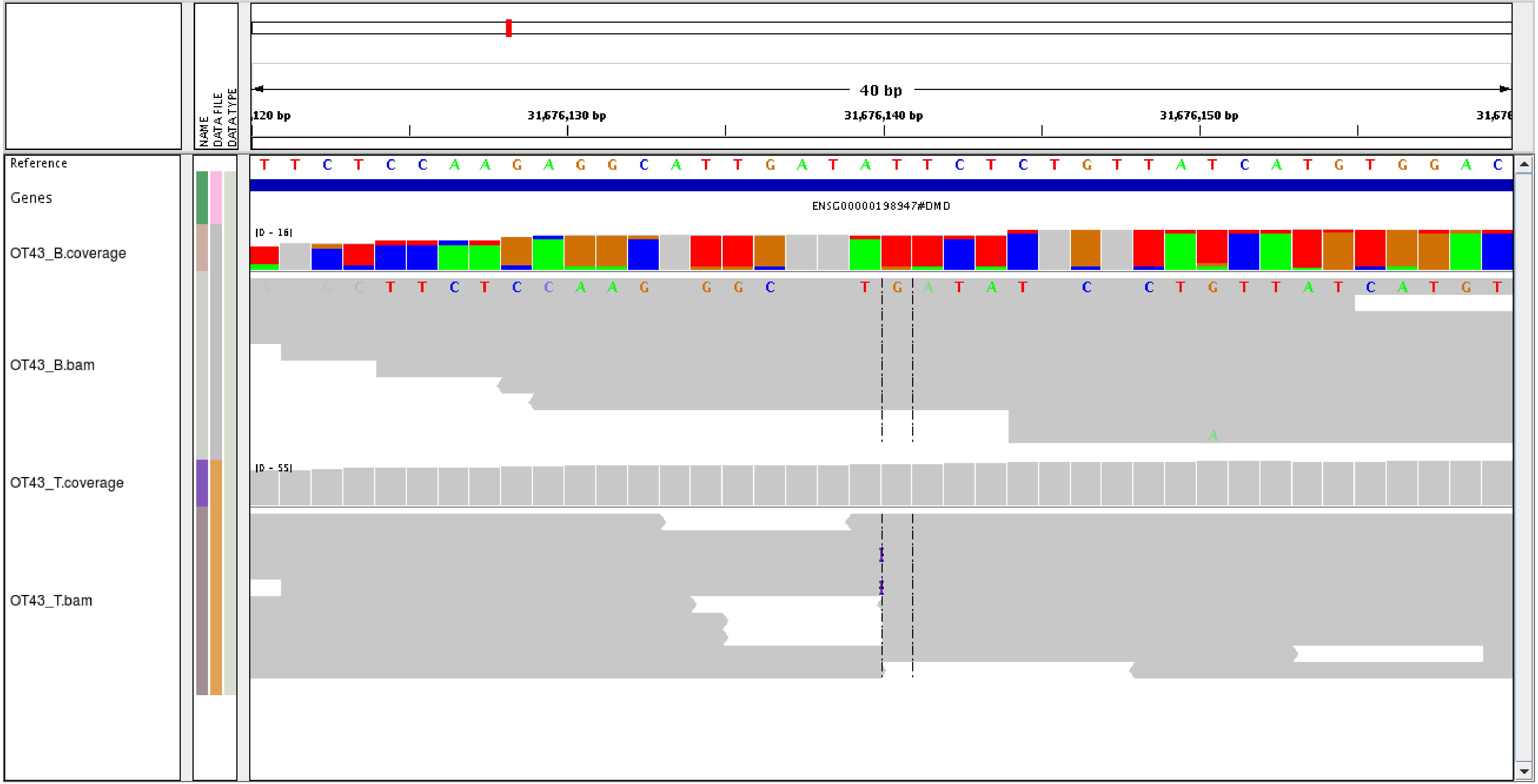

Event: chr2 202150039

Sample: OT44

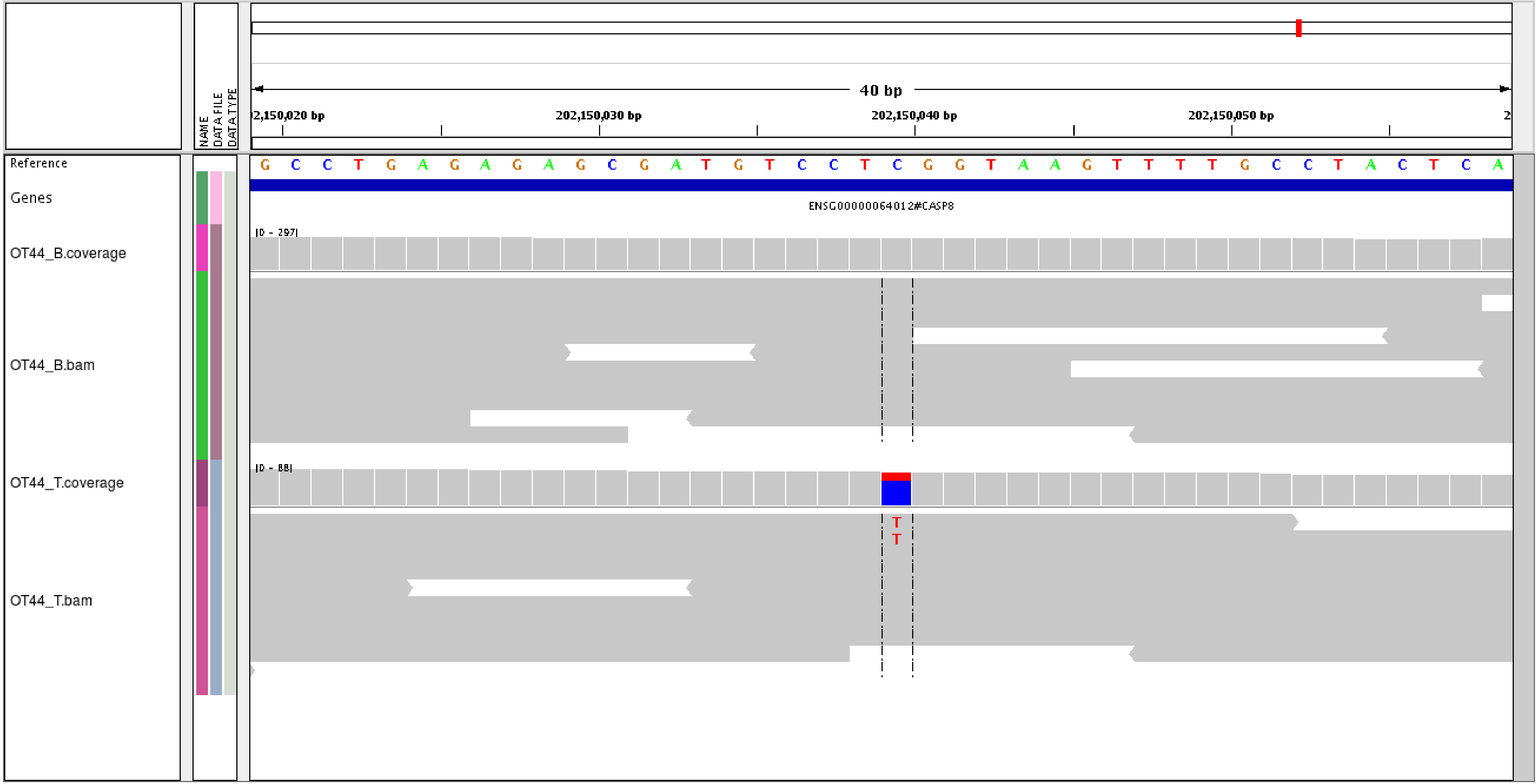

Event: chr4 187532672

Sample: OT44_B

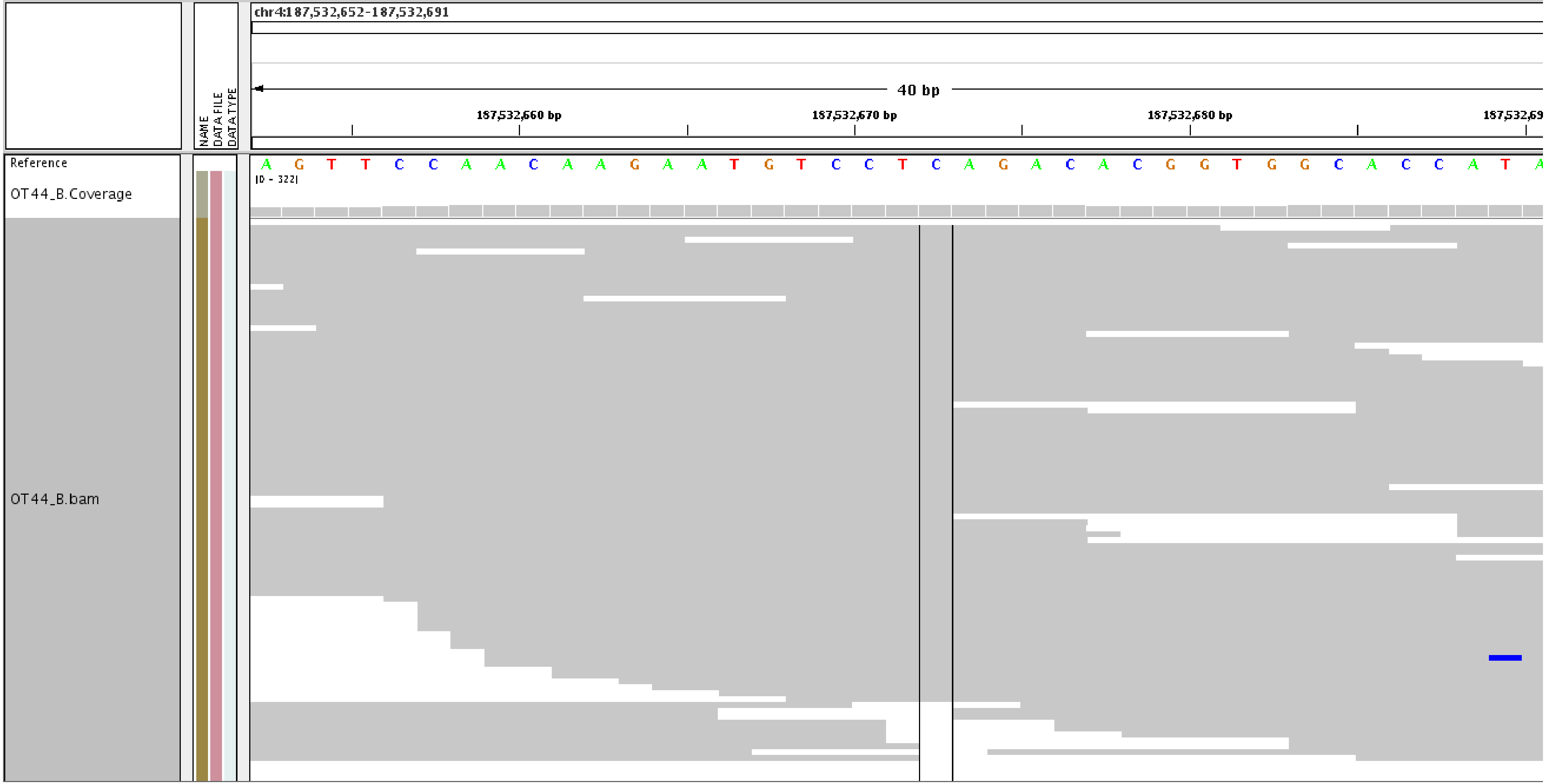

Event: chr4 187532672

Sample: OT44_T

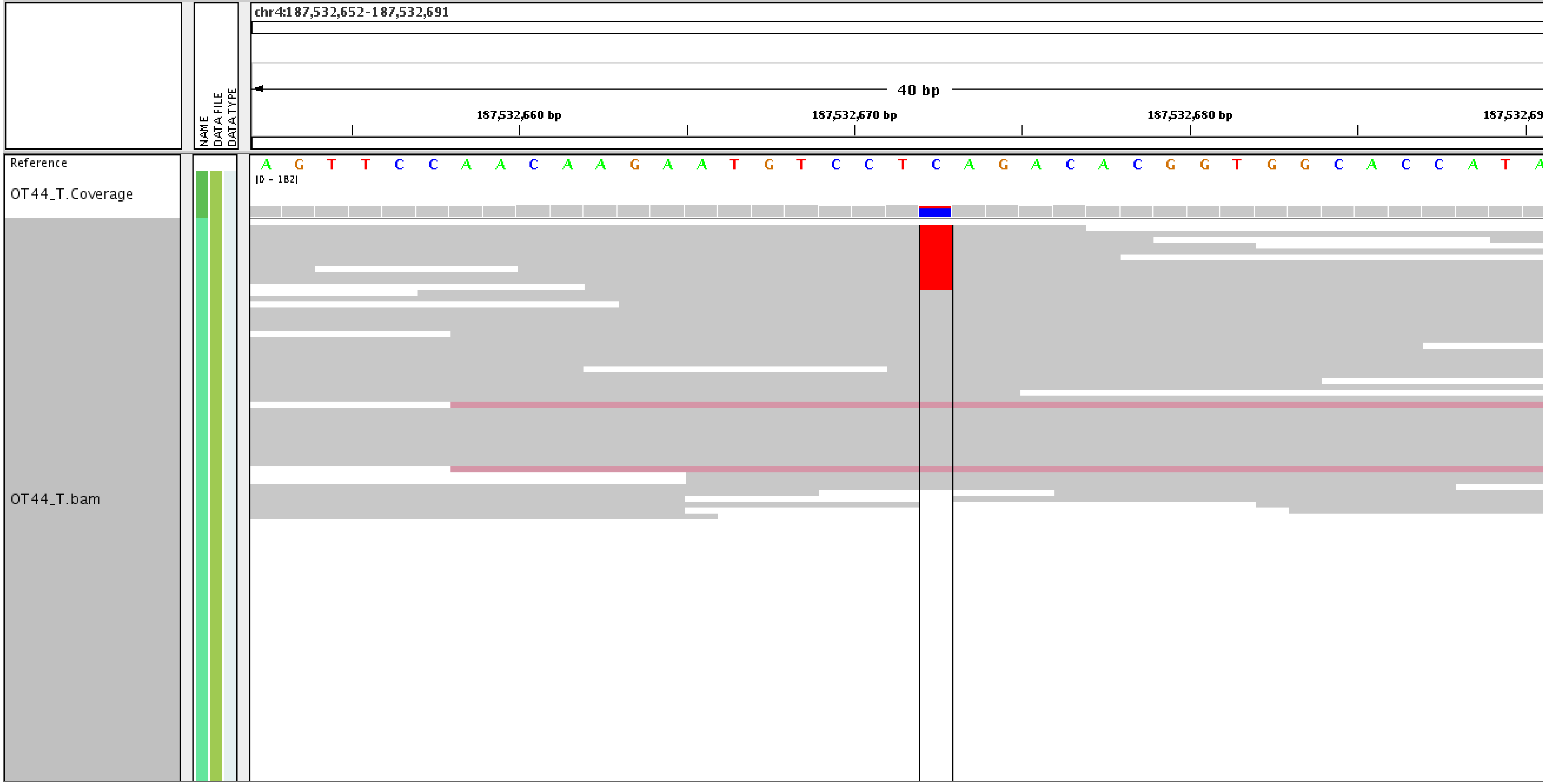

Event: chr4 187539951

Sample: OT44_B

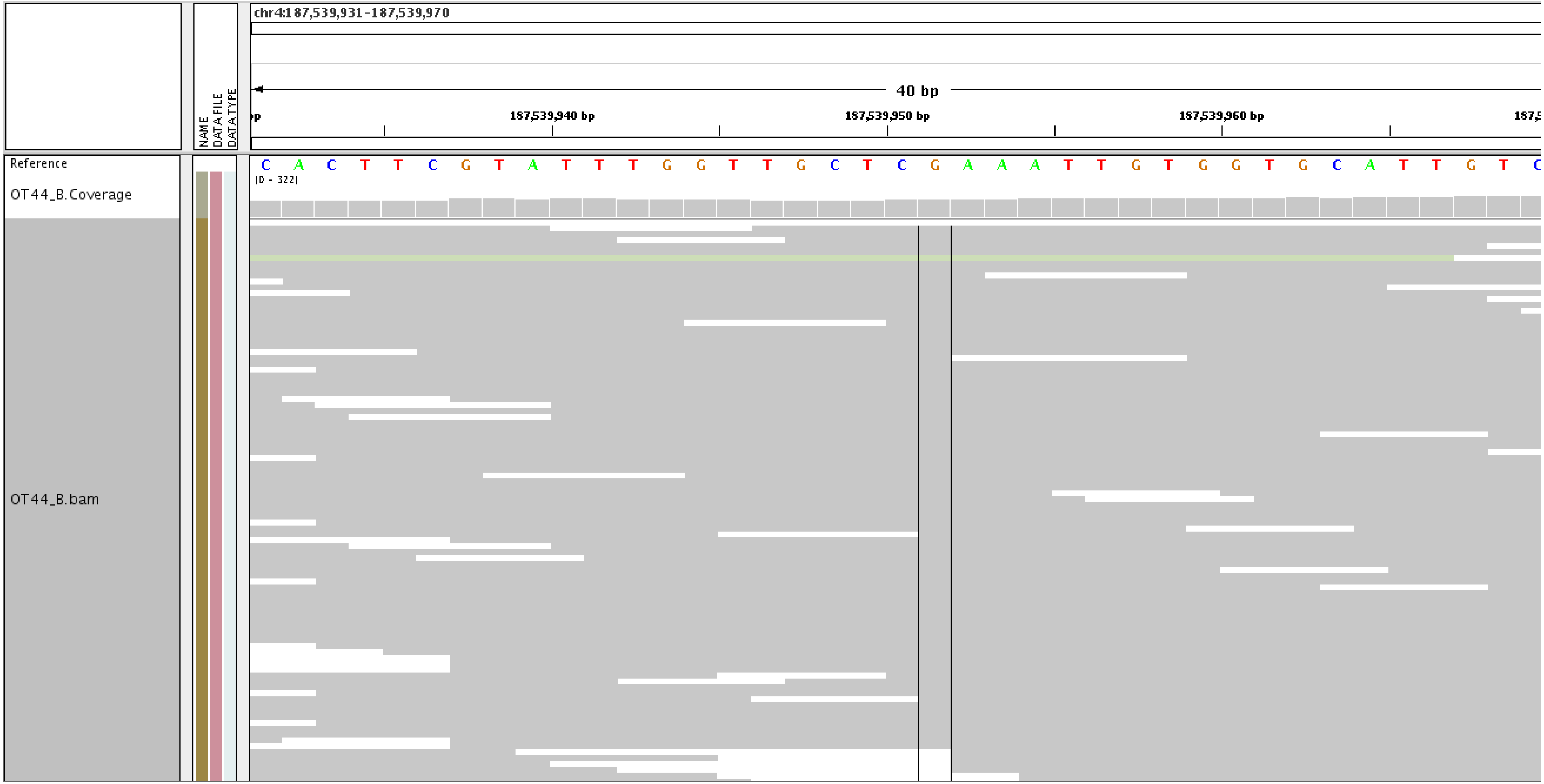

Event: chr4 187539951

Sample: OT44_T

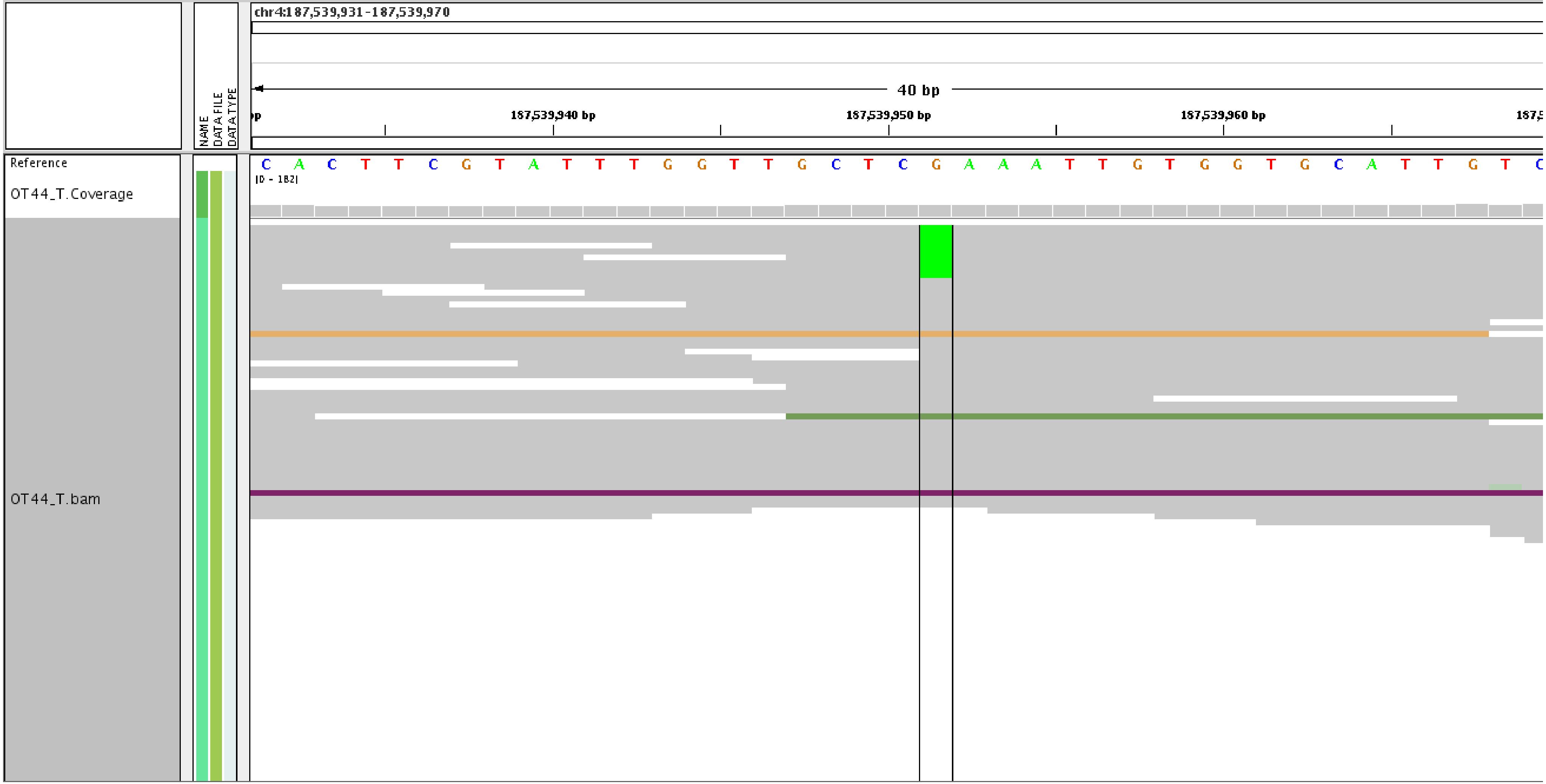

Event: chr5 86681150

Sample: OT44_B

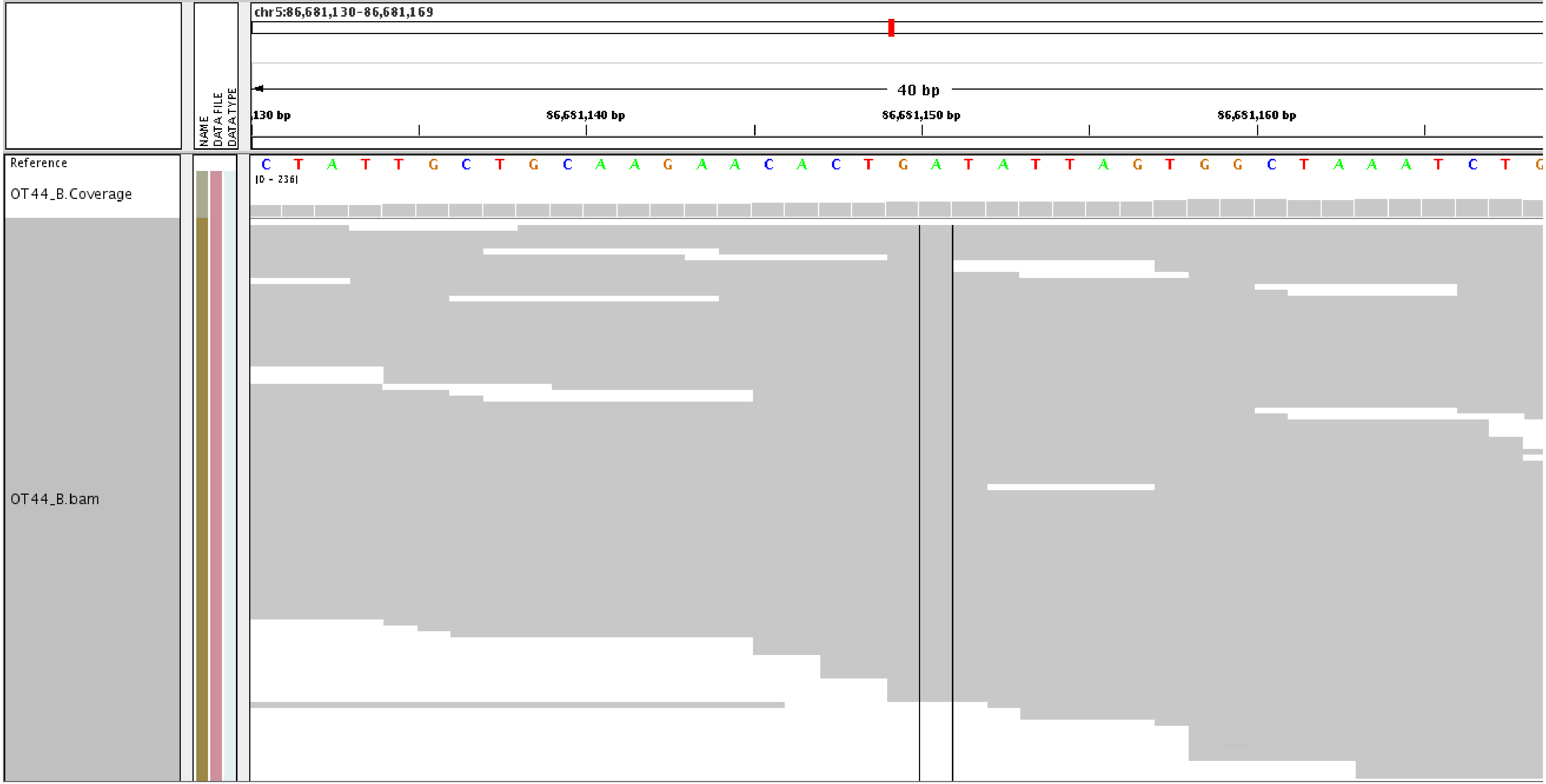

Event: chr5 86681150

Sample: OT44_T

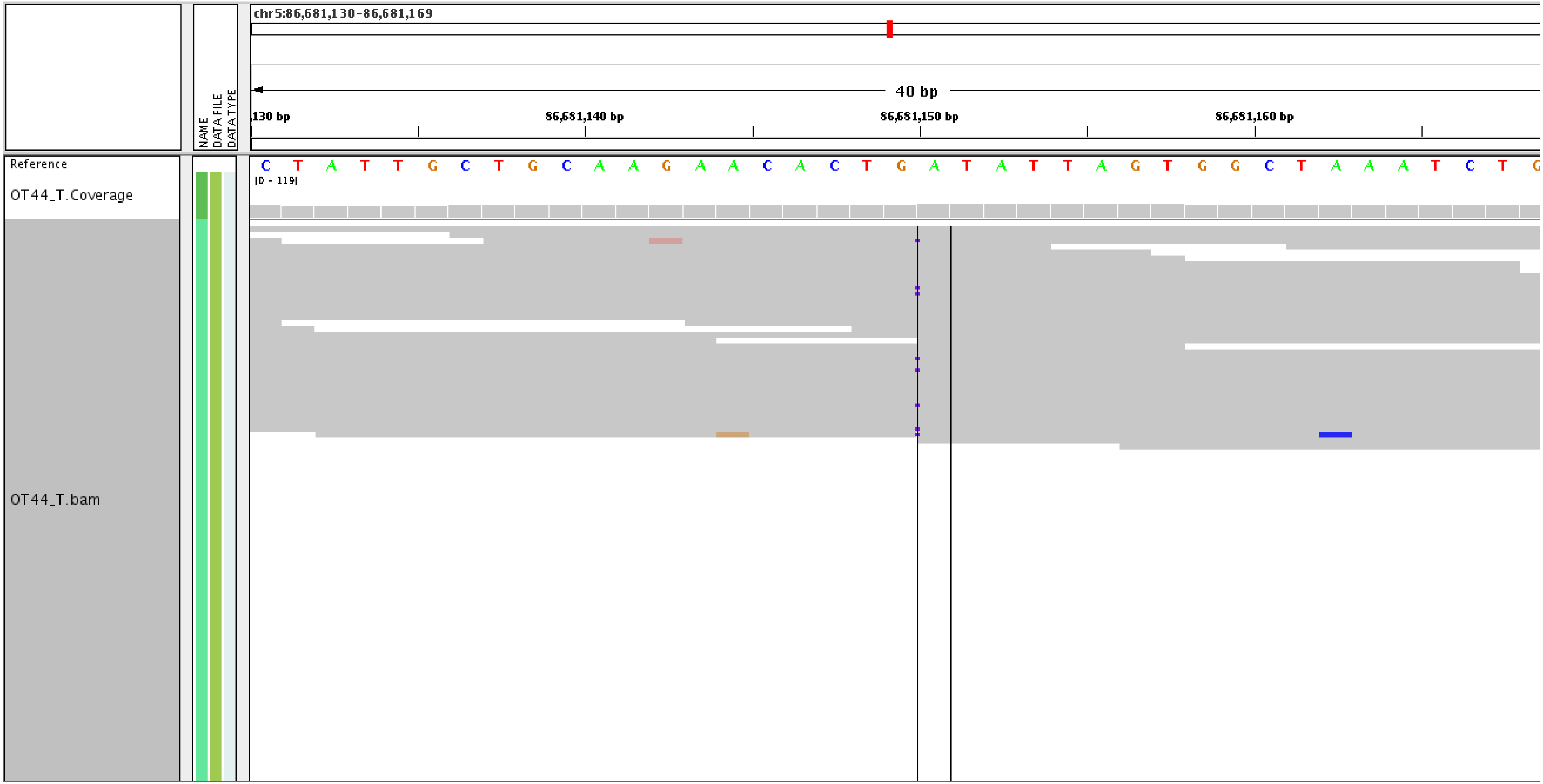

Event: chr17 7578532

Sample: OT44_B

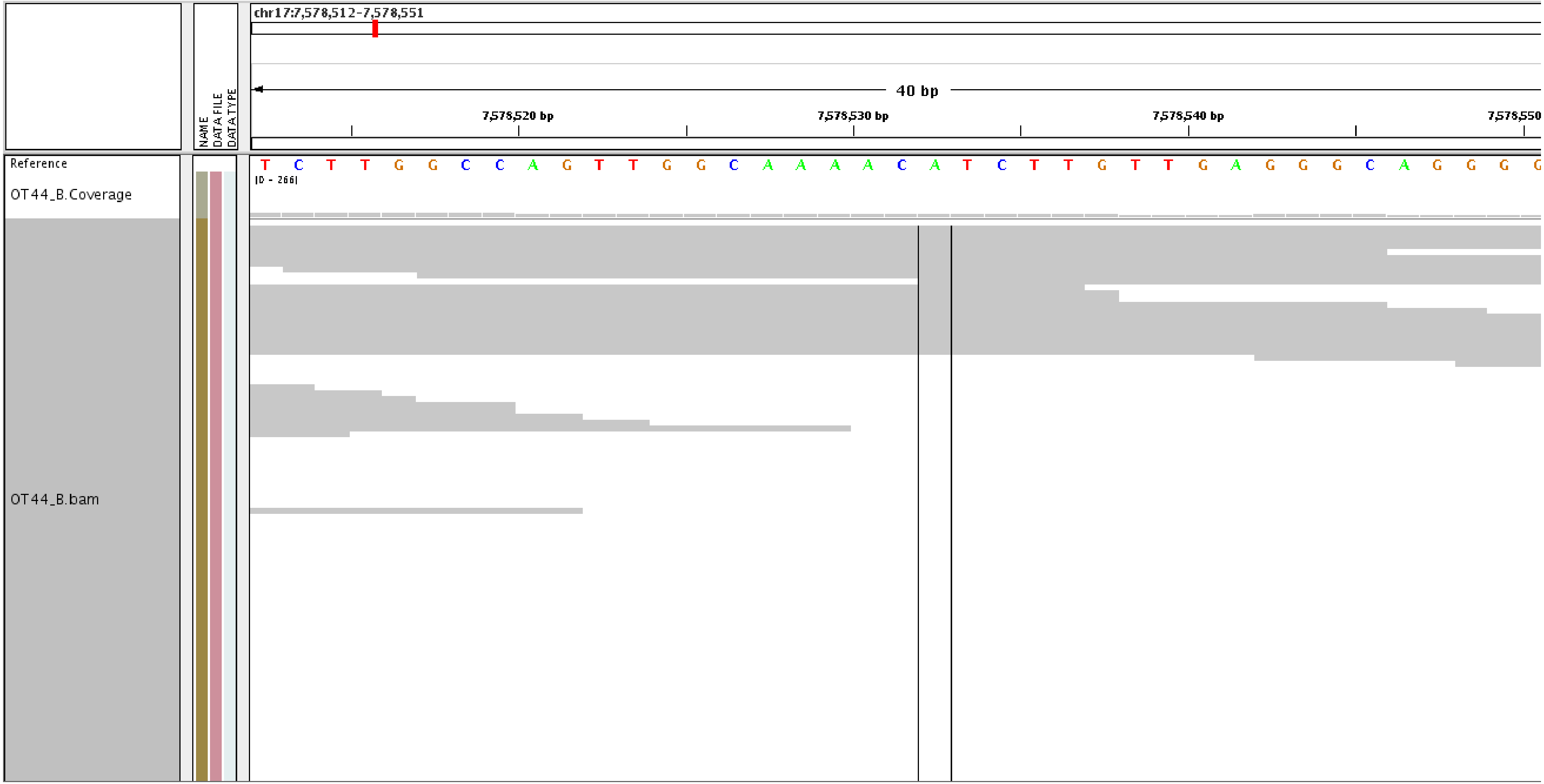

Event: chr17 7578532

Sample: OT44_T

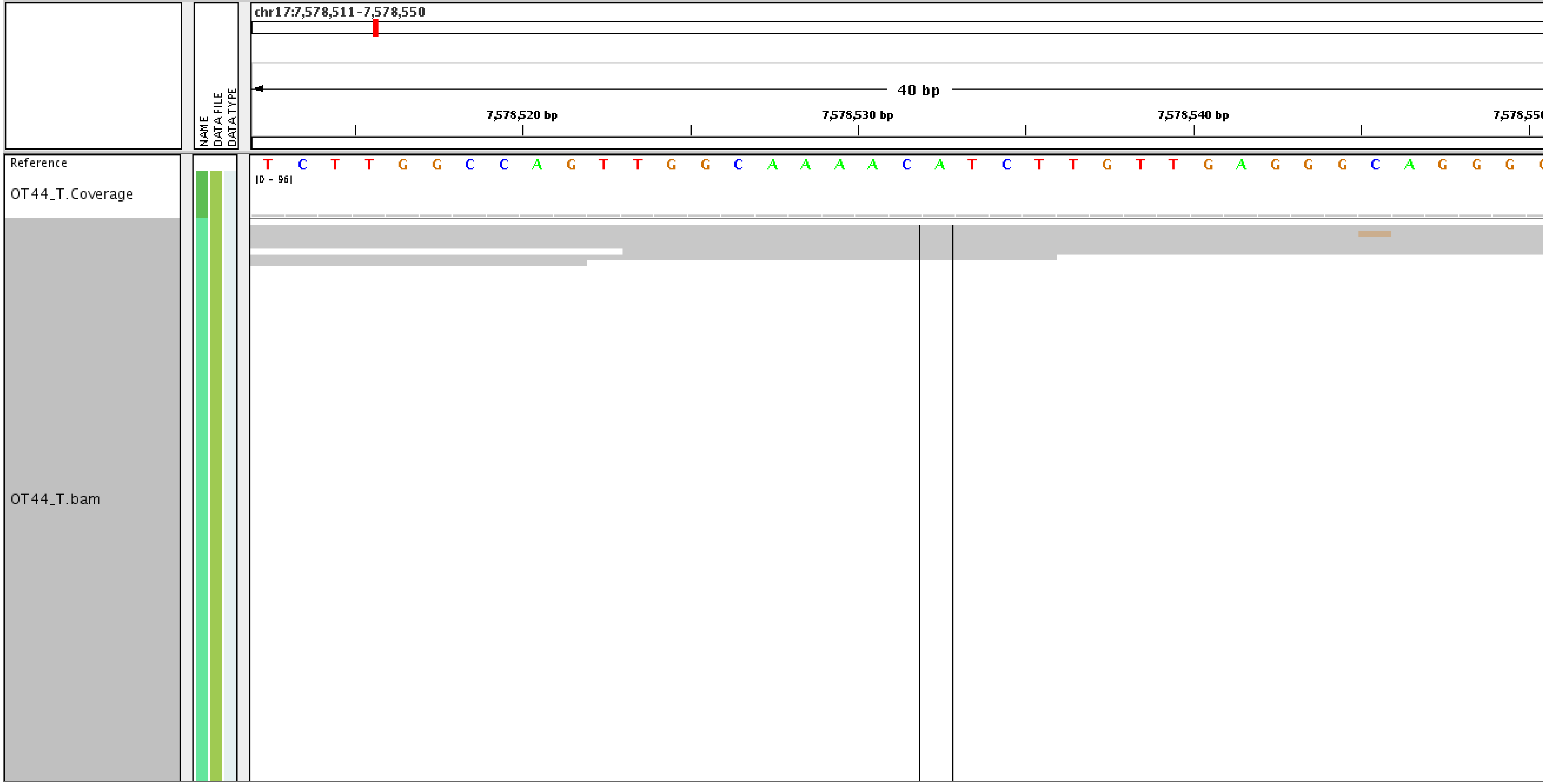

Event: chr14 23450620

Sample: OT46_B

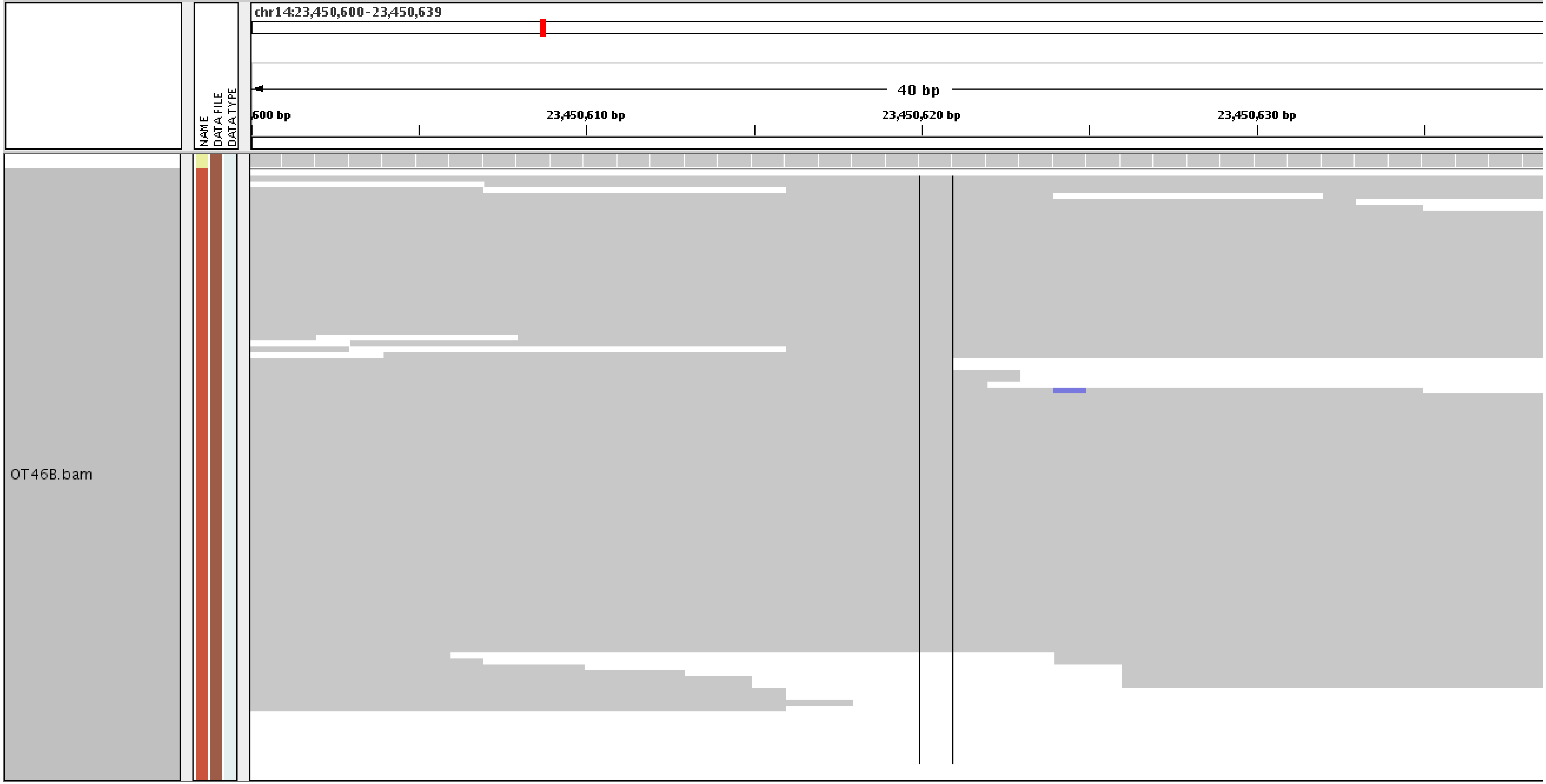

Event: chr14 23450620

Sample: OT46_T

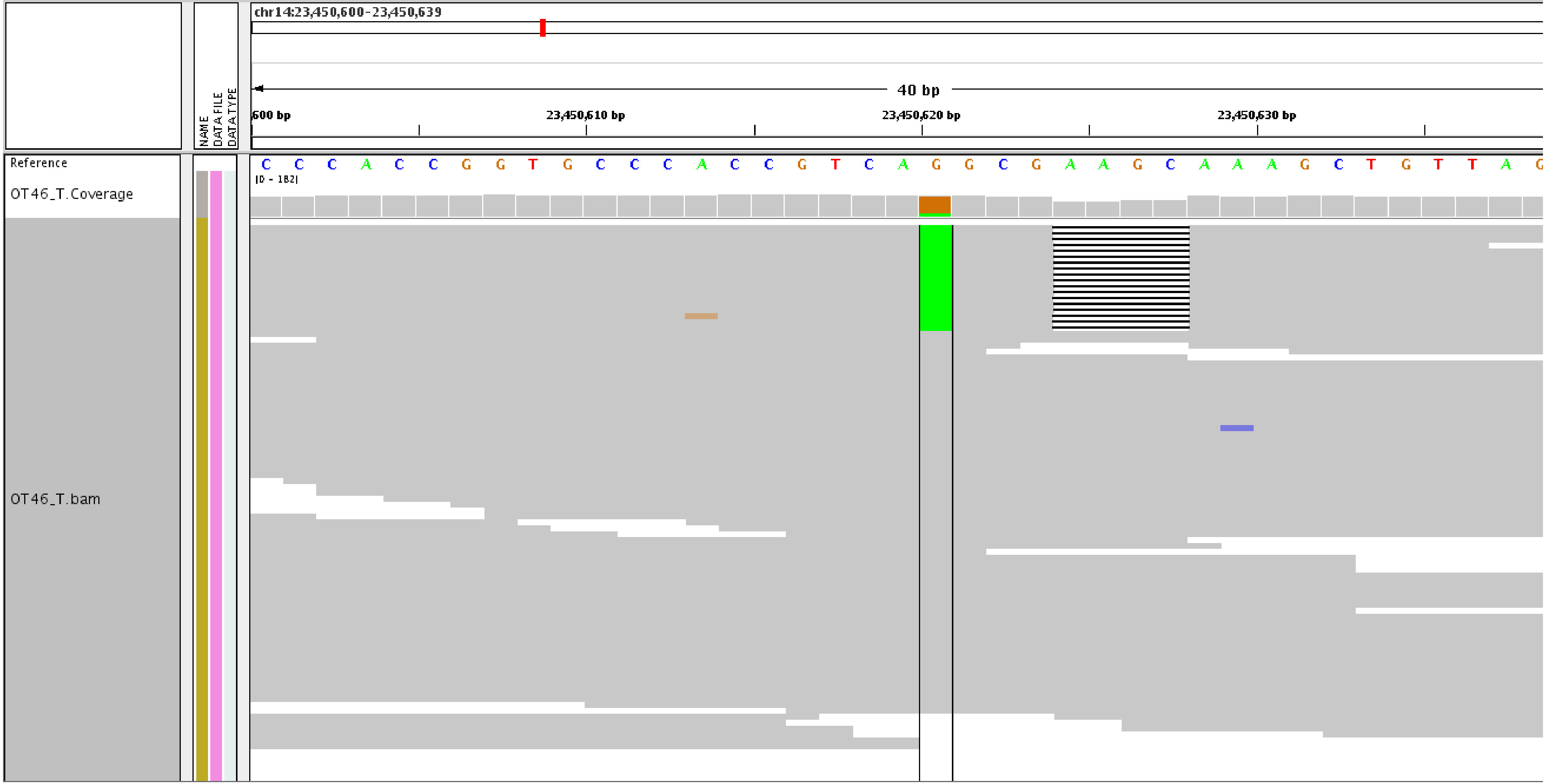

Event: chr14 23450624

Sample: OT46

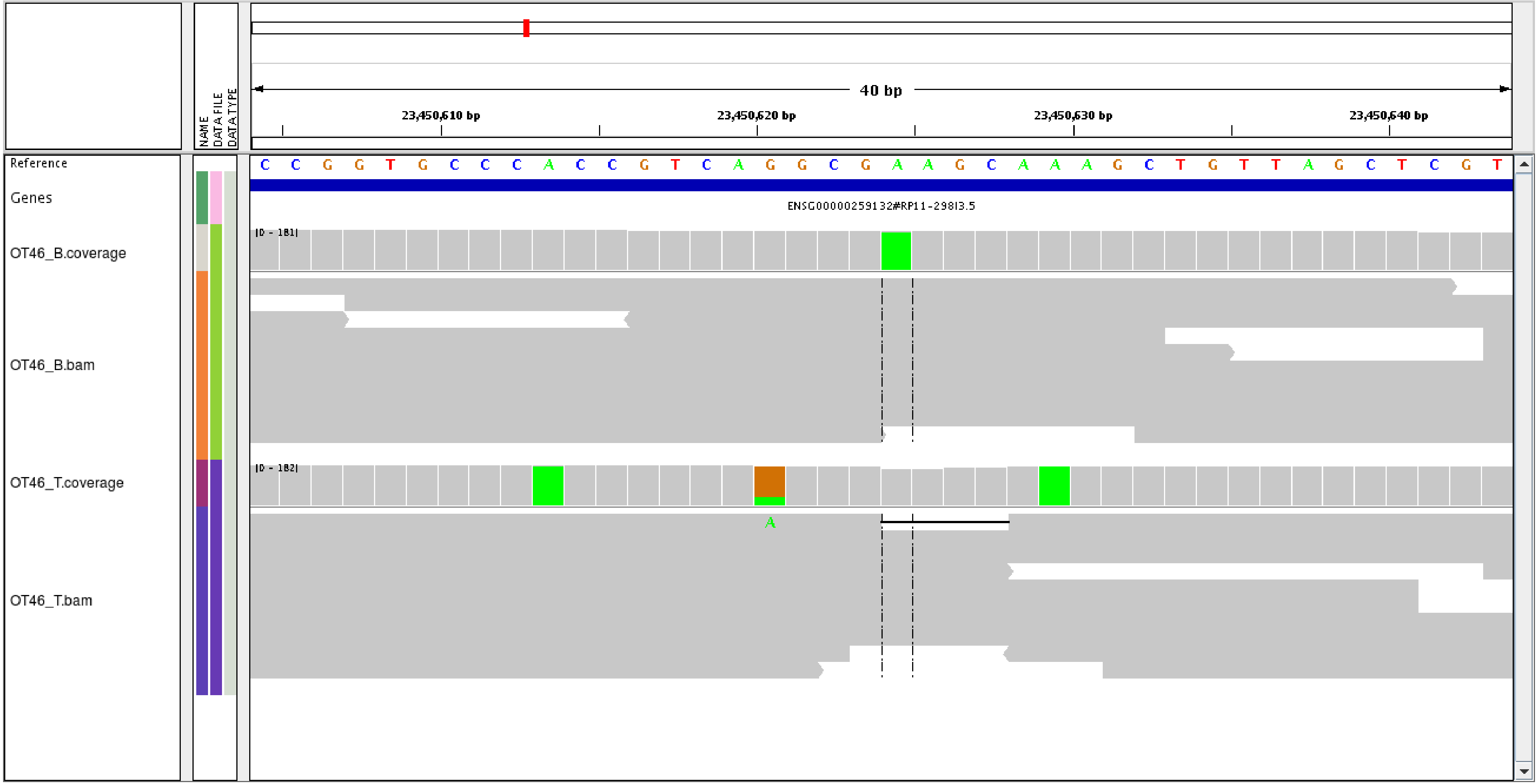

Event: chr17 7577532

Sample: OT46

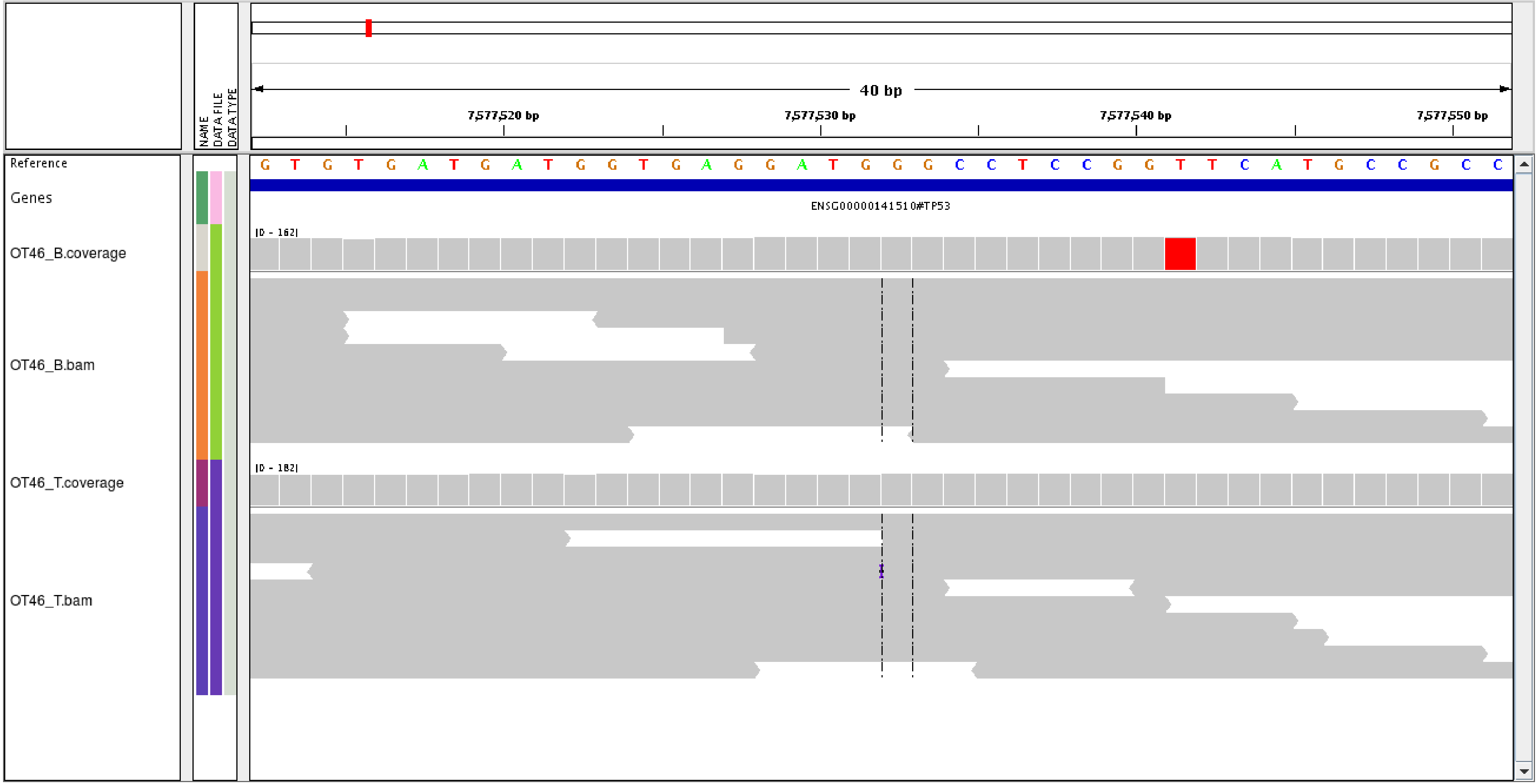

Event: chr6 29911239

Sample: OT49_B

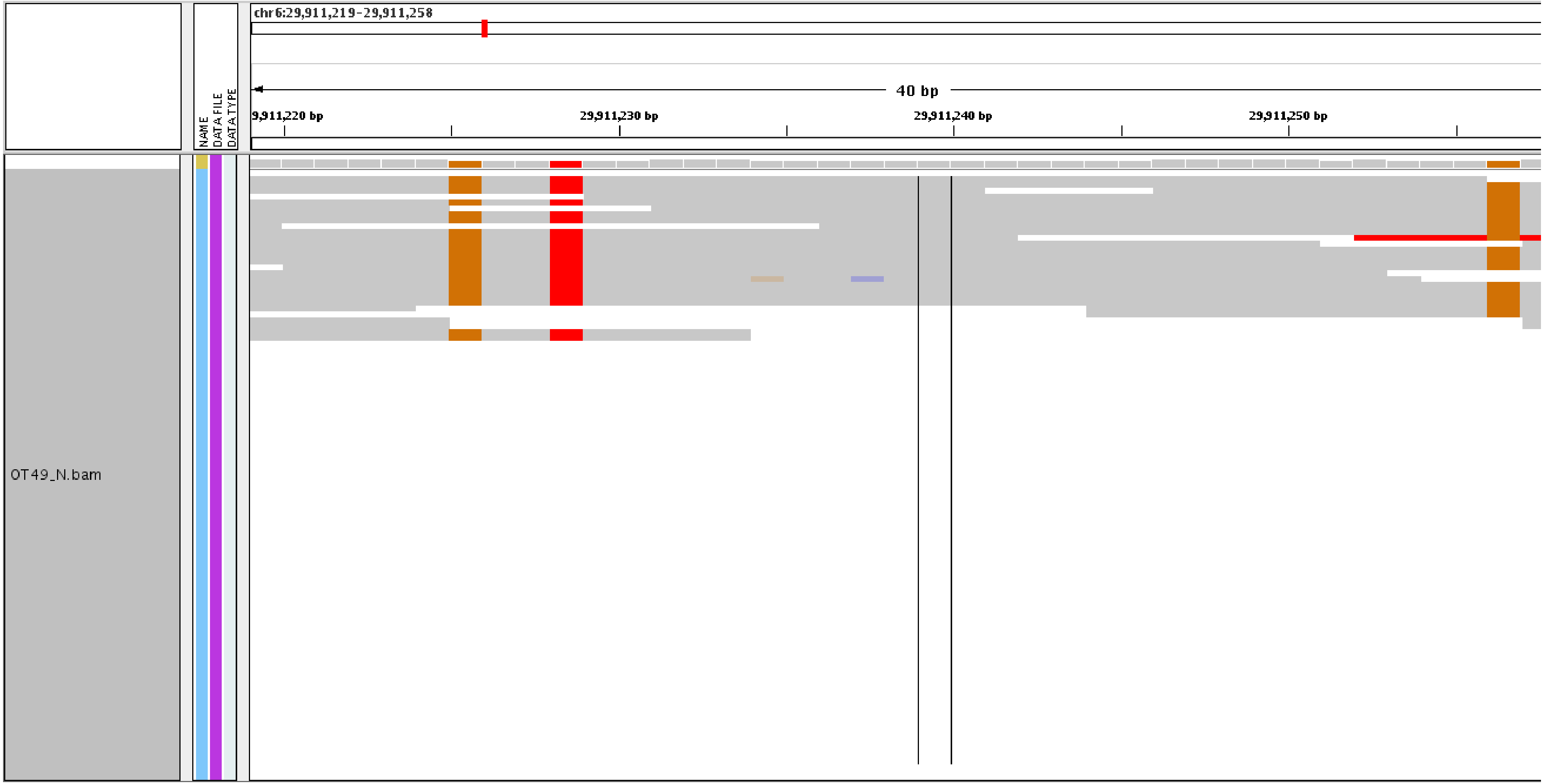

Event: chr6 29911239

Sample: OT49_T

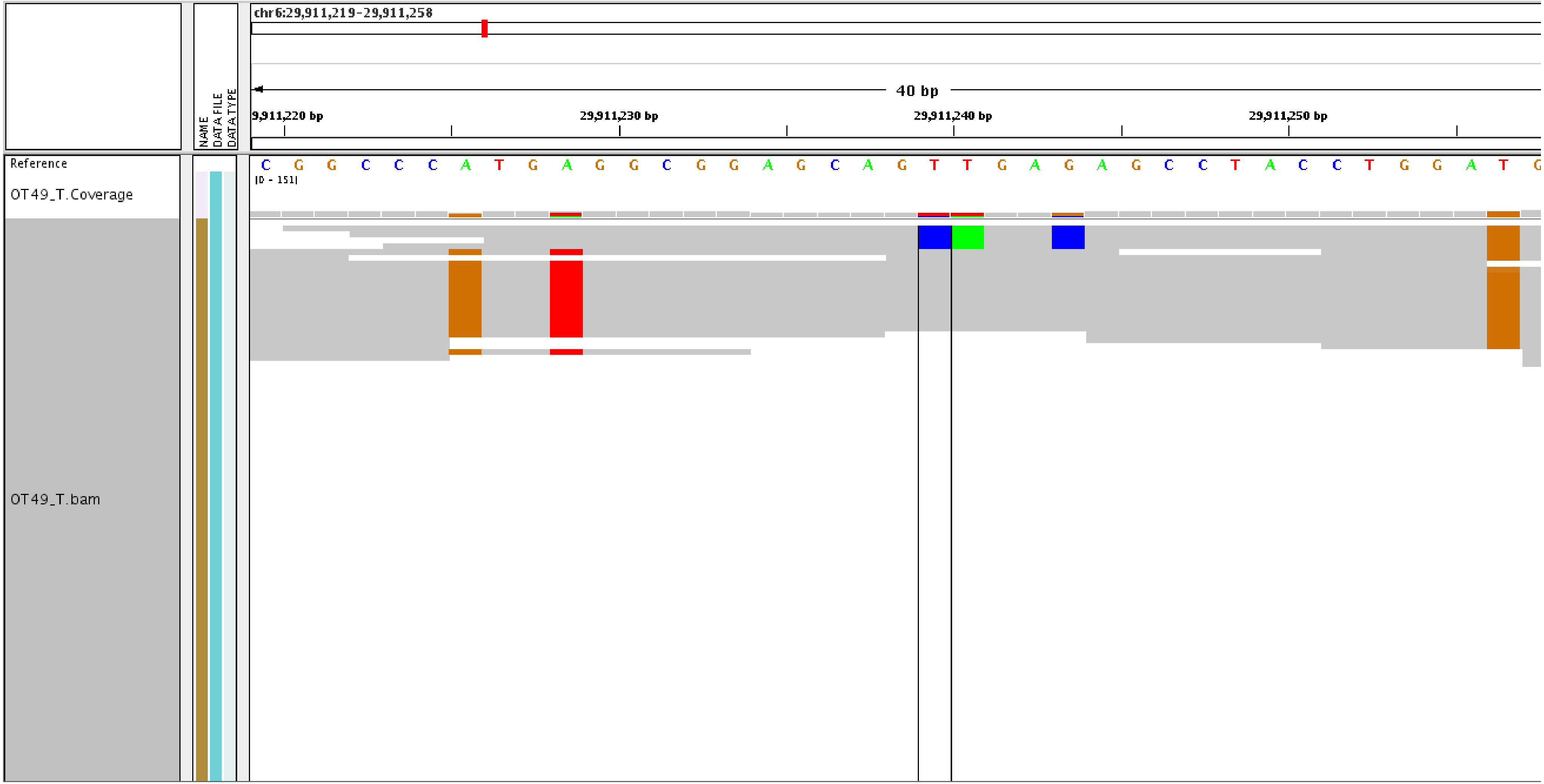

Event: chr6 29911240

Sample: OT49

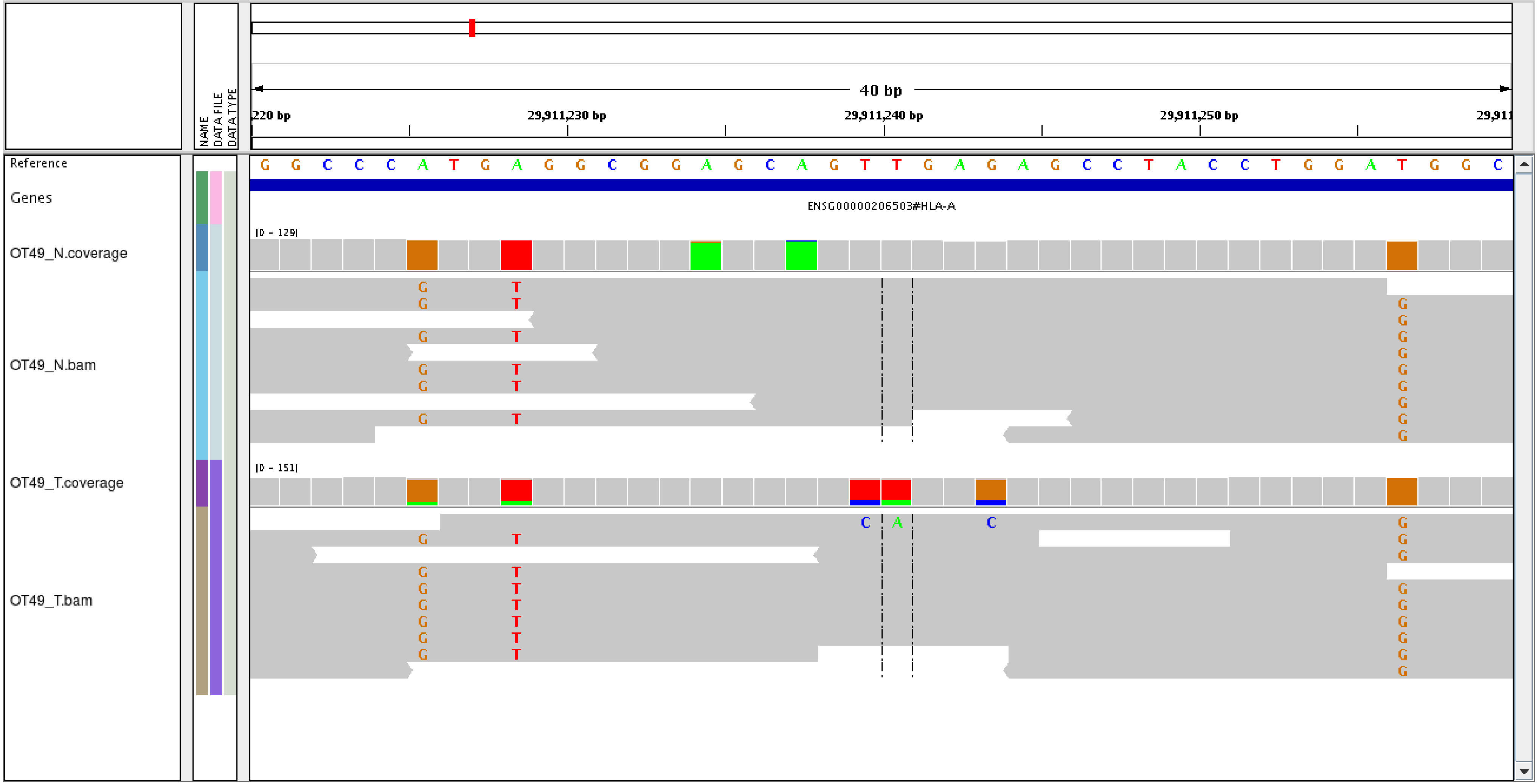

Event: chr6 29911243

Sample: OT49

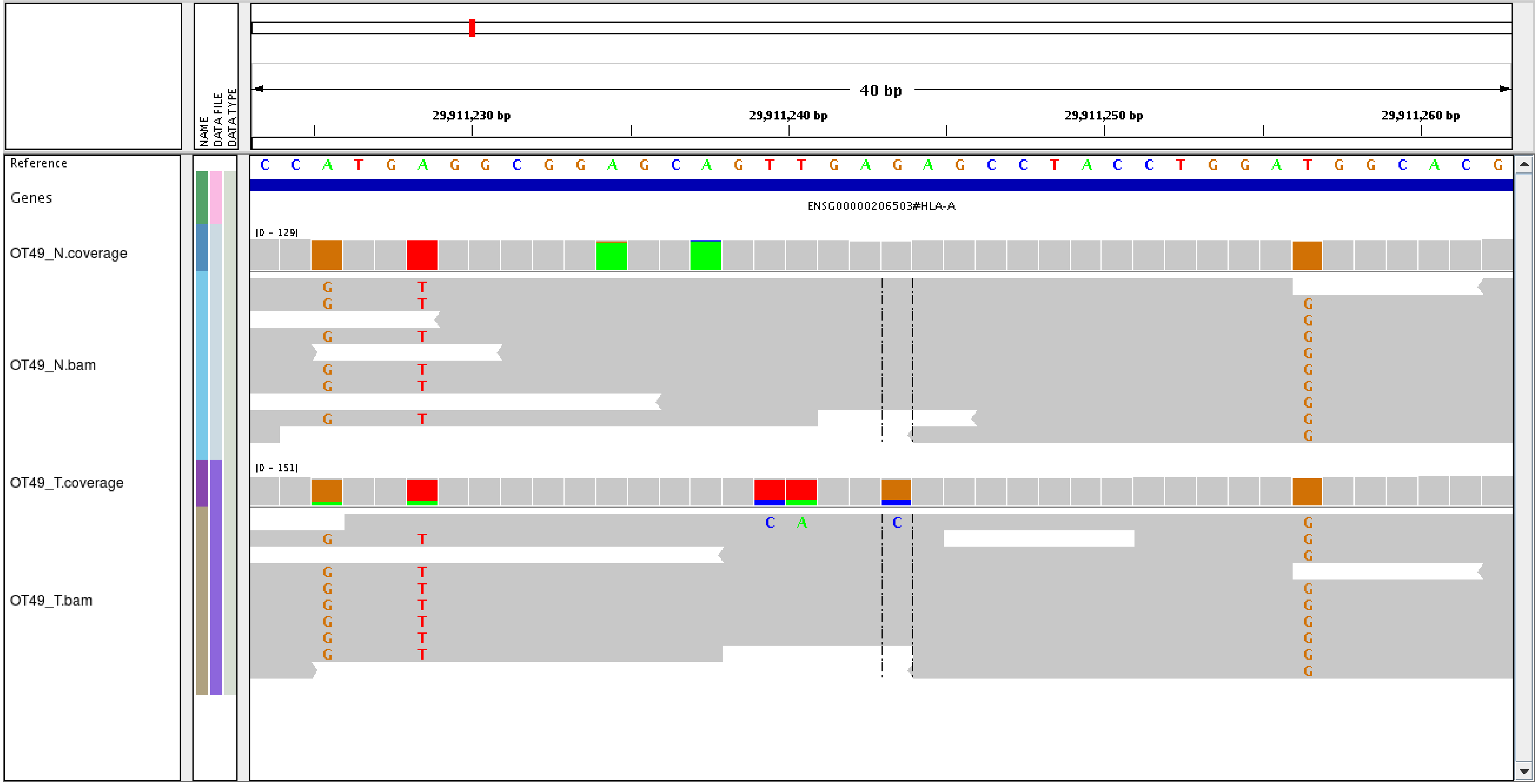

Event: chr1 120539907

Sample: OT50_N

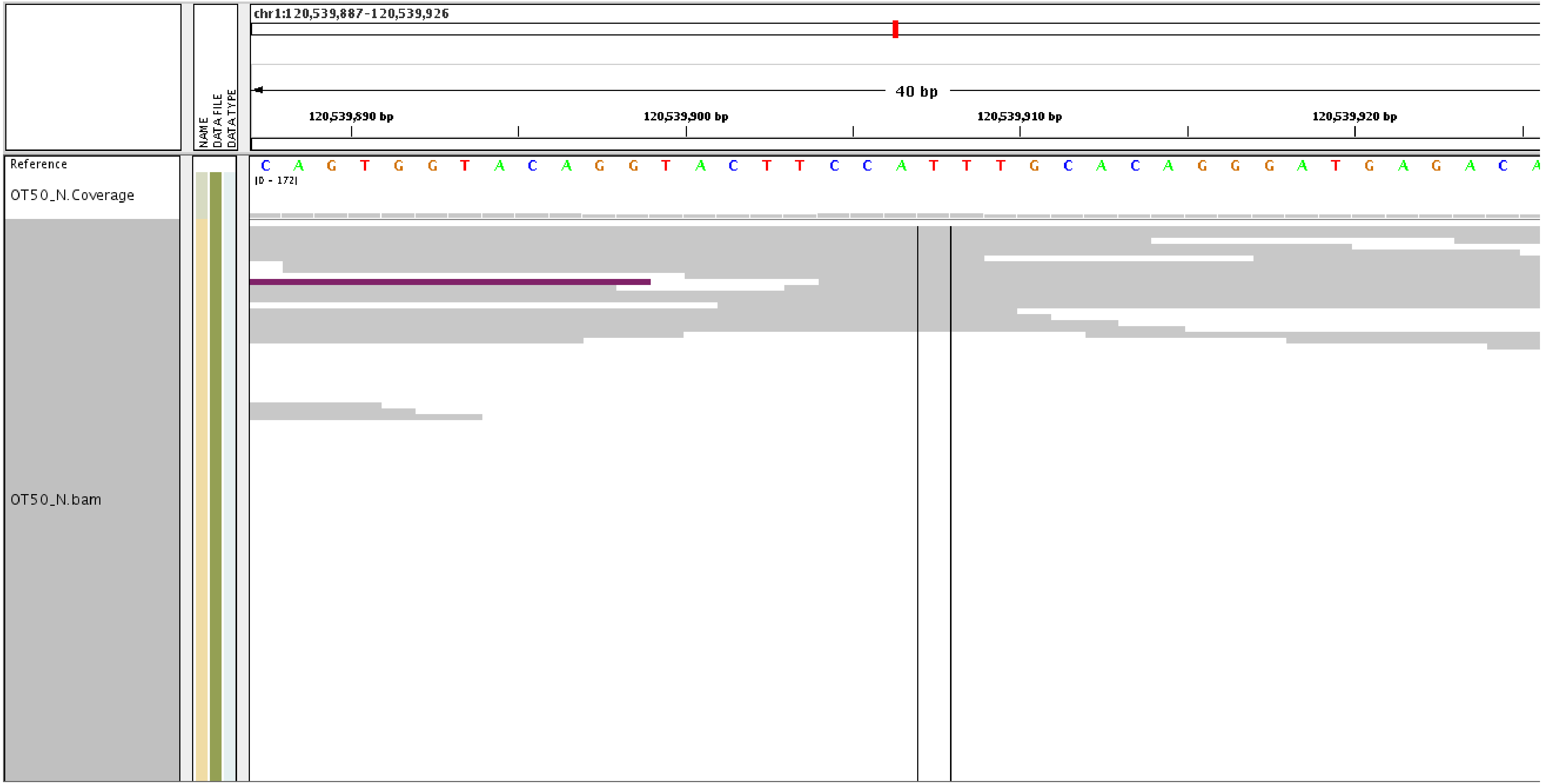

Event: chr1 120539907

Sample: OT50_T

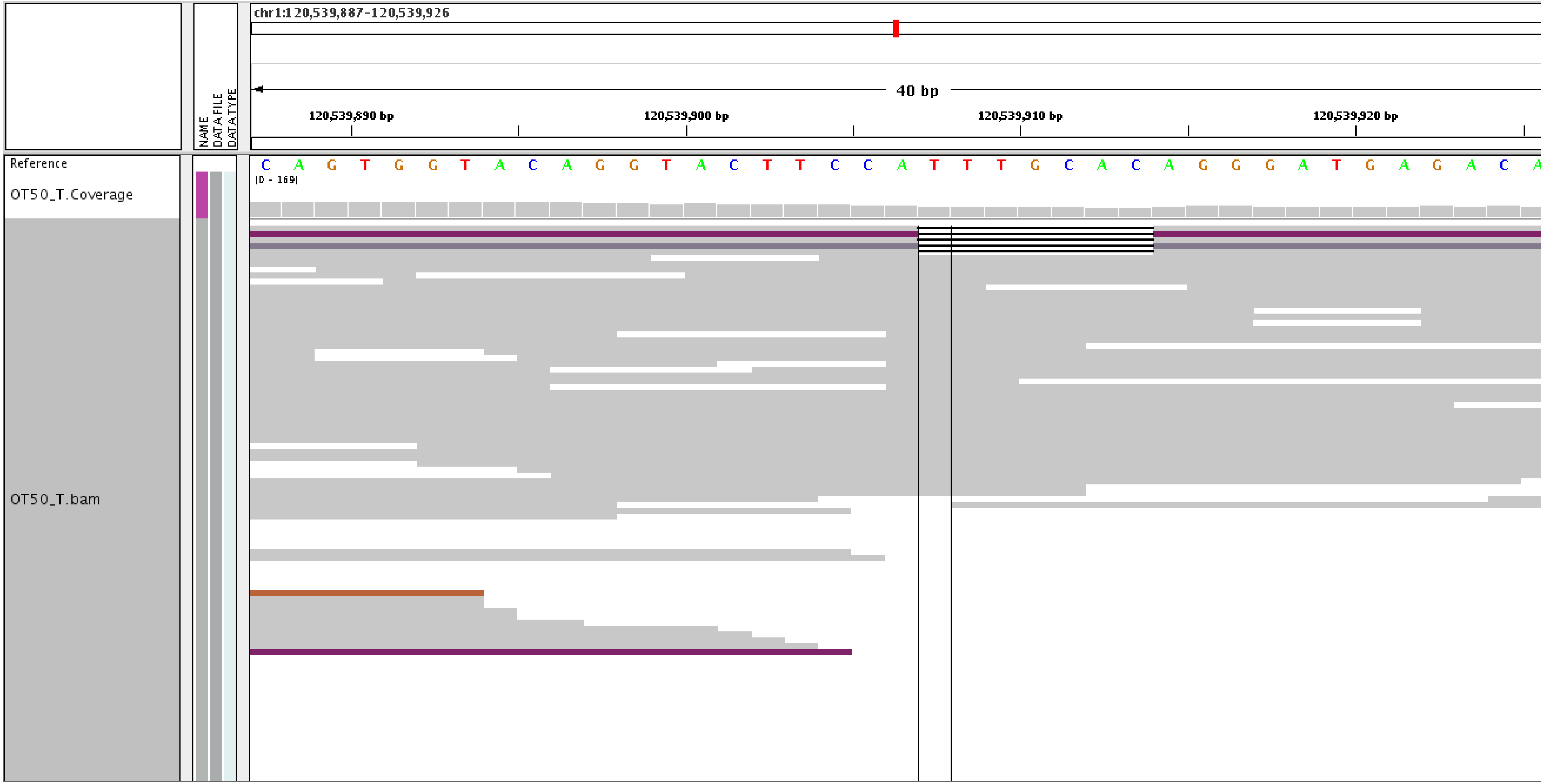

Event: chr17 7577579

Sample: OT50

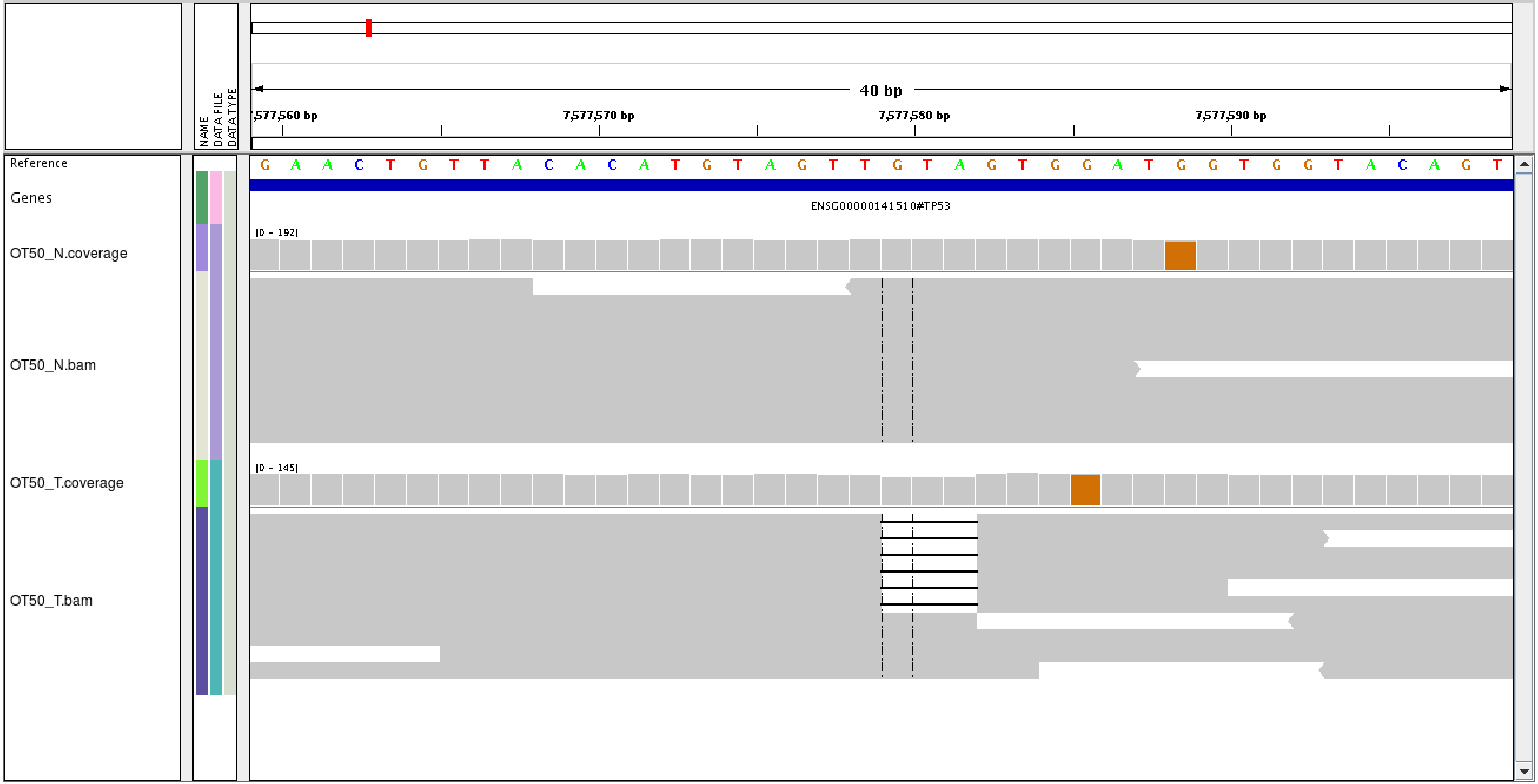

Event: chr12 49448728

Sample: OT51

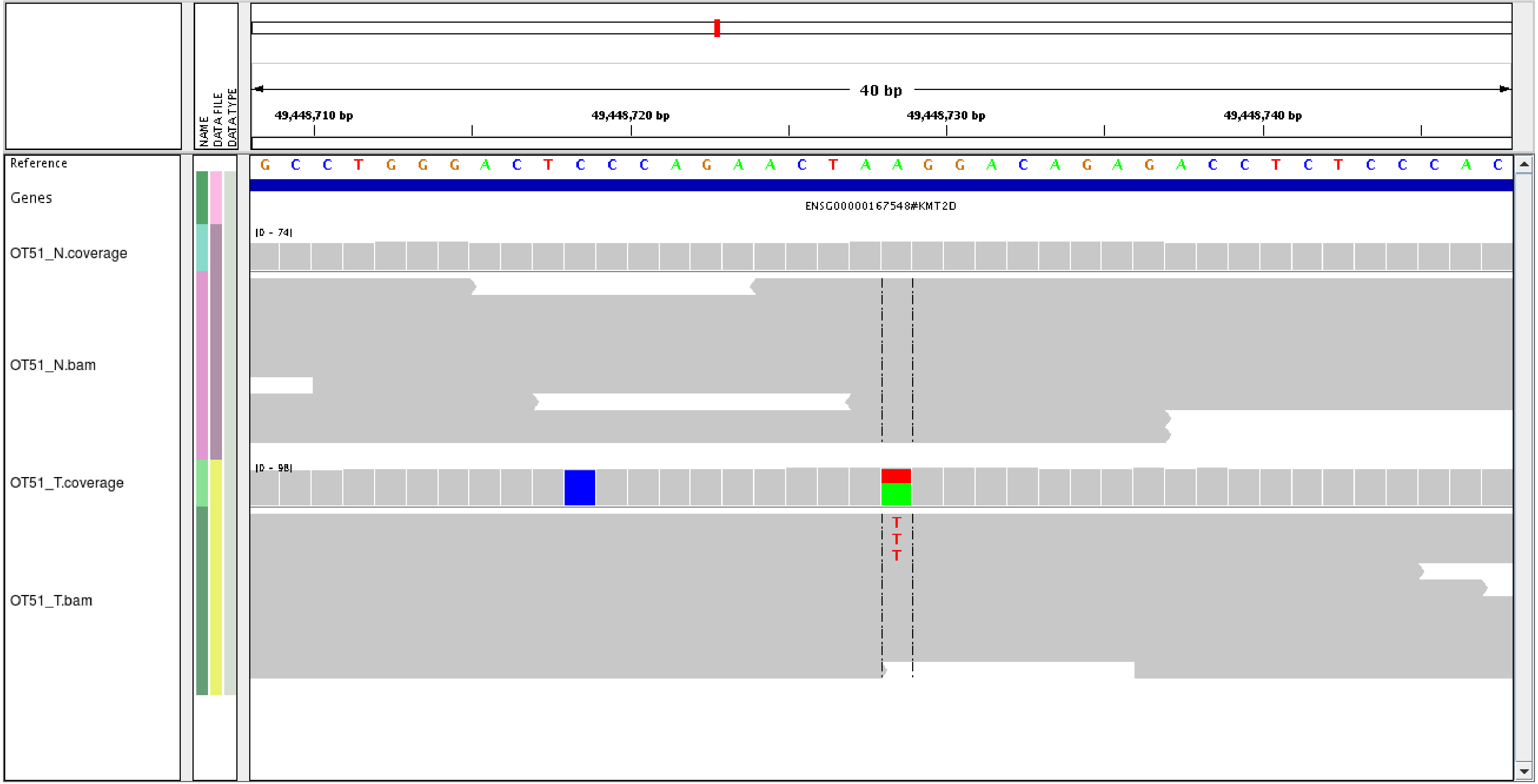

Event: chr17 7577498

Sample: OT51

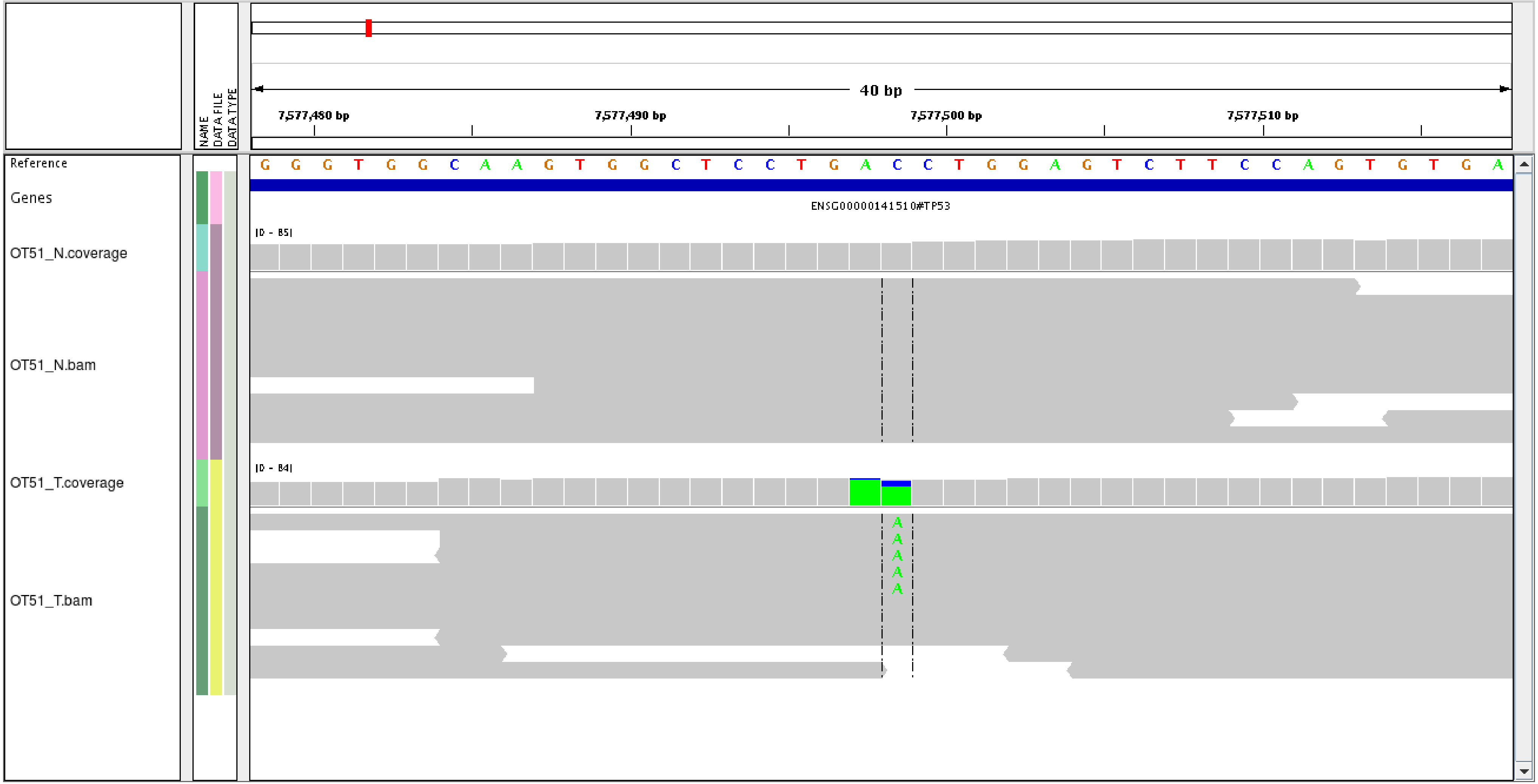

Event: chr6 80745035

Sample: OT53

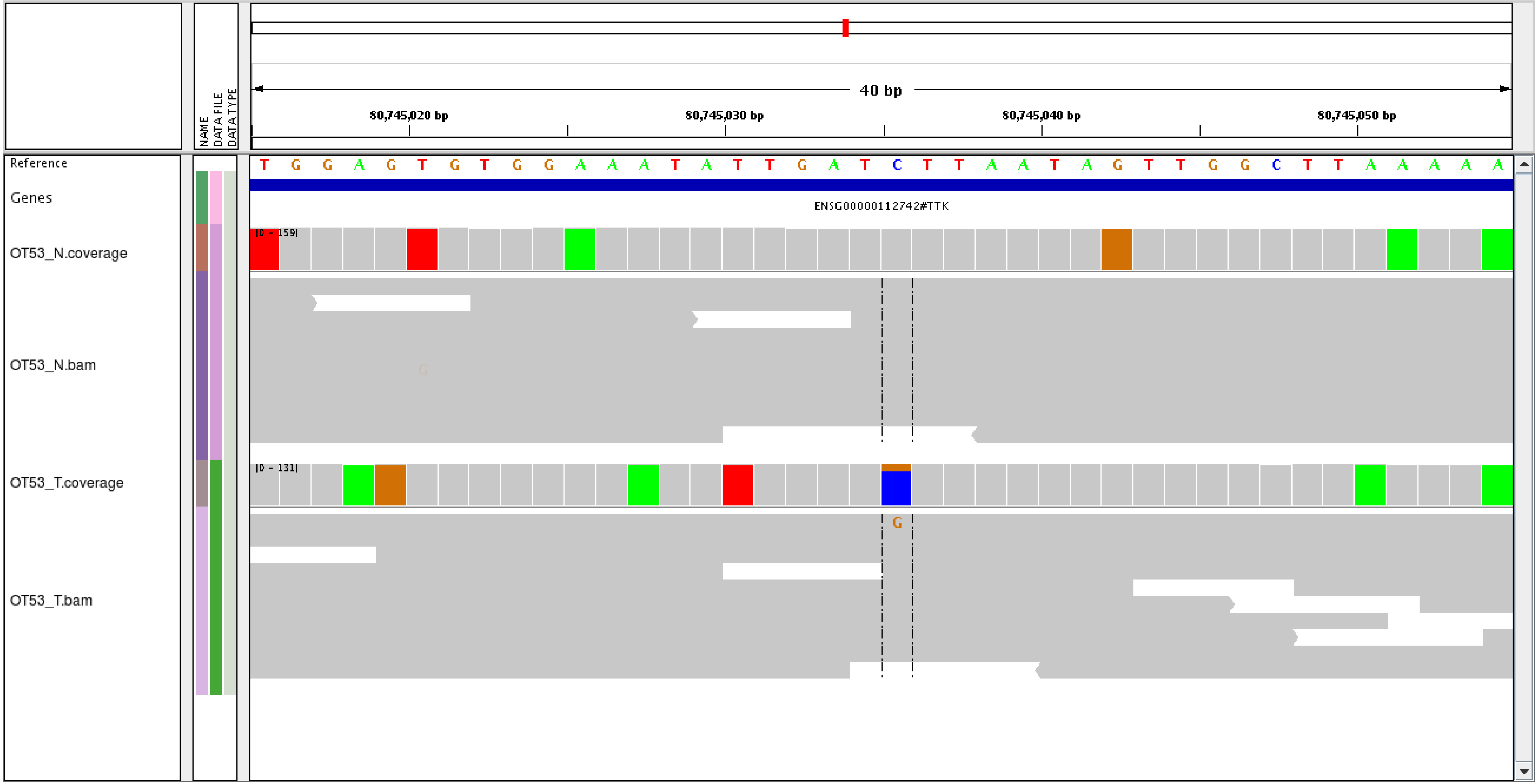

Event: chr8 95531469

Sample: OT53

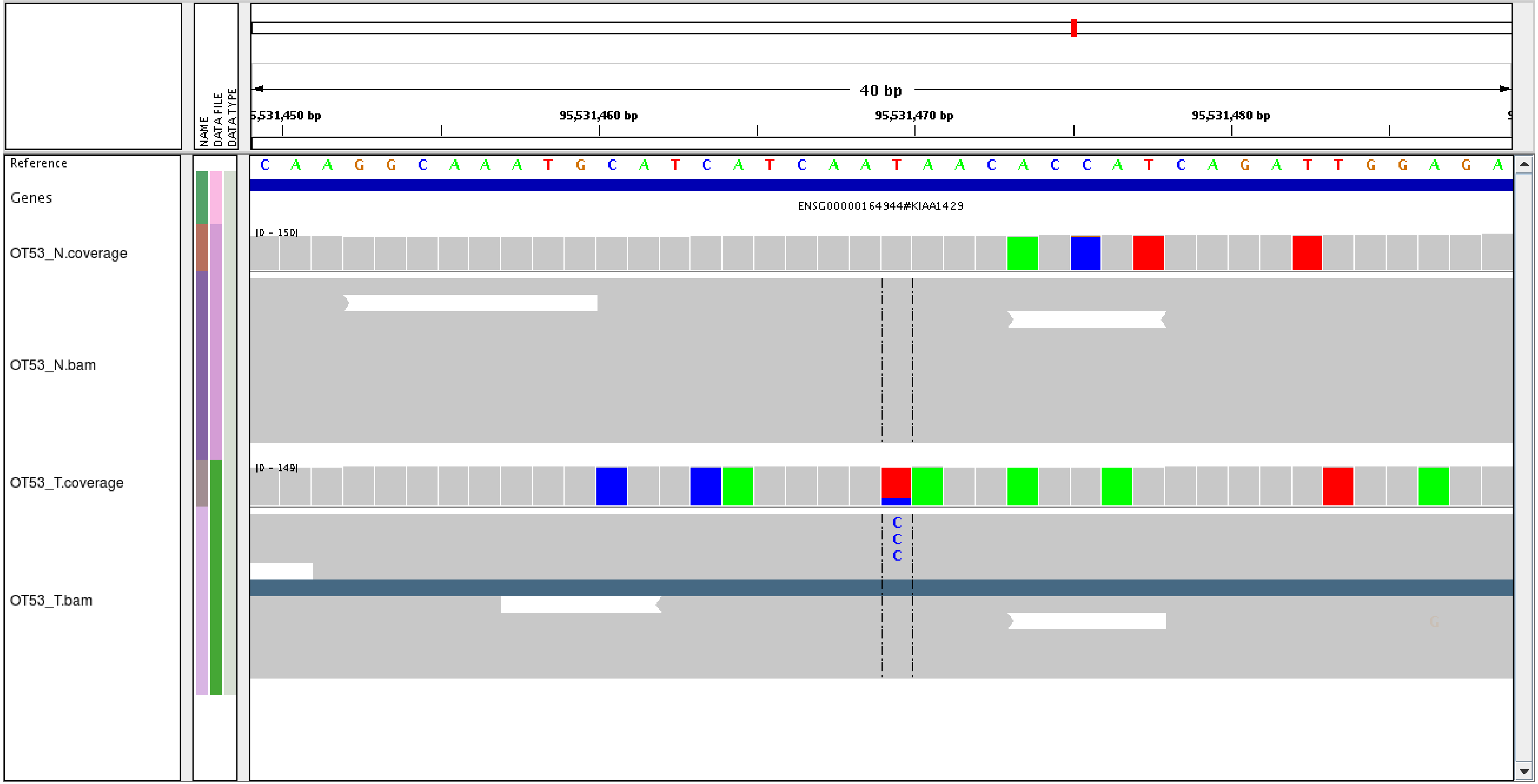

Event: chr17 7579391

Sample: OT54

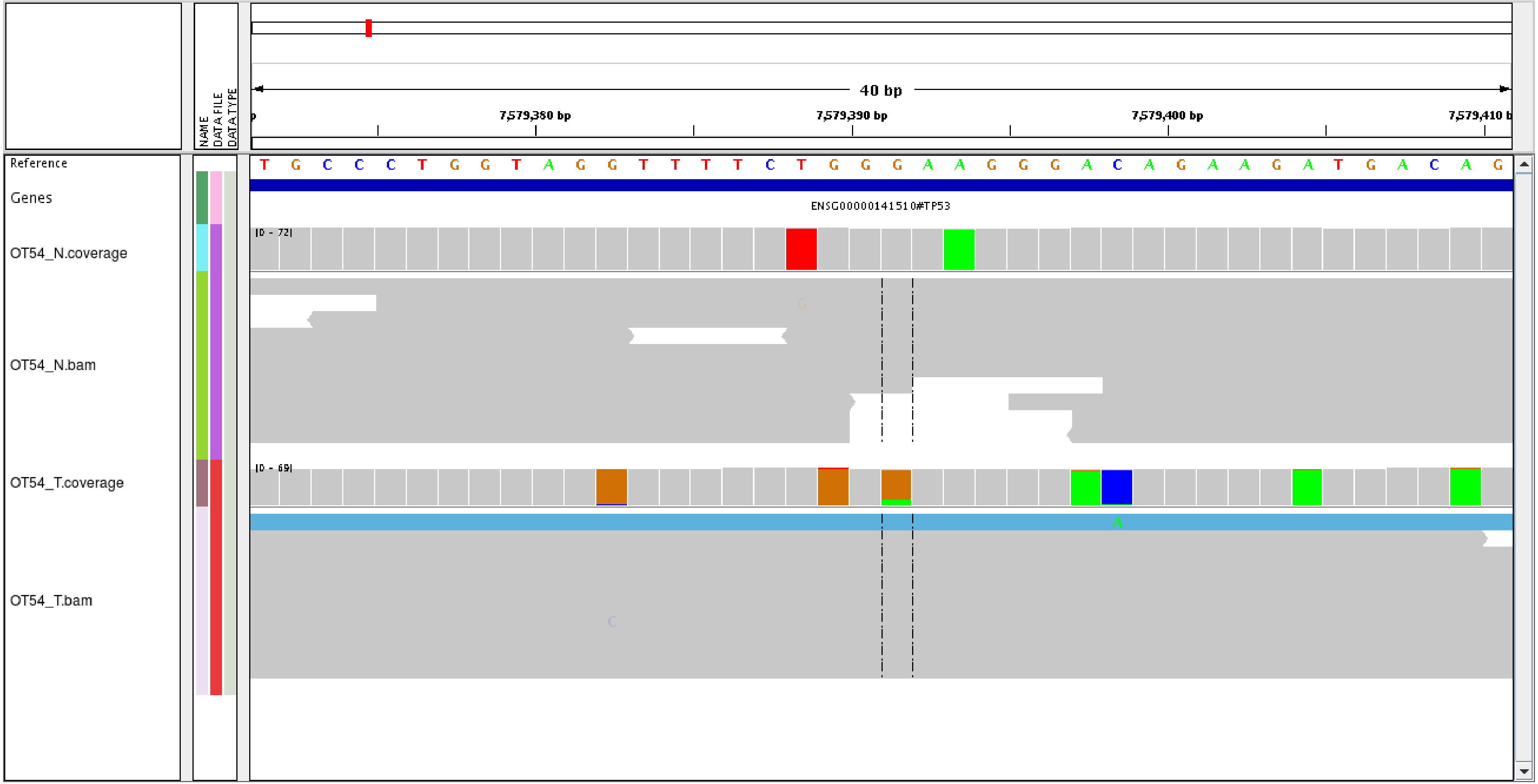

Event: chr2 178096452

Sample: OT55_B

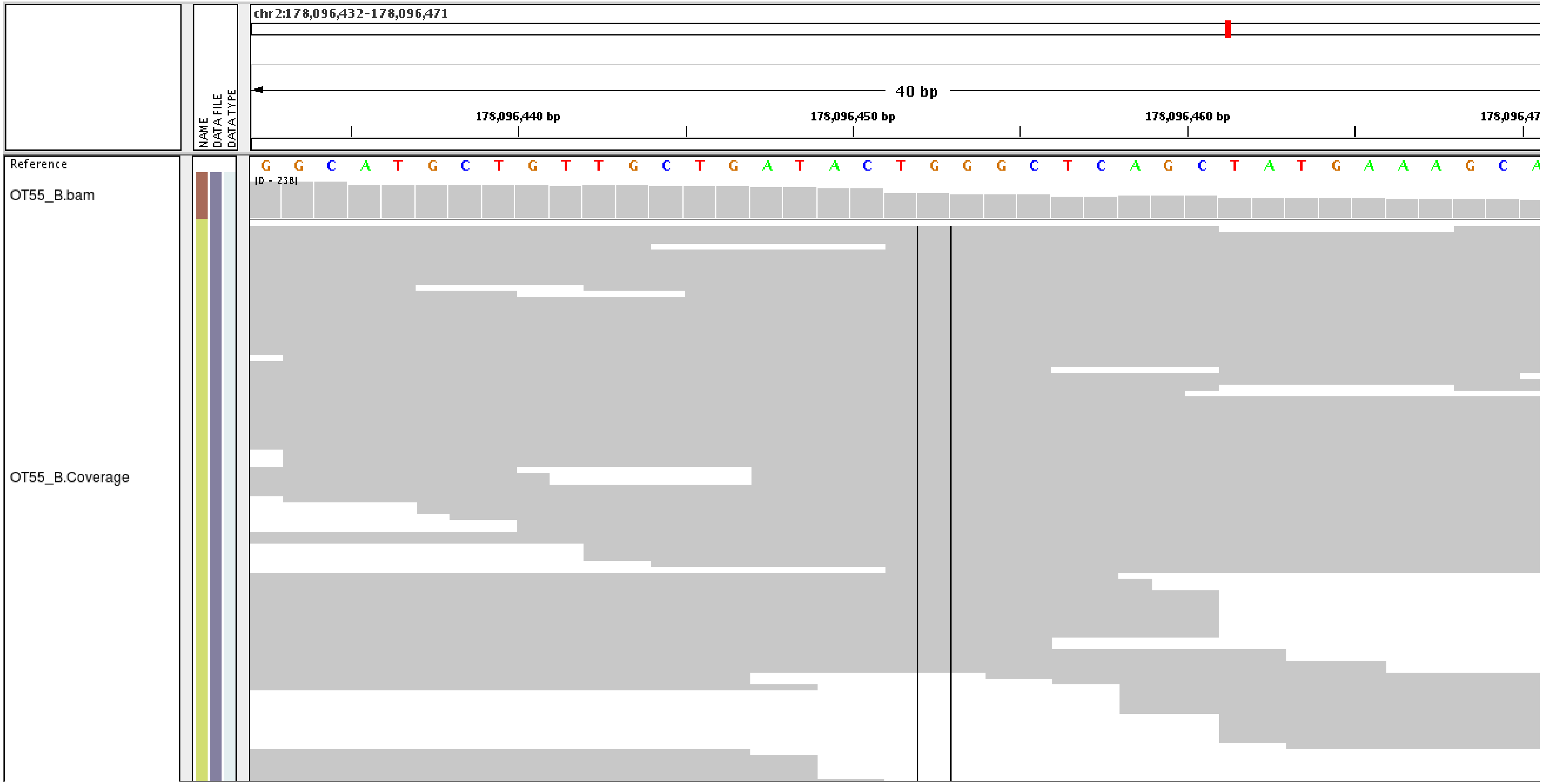

Event: chr2 178096452

Sample: OT55_T

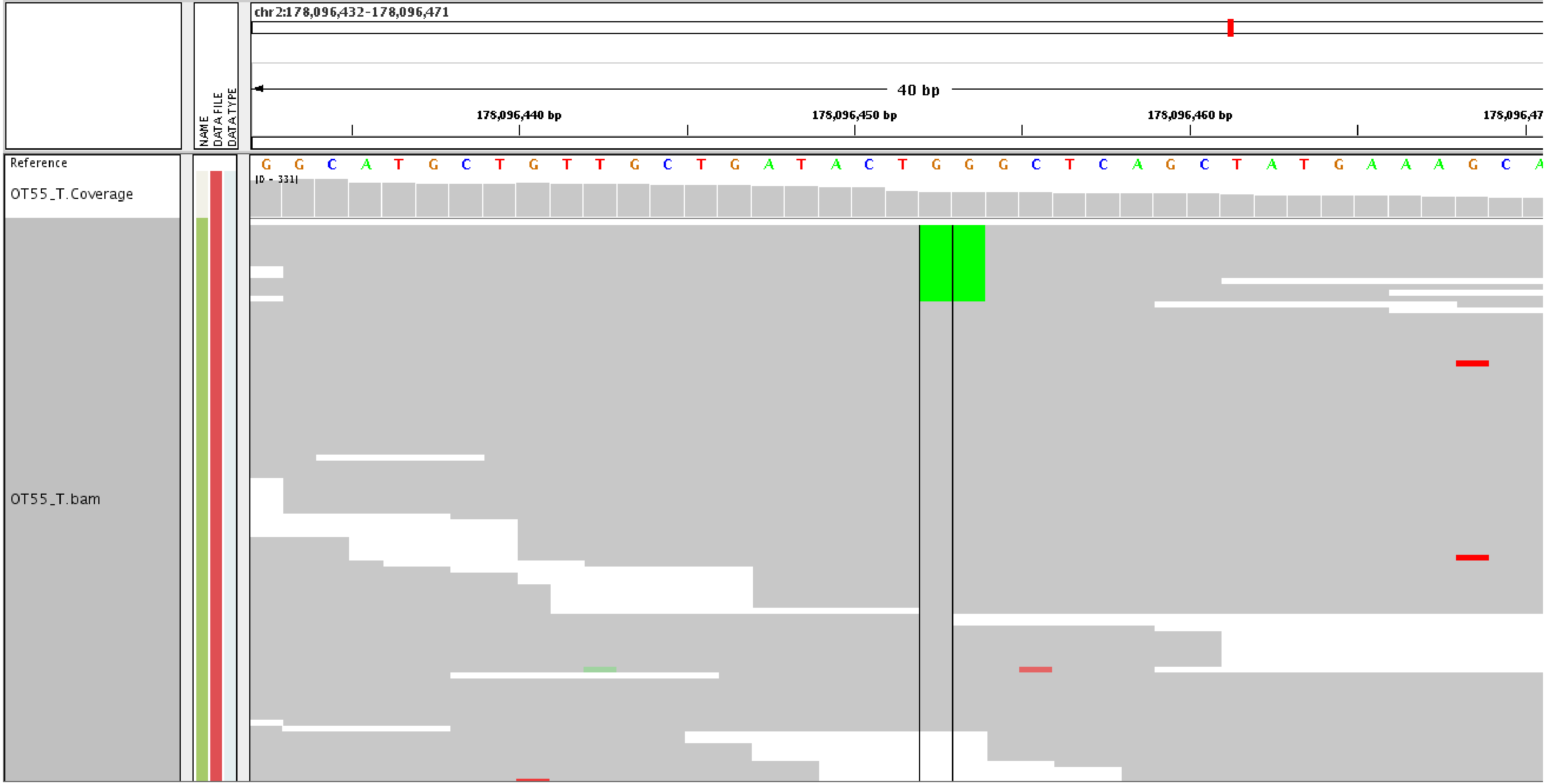

Event: chr2 178096453

Sample: OT55

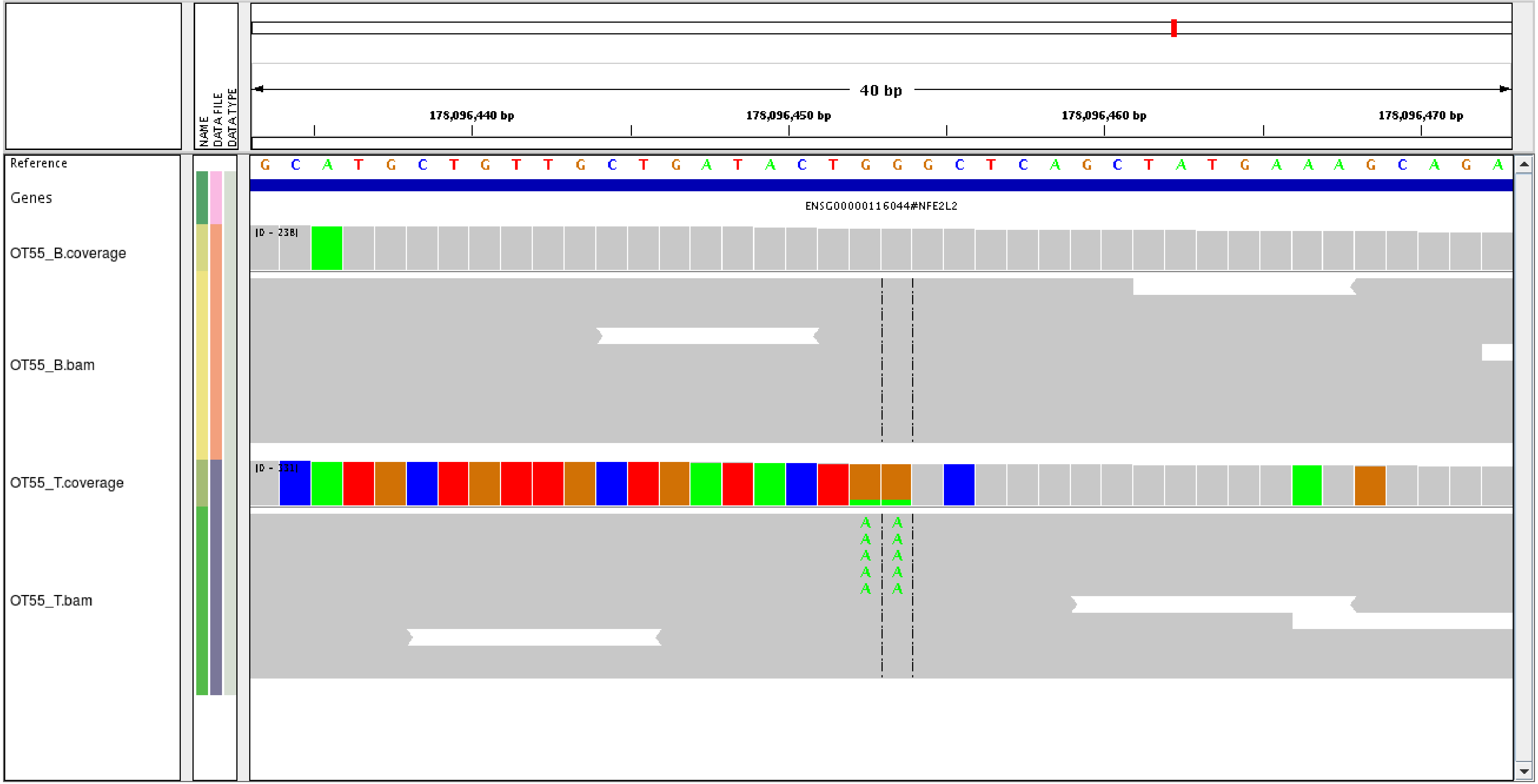

Event: chr2 178098953

Sample: OT55

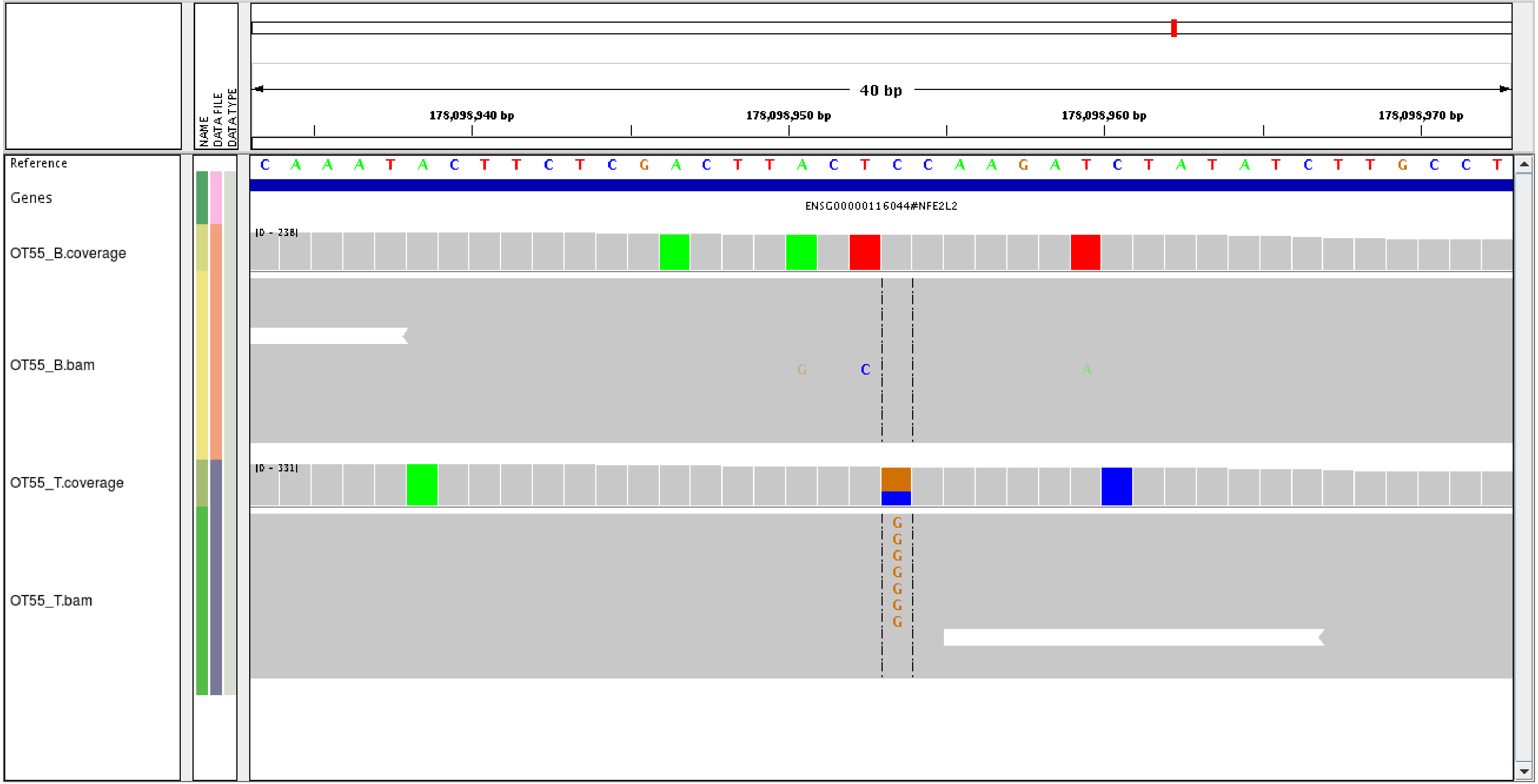

Event: chr5 86649055

Sample: OT55_B

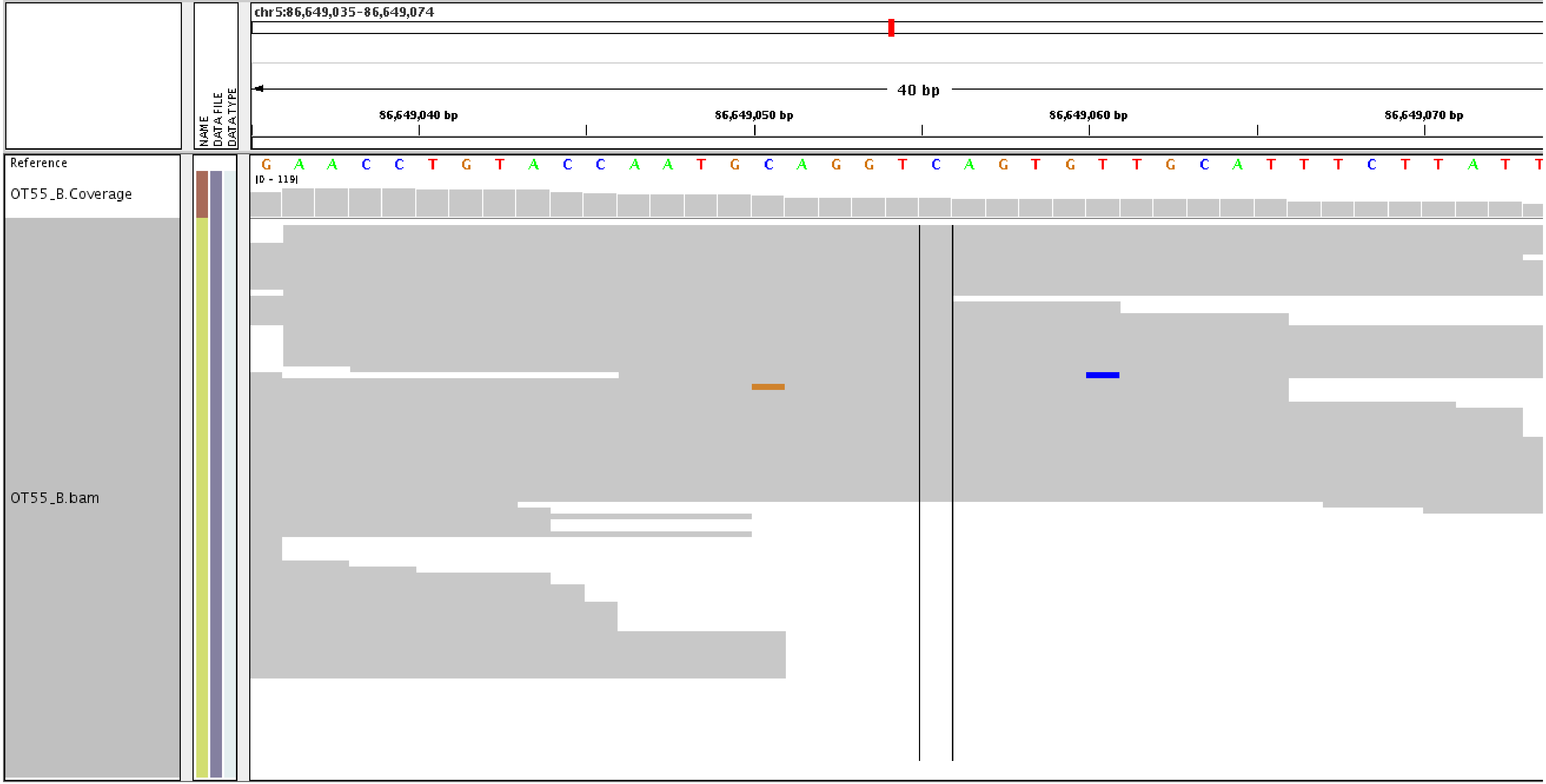

Event: chr5 86649055

Sample: OT55_T

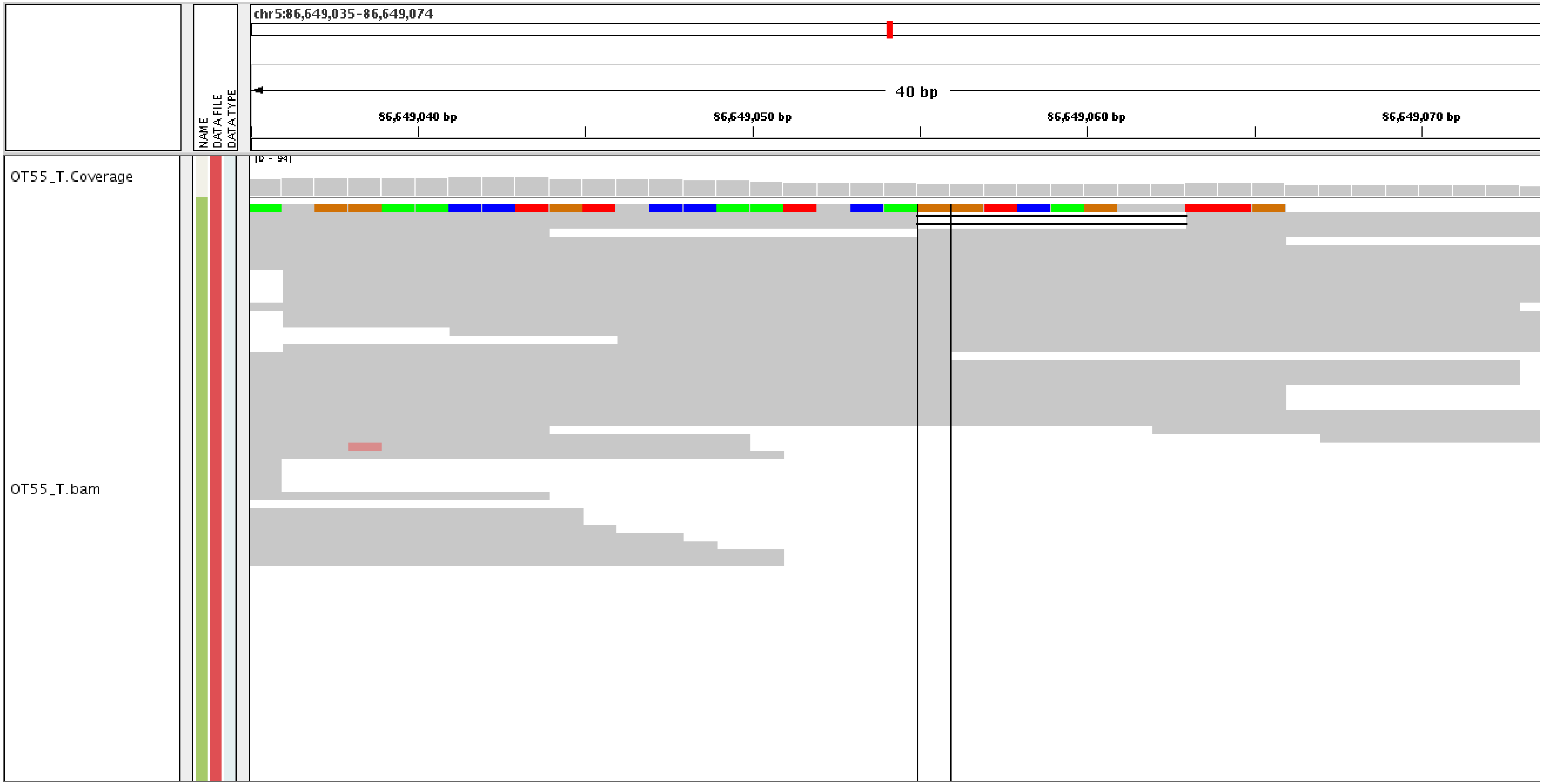

Event: chr17 7578475

Sample: OT55

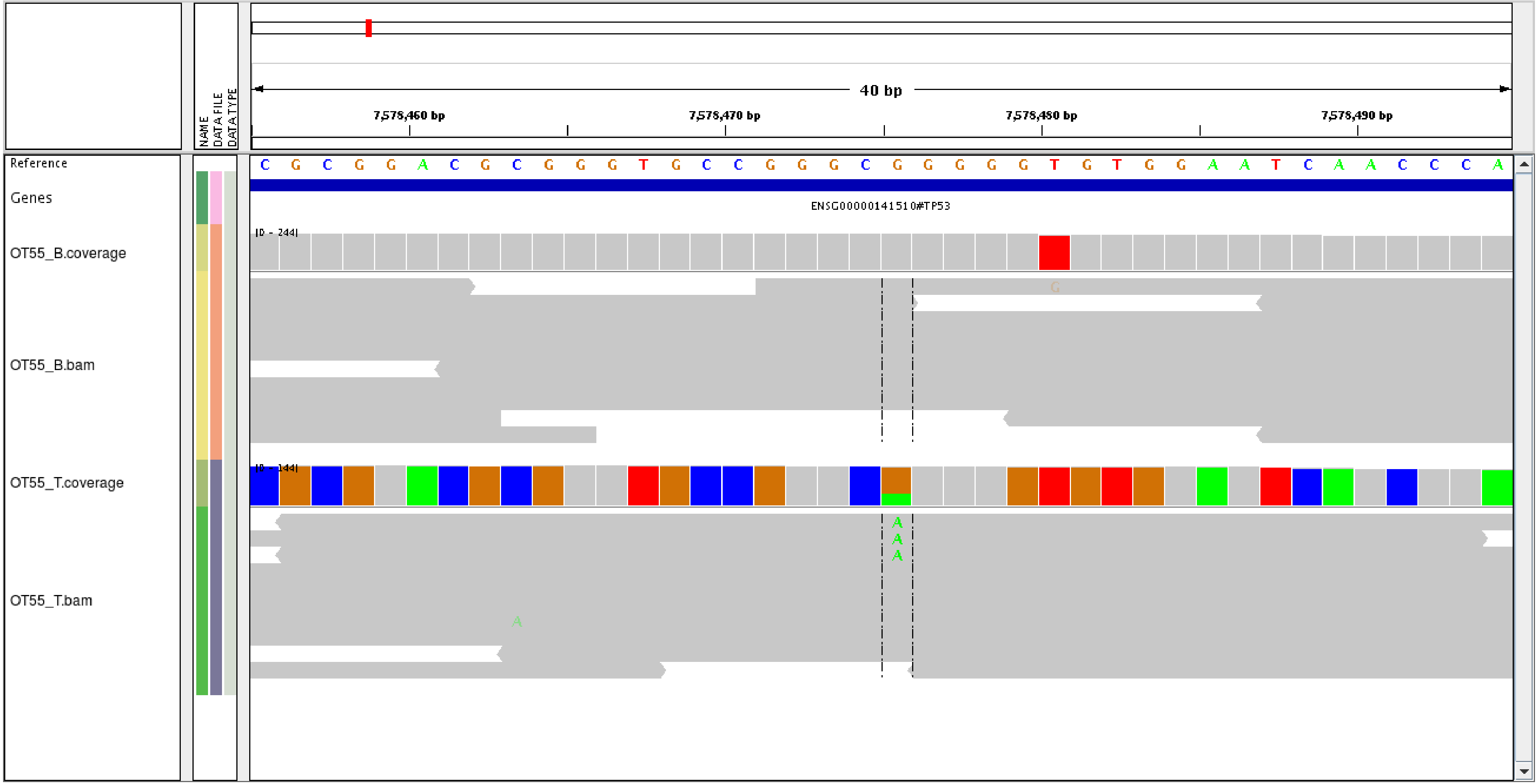

Supplement: Supplementary file 1 [file f1000research-4-7870-s0000.tgz › 4007fd05-e466-4e59-a4ef-512f9d41f650.zip!/Additional files/Additional file 20.pdf]
