# Supplementary material for: Integrated analysis of oral tongue squamous cell carcinoma identifies key variants and pathways linked to risk habits, HPV, clinical parameters and tumor recurrence
Source: F1000Res. 2015 Nov 5;4:1215. [Version 1] doi: 10.12688/f1000research.7302.1 (PMC4706066; doi:10.12688/f1000research.7302.1)
Supplement: Supplementary file 1 [file f1000research-4-7870-s0000.tgz › 4007fd05-e466-4e59-a4ef-512f9d41f650.zip!/Additional files/Additional file 3.pdf]

A. Discovery

Chr	Strand	Position	Allele	Symbol	CE validation tumor samples			CE validation normal samples		
					Sample	Change	Picture	Sample	Change	Picture
14	+	23450620	G/A	AJUBA	OT46_T	G/R(G or A)	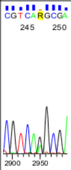	OT46_B	G/G	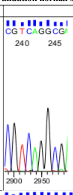
14	+	23450624	AAGC/-	AJUBA	OT46_T	AAGC/-	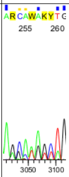	OT46_B	AAGC/AAGC	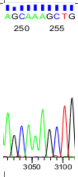
10	+	61956370	G/C	ANK3	OT18_T	G/C	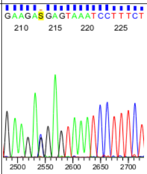	OT18_N	G/G	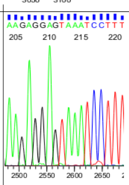
9	+	21970971	G/C	CDKN2A	OT32_T	G/C	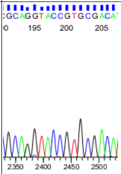	OT32_B	G/G	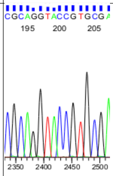
9	+	21971110	T/C	CDKN2A	OT21_T	T/C	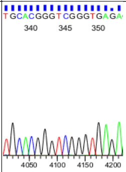	OT21_B	T/T	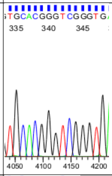
9	+	21971186	G/A	CDKN2A	OT38_T	G/A	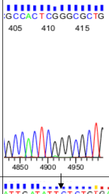	OT38_N	G/G	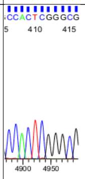
X	+	31676140	-/TT	DMD	OT43_T	-/TT	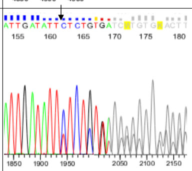	OT43_B	-/-	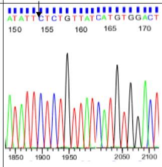
X	+	32519887	CC/-	DMD	OT27_T	CC/CC	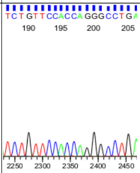	OT27_B	CC/CC	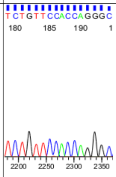
4	+	187532672	C/T	FAT1	OT44_T	C/T	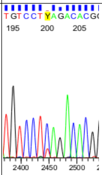	OT44_B	C/C	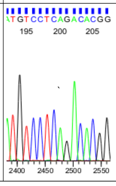

4	+	187539951	G/A	FAT1	OT44_T	G/A	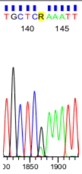	OT44_B	G/G	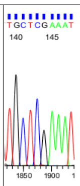
4	+	187549359	G/C	FAT1	OT24_T	Noisy data	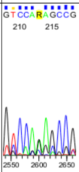	OT24_B	Noisy data	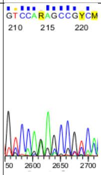
6	+	29910716	C/G	HLA-A	OT8_T	C/T	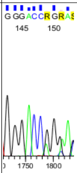	OT8_B	C/T	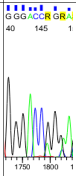
6	+	29910730	T/C	HLA-A	OT8_T	T/C	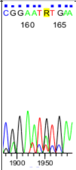	OT8_B	T/C	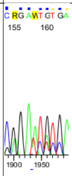
6	+	29911239	T/C	HLA-A	OT49_T	T/T	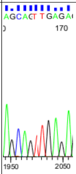	OT49_N	T/T	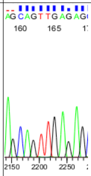
6	+	29911240	T/A	HLA-A	OT49_T	T/T	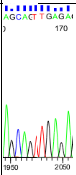	OT49_N	T/T	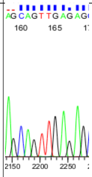
6	+	29911243	G/C	HLA-A	OT49_T	G/G	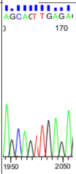	OT49_N	G/G	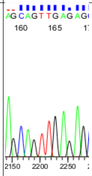
13	+	111366561	CCATCGAGTCC/-	ING1	OT42_T	CCATCGAGTCC/-	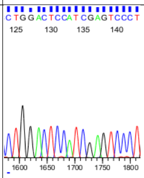	OT42_B	CCATCGAGTCC/-	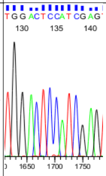

19	+	10602439	C/T	KEAP1	OT42_T	C/T	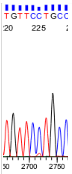	OT42_B	C/C	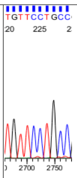
12	+	49448728	A/T	MLL2/KMT2D	OT51_T	A/T	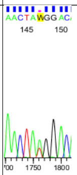	OT51_N	A/A	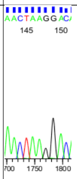
2	+	178096452	G/A	NFE2L2	OT55_T	G/A	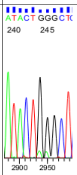	OT55_B	G/G	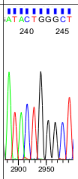
2	+	178096453	G/A	NFE2L2	OT55_T	G/A	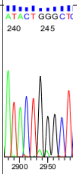	OT55_B	G/G	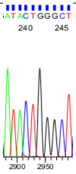
2	+	178098953	C/G	NFE2L2	OT55_T	C/G	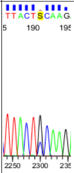	OT55_B	C/C	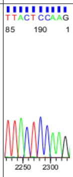
9	+	139405261	C/T	NOTCH1	OT6_T	C/T	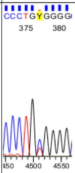	OT6_N	C/T	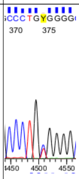
9	+	139409752	CGGCTCACAG/-	NOTCH1	OT14_T	CGGCTCACAG /-	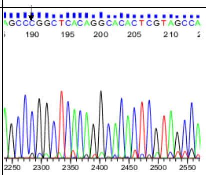	OT14_B	CGGCTCACAG/C GGCTCACAG	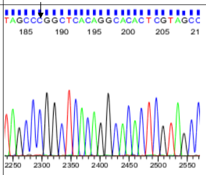
9	+	139413179	-/GCAGTT	NOTCH1	OT32_T	-/GCAGTT	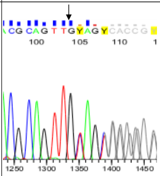	OT32_B	-/-	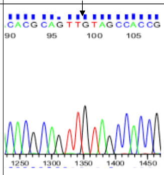

1	+	120464917	G/A	NOTCH2	OT10_T	G/A	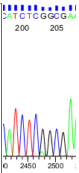	OT10_B	G/G	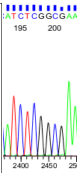
1	+	120539907	TTTGCAC/-	NOTCH2	OT50_T	TTTGCAC/-	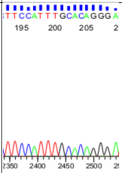	OT50_N	TTTGCAC/-	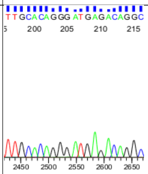
1	+	120572547	T/C	NOTCH2	OT21_T	T/C	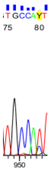	OT21_B	T/C	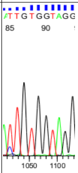
1	+	120611964	G/C	NOTCH2	OT33_T	G/G	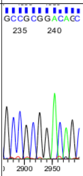	OT33_B	Noisy data	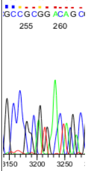
1	+	228464276	T/C	OBSCN	OT27_T	T/C	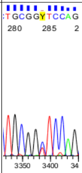	OT27_B	T/C	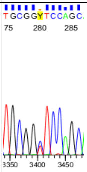
1	+	228506577	T/-	OBSCN	OT41_T	T/T	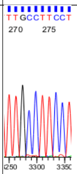	OT41_N	T/T	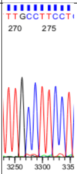
1	+	228564884	G/A	OBSCN	OT2_T	G/A	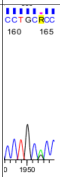	OT2_N	G/A	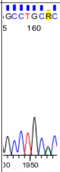
3	+	178942592	T/A	PIK3CA	OT11_T	T/A	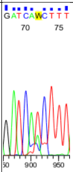	OT11_B	T/T	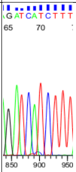

3	+	178952085	A/G	PIK3CA	OT27_T	A/G	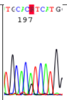	OT27_B	A/A	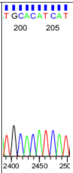
5	+	86564737	GACTCTCTGGATG/-	RASA1	OT32_T	Noisy data	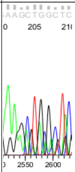	OT32_B	Noisy data	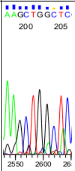
5	+	86648996	T/A	RASA1	OT42_T	T/A	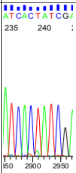	OT42_B	T/T	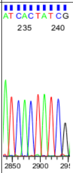
5	+	86649055	CAGTGTTG/-	RASA1	OT55_T	CAGTGTTG/CAGTGTT	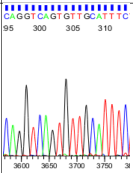	OT55_B	CAGTGTTG/CAGTGTT	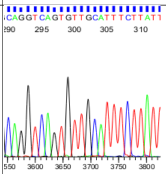
5	+	86672329	C/T	RASA1	OT11_T	C/T	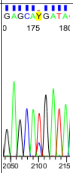	OT11_B	C/C	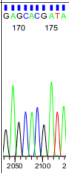
5	+	86681150	-/ATATT	RASA1	OT44_T	-/ATATT	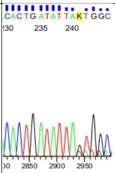	OT44_B	-/ATATT	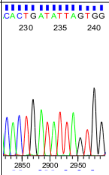
6	+	80745035	C/G	TTK	OT53_T	C/G	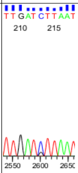	OT53_N	C/C	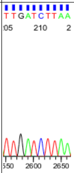
21	+	44513282	CCACCG/-	U2AF1	OT43_T	CCACCG/CCACC	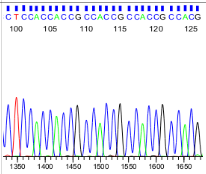	OT43_B	CCACCG/CCACC	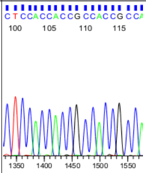

2	+	202136260	AGA/-	CASP8	OT25_T	AGA/-	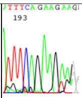	OT25_B	AGA/AGA	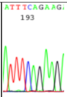
2	+	202141594	C/A	CASP8	OT7_T	C/C	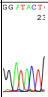	OT7_B	C/C	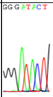
2	+	202149914	T/A	CASP8	OT27_T	T/A	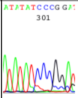	OT27_B	T/T	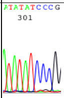
2	+	202150039	C/T	CASP8	OT44_T	C/T	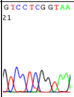	OT44_B	C/C	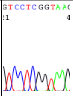
17	+	7574003	G/A	TP53	OT27_T	G/A	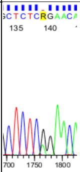	OT27_B	G/G	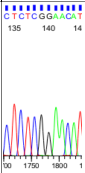
17	+	7574017	C/T	TP53	OT42_T	C/T	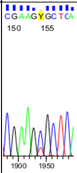	OT42_B	C/C	Noisy data
17	+	7577105	G/A	TP53	OT12_T	G/A	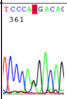	OT12_B	G/G	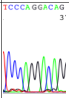
17	+	7577120	C/T	TP53	OT1_T	C/T	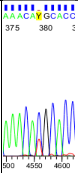	OT1_N	C/C	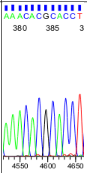

17	+	7577498	C/A	TP53	OT51_T	C/A	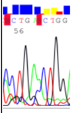	OT51_N	Noisy data	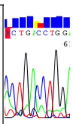
17	+	7577527	G/-	TP53	OT37_T	G/-	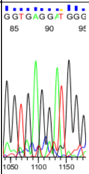	OT37_N	G/G	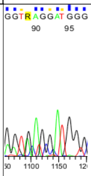
17	+	7577532	-/GGCCTCCGGTTCAT	TP53	OT46_T	Noisy data	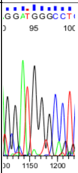	OT46_B	Noisy data	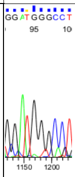
17	+	7577539	G/A	TP53	OT27_T	G/A	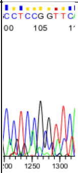	OT27_B	G/G	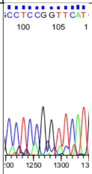
17	+	7577570	C/T	TP53	OT9_T	C/T	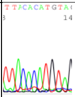	OT9_B	C/T	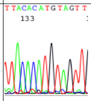
17	+	7577579	GTA/-	TP53	OT50_T	GTA/-	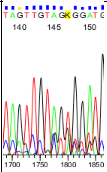	OT50_N	GTA/GTA	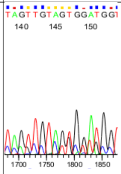
17	+	7578190	T/G	TP53	OT18_T	T/G	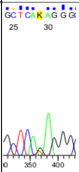	OT18_N	T/T	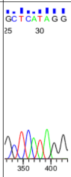
17	+	7578211	C/A	TP53	OT24_T	C/A	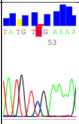	OT24_B	C/C	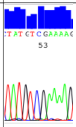

17	+	7578212	G/A	TP53	OT6_T	G/A	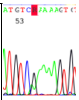	OT6_N	G/G	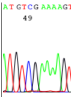
17	+	7578395	G/A	TP53	OT23_T	G/G	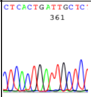	OT23_B	G/G	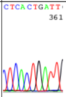
17	+	7578406	C/T	TP53	OT25_T	C/T	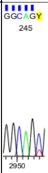	OT25_B	C/C	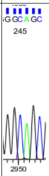
17	+	7578462	-/A	TP53	OT11_T	-/A	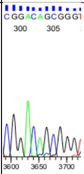	OT11_B	-/-	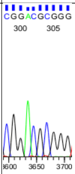
17	+	7578475	G/A	TP53	OT55_T	G/A	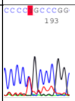	OT55_B	G/G	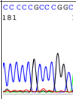
17	+	7578524	G/A	TP53	OT22_T	G/A	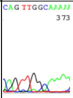	OT22_N	G/G	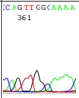
17	+	7578532	A/G	TP53	OT40_T	A/G	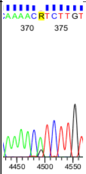	OT44_B	A/A	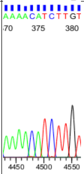
17	+	7579391	G/A	TP53	OT54_T	G/A	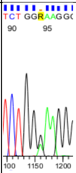	OT54_N	G/G	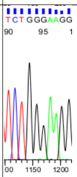

B. Validation

Chr	Strand	Position	Allele	Symbol	Sample	Change	Picture
17	+	7579406	G/T	TP53	OT98	G/T	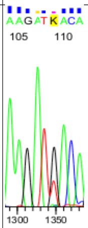
17	+	7579476	G/C	TP53	OT98	G/C	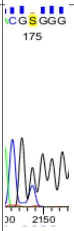
17	+	7579472	G/C	TP53	OT105	G/C	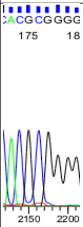
17	+	7579476	G/C	TP53	OT66	G/C	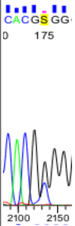
17	+	7577517	A/C	TP53	OT66	A/C	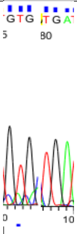
17	+	7579476	G/C	TP53	OT86	G/C	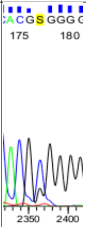

17	+	7578203	C/T	TP53	OT86	C/T	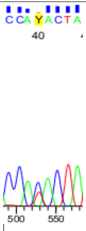
17	+	7579851	G/T	TP53	OT87	G/T	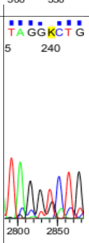
17	+	7579472	G/C	TP53	OT106	G/C	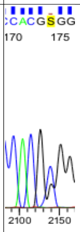
17	+	7577121	G/A	TP53	OT90	G/A	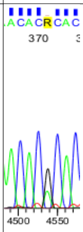
17	+	7579841	T/A	TP53	OT99	T/A	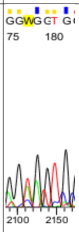
17	+	7579891	G/T	TP53	OT99	G/T	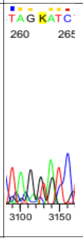

17	+	7577120	C/T	TP53	OT60	C/T	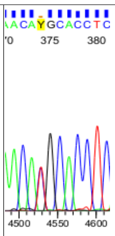
17	+	7579472	G/C	TP53	OT60	G/C	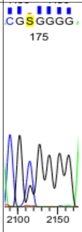
17	+	7577583	G/T	TP53	OT89	G/T	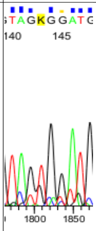
17	+	7579472	G/C	TP53	OT107	G/C	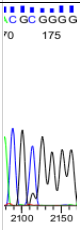
17	+	7579472	G/C	TP53	OT70	G/C	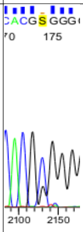
17	+	7579472	G/C	TP53	OT94	G/C	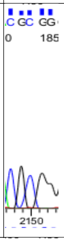

17	+	7579472	G/C	TP53	OT93	G/C	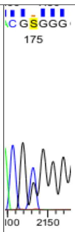
17	+	7579472	G/C	TP53	OT96	G/C	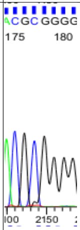
17	+	7579851	G/C	TP53	OT102	G/C	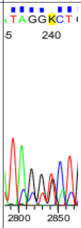
17	+	7579472	G/C	TP53	OT102	G/C	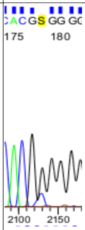
17	+	7577525	G/A	TP53	OT102	G/A	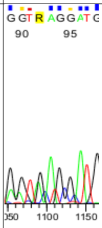
17	+	7579904	C/A	TP53	OT102	C/A	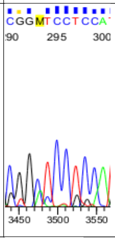

17	+	7577582	T/G	TP53	OT102	T/G	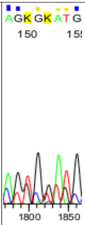
17	+	7577584	G/T	TP53	OT102	G/T	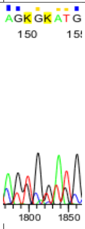
17	+	7579472	G/C	TP53	OT109	G/C	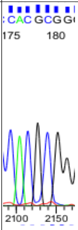
17	+	7579472	G/C	TP53	OT101	G/C	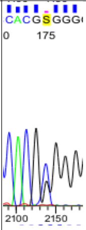
17	+	7579472	G/C	TP53	OT74	G/C	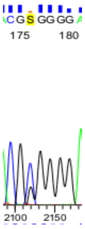
17	+	7577510	C/T	TP53	OT111	C/T	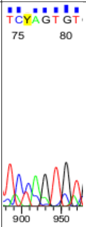

17	+	7577035	T/C	TP53	OT111	T/C	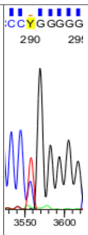
17	+	7577525	G/A	TP53	OT111	G/A	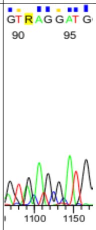
17	+	7577582	G/A	TP53	OT111	G/A	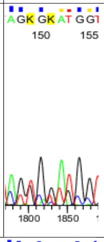
17	+	7577584	G/T	TP53	OT111	G/T	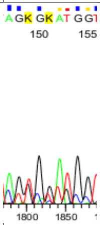
17	+	7579472	G/C	TP53	OT63	G/C	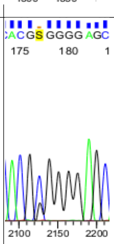
17	+	7579472	G/C	TP53	OT79	G/C	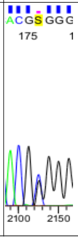

17	+	7579472	G/C	TP53	OT113	G/C	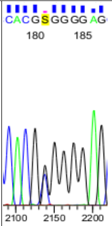
17	+	7579472	G/C	TP53	OT60	G/C	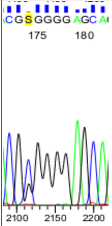
17	+	7577120	C/T	TP53	OT60	C/T	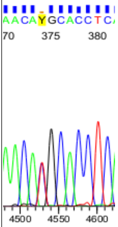
2	+	202149698	C/T	CASP8	OT66	C/T	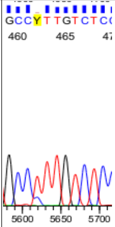
2	+	202149698	C/T	CASP8	OT66	C/T	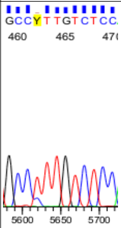
2	+	202136293	A/C	CASP8	OT86	A/C	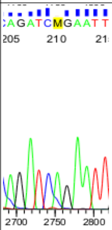

2	+	202149696	C/T	CASP8	OT87	C/T	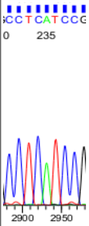
2	+	202149696	C/T	CASP8	OT106	C/T	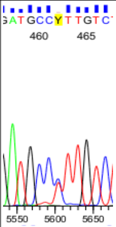
2	+	202149583	-/G	CASP8	OT60	-/G	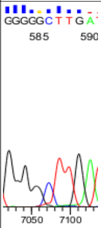
5	+	86672225	A/C	RASA	OT64	A/C	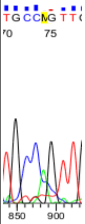
5	+	86672228	T/C	RASA	OT90	T/C	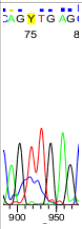
5	+	86648981	G/A	RASA	OT76	G/A	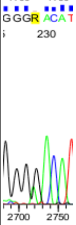

5	+	86648975	A/C	RASA	OT102	A/C	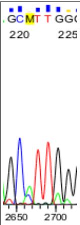
5	+	86648981	G/A	RASA	OT110	G/A	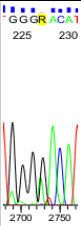
5	+	86681147	A/C	RASA	OT57	A/C	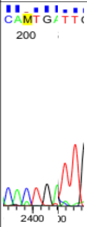
5	+	8668141	A/C	RASA	OT76	A/C	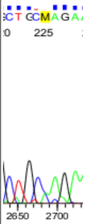
5	+	8668151	G/T	RASA	OT76	G/T	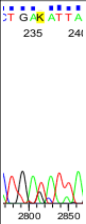
5	+	8668172	G/A	RASA	OT76	G/A	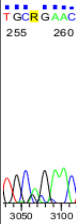

9	+	21970915	G/C	CDKN2A	OT62	G/C	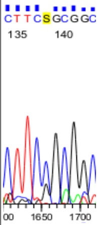
9	+	21970940	C/T	CDKN2A	OT62	C/T	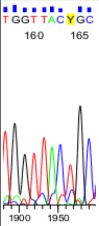
9	+	21971095	C/T	CDKN2A	OT62	C/T	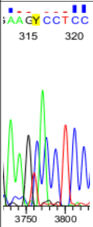
9	+	21970903	C/T	CDKN2A	OT65	C/T	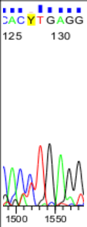
9	+	21970915	G/C	CDKN2A	OT65	G/C	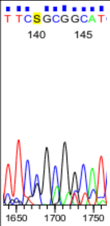
9	+	21970930	G/T	CDKN2A	OT65	G/T	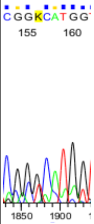

9	+	21970940	C/T	CDKN2A	OT65	C/T	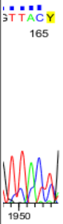
9	+	21970914	G/C	CDKN2A	OT67	G/C	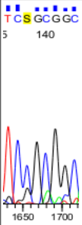
9	+	21970915	C/G	CDKN2A	OT75	C/G	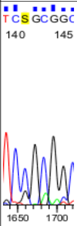
9	+	21970900	C/T	CDKN2A	OT77	C/T	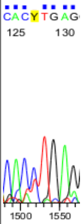
9	+	21970910	C/G	CDKN2A	OT77	C/G	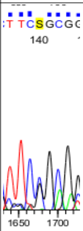
9	+	21970930	G/T	CDKN2A	OT77	G/T	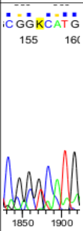

9	+	21970940	C/T	CDKN2A	OT77	C/T	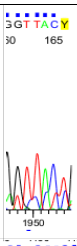
9	+	21970947	G/T	CDKN2A	OT77	G/T	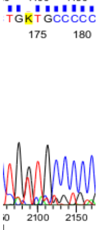
9	+	21970986	A/T	CDKN2A	OT77	A/T	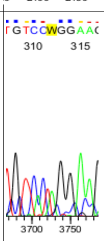
9	+	21970915	G/C	CDKN2A	OT78	G/C	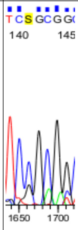
9	+	21970914	G/C	CDKN2A	OT82	G/C	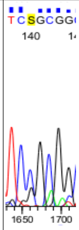
9	+	21970914	G/C	CDKN2A	OT91	G/C	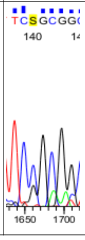

9	+	21970948	G/T	CDKN2A	OT91	G/T	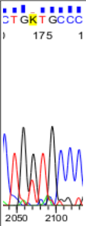
9	+	21970914	G/C	CDKN2A	OT103	G/C	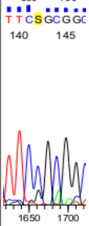
9	+	21970914	G/C	CDKN2A	OT102	G/C	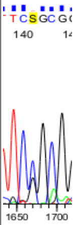
9	+	21971111	G/A	CDKN2A	OT102	G/A	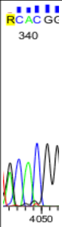
9	+	**21971085**	A/T	CDKN2A	OT112	A/T	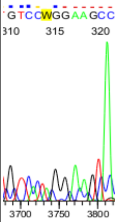

C. Frequency of changes

Gene	Frequency of changes	
	Discovery set	Discovery and Validation sets (for >=5 frequency from discovery set)
AJUBA	2	
ANK3	2	
CASP8	6	6.90
CDKN2A	6	16.47
DMD	2	
FAT1	2	
HLA-A	0	
ING1	0	
KEAP1	2	
MLL2/KMT2D	2	
NFE2L2	2	
NOTCH1	4	
NOTCH2	2	
OBSCN	0	
PIK3CA	4	
RASA1	6	10.71
TP53	36	34.78
TTK	0	
U2AF1	0	
