# Supplementary material for: Integrated analysis of oral tongue squamous cell carcinoma identifies key variants and pathways linked to risk habits, HPV, clinical parameters and tumor recurrence
Source: F1000Res. 2015 Nov 5;4:1215. [Version 1] doi: 10.12688/f1000research.7302.1 (PMC4706066; doi:10.12688/f1000research.7302.1)
Supplement: Supplementary file 1 [file f1000research-4-7870-s0000.tgz › 4007fd05-e466-4e59-a4ef-512f9d41f650.zip!/Additional files/Additional file 4.pdf]

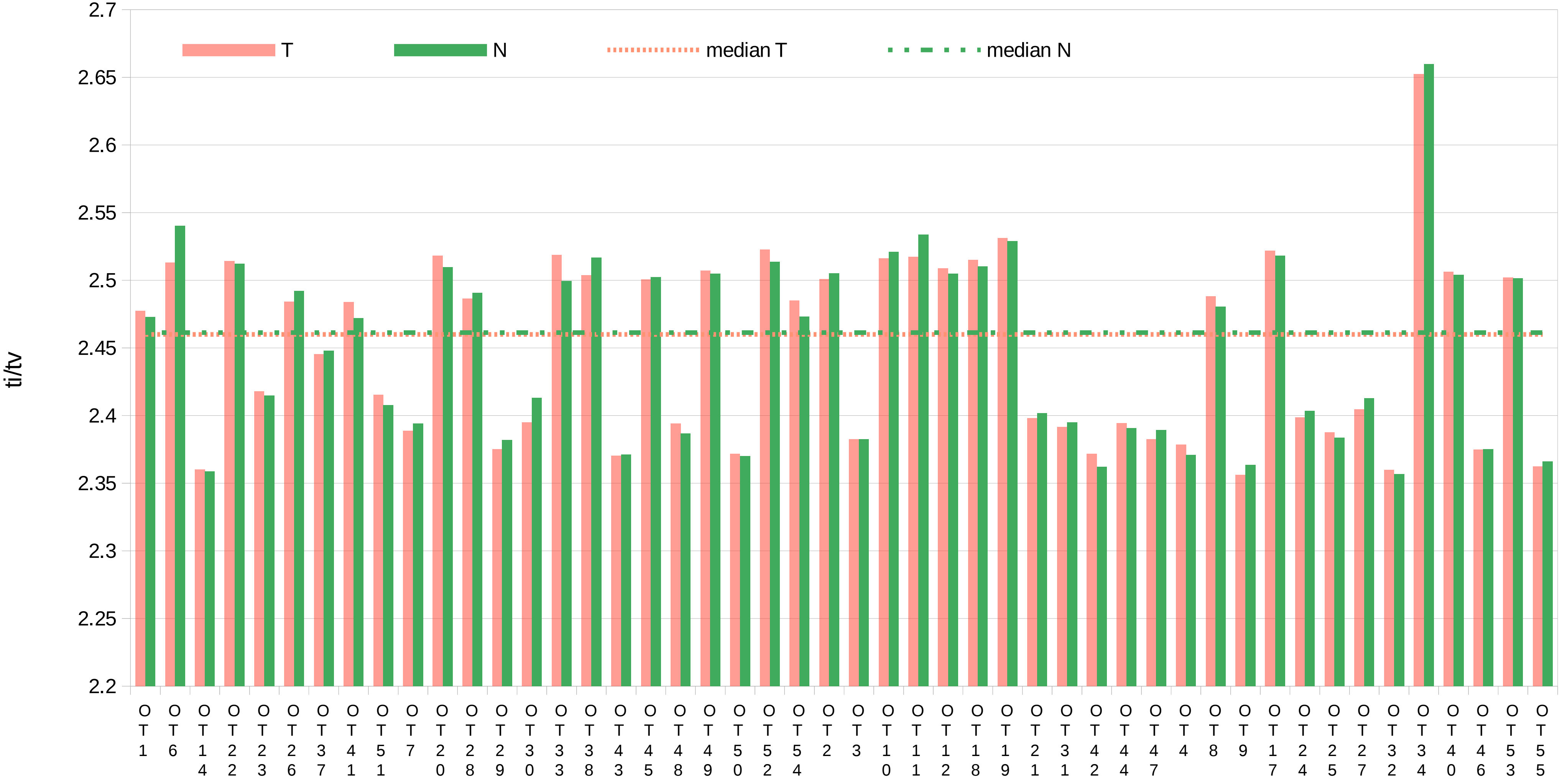
ti/tv
T
N
median T
median N
2.7
2.65
2.6
2.55
2.5
2.45
2.4
2.35
2.3
2.25
2.2
OT1
OT6
OT14
OT22
OT23
OT26
OT37
OT41
OT51
OT7
OT20
OT28
OT29
OT30
OT33
OT38
OT43
OT45
OT48
OT49
OT50
OT52
OT54
OT2
OT3
OT10
OT11
OT12
OT18
OT19
OT21
OT31
OT42
OT44
OT47
OT4
OT8
OT9
OT17
OT24
OT25
OT27
OT32
OT34
OT40
OT46
OT53
OT55
