# Supplementary material for: Integrated analysis of oral tongue squamous cell carcinoma identifies key variants and pathways linked to risk habits, HPV, clinical parameters and tumor recurrence
Source: F1000Res. 2015 Nov 5;4:1215. [Version 1] doi: 10.12688/f1000research.7302.1 (PMC4706066; doi:10.12688/f1000research.7302.1)
Supplement: Supplementary file 1 [file f1000research-4-7870-s0000.tgz › 4007fd05-e466-4e59-a4ef-512f9d41f650.zip!/Additional files/Additional file 5.pdf]

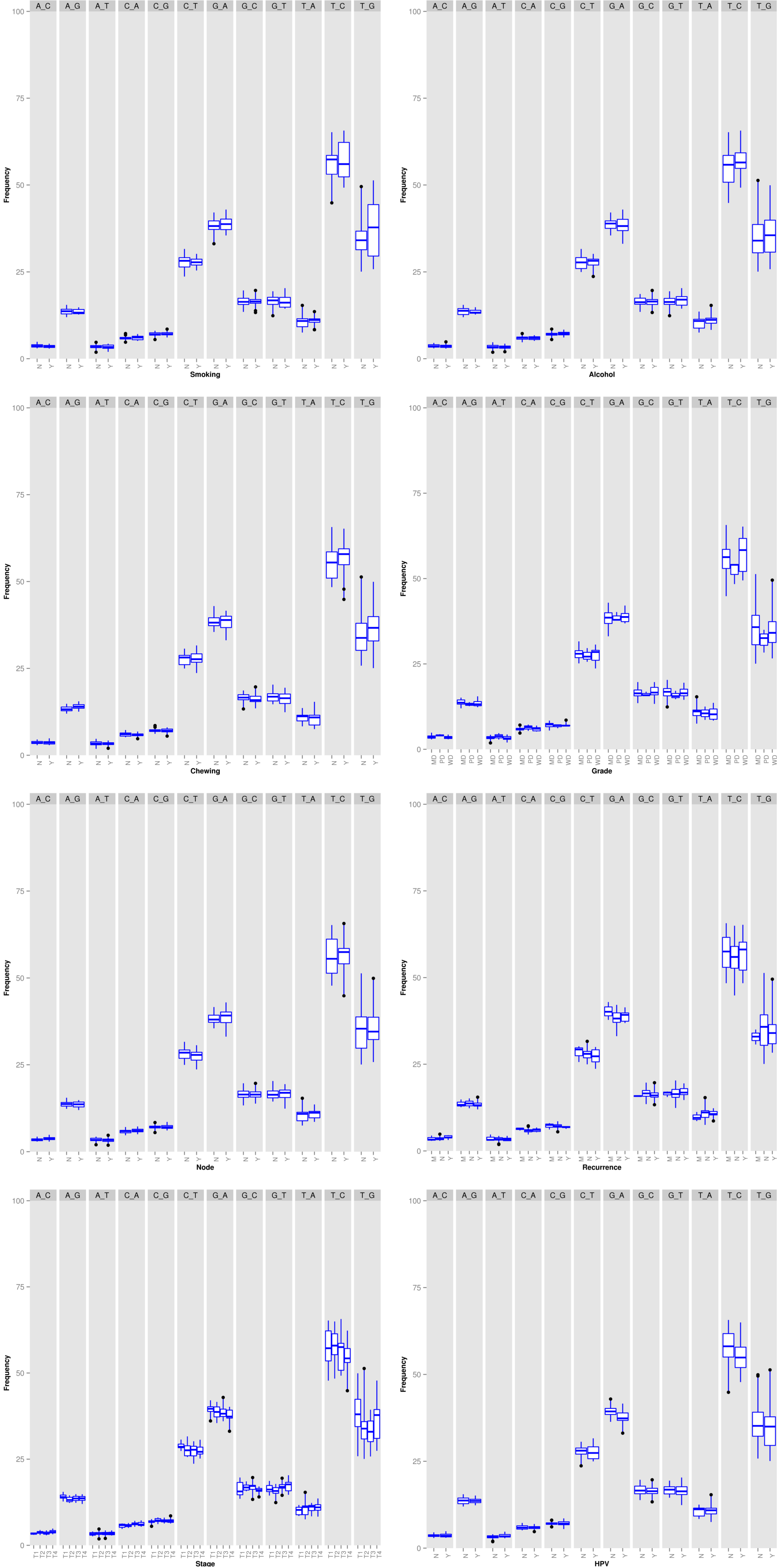
A_C
A_G
A_T
C_A
C_G
C_T
G_A
G_C
G_T
T_A
T_C
T_G
Frequency
0
25
50
75
100
N
Y
Smoking
Alcohol
Chewing
MD
PD
WD
Grade
Node
M
Recurrence
T1
T2
T3
T4
Stage
HPV
