# Supplementary material for: Integrated analysis of oral tongue squamous cell carcinoma identifies key variants and pathways linked to risk habits, HPV, clinical parameters and tumor recurrence
Source: F1000Res. 2015 Nov 5;4:1215. [Version 1] doi: 10.12688/f1000research.7302.1 (PMC4706066; doi:10.12688/f1000research.7302.1)
Supplement: Supplementary file 1 [file f1000research-4-7870-s0000.tgz › 4007fd05-e466-4e59-a4ef-512f9d41f650.zip!/Additional files/Additional file 8.pdf]

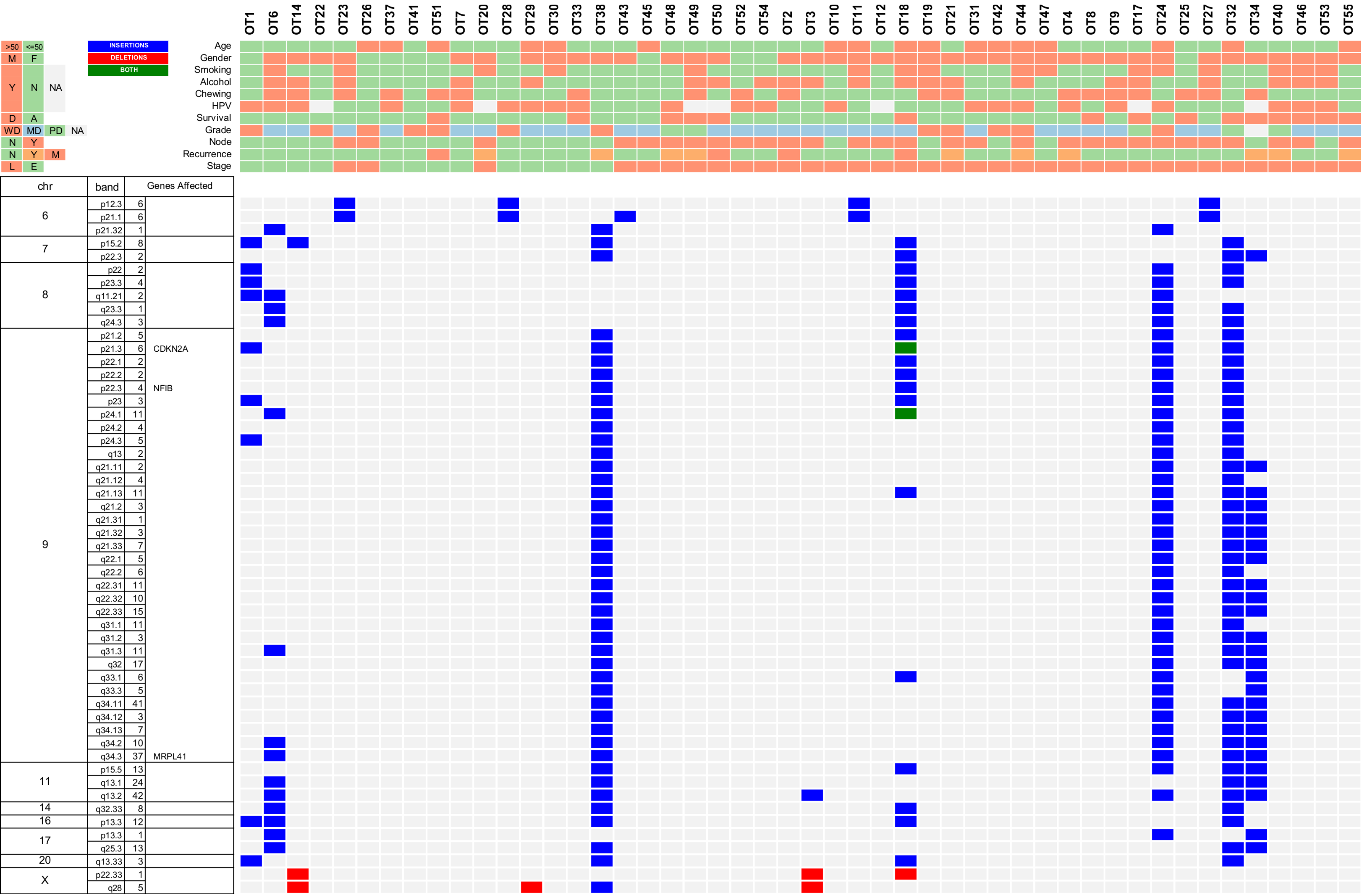

OT1 OT6 OT14 OT22 OT23 OT26 OT37 OT41 OT51 OT7 OT20 OT28 OT29 OT30 OT33 OT38 OT43 OT45 OT48 OT49 OT50 OT52 OT54 OT2 OT3 OT10 OT11 OT12 OT18 OT19 OT21 OT31 OT42 OT44 OT47 OT4 OT8 OT9 OT17 OT24 OT25 OT27 OT32 OT34 OT40 OT46 OT53 OT55
>50 <=50
M F
Y N NA
D A
WD MD PD NA
N Y
N Y M
L E
INSERTIONS
DELETIONS
BOTH
Age
Gender
Smoking
Alcohol
Chewing
HPV
Survival
Grade
Node
Recurrence
Stage
chr
band
Genes Affected
6
p12.3 6
p21.1 6
p21.32 1
7
p15.2 8
p22.3 2
8
p22 2
p23.3 4
q11.21 2
q23.3 1
q24.3 3
9
p21.2 5
p21.3 6 CDKN2A
p22.1 2
p22.2 2
p22.3 4 NFIB
p23 3
p24.1 11
p24.2 4
p24.3 5
q13 2
q21.11 2
q21.12 4
q21.13 11
q21.2 3
q21.31 1
q21.32 3
q21.33 7
q22.1 5
q22.2 6
q22.31 11
q22.32 10
q22.33 15
q31.1 11
q31.2 3
q31.3 11
q32 17
q33.1 6
q33.3 5
q34.11 41
q34.12 3
q34.13 7
q34.2 10
q34.3 37 MRPL41
11
p15.5 13
q13.1 24
q13.2 42
14
q32.33 8
16
p13.3 12
17
p13.3 1
q25.3 13
20
q13.33 3
X
p22.33 1
q28 5
