# Supplementary material for: Integrated analysis of oral tongue squamous cell carcinoma identifies key variants and pathways linked to risk habits, HPV, clinical parameters and tumor recurrence
Source: F1000Res. 2015 Nov 5;4:1215. [Version 1] doi: 10.12688/f1000research.7302.1 (PMC4706066; doi:10.12688/f1000research.7302.1)
Supplement: Peer review history

# Peer review: Referee response for version 1

**DOI:** 10.5256/f1000research.7870.r11116

**Published:** 2015-12-17

**Authors:** Thomas Carey

**Affiliations:** 1 Laboratory of Head and Neck Center Biology, Department of Otolaryngology-Head and Neck Surgery, University of Michigan, Ann Arbor, MI, USA

This article reports the the genetic analysis of 50 oral tongue primary tumor cancers treated at a single center, listed as paired sets. The study appears to be well done and thorough with carefully performed informatics.  

In the abstract it is stated that 50 paired primary oral tongue cancers were studied, but doesn't state whether the tumors were paired to normal blood or other tissue.

The analysis includes single nucleotide variations, copy number variations, indels, regions with loss of heterozygosity. These somatic variations are linked to clinical parameters. In addition HPV was assessed by q-PCR and found to have a very high rate of positivity, which is surprising given the reported site of oral tongue. It was not clear if all HPV positive cases also had p16 expression. It would be valuable for the reader to know what portions of the HPV transcriptome was assessed. Did the authors examine E6 and E7 oncogene expression? It is questionable whether the HPV is a driver in 22 of 50 tumors. It would be important to verify the activity of HPV by assessing viral oncogene expression and level of expression. In tumors with HPV and mutant p53 it is unlikely that those are driven by HPV, given that p53 mutations in other HPV positive head and neck tumors is extremely rare. It would be helpful to have a table that shows how often mutant p53 was found in the HPV positive tumors and whether those patients had excessive use of carcinogenic substances. 

There are some novel findings in this study and some very surprising correlations, for example, CDKN2A mutations were found only in non-smokers. This is highly surprising given that in the US abnormalities of this locus is common in head and neck cancers in smokers. Thus, grouping never smokers and former smokers may not be a fair grouping. What does former smoker mean in this population?  

Only smoking and oral tobacco and alcohol use are discussed as etiologic factors. Are there no other factors in this part of India? Is betel nut or oral tobacco mixed with other substances included in the oral tobacco use category? Since these are all oral tongue cancers the information about etiology should be made more clear.  

The mutual exclusivity of several genetic variants is interesting but needs validation.

The followup period is fairly short for the clinical correlates and relatively few patients recurred.

Inclusion of a table of the clinical characteristics showing a breakdown of the T-class, N-class stage and outcome would be helpful to evaluate the clinical outcome and the association with the minimal gene signature. However, the non-recurrent tumors have minimal genetic changes, whereas the recurrent and metastatic tumors are far more complex. So the low genetic complexity alone may be a marker of good outcome rather than the minimal gene signature described for poor outcome. Addition of the primary tumor size, nodal status and stage would be informative on figure 5, which shows the minimal signature set for tumor recurrence.

I have read this submission. I believe that I have an appropriate level of expertise to confirm that it is of an acceptable scientific standard.

# Peer review: Referee response for version 1

**DOI:** 10.5256/f1000research.7870.r11114

**Published:** 2015-11-09

**Authors:** Nishant Agrawal

**Affiliations:** 1 Ludwig Center for Cancer Genetics and Therapeutics, The Johns Hopkins University, Baltimore, MD, USA

The authors report an integrated genetic, epigenetic, and expression analysis from 50 oral tongue SCC. The findings were confirmed using a data set from TCGA. The manuscript presents surprising data which is interesting and has potential clinical implications. 

1. It is surprising that 23 of 50 patients with oral tongue SCC are HPV/p16 positive. This is not entirely consistent with previously published literature. Although the authors comment on this, this finding is rather “unique.”
2. It is very possible that the improved survival is due to HPV status and not mutation status as these results maybe confounded. 
3. Although 50 patients is a relatively large sample size for such a study, a much larger study is necessary to really have immediate clinical impact.

I have read this submission. I believe that I have an appropriate level of expertise to confirm that it is of an acceptable scientific standard.
